# Size and Polarizability as Design Principles for Stereoselective Catalysis

**DOI:** 10.1002/chem.202502741

**Published:** 2025-11-02

**Authors:** Caroline M. Carter, Jan Brossette, Hendrik Zipse

**Affiliations:** ^1^ Department of Chemistry LMU Munich Butenandtstraße 5–13 81377 Munich Germany

**Keywords:** isotropic polarizability, London dispersion forces, molecular volume, noncovalent interactions, stereoselective catalysis

## Abstract

Molecular volumes and isotropic polarizabilities of a larger number of carbocyclic π‐systems ranging from benzene on the small to heptacene on the large end are provided in order to facilitate the analysis of positively size‐dependent catalytic processes, where an increase in substrate or catalyst size leads to an increase in reaction rate and/or reaction stereoselectivity. The utility of this approach is demonstrated for selected stereoselective catalytic processes from the contemporary literature.

## Graphical abstract

In recent years, noncovalent interactions (NCIs) have been recognized as a key component in substrate activation and control in stereoselective catalysis. We demonstrate that size and polarizability serve as powerful, yet simple descriptors for catalyst and substrate design on a quantitative level.

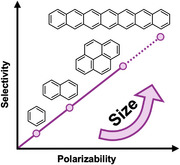

## 1 Introduction

**Figure 1:**
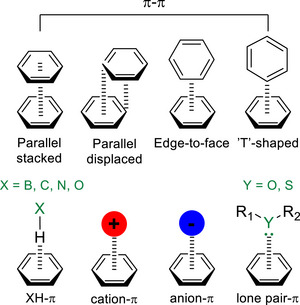
Different types of noncovalent interactions involving π‐systems.

Noncovalent interactions (NCIs), especially London dispersion (LD) interactions, have emerged as a hot topic in molecular chemistry.^[^ ^1^, ^2^ ^]^ In molecular catalysis LD interactions have been increasingly exploited as an attractive force to enhance catalyst selectivity and effectiveness.^[^ ^3^, ^4^, ^5^, ^6^ ^]^ Various classes of catalysts such as chiral phosphoric acids (CPAs),^[^ ^7^ ^]^ ureas,^[^ ^8^ ^]^ or pyridines,^[^ ^9^ ^]^ have successfully utilized NCIs and LD interactions to achieve enhanced stereochemical control. Among these, NCIs involving π‐systems represent one of the most prevalent interaction types, appearing in different forms such as π‐π stacking, XH–π interactions (X = B, C, N, O), cation‐π, anion‐π or lone pair‐π (Figure 1)^[^ ^10^, ^11^ ^]^

Particularly, cation‐π interactions^[^ ^12^, ^13^ ^]^ have recently gained recognition as an important directing effect in catalysis.^[^ ^14^ ^]^ Moreover, the strength of these cation‐π interactions systematically increases with the size^[^ ^15^ ^]^ and the polarizability of the of the π‐system.^[^ ^16^ ^]^ That this may serve as a design principle for catalysis will be demonstrated in the following. While polarizability has been partially recognized as an important property in catalysis,^[^ ^17^, ^18^ ^]^ and particularly in enantioselective transformations,^[^ ^6^, ^19^, ^20^ ^]^ we here aim to highlight its full range of potential effects. As quantitative data‐driven approaches^[^ ^21^, ^22^, ^23^, ^24^ ^]^ such as machine learning (ML)^[^ ^25^, ^26^ ^]^ or quantitative structure‐selectivity relationships (QSSR)^[^ ^27^ ^]^ have emerged as powerful tools for catalysis research, we also want to highlight size and polarizability as useful descriptors in model development.

Polarizability can be most effectively enhanced by increasing the size of catalyst side‐chains, particularly through the incorporation of larger aryl substituents, which strengthen aryl‐π interactions. Recent efforts to test quantitative models for their ability to reproduce experimentally observed selectivity data have indicated that side‐chain molecular volumes and side‐chain polarizabilities (either on the substrate or the catalyst side) are well suited for this purpose.^[^ ^4^, ^6^, ^9^ ^]^ However, application of this type of reactivity/selectivity analysis often hinges on the availability of molecular volume and polarizability data for aromatic side‐chains of variable size. In the following we therefore describe a dataset focused on aromatic side‐chains of variable size with the goal of facilitating the analysis of size‐dependent phenomena in catalytic processes, the development of descriptor‐based reactivity models, and the use of these models for substrates and catalysts decorated with uncommonly large side‐chains.

## 2 Size and Polarizability as Descriptors

### 2.1 Benzenoid π‐Systems

**Figure 2:**
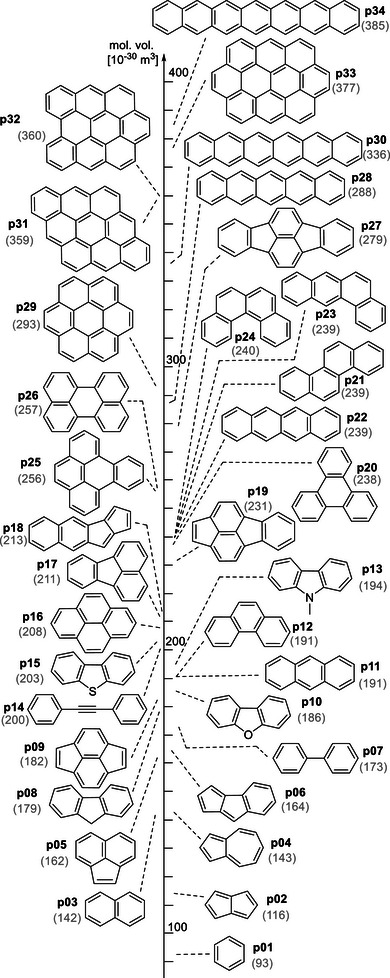
Molecular volume parameters (in units of 10^−30^ m^3^) for selected carbo‐ and heterocyclic π‐systems **p01 **– **p34** calculated at the SMD(Et_2_O)/D3‐B3LYP/6–31+G(d) level of theory.

Molecular volume data for a library of carbo‐ and heterocyclic π‐systems (**p01**–**p34**) ranging from benzene (**p01**) on the small to heptacene (**p34**) on the large end is shown in graphical manner in Figure 2. The molecular volumes are calculated with the GEPOL algorithm^[^ ^28^ ^]^ employed in PCM‐type continuum solvation models such as SMD^[^ ^29^ ^]^ for the construction of solute cavities. We select here Et_2_O as the solvent in order to match kinetic resolution experiments performed in this medium,^[^ ^9^, ^30^, ^31^, ^32^, ^33^ ^]^ but also note that closely similar molecular volumes are obtained for other organic solvents of low/medium polarity. The molecular volumes are given in units of 10^−30^ m^3^, which is identical to Å^3^. From the basically identical volumes of anthracene (**p11**, 191x10^−30^ m^3^) and phenanthrene (**p12**, 191x10^−30^ m^3^), we see that different modes of annelating benzene rings do not lead to notably different molecular volumes. This is also seen for the systems **p20**–**p24**, all of which have volumes of approximately 239x10^−30^ m^3^.

**Figure 3:**
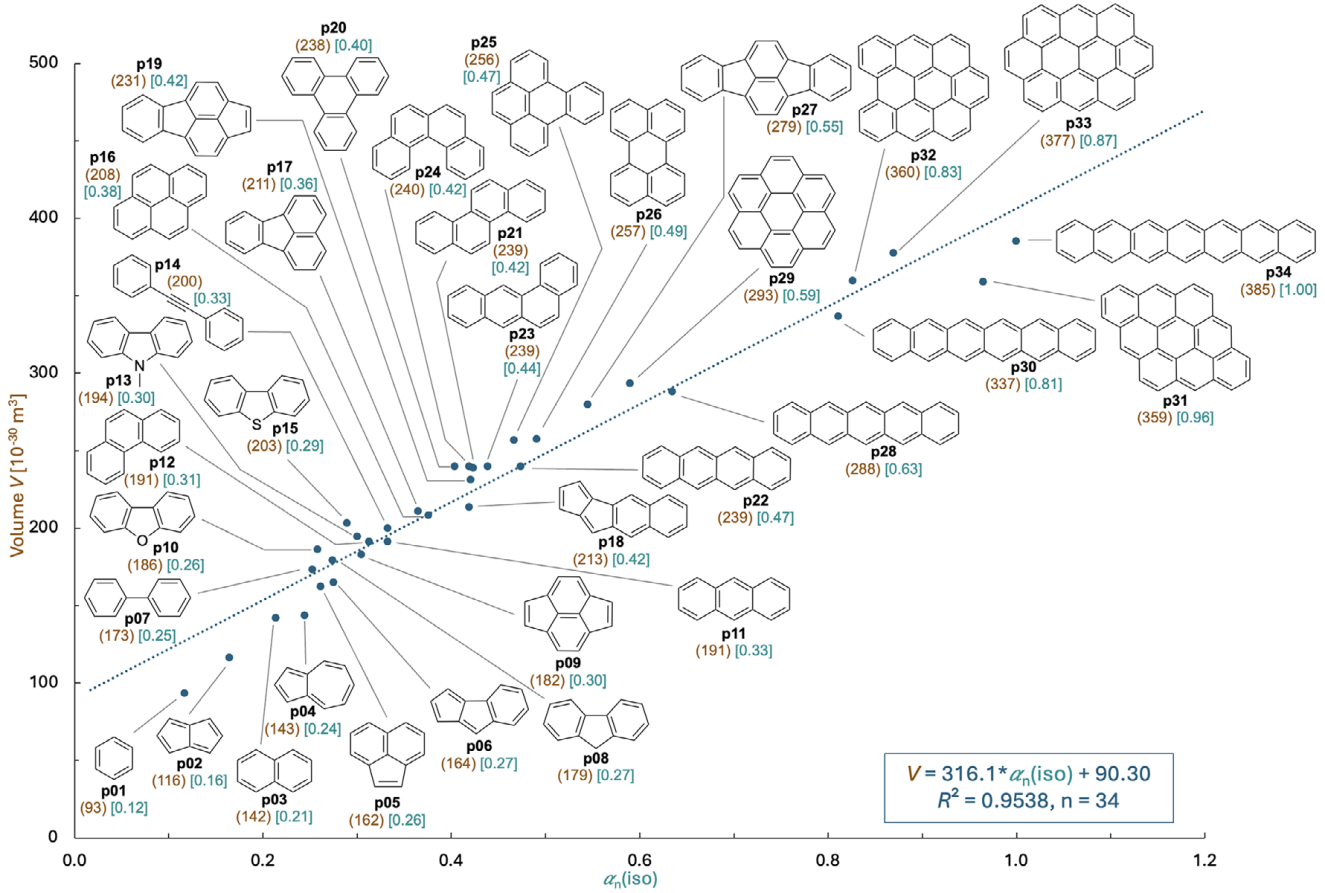
Correlation between molecular volumes *V* (in 10^−30^ m^3^) and normalized isotropic polarizabilities (*α*_n_(iso)) for 34 aryl systems **p01 **– **p34** calculated at the SMD(Et_2_O)/D3‐B3LYP/6–31+G(d) level of theory.

One possible reason for the positive relationship between system size and reaction rate/reaction selectivity may be due to the size‐dependent isotropic molecular polarizability *α*(iso) of π‐systems. This property describes the response of a molecular system to an external electric field, and correlates positively with NCIs such as LD or cation‐π interactions.^[^ ^12^, ^34^ ^]^ From a technical perspective, the calculation of molecular polarizabilities is more demanding as compared to the calculation of molecular volumes. Some of the associated challenges can be addressed by using normalized values *α*_n_ referenced to heptacene (**p34**) as the largest system considered in this study, rather than absolute molecular polarizability values *α* (see Supporting Information for details). The normalized polarizabilities thus range from *α*_n_ = 1.0 for heptacene (**p34**) down to *α*_n_ = 0.12 for benzene (**p01**). How well system size and polarizability values *α*_n_ correlate is shown in Figure 3 for all π‐systems presented in Figure 2. A good linear correlation with *R* ^2^ = 0.9538 is observed, in full agreement with the expected relationship between these two quantities. The formal generation of N‐heterocycles by substitution of carbon atoms with nitrogen reduces the isotropic polarizability without significantly affecting molecular volume (see Supporting Information for further details). While molecular volume remains a strong predictor of polarizability across heteroatom‐containing aryl systems (N, O, or S), N‐substituted rings consistently show higher polarizability than O or S analogs. As exemplified by N‐methylcarbazole (**p13**) and dibenzofuran (**p10**), heterocyclic systems may differ not only in the choice of heteroatom (e.g., N vs. O), but also in the number of attached substituents (such as the methyl group in **p13**).

### 2.2 Aryl System Variation

Often π‐systems connect to substrate or catalyst scaffolds through C(sp^3^) link atoms. To enable a comprehensive analysis of the influence of these link atoms, data for the 34 selected π‐systems were complemented with those for the respective methyl‐substituted derivatives. The addition of a methyl group (systems **01m** – **029m** in SI) to the π‐system consistently increases the molecular volume by 17.72x10^−30^ m^3^ and the isotropic polarizability by 2.34x10^−30^ m^3^ regardless of the position of attachment. These incremental changes are so systematic that correlations of experimental results with data for the methyl‐substituted π‐system will be closely similar to correlations with data for the π‐system themselves. Beyond changing the polarizability of a given side chain, the placement of C(sp^3^) link atoms will also impact the orientation and the conformational flexibility of the side chain relative to the reacting substrates. This latter effect may actually dominate in cases where notable differences in reaction rates or reaction selectivity are observed for catalysts differing in the point of attachment of a given π‐systems.^[^ ^35^, ^36^ ^]^ Volume/polarizability data for secondary alcohols containing π‐systems frequently employed in enantioselective transformations such as kinetic resolutions^[^ ^9^, ^37^ ^]^ have also been computed in order to analyze the difference between full substrates and the aryl side‐chain (see Supporting Information). The formal addition of EtOH to convert reference π‐systems into secondary alcohols (systems **1a** – **1d** and **1o** – **1q** in Supporting Information) increases the volume by 63.40 and the polarizability by 5.25x10^−30^ m^3^. These changes are again so systematic that using the parent π‐system as a simplified version of a substrate alcohol will give the same reliable and consistent correlation results. This is less so for substrate systems with multiple substitutions (such as alcohols **1** **g** – **1n**), where the simplification to the corresponding parent π‐system does not reflect the influence of the substitution pattern on size and polarizability.

## 3 Applications

**Figure 4:**
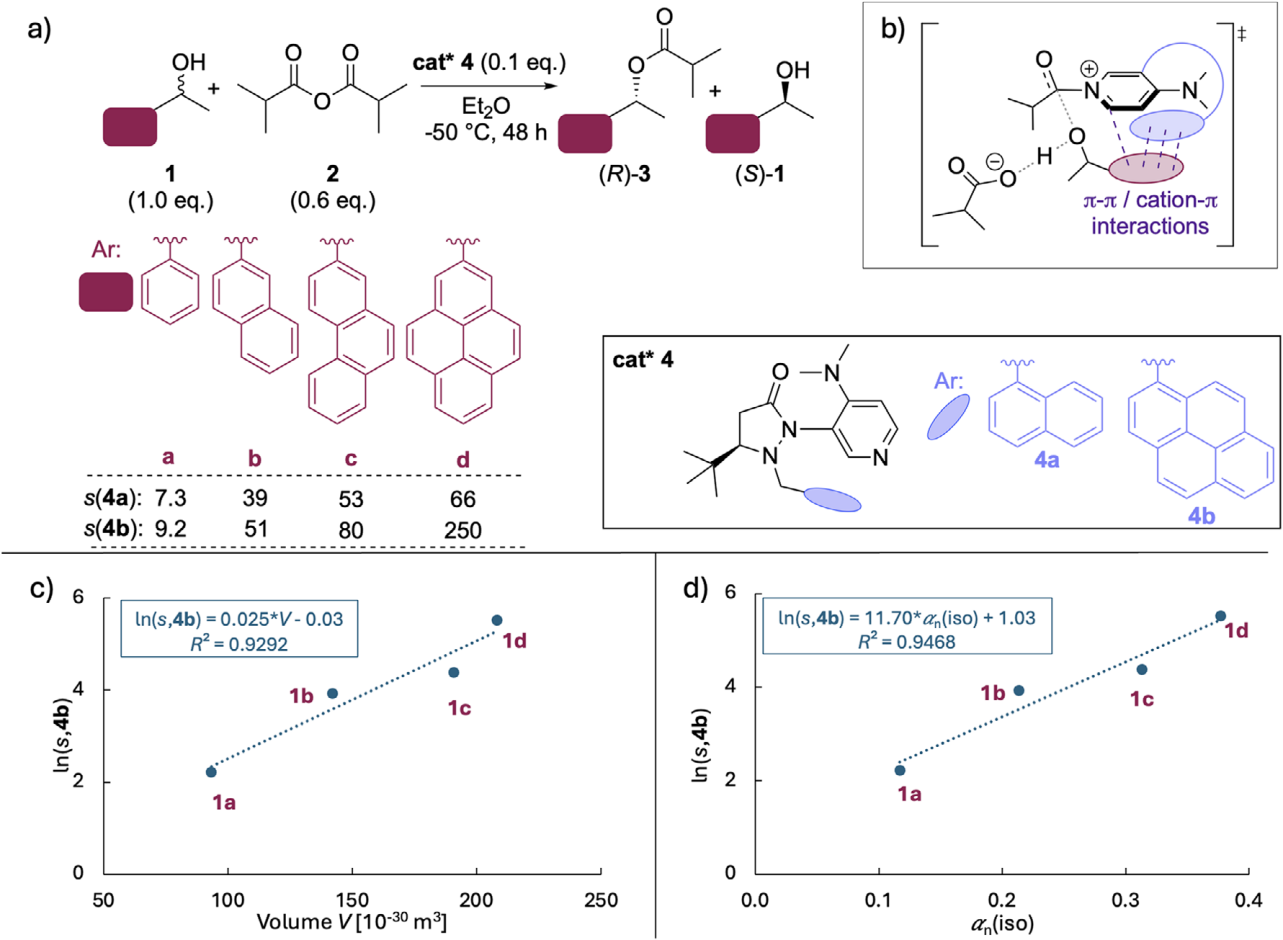
a) Kinetic resolution of alcohols **1a**–**1d** with isobutyric anhydride mediated by chiral pyridine catalysts **4a** or **4b**, with b) the corresponding transition state for the reaction, and c) correlation of ln(*s*,**4b**) for the reaction with alcohols **1a **– **1d** with aryl side‐chain molecular volume *V* and d) aryl side‐chain polarizability *α*_n_(iso).^[^ ^9^ ^]^ Selectivity *s* is defined as *s* = *k*_fas_ *_t_*/*k*_slow_ or here *s* = *k*_R_ */k*_S_.

With volume and polarizability data for a large number of (hetero)‐aromatic π‐systems in hand, we can explore possible relationships with the selectivity data shown in Figure 4. Studying the kinetic resolution of 1‐substituted ethanol substrates **1a** – **1d** with isobutyric anhydride (**2**) mediated by chiral Sibi‐type pyridine catalysts **4a** or **4b**, we have recently shown that reaction rates and reaction enantioselectivity respond positively to substrate size in the sense that fastest reaction rates and highest selectivities were measured for the largest substrate alcohol. Indeed, for catalyst **4b**, selectivity *s* values (with *s* = *k*_fast_/*k*_slow_ or here *s* = *k*_R_ */k*_S_) range from 9.2 for alcohol **1a** to 250 for **1d**.^[^ ^9^ ^]^ This enhanced selectivity is accompanied by a much larger increase in reaction rate for alcohols of (*R*)‐configuration as compared to alcohols of (*S*)‐configuration with, for catalyst **4b**, (*R*)‐*k*(**1d**)/(*R*)‐*k*(**1a**) = 44 and (*S*)‐*k*(**1d**)/(*S*)‐*k*(**1a**) = 1.6.^[^ ^9^ ^]^ In detailed quantum chemical studies of the rate‐ and selectivity‐determining step of this esterification reaction, we have shown that the side‐chain of catalyst **4a** acts on the substrate side‐chain in the transition state for the faster reacting alcohol *(R)*‐**1** (Figure 4b), but not in the transition state for the slower reacting alcohol *(S)*‐**1**.^[^ ^9^ ^]^

Given the larger range of numerical values, we focus on the results for catalyst **4b**. Expressing the reaction selectivity as the natural logarithm ln(*s*,**4b**), we obtain the two correlations shown in Figure 4. In Figure 4c, a positive correlation is observed between reaction selectivity and the molecular volume *V* of the aromatic substrate side‐chain with a slope of +0.025. This implies a strong dependence of reaction selectivity on side‐chain size, and the correlation coefficient is impressively good (*R* ^2^ = 0.9292), especially considering the simplicity of this single descriptor approach. A similarly good correlation is observed between reaction selectivity and the normalized polarizability *α*_n_ with a slope of +11.70, indicating a strong increase in selectivity with increasing polarizability (Figure 4d). The correlation coefficient is in this case even better (*R* ^2 ^= 0.9468). Using the molecular volume or polarizability of the full substrate alcohol and not only of the aromatic side‐chain offered marginal improvements in correlations, and we can thus conclude that using side‐chain properties alone is a suitable surrogate for evaluating the size‐dependent increase in selectivity and reactivity. As correlation coefficients were consistently found to be higher in our analysis compared to molecular volumes, subsequent correlations are presented using normalized isotropic polarizabilities *α*_n_ only. Literature examples of stereoselective catalytic processes, where side‐chain enlargement leads to enhancements of selectivity or reaction rate (or both), can conceptually be divided into two groups. The first examines the size‐variations of the catalyst side‐chain (Section 3.1), while the second focusses on varying substrate substituents (Section 3.2). The example presented in Figure 4 belongs to the second group of reactions.

### 3.1 Size‐Variation of Catalyst Side‐Chain

**Figure 5:**
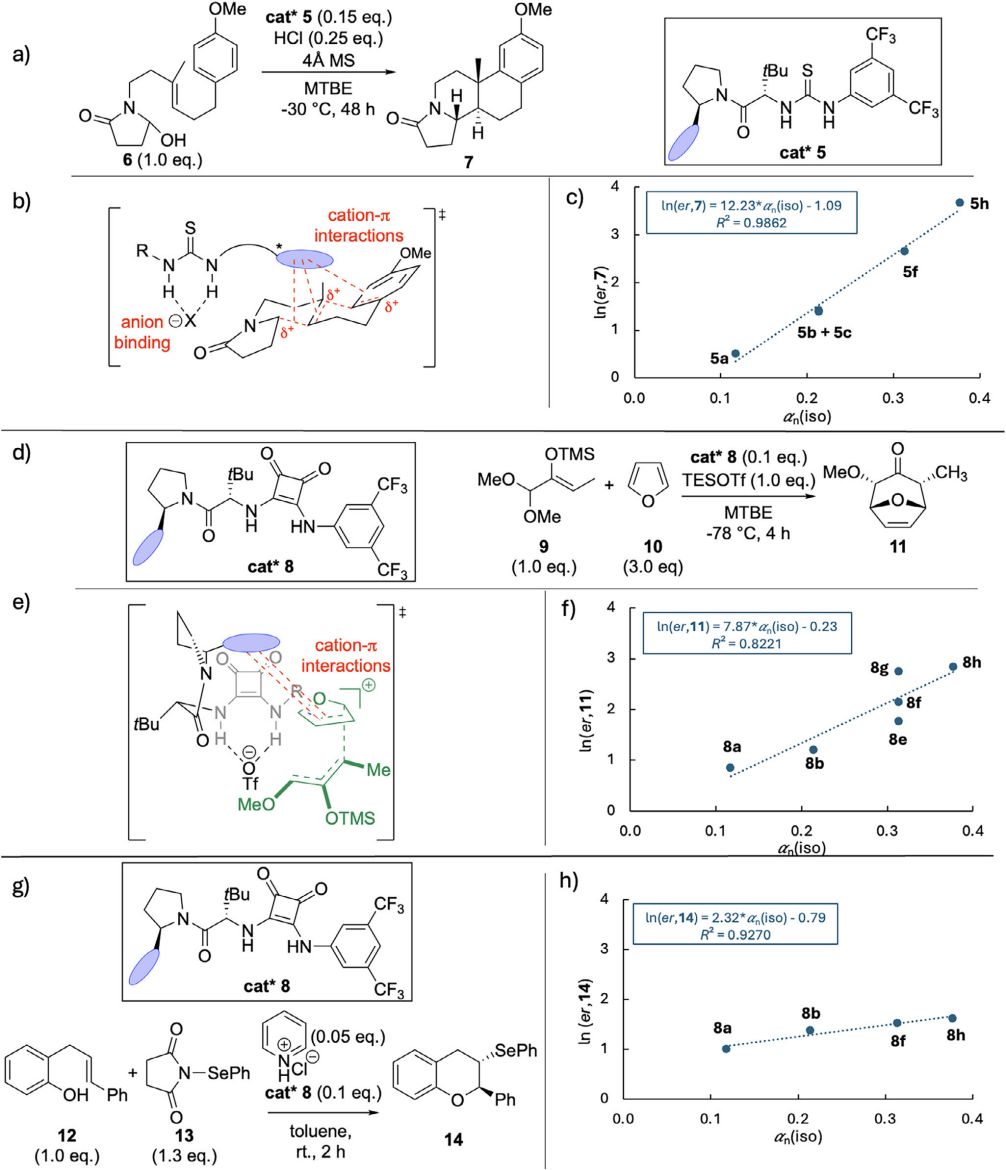
a) Enantioselective thiourea‐catalyzed cyclization of **6** mediated by catalyst **5**,^[^ ^8^ ^]^ with b) a sketch of the selectivity‐determining transition state, and c) correlation of product ln(*er*,**7**) with aryl side‐chain polarizability *α*_n_(iso).^[^ ^8^ ^]^ d) Enantioselective [4 + 3] cycloaddition of furan mediated by catalyst **8** with e) a sketch of selectivity‐determining transition state, and f) correlation of product ln(*er*,**11**) with aryl side‐chain polarizability *α*_n_(iso).^[^ ^35^ ^]^ g) Enantioselective cyclization of phenol **12** mediated by catalyst **8** and h) correlation of product ln(*er*,**14**) with aryl side‐chain polarizability *α*_n_(iso).^[^ ^38^ ^].^

In the first category falls the enantioselective thiourea‐catalyzed cyclization of alkene **6**, where larger aromatic side‐chains in the thiourea catalysts **5a** – **5e** lead to higher product *ee* values as well as higher yields of cyclized product **7** (Figure 5a and Table 1).^[^ ^8^ ^]^ These trends have been rationalized by Knowles and Jacobsen with a combination of two NCIs in the selectivity‐determining transition state illustrated in Figure 5b: a) hydrogen bonding interactions between a reagent‐derived anion and the thiourea‐fragment of catalyst **5**, and b) cation‐π interactions between the catalyst aromatic side‐chain and the positive charge delocalized over several centers of the reacting substrate, the size of which is expected to depend on the size of the catalyst π‐system. The latter idea has recently been invoked by Asensio et al.^[^ ^39^ ^]^ in studies probing the impact of pyrene‐based protecting groups on the rate and selectivity of glycosylation reactions. A clear trend on π‐system size has, however, not been observed in the glycosylations studied. After converting the experimentally measured *ee*(%) values into product enantiomeric ratios (*er*) (Table 1), a very good correlation of product ln(*er*) with side‐chain polarizabilities *α*_n_ (*R* ^2^ = 0.9862, Figure 5c) can be found. This latter finding is in agreement with the assumption that catalyst side‐chain effects on reaction selectivity and reaction rate are primarily rooted in attractive side‐chain/substrate interactions dominated by LD forces or other NCIs correlated to side‐chain polarizability. That the reaction yield also increases with selectivity is in agreement with this interpretation. The correlation slopes in Figure 4 and Figure 5c are very similar (around +12.00), confirming that both reactions undergo interactions between a large π‐system and a positive charge in the rate determining step. These findings are in accordance with the previously published correlation of side‐chain polarizability with ln(*er*), which stipulate that cation‐π interactions are the key driver of enantioselectivity.^[^ ^6^ ^]^ Catalyst **5** was also tested for the enantioselective thiourea‐catalyzed ring‐opening of episulfonium ions with indole, where increasing the size of the aryl system up to pyrene improved the reaction selectivity, while aryl systems, such as chrysene, lead to a slight decrease in enantioselectivity.^[^ ^40^ ^]^ The authors hypothesized that the larger systems may be hindered by steric or other factors which would reduce their participation in cation‐π interactions. Solubility for large aryl systems is widely known to be poor in organic solvents of low polarity and could also play a role in reduced performance.

**Table 1:** Structures of all catalyst aryl side‐chains used in Figure 5, Figure 6, and Figure 7, together with a summary of enantiomeric excess (*ee* in %), enantiomeric ratio (*er*), and yield (in %) for the formation of product **7** with catalyst **5**, products **11** and **14** with catalyst **8**, and of product **22** with catalyst **20**. For the formation of product **18** and **19** with catalyst **15** *er*, diastereomeric ratio (*dr*) and regioisomeric ratio (*rr*), and yield (in %) are included.

cat.			Catalyst aryl side‐chain:							
	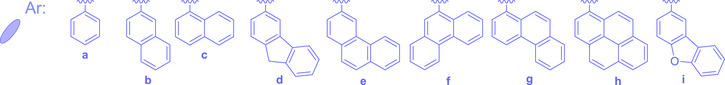									
**5** ^[^ ^8^ ^]^	%*ee*	25	61	60	‐	‐	87	‐	95	‐
	ln(*er*,**7**)	0.51	1.42	1.39	‐	‐	2.67	‐	3.66	‐
	%yield	12	33	46	‐	‐	52	‐	78	‐
**8** ^[^ ^35^ ^]^	%*ee*	40	54	‐	‐	71	79	88	89	‐
	ln(*er*,**11**)	0.85	1.21	‐	‐	1.77	2.14	2.75	2.84	‐
	%yield	49	56	‐	‐	69	76	74	82	‐
**8** ^[^ ^38^ ^]^	%*ee*	46	60	‐	‐	‐	64	‐	67	‐
	ln(*er*,**14**)	0.99	1.39	‐	‐	‐	1.52	‐	1.62	‐
	%yield	82	82	‐	‐	‐	88	‐	84	‐
**15** ^[^ ^41^ ^]^	ln(*rr*)	‐	0.36	‐	1.15	1.59	‐	‐	2.94	‐
	ln(*dr*)	‐	2.85	‐	1.39	1.82	‐	‐	3.48	‐
	ln(er,**18a**)	‐	0.90	‐	1.52	2.04	‐	‐	4.60	‐
	%yield	‐	86	‐	92	91	‐	‐	32	‐
**20** ^[^ ^42^ ^]^	%*ee*	60	78	‐	‐	‐	90	‐	‐	95
	ln(*er*,**22**)	1.39	2.09	‐	‐	‐	2.94	‐	‐	3.66
	%yield	28	48	‐	‐	‐	69	‐	‐	69

A second example in increasing the size of catalyst side‐chain concerns the enantioselective [4 + 3] cycloaddition of **9** with furan mediated by catalyst **8** reported by Jacobsen and coworkers (Figure 5d).^[^ ^35^ ^]^ Cation‐π interactions were shown to play a key role in the selectivity‐determining transition state illustrated in Figure 5e, and again we note an increase in product enantioselectivity and yield with increasing side‐chain size (Table 1).^[^ ^35^ ^]^ Quantitative analysis of these effects by using side‐chain polarizability as descriptor is shown in Figure 5f, which is in support of attractive side‐chain/substrate interactions as the origin of enhanced reaction enantioselectivity. Not included in these correlations is the catalyst variation without a substituent at the pyrrolidine C2 position (corresponding to Ar = H) with a product *ee* value of 6%. The lower fidelity of these correlations compared to those in Figure 5c likely reflects conformational effects, which may originate from variation in cation‐π overlap or steric factors, as the same (phenanthryl) substituent in catalysts **8e**–**8** **g** shows somewhat different selectivities in different orientations. Catalyst **8** (**8a**, **8b**, **8** **g**, **8** **h**) has also been employed by Jacobsen and coworkers in the stereoselective cyclization of phenol **12** (Figure 5g).^[^ ^38^ ^]^ Product **14** is once more formed in the highest selectivity when using catalyst **8** **h** with the largest (pyrenyl) side‐chain. Product enantiomeric ratios correlate again quite well with side‐chain polarizability (Figure 5h). For this particular example, molecular volume correlated slightly better than polarizability, while reaction yields are similarly good for all four catalysts studied.

**Figure 6:**
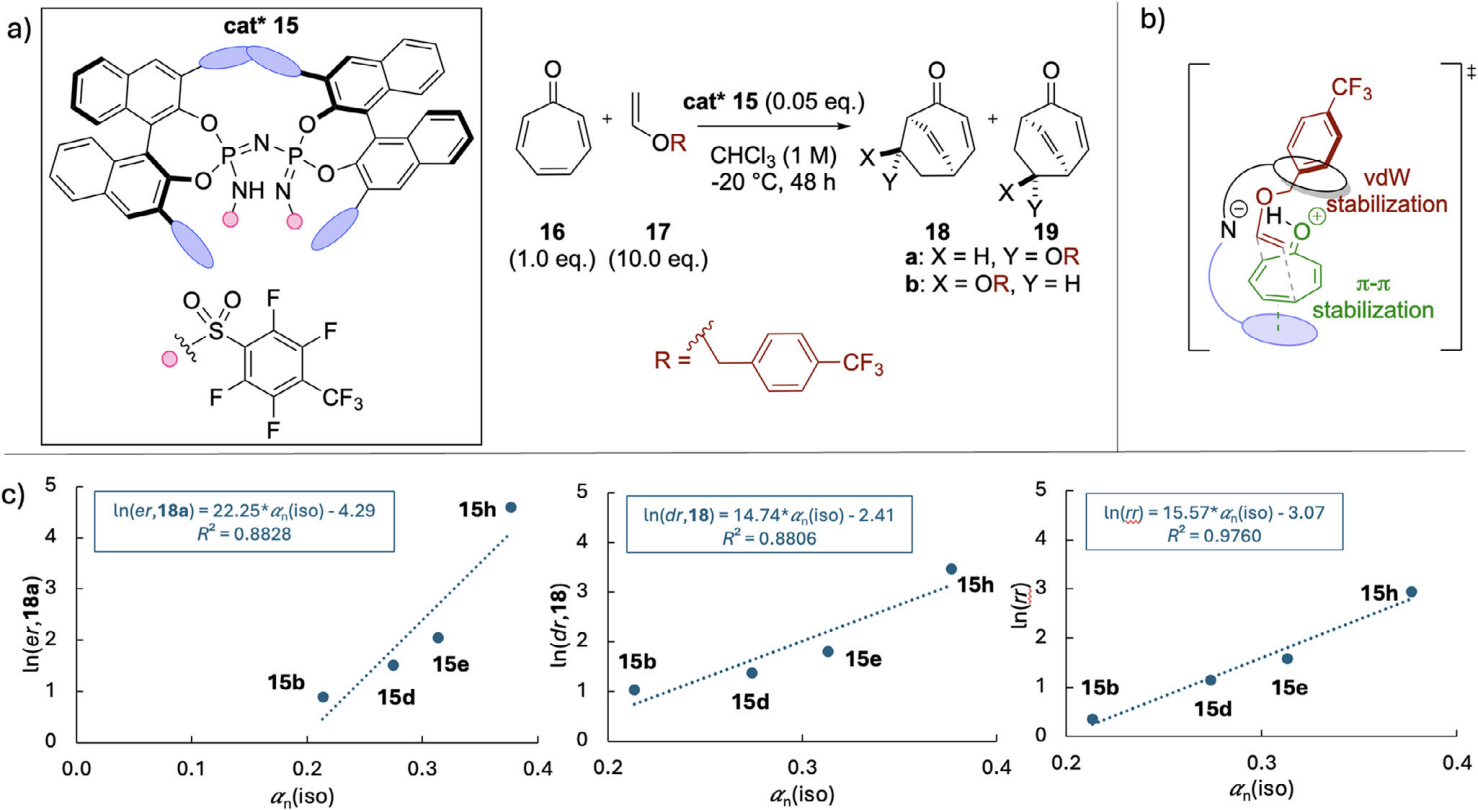
a) Enantioselective [4 + 2] cycloaddition of tropone (**16**) with vinyl ether **17** to form Umpolung product **18** and product **19** mediated by catalyst **15** with b) the transition state of the enantio‐ and rate‐determining steps where the catalyst aryl side‐chain of the catalyst performs π‐π stacking with **16** while its BINOL structure performs *van der Wals* interactions with the R group of **17**. c) Correlation of enantioselectivity (left), diastereoselectivity (middle) and regioisomeric ratio (right) with catalyst aryl side‐chain polarizability *α*_n_.^[^ ^41^ ^].^

Catalyst **15** was designed by List and coworkers to reverse the intrinsic regioselectivity of the [4 + 2] cycloadditions of tropone (**16**) with vinyl ether **17** as governed by frontier molecular orbital (FMO) theory to predominantly form the Umpolung cycloaddition product **18** over the FMO favored product **19** (Figure 6a).^[^ ^41^ ^]^ This reversal is attributed to enhanced NCIs between catalyst and substrates, specifically π‐π stacking and cation‐π interactions, with the use of larger aryl side‐chains such as pyrene (**15** **h**) over naphthalene (**15b**) as illustrated in the transition state sketch of the enantio‐ and rate‐determining step in Figure 6b.^[^ ^41^ ^]^ Variation of the size of the catalyst aryl side‐chain shows a correlation between side‐chain polarizability and ln(*er*) for product **18a** with an *R* ^2^ of 0.8828 (Figure 6c).^[^ ^41^ ^]^ Moreover, both regioisomeric ratio (*rr*) and diastereoselectivity (*dr*) were also found to correlate with side‐chain polarizability with *R* ^2^ values of 0.8806 and 0.9760, respectively.

**Figure 7:**
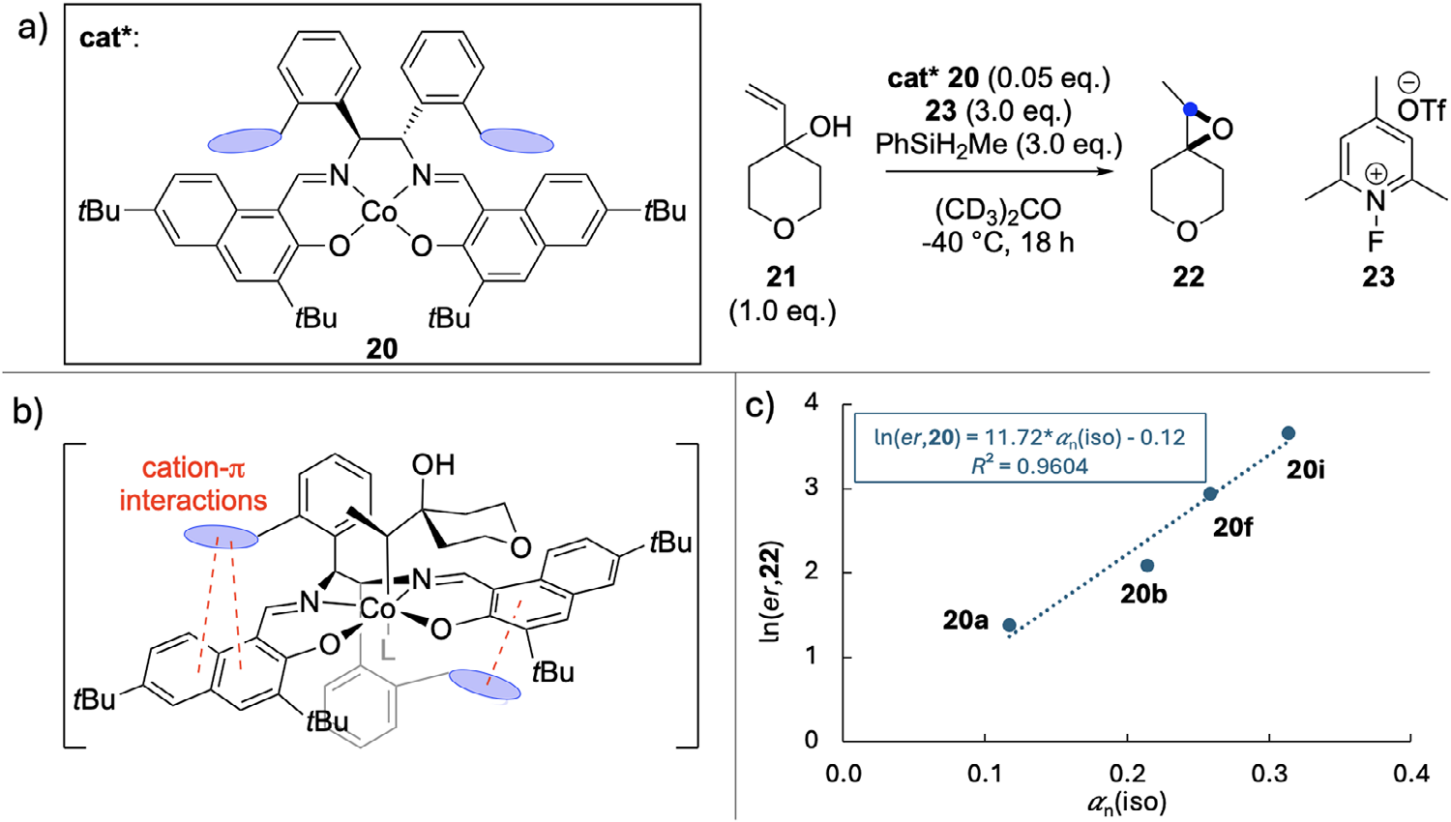
a) Enantioselective formation of epoxide **22** from **21** mediated by cobalt complex **20**. b) Proposed intermediate in the catalytic transformation (with L = solvent) with catalyst **20** linked to substrate **21** and the possible intracatalyst cation‐π interactions. c) Correlation of enantioselectivity (*er*) with catalyst aryl side‐chain polarizability *α*_n_(iso).^[^ ^42^ ^].^

This type of correlation can also be observed for transition metal‐mediated reactions, such as for cobalt complex **20** designed by Pronin and coworkers shown in Figure 7.^[^ ^42^ ^]^ This catalyst performs an enantioselective epoxidation of allylic tertiary alcohol **21** to product **22** via a radical‐polar crossover process triggered by hydrogen atom transfer (HAT). Varying the aryl side‐chain from phenyl **20a** to phenanthrene **20f** increased the reaction *ee* from 60 to 90%, while the incorporation of a heteroatom via the dibenzofuran system **20i** further increased the polarizability, resulting in a strong correlation between ln(*er*,**20**) and the side‐chain isotropic polarizability, with *R* ^2 ^= 0.9604. Temperature controlled experiments indicated that the enantioselectivity of epoxide formation is enthalpically controlled, with a suggested increase in cation‐π interactions with larger aryl systems as shown in Figure 7b. Preliminary experiments appear inconclusive as to whether the positive correlation shown in Figure 7c can be extended further to larger ligand systems. This likely indicates that additional factors are at play together with size and polarizability effects discussed here.^[^ ^42^ ^]^

In all examples described in Figure 4 – Figure 7, the enantioselective transformation was shown to positively correlate with an increase in isotropic polarizability of the catalyst side‐chain. This can best be understood by assuming that in all these transformations, the transition states for formation of the major enantiomer are stabilized through cation‐π interactions (rather than invoking destabilization of the transition states for formation of the minor enantiomer through repulsive steric effects). Experimental validation of this hypothesis requires the determination of reaction rates, which are expected to increase with reaction selectivity and catalyst size (see ref. [4] for a general discussion of this point). That the correlation of catalyst size with reaction rates is not explored too often is simply due to the scarcity of rate data (see refs. [9] and [40] for examples).

### 3.2 Size Variations of Substrate Side‐Chain

**Figure 8:**
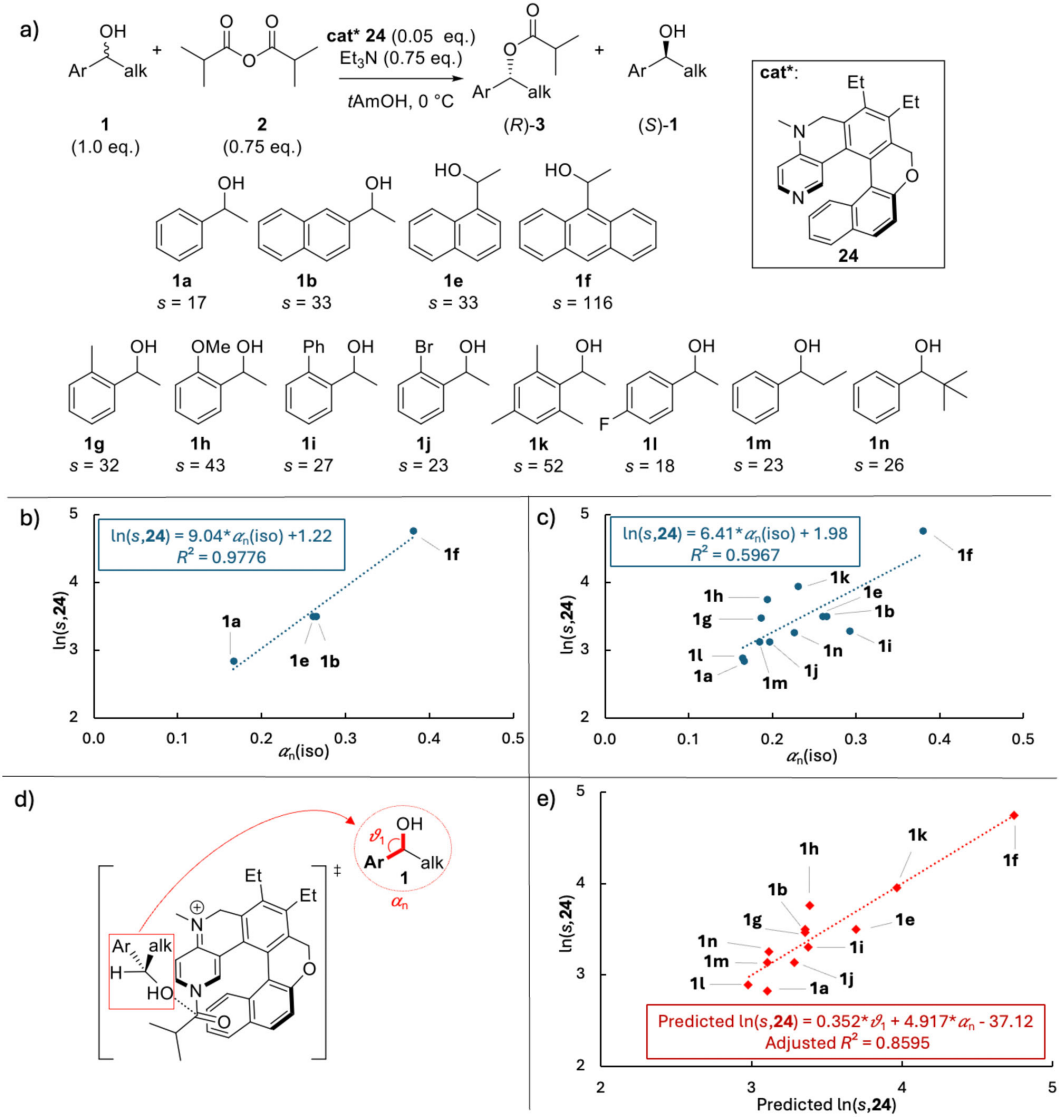
a) Enantioselective acylation of secondary alcohols (**1a **– **1n**) mediated by catalyst **24**. Correlation of ln(*s*,**24**) with the normalized isotropic polarizability *α*_n_(iso) of alcohol substrates for b) alcohols **1a**, **1b**, **1e**, and **1f**; and c) all 12 alcohols. d) Transition state of the acylation step as proposed by Carbery and coworkers.^[^ ^37^ ^]^ e) Dual linear regression analysis and plot of predicted ln(*s*,**24**) against experimental ln(*s*,**24**) values using two descriptors: the angle *θ*_1_ between the alcohol and the aryl‐group attachment carbon (highlighted in red), and the normalized isotropic polarizability *α*_n_ of the whole molecule.

The helical Lewis‐base catalyst **24** was designed by Carbery and coworkers for the kinetic resolution of secondary alcohol **1** (Figure 8).^[^ ^37^ ^]^ Increasing the size of the aryl side‐chain from phenyl to naphthyl, and then anthracene increases selectivity from *s *= 17 for **1a**, *s* = 33 for **1b** and **1e**, and *s* = 116 for **1f**.^[^ ^37^ ^]^ An excellent correlation between experimental ln(*s*,**24**) and the isotropic polarizability of the substrate side‐chain was obtained with *R* ^2^ = 0.9776 (Figure 8b). This effect was similarly also observed for the Sibi catalyst as shown in Figure 4.^[^ ^9^ ^]^ However, this excellent correlation only results from a careful selection of the substrate with increasing π‐systems and the absence of other substituents. Indeed, the correlation for all alcohols shown in Figure 8a yields a lower correlation coefficient with *R* ^2^ = 0.5967 (Figure 8c), indicating that a simple one descriptor model is insufficient for the complete set of substrate alcohols, and that a multidescriptor model may be required for better predictions. The angle *θ*_1_ between the alcohol and the aryl‐group attachment carbon (highlighted in red with the reaction's proposed transition state in Figure 8d), is one of the geometric descriptors considered in combination with charge‐ and polarizability‐dependent descriptors in building a two‐descriptor model (see Supporting Information for details). As shown in Figure 8e, linear combination of the values of angle *θ*_1_ with the normalized isotropic polarizability of the alcohols substrates **1** yields a useful dual descriptor model with a good correlation between predicted and experimental ln(*s*,**24**) values with an adjusted *R* ^2^ = 0.8595. This illustrates the utility of isotropic polarizability data in the development of dual‐ or multi‐descriptor models in stereoselective catalysis.

## 4 Summary and Outlook

The analysis of positively size‐dependent catalytic processes, where an increase in substrate or catalyst size leads to an increase in reaction rate and/or reaction stereoselectivity, can be facilitated through correlations with size or polarizability values of either the side‐chains themselves or the complete substrates. The value of this type of analysis clearly lies in its ability to predict the outcome of reactions with further size increases in a straight‐forward manner.

## Supporting Information

The size and polarizability data used in this manuscript have been deposited on the Zenodo platform (DOI: 10.5281/zenodo.8234174). The authors have cited additional references within the Supporting Information.

## Conflict of Interest

The authors declare no conflict of interest.

## Supporting information



Supporting Information

## Data Availability

The data that support the findings of this study are available in the supplementary material of this article.
