# Supplementary material for: Size and Polarizability as Design Principles for Stereoselective Catalysis
Source: Chemistry. 2025 Nov 2;31(68):e02741. doi: 10.1002/chem.202502741 (PMC12679343; doi:10.1002/chem.202502741)
Supplement: Supplementary file 1 — Supporting Information [file CHEM-31-e02741-s001.pdf]

Supplementary Information

Size and Polarizability as Design Principles in Enantioselective Catalysis

C. Carter[a], J. Brossette[a], H. Zipse*[a]

[a] C. M. Carter, J. Brossette, Prof. Dr., H., Zipse
Department of Chemistry
LMU Munich
Butenandtstraße 5–13, 81377, Munich, Germany
E-mail: zipse@cup.uni-muenchen.de

Table of Contents

1. Methods ... 2
   1.1. Conformer generation: ... 2
   1.2. Procedure for the calculation of polarizabilities ... 2
2. Calculation Results ... 3
   2.1. Carbocyclic systems ... 3
   2.2. Methylated aryl systems ... 5
   2.3. Secondary aryl alcohols ... 9
   2.4. Heteroaromatic systems ... 12
3. Volume and polarizability scales ... 13
4. Correlation analysis ... 16
   4.1. Carbocyclic systems ... 16
   4.2. Effects of methyl substitution ... 21
   4.3. Effects of alcohol substitutions. ... 22
   4.4. Heteroaromatic systems ... 24
5. Multi-descriptor model ... 27
6. Cartesian coordinates ... 34
   6.1. Parent π-systems ... 34
   6.2. Methylated π-systems ... 39
   6.3. Secondary aryl alcohols ... 59
   6.4. Heteroaromatic systems ... 62
7. References ... 65

1. Methods

All quantum chemical calculations have been performed using the Gaussian 09 program package.[1] The B3LYP[2] hybrid functional complemented with the D3[3] dispersion correction together with the 6-31+G(d) basis set[4] was used for Geometry optimization. The nature of all stationary points was verified through the computation of the vibrational frequencies at the same level of theory. Solvation effects have been considered by using the SMD continuum solvation model (Et_2O).[5] Alternatively, the M06-2X[6] hybrid meta-GGA functional in combination with the def2-TZVPP[7] basis set was employed for geometry optimizations. Natural bond orbital (NBO) charges were computed using the NBO program package (Version 3.1).[8] Local polarizabilities were computed according to the Tkatchenko-Scheffler[9] scheme using the Multiwfn program package (Version 3.8).[10,11] Multiple linear regression analysis were performed in R (4.5.1) using the implemented "lm" function to fit linear models.[12] Buried Volumes were analyzed using ChimeraX (version 1.9)[13–15] together with the SEQCROW plugin.[16,17]

1.1. Conformer generation

Conformers were generated manually for all aryl systems, with the exception of secondary alcohols **1a** – **1r**. For these, the conformers were generated with Maestro in the OPLS_2005 force field in the absence of solvent and a 50 kJ/mol energy window.[18]

1.2. Procedure for the calculation of polarizabilities

1) The Polarizable continuum model (PCM) "Cavity surface area" (in Å^2) and the "Cavity volume" (in Å^3) are those reported for the optimized structure (default: SMD(Et_2O)/D3-B3LYP /6-31+G(d) level of theory) and can be found in the *Gaussian* output file in the format:

```
--------------------------------------------------------------------------
 Polarizable Continuum Model (PCM)
 =================================
.
.
GePol: Cavity surface area                 =    112.348 Ang**2
GePol: Cavity volume                       =     93.318 Ang**3
--------------------------------------------------------------------------
```

2) The average molecular polarizability α(iso) (in Bohr^3) can be found in the *Gaussian* output file when searching for "Isotropic polarizability" in the format:

```
Isotropic polarizability for W=    0.000000       85.18 Bohr**3.
```

With 1 Bohr = 0.529177 Å, we can convert the above value to Å^3 by multiplying with $(0.529177)^3$ = 0.14818. We thus obtain in this case: α(iso) = 85.18 Bohr^3 = 12.62 Å^3.

2. Calculation Results

2.1. Carbocyclic systems

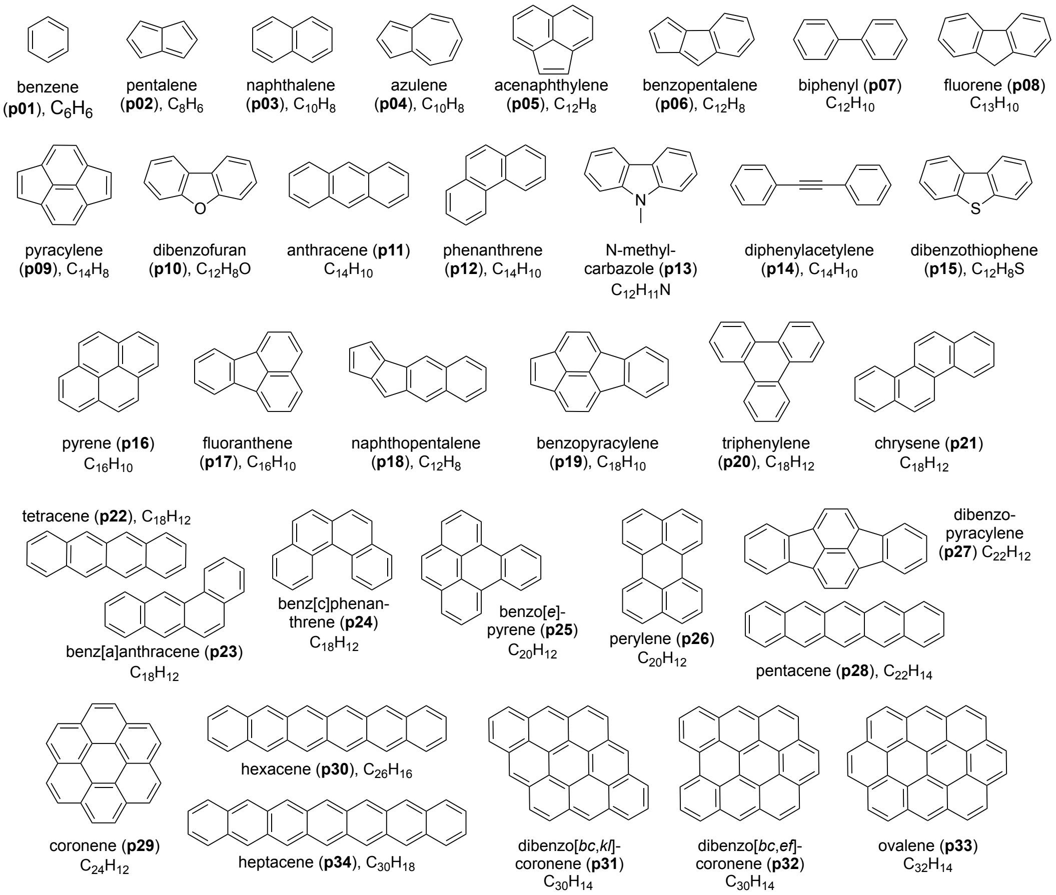

Figure S1. Structures of aryl π-systems **p1** - **p34**.

Table S1. Results for the aryl π-systems **p1** - **p34** shown in Figure S1 calculated at the SMD(Et_2O)/D3-B3LYP/6-31+G(d) level of theory (sorted by SMD cavity volume).

Molecule name	π-system	filename	E_{tot} (D3-B3LYP/ 6-31+G(d))	SMD cavity surface [10^{-20} m^2]	SMD cavity volume [10^{-30} m^3]	vol_n [d]	α(iso) [10^{-30} m^3][a] (exp)[b] [M062X][c]	α_n(iso) [d]
benzene	**p01**	pi_001.log	-232.2720356	112.378	93.289	0.24	12.62 (9.96) [9.53]	0.12
pentalene	**p02**	pi_025.log	-308.3994931	134.056	115.749	0.30	17.77	0.16
naphthalene	**p03**	pi_002.log	-385.9301965	159.227	141.981	0.37	23.03 (17.40) [16.69]	0.21
azulene	**p04**	pi_003.log	-385.8776585	160.508	143.253	0.37	26.33 (15.52) [18.36]	0.24
acenaphthylene	**p05**	pi_004.log	-462.1316825	176.087	162.299	0.42	28.22	0.26
benzopentalene	**p06**	pi_026.log	-462.0832703	181.178	164.314	0.43	29.67	0.27
biphenyl	**p07**	pi_017.log	-463.3525582	193.147	173.085	0.45	27.21 (19.64) [20.12]	0.25

fluorene	**p08**	pi_018.log	-501.4719203	195.614	178.743	0.46	29.53	0.27
							(21.68)	
							[21.62]	
pyracylene	**p09**	pi_022.log	-538.3095459	193.299	182.399	0.47	32.89	0.30
dibenzofuran	**p10**	pi_019.log	-537.3756858	195.848	185.737	0.48	27.88	0.26
anthracene	**p11**	pi_006.log	-539.5821328	205.955	190.664	0.49	35.94	0.33
							(26.70)	
							[25.34]	
phenanthrene	**p12**	pi_005.log	-539.5905929	204.040	190.816	0.50	33.79	0.31
							(24.78)	
							[24.09]	
N-methyl carbazole	**p13**	pi_021.log	-556.8368120	210.438	193.965	0.50	32.49	0.30
diphenylacetylene	**p14**	pi_028.log	-539.5123251	224.483	199.674	0.52	33.53	0.31
							(24.44)	
							[26.12]	
		pi_029.log	-539.5136657	224.404	199.620	0.52	35.87	0.33
		pi_030.log	-539.5136657	224.406	199.621	0.52	35.87	0.33
dibenzothiophene	**p15**	pi_020.log	-860.3545830	209.608	202.973	0.53	31.21	0.29
pyrene	**p16**	pi_007.log	-615.8305322	216.931	208.124	0.54	40.66	0.38
							(29.72)	
							[27.88]	
fluoranthene	**p17**	pi_016.log	-615.8077416	223.231	210.861	0.55	39.39	0.36
							(28.34)	
							[27.59]	
naphthopentalene	**p18**	pi_027.log	-615.7471093	227.172	212.760	0.55	45.20	0.42
benzopyracylene	**p19**	pi_023.log	-691.9903501	240.282	230.759	0.60	45.49	0.42
triphenylene	**p20**	pi_012.log	-693.2477646	245.147	238.435	0.62	43.68	0.40
							(32.74)	
							[31.05]	
chrysene	**p21**	pi_008.log	-693.2485519	247.603	238.835	0.62	45.76	0.42
							(34.48)	
							[32.35]	
tetracene	**p22**	pi_009.log	-693.2316072	252.622	239.329	0.62	51.17	0.47
							(34.60)	
							[35.47]	
benz[a]anthracene	**p23**	pi_010.log	-693.2451716	249.639	239.136	0.62	47.41	0.44
							(32.86)	
							[33.25]	
benz[c]phenanthrene	**p24**	pi_011.log	-693.2389443	247.467	239.646	0.62	45.25	0.42
benzo[*e*]pyrene	**p25**	pi_036.log	-769.4892507	259.200	256.404	0.67	50.39	0.47
perylene	**p26**	pi_015.log	-769.4779161	259.774	257.002	0.67	53.00	0.49
							(37.14)	
							[35.80]	
dibenzopyracylene	**p27**	pi_024.log	-845.6693842	287.371	279.136	0.72	58.81	0.55
pentacene	**p28**	pi_034.log	-846.8798720	299.261	287.978	0.75	68.46	0.63
coronene	**p29**	pi_014.log	-921.9810821	287.059	292.581	0.76	63.73	0.59
							(47.18)	
							[42.57]	
hexacene	**p30**	pi_035.log	-1000.5275995	345.888	336.621	0.87	87.49	0.81
dibenzo[bc,kl]coronene	**p31**	pi_033.log	-1151.8543996	344.691	358.691	0.93	104.02	0.96
dibenzo[*bc*,*ef*]coronene	**p32**	pi_038.log	-1151.8625484	343.603	359.544	0.93	89.11	0.83
ovalene	**p33**	pi_039.log	-1228.1185651	357.075	377.077	0.98	93.75	0.87
heptacene	**p34**	pi_037.log	-1154.1749847	392.515	385.266	1.00	107.92	1.00

[a] using SMD(Et_2O)/D3-B3LYP/6-31+G(d) geometries; [b] experimental values as described by Gussoni et al.[19]; [c] using gas phase M06-2X/def2-TZVPP geometries and energies. [d] Normalized SMD cavity volumes or isotropic polarizabilities using the values of heptacene calculated at SMD(Et_2O)/D3-B3LYP/6-31+G(d) level.

2.2. Methylated aryl systems

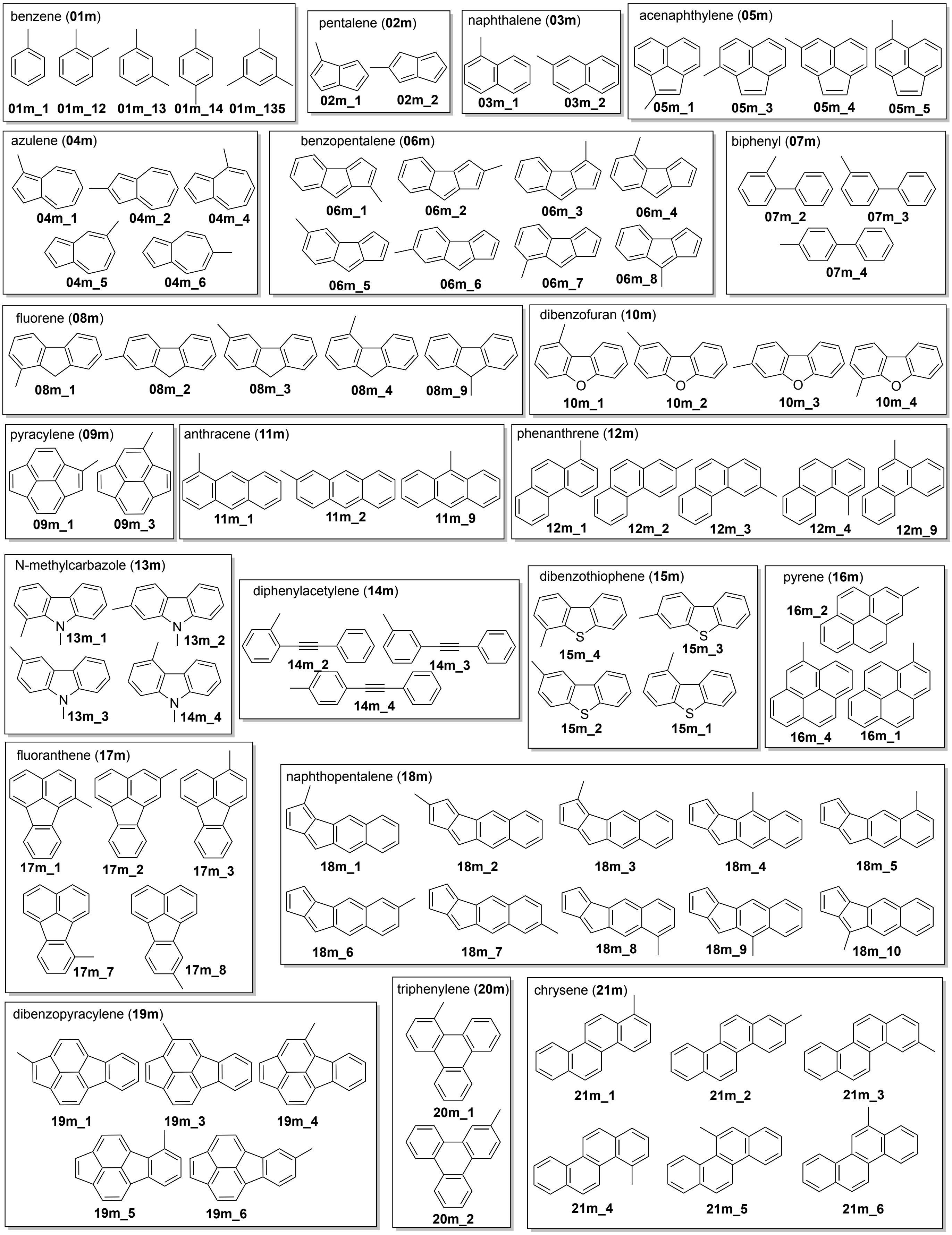

Figure S2. Structures of methylated aryl groups **01m - 21m.** Numbering follows the π-system numbering from Figure S1.

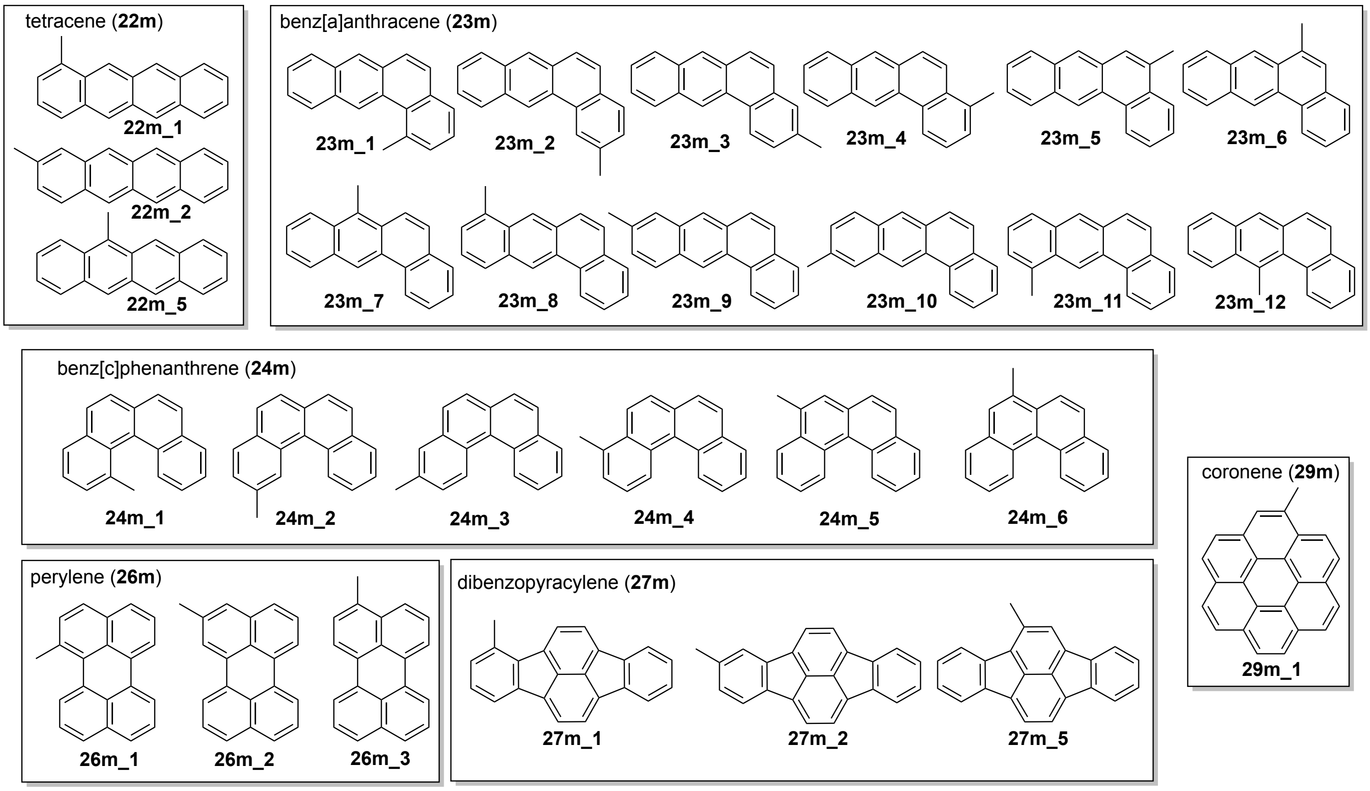

Figure S3. Structures of methylated aryl groups **22m-29m.** Numbering follows the π-system numbering from Figure S1.

Table S2. Results for the methylated aryl **m**-systems **01m** – **29m** shown in Figure S1 calculated at the SMD(Et_2O)/D3-B3LYP/6-31+G(d) level of theory (sorted by π-sytem).

π-system		π-system Methyl-pos	E_{tot} (D3-B3LYP/ 6-31+G(d))	SMD cavity surface [10^{-20} m^2]	SMD cavity volume [10^{-30} m^3]	vol_n[d]	α(iso) [10^{-30} m^3][a] (exp)[b] [M062X][c]	α_n(iso) [d]
benzene	**01m**	**01m_1**	-271.5939074	133.112	111.095	0.29	14.99<br>(11.86)<br>[11.52	0.14
		**01m_12**	-310.9157008	151.248	128.434	0.33	17.33<br>(13.65)<br>[13.38]	0.16
		**01m_14**	-310.9151154	153.758	128.801	0.33	17.39<br>(13.73)<br>[13.55]	0.16
		**01m_13**	-310.9153533	152.945	128.812	0.33	17.39<br>(13.65)<br>[13.38]	0.16
		**01m_135**	-350.2369231	173.491	146.186	0.38	19.77<br>(15.54)<br>[15.51]	0.18
pentalene	**02m**	**02m_1**	-347.726918	155.473	133.887	0.35	20.76	0.19
		**02m_2**	-347.723835	154.690	133.444	0.35	20.16	0.19
naphthalene	**03m**	**03m_1**	-425.2515904	177.286	159.145	0.41	25.43<br>(19.35)<br>[18.61]	0.24
		**03m_2**	-425.2525817	179.519	159.489	0.41	25.66<br>(19.52)<br>[18.90]	0.24
azulene	**04m**	**04m_1**	-425.1994793	180.868	161.479	0.42	28.88	0.27
		**04m_2**	-425.2014110	182.047	161.609	0.42	29.33	0.27

		**04m_4**	-425.1988378	178.547	160.522	0.42	28.67	0.27
		**04m_5**	-425.1971107	181.058	161.487	0.42	28.95	0.27
		**04m_6**	-425.1987134	181.099	161.484	0.42	29.26	0.27
acenaphthylene	**05m**	**05m_1**	-501.4562173	196.428	179.984	0.47	30.62	0.28
		**05m_3**	-501.4549743	196.082	179.859	0.47	30.93	0.29
		**05m_4**	-501.4532683	195.754	179.513	0.47	30.82	0.29
		**05m_5**	-501.4540695	195.754	179.508	0.47	30.70	0.28
benzopentalene	**06m**	**06m_1**	-501.4073274	201.500	181.922	0.47	32.13	0.30
		**06m_2**	-501.4079418	201.634	181.415	0.47	32.26	0.30
		**06m_3**	-501.4092189	202.002	182.135	0.47	32.44	0.30
		**06m_4**	-501.4067733	199.290	181.313	0.47	31.96	0.30
		**06m_5**	-501.4052982	200.982	181.709	0.47	32.52	0.30
		**06m_6**	-501.4048724	201.228	181.406	0.47	32.34	0.30
		**06m_7**	-501.4053722	201.132	182.355	0.47	32.26	0.30
		**06m_8**	-501.4112213	199.977	181.347	0.47	32.29	0.30
biphenyl	**07m**	**07m_1**	-502.6715261	211.355	190.894	0.50	28.94	0.27
		**07m_2**	-502.6743175	212.905	190.758	0.50	29.54	0.27
		**07m_3**	-502.6743819	214.125	191.015	0.50	29.85	0.28
fluorene	**08m**	**08m_1**	-540.7948423	215.673	197.252	0.51	31.82	0.29
		**08m_2**	-540.7932923	216.301	196.87	0.51	32.18	0.30
		**08m_3**	-540.7934126	217.013	197.486	0.51	31.99	0.30
		**08m_4**	-540.7932012	213.426	197.203	0.51	31.87	0.30
		**08m_5**	-540.7915989	216.416	197.642	0.51	31.79	0.29
pyracylene	**09m**	**09m_1**	-577.6344310	214.33	200.336	0.52	35.40	0.33
		**09m_3**	-577.6323360	211.58	198.871	0.52	35.66	0.33
dibenzofuran	**10m**	**10m_1**	-576.6989542	214.281	203.355	0.53	30.22	0.28
		**10m_2**	-576.6968205	217.165	204.31	0.53	30.34	0.28
		**10m_3**	-576.6972886	215.829	203.036	0.53	30.54	0.28
		**10m_4**	-576.6988256	213.709	203.051	0.53	30.24	0.28
anthracene	**11m**	**11m_1**	-578.9039308	225.019	209.077	0.54	38.26	0.35
		**11m_2**	-578.9047652	227.595	210.018	0.55	38.75	0.36
		**11m_9**	-578.8988815	221.736	208.065	0.54	38.36	0.36
phenanthrene	**12m**	**12m_1**	-578.9112535	221.063	207.502	0.54	36.31	0.34
		**12m_2**	-578.9130061	224.623	208.730	0.54	36.51	0.34
		**12m_3**	-578.9127664	223.193	208.020	0.54	36.46	0.34
		**12m_4**	-578.9019600	219.615	207.758	0.54	36.46	0.34
		**12m_9**	-578.9120274	221.276	207.570	0.54	36.31	0.34
N-methylcarbazole	**13m**	**13m_1**	-596.1541834	226.453	211.052	0.55	34.68	0.32
		**13m_2**	-596.1582626	230.447	211.370	0.55	35.12	0.33
		**13m_3**	-596.1576000	230.986	211.697	0.55	34.97	0.32
		**13m_4**	-596.1595080	227.990	211.896	0.55	34.85	0.32
diphenylacetylene	**14m**	**14m_2**	-578.8366210	244.254	218.363	0.57	38.19	0.35
		**14m_3**	-578.8356133	245.984	218.741	0.57	38.32	0.36
		**14m_4**	-578.8359952	245.149	217.205	0.56	38.79	0.36
dibenzothiophene	**15m**	**15m_1**	-899.6745164	224.871	219.899	0.57	33.62	0.31
		**15m_2**	-899.6762884	229.663	220.964	0.57	33.71	0.31
		**15m_3**	-899.6764557	229.707	220.582	0.57	33.90	0.31
		**15m_4**	-899.6784049	224.398	218.682	0.57	33.65	0.31
pyrene	**16m**	**16m_1**	-655.1515064	235.863	226.358	0.59	43.44	0.40
		**16m_2**	-655.1525385	237.618	225.762	0.59	43.41	0.40
		**16m_4**	-655.1524991	236.207	227.206	0.59	43.14	0.40
fluoranthene	**17m**	**17m_1**	-655.1307718	240.846	228.535	0.59	41.93	0.39
		**17m_2**	-655.1295588	243.623	228.94	0.59	41.99	0.39
		**17m_3**	-655.1301932	241.428	227.745	0.59	42.07	0.39
		**17m_7**	-655.1304748	239.658	227.645	0.59	41.78	0.39
		**17m_8**	-655.1292975	243.466	228.561	0.59	42.07	0.39
naphthopentalene	**18m**	**18m_1**	-655.0707096	248.038	230.613	0.60	47.65	0.44
		**18m_2**	-655.0718264	246.853	229.651	0.60	47.91	0.44
		**18m_3**	-655.0723327	247.583	230.200	0.60	47.69	0.44
		**18m_4**	-655.0688510	242.789	229.914	0.60	47.48	0.44
		**18m_5**	-655.0682830	245.634	230.423	0.60	47.55	0.44
		**18m_6**	-655.0694337	247.456	230.713	0.60	48.24	0.45
		**18m_7**	-655.0691821	247.055	229.656	0.60	48.07	0.45
		**18m_8**	-655.0681866	245.152	229.586	0.60	47.80	0.44
		**18m_9**	-655.0682372	242.903	229.208	0.59	47.81	0.44

		**18m_10**	-655.0749100	247.176	230.418	0.60	47.36	0.44
benzopyracylene	**19m**	**19m_1**	-731.3150250	261.226	249.548	0.65	48.00	0.44
		**19m_3**	-731.3127339	259.301	247.807	0.64	48.32	0.45
		**19m_4**	-731.3128990	258.224	248.140	0.64	48.06	0.45
		**19m_5**	-731.3134845	257.843	247.531	0.64	47.97	0.44
		**19m_6**	-731.3122735	261.033	249.589	0.65	48.31	0.45
triphenylene	**20m**	**20m_1**	-732.5579640	262.267	256.437	0.67	46.35	0.43
		**20m_2**	-732.5700552	264.219	255.848	0.66	46.41	0.43
chrysene	**21m**	**21m_1**	-732.5695707	266.501	257.37	0.67	48.18	0.45
		**21m_2**	-732.5708375	267.465	256.692	0.67	48.59	0.45
		**21m_3**	-732.5707076	267.245	256.988	0.67	48.47	0.45
		**21m_4**	-732.5590488	265.137	257.098	0.67	48.51	0.45
		**21m_5**	-732.5586070	264.413	256.062	0.66	48.58	0.45
		**21m_6**	-732.5700543	266.721	257.675	0.67	48.26	0.45
tetracene	**22m**	**22m_1**	-732.5536069	272.403	258.943	0.67	53.45	0.50
		**22m_2**	-732.5545303	273.828	258.319	0.67	54.18	0.50
		**22m_5**	-732.5488229	268.615	257.322	0.67	53.54	0.50
benz[a]anthracene	**23m**	**23m_1**	-732.5569989	267.426	257.101	0.67	50.10	0.46
		**23m_2**	-732.5667555	271.425	258.044	0.67	49.98	0.46
		**23m_3**	-732.5672800	269.346	256.339	0.67	50.24	0.47
		**23m_4**	-732.5656100	268.116	256.952	0.67	49.98	0.46
		**23m_5**	-732.5666374	268.441	256.712	0.67	50.06	0.46
		**23m_6**	-732.5667941	267.993	256.494	0.67	49.71	0.46
		**23m_7**	-732.5608440	264.342	255.461	0.66	49.89	0.46
		**23m_8**	-732.5668987	268.553	257.135	0.67	49.80	0.46
		**23m_9**	-732.5675817	269.449	256.176	0.66	50.30	0.47
		**23m_10**	-732.5677053	270.77	256.932	0.67	50.24	0.47
		**23m_11**	-732.5668466	268.695	256.764	0.67	49.60	0.46
		**23m_12**	-732.5526673	265.145	256.746	0.67	50.17	0.46
benz[c]phenanthrene	**24m**	**24m_1**	-732.5505656	263.425	256.169	0.66	47.69	0.44
		**24m_2**	-732.5612800	267.666	256.962	0.67	47.82	0.44
		**24m_3**	-732.5614488	268.104	257.341	0.67	47.99	0.44
		**24m_4**	-732.5595366	265.568	256.98	0.67	47.73	0.44
		**24m_5**	-732.5604523	266.38	257.067	0.67	47.88	0.44
		**24m_6**	-732.5597662	266.39	256.955	0.67	47.77	0.44
perylene	**26m**	**26m_1**	-808.7906443	278.099	275.669	0.72	55.23	0.51
		**26m_2**	-808.8003352	280.465	275.72	0.72	55.54	0.51
		**26m_3**	-808.7994161	277.785	275.389	0.71	56.08	0.52
dibenzopyracylene	**27m**	**27m_1**	-884.9925630	304.404	295.678	0.77	61.29	0.57
		**27m_2**	-884.9912460	308.176	298.09	0.77	61.65	0.57
		**27m_5**	-884.9921100	304.549	295.903	0.77	61.48	0.57
coronene	**29m**	**29m_1**	-961.3024736	304.742	310.52	0.81	66.49	0.62

[a] using SMD(Et_2O)/D3-B3LYP/6-31+G(d) geometries; [b] experimental values as described by Gussoni et al.[19]; [c] using gas phase M06-2X/def2-TZVPP geometries and energies. [d] Normalized SMD cavity volumes or isotropic polarizabilities using the values of heptacene calculated at SMD(Et_2O)/D3-B3LYP/6-31+G(d) level.

2.3. Secondary aryl alcohols

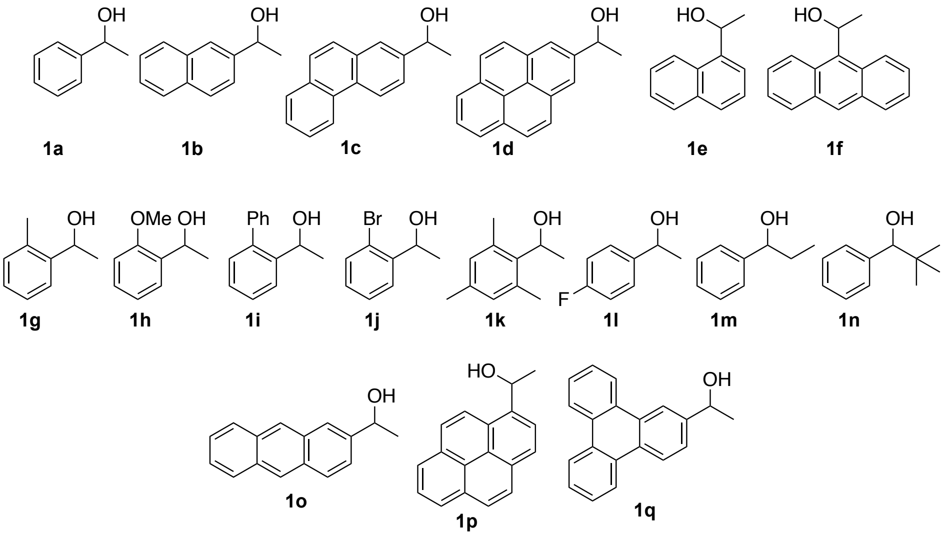

Figure S4. Structures of alcohols **1a** - **1q.**

Table S3. Results for alcohols **1a** - **1q** shown in Figure S4 calculated at the SMD(Et_2O)/D3-B3LYP/6-31+G(d) level of theory (sorted by E_{tot}).

Name (filename)	E_{tot} (D3-B3LYP/ 6-31+G(d))	SMD cavity surface [10^{-20} m^2]	SMD cavity volume [10^{-30} m^3]	vol_n[b]	α(iso) [10^{-30} m^3][a]	α_n(iso) [b]
**1-Phenylethanol (1a)**						
PhEtOH_001_opt_fr	-386.1337529	173.944	156.852	0.41	17.95	0.17
PhEtOH_006_opt_fr	-386.1320049	172.693	156.071	0.41	17.95	0.17
PhEtOH_002_opt_fr	-386.1318910	175.097	157.781	0.41	17.95	0.17
PhEtOH_003_opt_fr	-386.1318909	175.097	157.780	0.41	17.95	0.17
PhEtOH_005_opt_fr	-386.1316996	173.444	156.882	0.41	17.87	0.17
**1-(2-Naphthyl)ethanol (1b)**						
2npEtOH_003_opt_fr	-539.7924151	220.066	205.204	0.53	28.68	0.26
2npEtOH_001_opt_fr	-539.7919839	220.283	205.360	0.53	28.57	0.27
2npEtOH_011_opt_fr	-539.7906018	220.094	205.790	0.53	28.53	0.27
2npEtOH_004b_opt_fr	-539.7905452	219.967	205.643	0.53	28.57	0.27
2npEtOH_009_opt_fr	-539.7905451	219.967	205.643	0.53	28.57	0.27
2npEtOH_002_opt_fr	-539.7905305	221.311	206.038	0.53	28.65	0.27
2npEtOH_005_opt_fr	-539.7905305	221.311	206.037	0.53	28.64	0.26
2npEtOH_010c_opt_fr	-539.7905305	221.310	206.037	0.53	28.64	0.27
2npEtOH_008b_opt_fr	-539.7905295	220.500	205.385	0.53	28.61	0.27
2npEtOH_006_opt_fr	-539.7905036	219.547	205.022	0.53	28.60	0.26
2npEtOH_007_opt_fr	-539.7889639	219.495	206.110	0.53	28.64	0.26
**1-(2-Phenanthryl)ethanol (1c)**						
2PhAnEtOH_003_opt_fr	-693.4527668	265.123	254.663	0.66	39.57	0.37
2PhAnEtOH_001_opt_fr	-693.4524231	263.935	252.908	0.66	39.47	0.37
2PhAnEtOH_008_opt_fr	-693.4509377	265.404	254.569	0.66	39.51	0.37
2PhAnEtOH_007_opt_fr	-693.4508947	263.429	253.166	0.66	39.47	0.37
2PhAnEtOH_004c_opt_fr	-693.4508947	263.420	253.160	0.66	39.43	0.37
2PhAnEtOH_011_opt_fr	-693.4508946	263.430	253.168	0.66	39.42	0.37
2PhAnEtOH_002_opt_fr	-693.4508942	266.140	255.003	0.66	39.56	0.37
2PhAnEtOH_005_opt_fr	-693.4508942	266.140	255.003	0.66	39.56	0.37
2PhAnEtOH_006_opt_fr	-693.4508464	263.159	252.614	0.66	39.49	0.37
2PhAnEtOH_009_opt_fr	-693.4508189	263.712	253.937	0.66	39.51	0.37
2PhAnEtOH_010_opt_fr	-693.4500970	264.838	254.220	0.66	39.39	0.37
**1-(2-Pyrenyl)ethanol (1d)**						
2pyrEtOH_001_opt_fr	-769.6924436	279.238	273.121	0.43	46.41	0.71

2pyrEtOH_004_opt_fr	-769.6906289	279.455	272.472	0.43	46.33	0.71
2pyrEtOH_003_opt_fr	-769.6904902	280.123	273.510	0.43	46.39	0.71
2pyrEtOH_002_opt_fr	-769.6904902	280.123	273.510	0.43	46.37	0.71
2pyrEtOH_006_opt_fr	-769.6903280	277.650	271.598	0.43	46.29	0.70
2pyrEtOH_005_opt_fr	-769.6901317	277.629	271.873	0.43	46.36	0.71
**1-(1-Naphthyl)ethanol (1e)**						
1npEtOH_001_opt_fr	-539.7892681	216.874	204.985	0.53	28.20	0.26
1npEtOH_004_opt_fr	-539.7883728	213.555	203.443	0.53	28.24	0.26
1npEtOH_005_opt_fr	-539.7879155	214.605	204.343	0.53	28.25	0.26
1npEtOH_002_opt_fr	-539.7876963	216.470	204.777	0.53	28.23	0.26
1npEtOH_003_opt_fr	-539.7876303	216.856	205.398	0.53	28.19	0.26
1npEtOH_006_opt_fr	-539.7871876	215.826	205.286	0.53	28.23	0.26
1npEtOH_008_opt_fr	-539.7862531	214.076	203.989	0.53	28.29	0.26
1npEtOH_007_opt_fr	-539.7862225	213.872	203.515	0.53	28.24	0.26
**1-(9-Anthracenyl)ethanol (1f)**						
9AnEtOH_001_opt_fr	-693.4337576	257.544	251.980	0.65	41.09	0.38
9AnEtOH_002_opt_fr	-693.4337575	257.545	251.982	0.65	41.09	0.38
9AnEtOH_003_opt_fr	-693.4315040	257.721	252.183	0.65	41.13	0.38
**PhEtOH_2Me (1g)**						
PhEtOH_2Me_001_opt_fr	-425.4538926	191.934	173.800	0.45	20.20	0.19
PhEtOH_2Me_004_opt_fr	-425.4522653	188.644	173.351	0.45	20.30	0.19
PhEtOH_2Me_005_opt_fr	-425.4522420	191.147	173.457	0.45	20.23	0.19
PhEtOH_2Me_008_opt_fr	-425.4515343	192.256	174.479	0.45	20.24	0.19
PhEtOH_2Me_003_opt_fr	-425.4515342	192.257	174.481	0.45	20.24	0.19
PhEtOH_2Me_002_opt_fr	-425.4512083	187.216	172.547	0.45	20.28	0.19
PhEtOH_2Me_006_opt_fr	-425.4499886	189.143	173.692	0.45	20.26	0.19
PhEtOH_2Me_007_opt_fr	-425.4496482	187.038	172.378	0.45	20.31	0.19
**PhEtOH_2OMe (1h)**						
PhEtOH_2OMe_004_opt_fr	-500.6668106	199.855	191.681	0.50	21.03	0.19
PhEtOH_2OMe_001_opt_fr	-500.6653941	202.203	194.531	0.50	21.03	0.19
PhEtOH_2OMe_005_opt_fr	-500.6640510	201.577	193.924	0.50	21.04	0.19
PhEtOH_2OMe_003_opt_fr	-500.6640484	200.894	193.472	0.50	21.05	0.20
PhEtOH_2OMe_002b_opt_fr	-500.6624807	206.870	196.691	0.51	21.08	0.20
PhEtOH_2OMe_006_opt_fr	-500.6596169	199.226	191.570	0.50	21.21	0.20
**PhEtOH_2Ph (1i)**						
PhEtOH_2Ph_001b_opt_fr	-617.2105658	249.99	235.76	0.61	31.76	0.29
PhEtOH_2Ph_002_opt_fr	-617.2091501	244.904	234.794	0.61	31.88	0.30
PhEtOH_2Ph_005_opt_fr	-617.2091073	246.372	234.834	0.61	31.35	0.29
PhEtOH_2Ph_003_opt_fr	-617.2087333	250.357	235.58	0.61	31.38	0.29
PhEtOH_2Ph_004_opt_fr	-617.2084700	249.615	235.801	0.61	31.38	0.29
PhEtOH_2Ph_006_opt_fr	-617.2080784	245.208	234.268	0.61	31.11	0.29
PhEtOH_2Ph_007_opt_fr	-617.2064618	243.549	233.767	0.61	31.59	0.29
PhEtOH_2Ph_008_opt_fr	-617.2052005	243.394	233.525	0.61	31.55	0.29
**PhEtOH_2Br (1j)**						
PhEtOH_2Br_001_opt_fr	-2957.2673766	224.213	240.836	0.63	21.19	0.20
PhEtOH_2Br_002_opt_fr	-2957.2659213	223.645	240.328	0.62	21.20	0.20
PhEtOH_2Br_003_opt_fr	-2957.2655901	223.758	240.388	0.62	21.19	0.20
PhEtOH_2Br_004_opt_fr	-2957.2650827	218.888	235.538	0.61	21.28	0.20
PhEtOH_2Br_006_opt_fr	-2957.2650827	218.890	235.539	0.61	21.28	0.20
PhEtOH_2Br_007_opt_fr	-2957.2640382	230.071	243.978	0.61	21.29	0.20
PhEtOH_2Br_005_opt_fr	-2957.2628475	217.961	234.684	0.63	21.40	0.20
**PhEtOH_2-4-6Me (1k)**						
PhEtOH_2-4-6Me_002_opt_fr	-504.0911574	224.806	207.303	0.54	24.95	0.23
PhEtOH_2-4-6Me_003_opt_fr	-504.0900991	226.339	208.137	0.54	24.94	0.23
PhEtOH_2-4-6Me_004_opt_fr	-504.0895381	224.663	207.127	0.54	24.99	0.23
PhEtOH_2-4-6Me_001_opt_fr	-504.0878386	226.492	209.053	0.54	24.95	0.23
**PhEtOH_4F (1l)**						
PhEtOH_4F_001_opt_fr	-485.3764228	184.372	166.474	0.43	17.76	0.16
PhEtOH_4F_002_opt_fr	-485.3748649	184.217	166.047	0.43	17.75	0.16
PhEtOH_4F_003_opt_fr	-485.3745720	185.423	167.311	0.43	17.76	0.16
PhEtOH_4F_005_opt_fr	-485.3743310	183.777	166.594	0.43	17.75	0.16
PhEtOH_4F_004_opt_fr	-485.3743224	183.658	166.114	0.43	17.65	0.16

**PhPrOH (1m)**						
PhPrOH_003_opt_fr	-425.4523462	192.995	174.415	0.45	20.02	0.19
PhPrOH_001_opt_fr	-425.4519484	190.897	173.522	0.45	20.16	0.19
PhPrOH_004_opt_fr	-425.4510880	189.142	173.176	0.45	19.97	0.19
PhPrOH_011_opt_fr	-425.4506577	192.869	174.334	0.45	20.03	0.19
PhPrOH_009_opt_fr	-425.4505294	194.537	175.235	0.45	20.00	0.19
PhPrOH_005_opt_fr	-425.4504106	190.935	173.222	0.45	20.12	0.19
PhPrOH_006_opt_fr	-425.4500982	192.399	174.394	0.45	20.11	0.19
PhPrOH_008_opt_fr	-425.4500981	192.403	174.396	0.45	20.11	0.18
PhPrOH_002_opt_fr	-425.4500980	192.406	174.398	0.45	20.11	0.19
PhPrOH_010_opt_fr	-425.4498257	188.996	173.114	0.45	19.95	0.18
PhPrOH_007_opt_fr	-425.4495097	190.558	173.984	0.45	19.90	0.19
**PhtBuOH (1n)**						
PhtBuOH_001_opt_fr	-504.0891599	220.049	206.304	0.54	24.55	0.23
PhtBuOH_005_opt_fr	-504.0891598	220.049	206.304	0.54	24.55	0.23
PhtBuOH_004_opt_fr	-504.0877851	220.818	206.827	0.54	24.50	0.23
PhtBuOH_003_opt_fr	-504.0870336	221.82	207.551	0.54	24.47	0.23
PhtBuOH_002_opt_fr	-504.0870335	221.816	207.55	0.54	24.48	0.23
**1-(2-Anthracenyl)ethanol (1o)**						
2AnEtOH_003_opt_fr	-693.4446297	268.442	255.662	0.66	41.84	0.39
2AnEtOH_001_opt_fr	-693.4441340	265.919	253.42	0.66	41.72	0.39
2AnEtOH_004_opt_fr	-693.4428478	265.282	253.606	0.66	41.72	0.39
2AnEtOH_009_opt_fr	-693.4428478	265.283	253.606	0.66	41.72	0.39
2AnEtOH_011_opt_fr	-693.4427646	265.262	253.345	0.66	41.66	0.39
2AnEtOH_007_opt_fr	-693.4427646	265.263	253.346	0.66	41.66	0.39
2AnEtOH_006_opt_fr	-693.4426777	265.107	253.058	0.66	41.78	0.39
2AnEtOH_008_opt_fr	-693.4426530	268.296	255.468	0.66	41.71	0.39
2AnEtOH_012_opt_fr	-693.4426530	268.297	255.469	0.66	41.71	0.39
2AnEtOH_005c_opt_fr	-693.4426294	269.180	256.130	0.66	41.79	0.39
2AnEtOH_010_opt_fr	-693.4426294	269.189	256.132	0.66	41.80	0.39
2AnEtOH_002_opt_fr	-693.4426294	269.189	256.133	0.66	41.79	0.39
**1-(1-Pyrenyl)ethanol (1p)**						
1pyrEtOH_001_opt_fr	-769.6893477	274.462	271.369	0.43	46.31	0.70
1pyrEtOH_004_opt_fr	-769.6887279	271.898	270.020	0.43	46.32	0.70
1pyrEtOH_006_opt_fr	-769.6877809	271.803	270.925	0.43	46.43	0.70
1pyrEtOH_003_opt_fr	-769.6877690	273.697	271.100	0.43	46.28	0.70
1pyrEtOH_005_opt_fr	-769.6875227	274.477	272.038	0.43	46.29	0.71
1pyrEtOH_007_opt_fr	-769.6871813	273.720	272.470	0.43	46.33	0.71
1pyrEtOH_008_opt_fr	-769.6865672	272.159	270.093	0.43	46.28	0.70
1pyrEtOH_002_opt_fr	-769.6862004	276.732	272.737	0.43	46.47	0.71
1pyrEtOH_009_opt_fr	-769.6861729	271.737	270.921	0.43	46.41	0.70
**1-(2-Triphenylenyl)ethanol (1r)**						
2tPhnEtOH_003_opt_fr	-847.1100019	304.657	301.34	0.78	49.42	0.46
2tPhnEtOH_002_opt_fr	-847.1099824	304.708	301.069	0.78	49.33	0.46
2tPhnEtOH_004b_opt_fr	-847.1083459	303.99	300.46	0.78	49.36	0.46
2tPhnEtOH_006_opt_fr	-847.1082194	304.071	301.061	0.78	49.28	0.46
2tPhnEtOH_011_opt_fr	-847.1082194	304.072	301.061	0.78	49.28	0.46
2tPhnEtOH_008_opt_fr	-847.1081405	305.037	301.288	0.78	49.35	0.46
2tPhnEtOH_007_opt_fr	-847.1081196	306.765	302.543	0.79	49.41	0.46
2tPhnEtOH_010_opt_fr	-847.1081196	306.764	302.543	0.79	49.41	0.46
2tPhnEtOH_012_opt_fr	-847.1080342	303.916	300.979	0.78	49.36	0.46
2tPhnEtOH_005_opt_fr	-847.1077789	306.392	302.721	0.79	49.31	0.46
2tPhnEtOH_001_opt_fr	-847.1077475	307.235	303.429	0.79	49.27	0.46
2tPhnEtOH_009_opt_fr	-847.1075503	305.488	302.337	0.78	49.19	0.46
2tPhnEtOH_013_opt_fr	-847.1063723	303.719	301.184	0.78	49.37	0.46

[a] using SMD(Et_2O)/D3-B3LYP/6-31+G(d) geometries; [b] normalized SMD cavity volumes or isotropic polarizabilities using the values of heptacene calculated at SMD(Et_2O)/D3-B3LYP/6-31+G(d) level.

2.4. Heteroaromatic systems

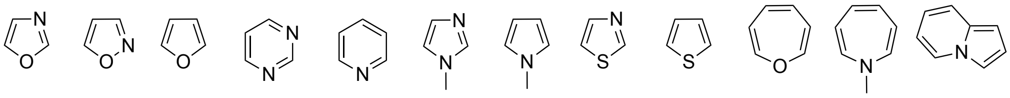

h01 oxazole, **h02** isoxazole, **h03** furan, **h04** pyrimidine, **h05** pyridine, **h06** 1-methyl imidazole, **h07** 1-methyl pyrrole, **h08** thiazole, **h09** thiophene, **h10** oxepine, **h11** 1-methyl azepine, **h12** indolizine

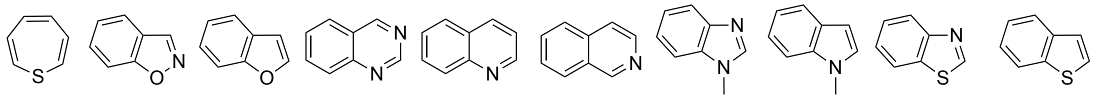

h13 thiepine, **h14** benzo-isoxazole, **h15** benzofuran, **h16** quinazoline, **h17** isoquinoline, **h18** quinoline, **h19** 1-methyl benzimidazole, **h20** 1-methyl indole, **h21** benzo-thiazole, **h22** benzo-thiophene

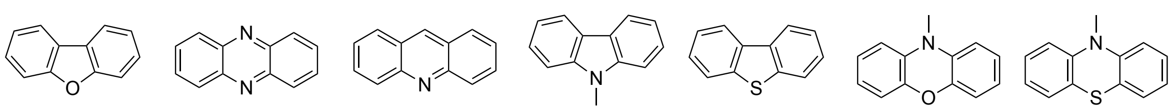

h23 / p10 dibenzofuran, **h24** phenazine, **h25** acridine, **h26 / p13** N-methyl carbazole, **h27 / p15** dibenzo-thiophene, **h28** 10-methyl phenoxazine, **h29** 10-methyl phenothiazine

Figure S5. Structures of selected heteroaromatic systems.

Table S4. Results for the heteroaromatic systems shown Figure S5 calculated at the SMD(Et_2O)/D3-B3LYP/6-31+G(d) level of theory.

Name (filename if different)	Atom	Number	E_{tot} (D3-B3LYP/ 6-31+G(d))	SMD cavity surface [10^{-20} m^2]	SMD cavity volume [10^{-30} m^3]	α(iso) [10^{-30} m^3][a]	α_n(iso)[b]
oxazole	O+N	**h01**	-246.0901235	103.096	88.425	7.31	0.07
isoxazole	O+N	**h02**	-246.0555118	103.173	88.550	7.44	0.07
furan	O	**h03**	-230.0402659	104.596	89.497	8.35	0.08
pyrimidine	N	**h04**	-264.3509873	108.754	90.940	10.36	0.10
pyridine	N	**h05**	-248.3095512	110.619	92.184	11.49	0.11
1-methylimidazole (imidazole)	N	**h06**	-265.5604984	116.684	95.473	10.72	0.10
1-methylpyrrole (pyrrole)	N	**h07**	-249.5057030	118.836	97.002	12.00	0.11
thiazole	S+N	**h08**	-569.0658853	116.638	105.131	10.20	0.09
thiophene	S	**h09**	-553.0213059	118.62	106.455	11.25	0.10
oxepine	O	**h10**	-307.4272284	131.825	117.816	13.78	0.13
1-methylazepine (azepine)	N	**h11**	-326.8833675	146.032	125.700	17.83	0.17
indolizine	N	**h12**	-363.8349571	145.632	127.981	19.90	0.18
thiepine	S	**h13**	-630.4055515	145.194	134.939	16.83	0.16
benzoisoxazole (Bzisoxazole)	O+N	**h14**	-399.7562524	149.376	136.904	16.23	0.15
benzofuran (Bzfuran)	O	**h15**	-383.7065154	150.883	137.853	17.57	0.16
quinazoline	N	**h16**	-418.0113373	155.086	139.431	20.40	0.19
quinoline	N	**h17**	-401.9691926	157.132	140.824	21.80	0.20
isoquinoline	N	**h18**	-401.9677154	157.529	141.008	21.64	0.20
1-methylbenzimidazole (Bzimidazole)	N	**h19**	-419.2253738	163.339	144.372	20.15	0.19
1-methylindole (indole)	N	**h20**	-403.1701569	165.747	146.072	21.70	0.20
benzothiazole (Bzthiazole)	S+N	**h21**	-722.7322916	162.396	153.706	19.69	0.18
benzothiophene (Bzthiophene)	S	**h22**	-706.6866049	164.166	154.683	20.95	0.19
dibenzofuran	O	**h23 / p10**	-537.3756858	195.848	185.737	27.9	0.26
phenazine	N	**h24**	-571.6578559	201.015	187.866	33.78	0.31
acridine	N	**h25**	-555.6229105	203.527	189.548	34.56	0.32
N-methylcarbazole	N	**h26 / p13**	-556.8368120	210.438	193.965	32.5	0.30
dibenzothiophene	S	**h27 / p15**	-860.3545830	209.608	202.973	31.2	0.29
10-methylphenoxazine (phenoxazine)	O+N	**h28**	-632.0337391	221.398	214.859	32.02	0.30
10-methylphenothiazine (phenothiazine)	S+N	**h29**	-955.0076791	234.377	231.817	35.03	0.32

[a] using SMD(Et_2O)/D3-B3LYP/6-31+G(d) geometries; [b] normalized SMD cavity volumes or isotropic polarizabilities using the values of heptacene calculated at SMD(Et_2O)/D3-B3LYP/6-31+G(d) level

3. Volume and polarizability scales

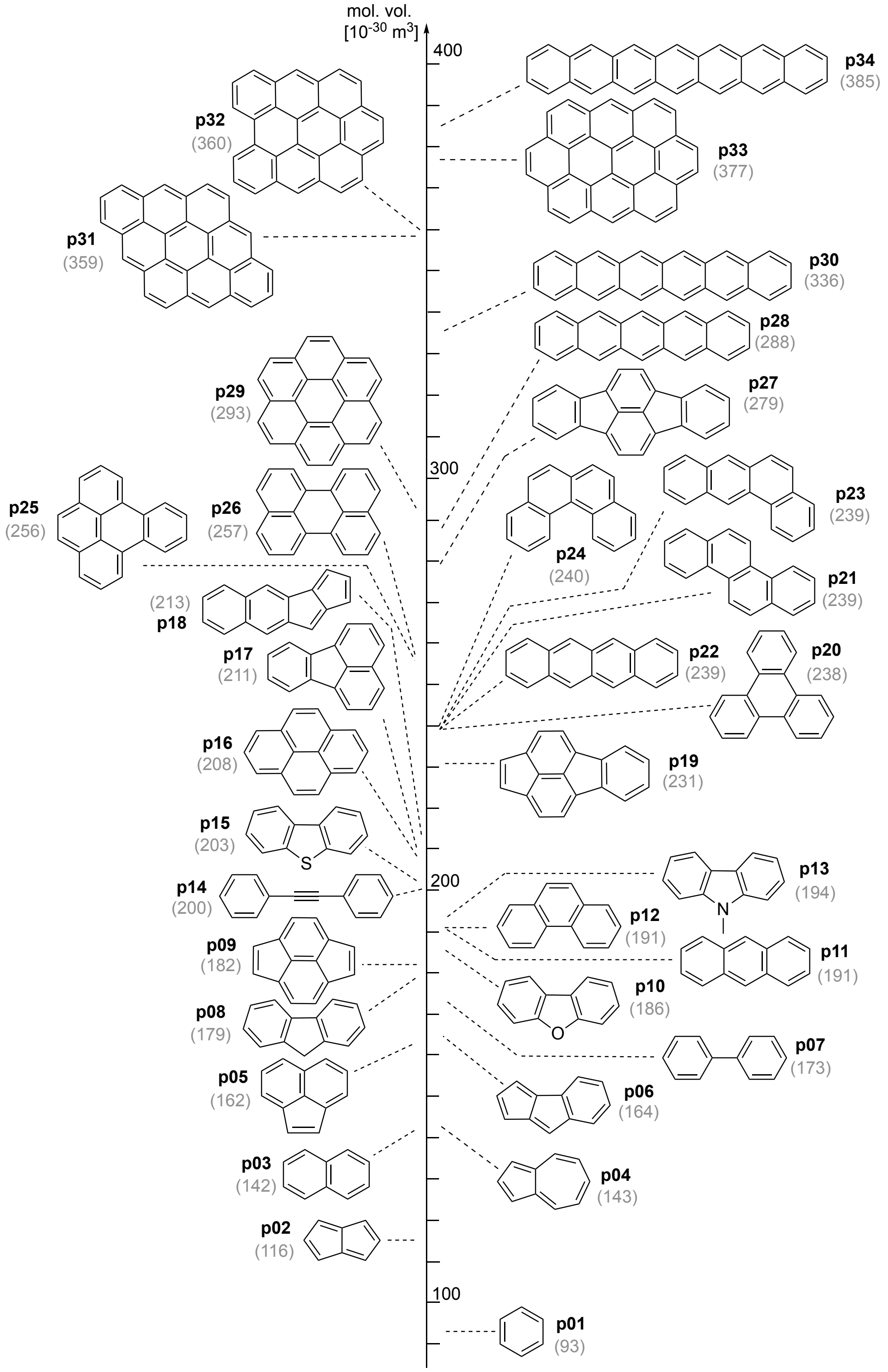

Figure S6. Molecular volume parameters (in units of 10^{-30} m^3) for selected carbo- and heterocyclic π-systems **p01 - p34** calculated at the SMD(Et_2O)/D3-B3LYP/6-31+G(d) level of theory.

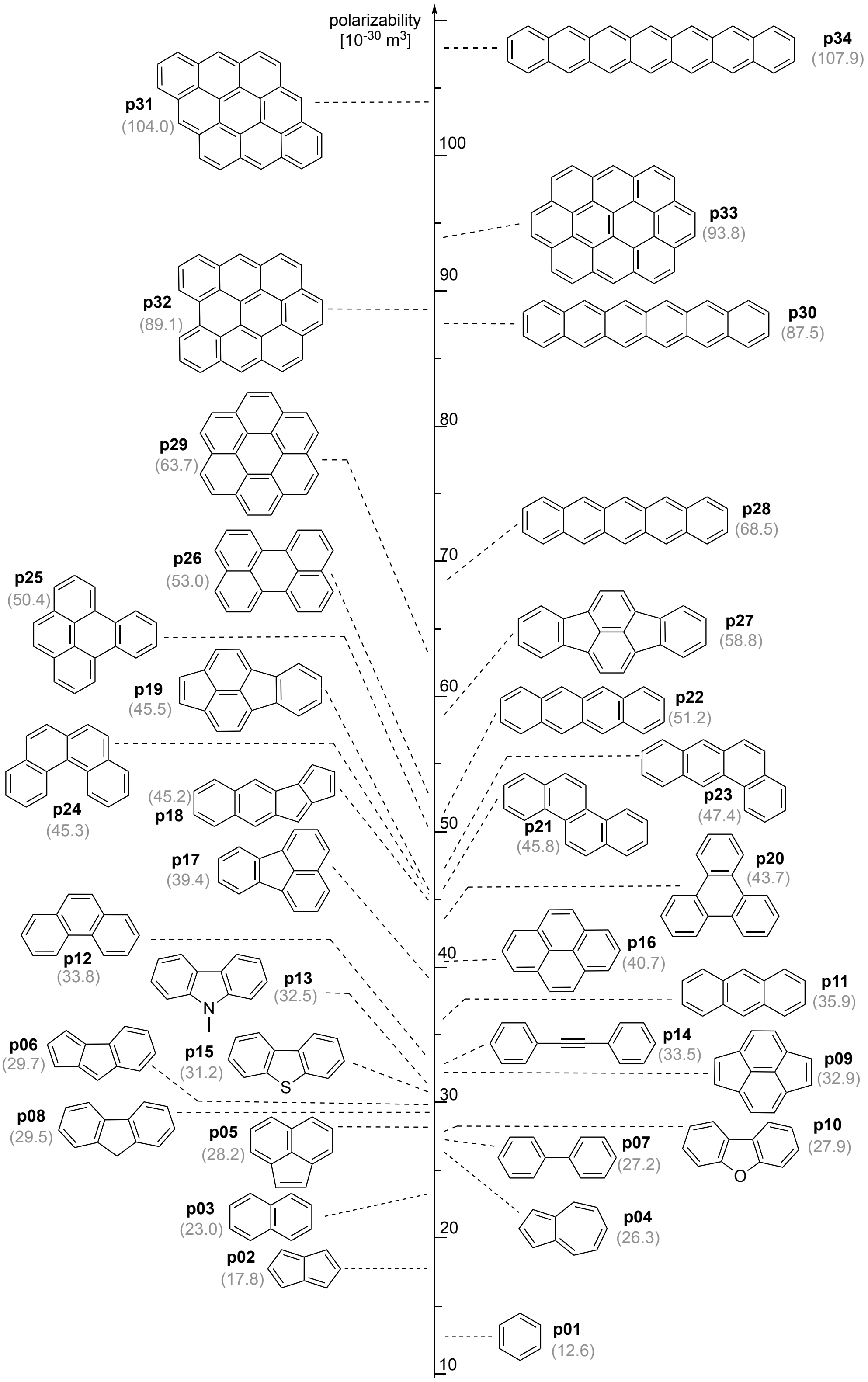

Figure S7. Isotropic polarizability α (in units of 10^{-30} m^3) for selected carbo- and heterocyclic π-systems **p01 - p34** calculated at the SMD(Et_2O)/D3-B3LYP/6-31+G(d) level of theory.

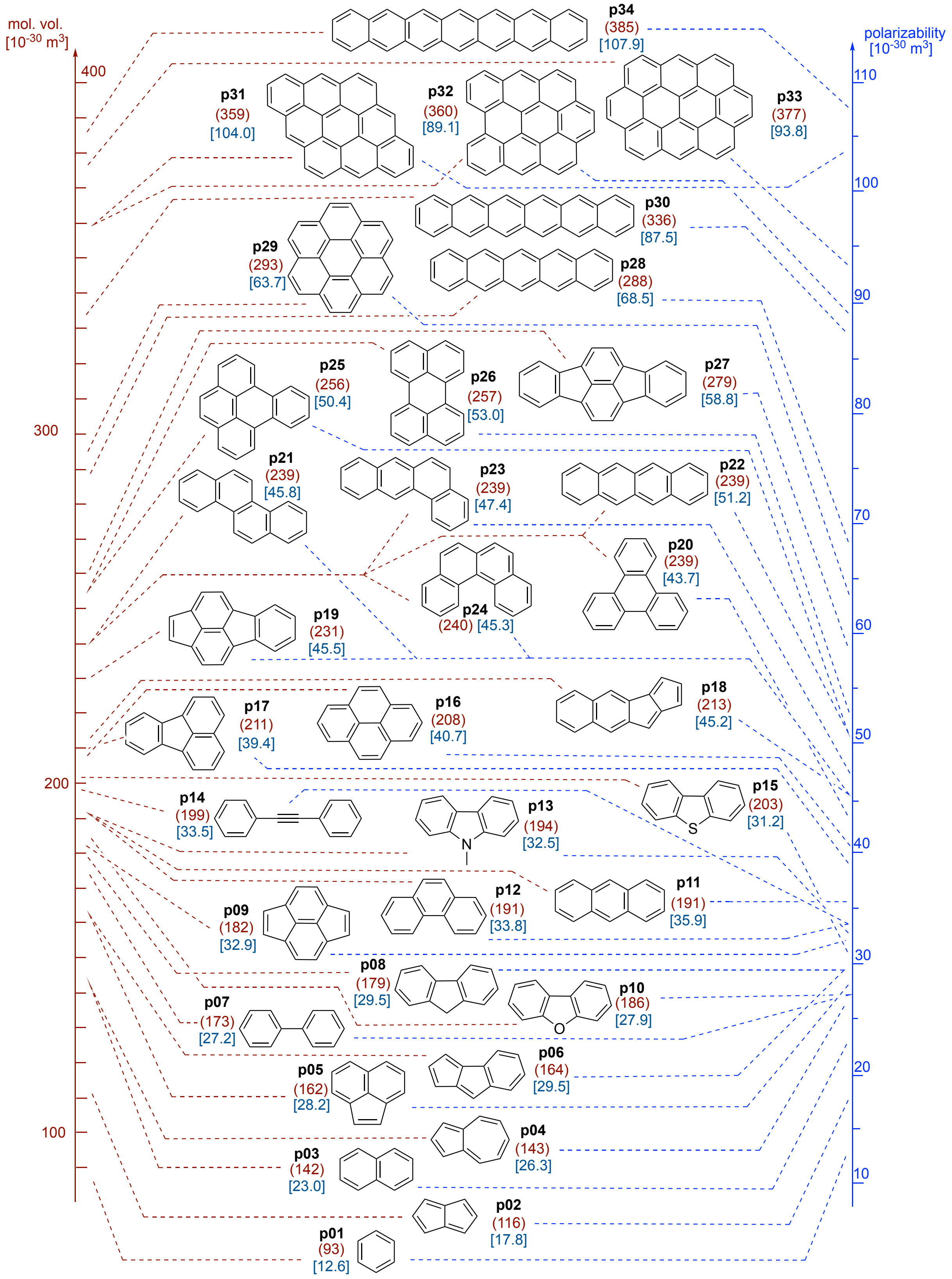

Figure S8. Molecular volume and isotropic polarizability α parameters (in units of 10^{-30} m^3) for selected carbo- and heterocyclic π-systems **p01 - p34** calculated at the SMD(Et_2O)/D3-B3LYP/6-31+G(d) level of theory.

4. Correlation analysis

4.1. Carbocyclic systems

For 23 of the systems shown in Table S1 and Table S2, the isotropic gas phase molecular polarizability α(iso) is known experimentally (Gussoni et al.[19]). Anecdotal evidence suggests that these can be reproduced with good accuracy through gas phase calculations at the M06-2X/def2-TZVPP level of theory. From the correlation shown in Figure S9 we find this to be true with R^2 = 0.9825, a slope of 1.070 and an intercept of + 1.106.

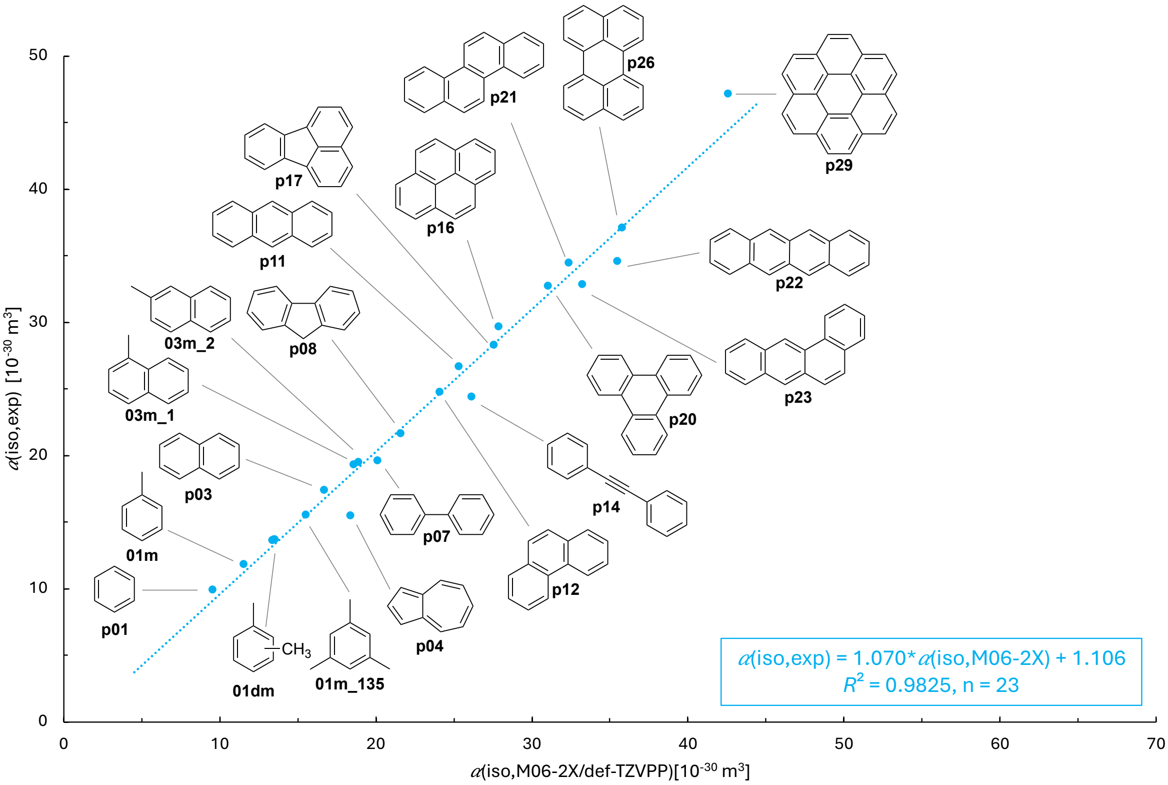

Figure S9. Correlation between experimental gas phase isotropic polarizability α(iso,exp) and gas phase isotropic polarizability α(iso,M06-2X) calculated at the M06-2X/def2-TZVPP level of theory.

Comparing the experimental gas phase polarizabilities with those obtained from solution phase SMD(Et_2O)/D3-B3LYP/6-31+G(d) calculations used in earlier studies gives a slightly better correlation with R^2 = 0.9834, but with rather different slope (0.693) and intercept (+1.155) parameters (Figure S10). The slope of 0.693 indicates that the calculate polarizabilities are much larger as compared to the experimental gas phase values.

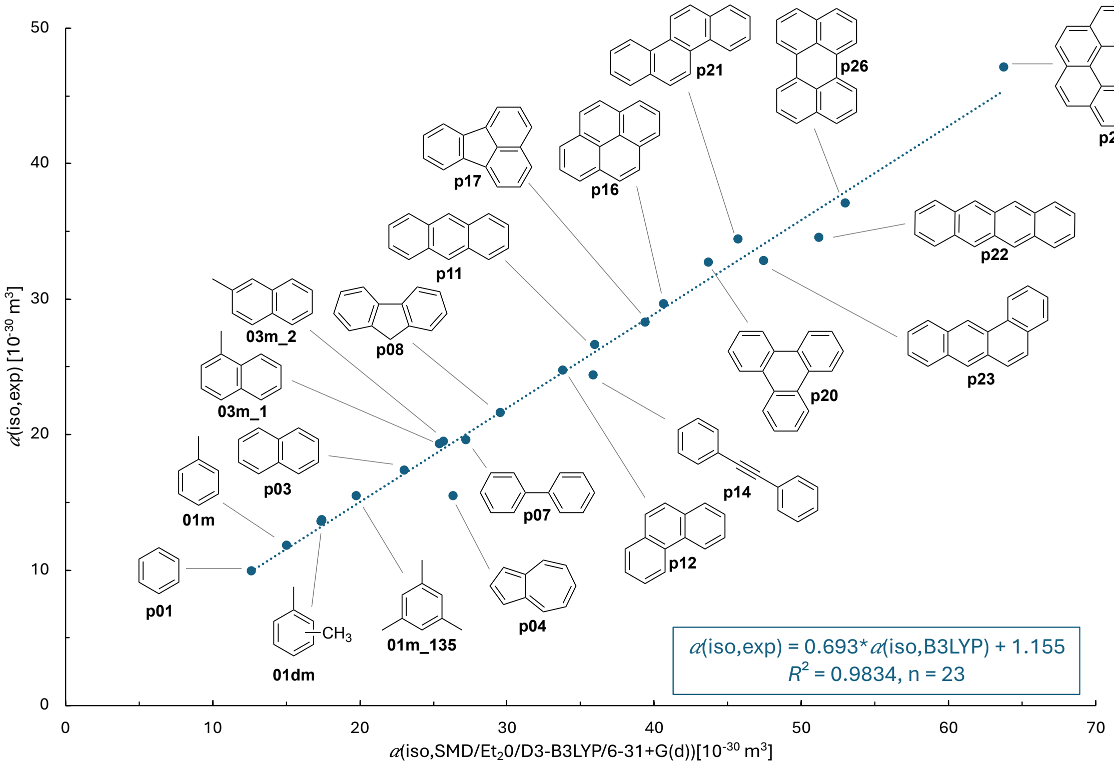

Figure S10. Correlation between experimental gas phase isotropic polarizability α(iso,exp) and solution phase isotropic polarizability α(iso,B3LYP) calculated at the SMD(Et_2O)/D3-B3LYP /6-31+G(d) level of theory.

Table S5. Results for naphthalene (**p03**) calculated at several levels of theory.

Entry	file	level of theory	E_{tot} (Hartree)	α(iso) [10^{-30} m^3]
1		exp.[a]		17.40
2	pi_002w	B3LYP-D3/6-31+G(d)	-385.9181771	17.27
3	pi_002p	SMD(Et_2O)/B3LYP-D3/6-31+G(d)	-385.9303319	23.03
4	pi_002y	M06-2X/6-31G(d)	-385.7188067	14.40 (14.21)[c]
5	pi_002z	M06-2X/6-31+G(d)	-385.7298679	16.71
6	pi_002v	M06-2X/cc-pVTZ	-385.8552780	16.19
7	pi_002x	M06-2X/def2-TZVPP	-385.8629525	16.69
8	pi_002u	M06-2X/aug-cc-pVTZ	-385.8586521	17.38
9	pi_002t	SMD(Et_2O)/M06-2X/6-31+G(d)	-385.7426020	22.15

[a] Experimental values as described by Gussoni et al.[19]; [c] Computed value taken from Sastry et al.[20] where a value of 95.921 (au^3) has been reported. Unit conversion then yields α(iso) = 14.21 (10^{-30} m^3).

The value of +23.03 calculated for naphthalene (**p03**) (entry 3, Table S5) at the SMD(Et_2O)/D3-B3LYP/6-31+G(d) level of theory as compared to the experimental gas phase value of 17.40 (entry 1) may serves as an illustrative example here. Further analysis of polarizability values calculated with selected other computational methods indicates, that the enlarged polarizabilities obtained with the SMD solvation model simply reflect a combination of the

polarizability of the molecular system itself as well as that of the solvation model. The good correlation coefficient found for SMD(Et_2O)/D3-B3LYP/6-31+G(d) calculations for the references data set shown in Figure S9 and Figure S10 also shows this effect to be highly systematic in nature.

For the complete dataset shown in Figure S1 and Table S1, the correlation of isotropic polarizabilities with molecular volumes can be explored for the systems **p01 - p34** (Figure S11). This now also includes the largest carbocyclic systems (up to heptacene, **p34**). Unfortunately, experimental data only exist for 23 out of the 34 systems. A good linear correlation with R^2 = 0.9542 is found. Despite the (formally) identical units employed for both quantities (Å^3 or 10^{-30} m^{-3}), the slope and intercept parameters can in this case not be used directly to make further statements on the meaning of this correlation simply because the absolute range of volume and polarizability data are quite different. This can be addressed by using normalized instead of absolute values.

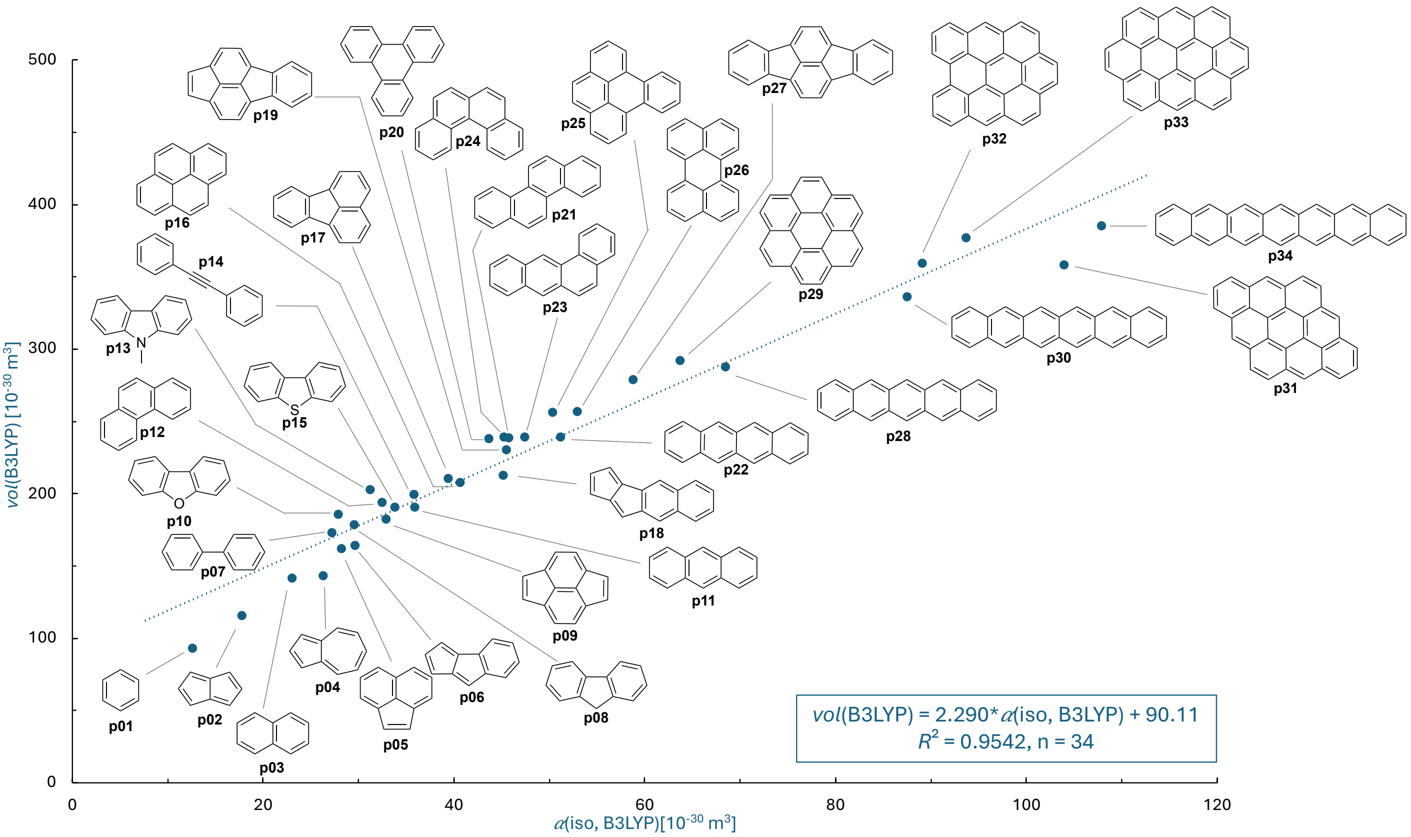

Figure S11. Correlation between molecular volumes (*vol*(B3LYP) in 10^{-30} m^{-3}) and isotropic polarizabilities (α(iso,B3LYP)) for aryl systems **p01 - p34** (data from Table S1) calculated at the SMD(Et_2O)/D3-B3LYP /6-31+G(d) level of theory.

Normalized cavity volumes (vol_n(**px**)) or polarizabilities (α_n(iso, **px**)) can be obtained by dividing the value for a given system by that for of the largest system. The largest system studied here (by molecular volume) is heptacene (**p34**) with the following values: *vol* = 385.266x10^{-30} m^{-3} and α(iso) = 107.92x10^{-30} m^{-3}, both calculated at the SMD(Et_2O)/D3-B3LYP /6-31+G(d) level of theory. The above definitions give vol_n(**px**) and α_n(iso, **px**) as defined in Eq. 1 and Eq. 2 respectively.

$$vol_{\text{n}}(\mathbf{px}) = \frac{vol\ (\mathbf{px})}{385.266} \qquad \text{Eq. 1}$$

$$\alpha_{\text{n}}(\text{iso}, \mathbf{px}) = \frac{\alpha(\text{iso},\ \mathbf{px})}{107.92} \qquad \text{Eq. 2}$$

Normalized cavity volume ranges from vol_n(**p01**) = 93.289/385.266 = 0.24 for benzene to 1.0 for heptacene (**p34**), and normalized polarizabilities range from α_n(iso, **p01**)) = 12.62 / 107.92 = 0.12 for benzene to 1.0 for heptacene. Of consequence, the normalized values are, by definition, dimensionless, and are thus practically independent from their absolute values. For the sake of correlating experimental rate and selectivity data, the use of normalized polarizability values is clearly preferrable. The correlation of normalised isotropic polarizabilities with the normalised molecular volumes can be found in Figure S12. The slope amounts to 0.821, which implies the polarizability is the more sensitive parameter here as compared to the corresponding molecular volumes, suggesting that size alone does not fully determine the electronic polarizability for the systems studied here. This also shows that, while size is a good indicator for predicting polarizability, as the larger the systems grow, the quality of the prediction reduces. The correlation coefficient shown in Figure S11 and Figure **S12** is, of course, identical.

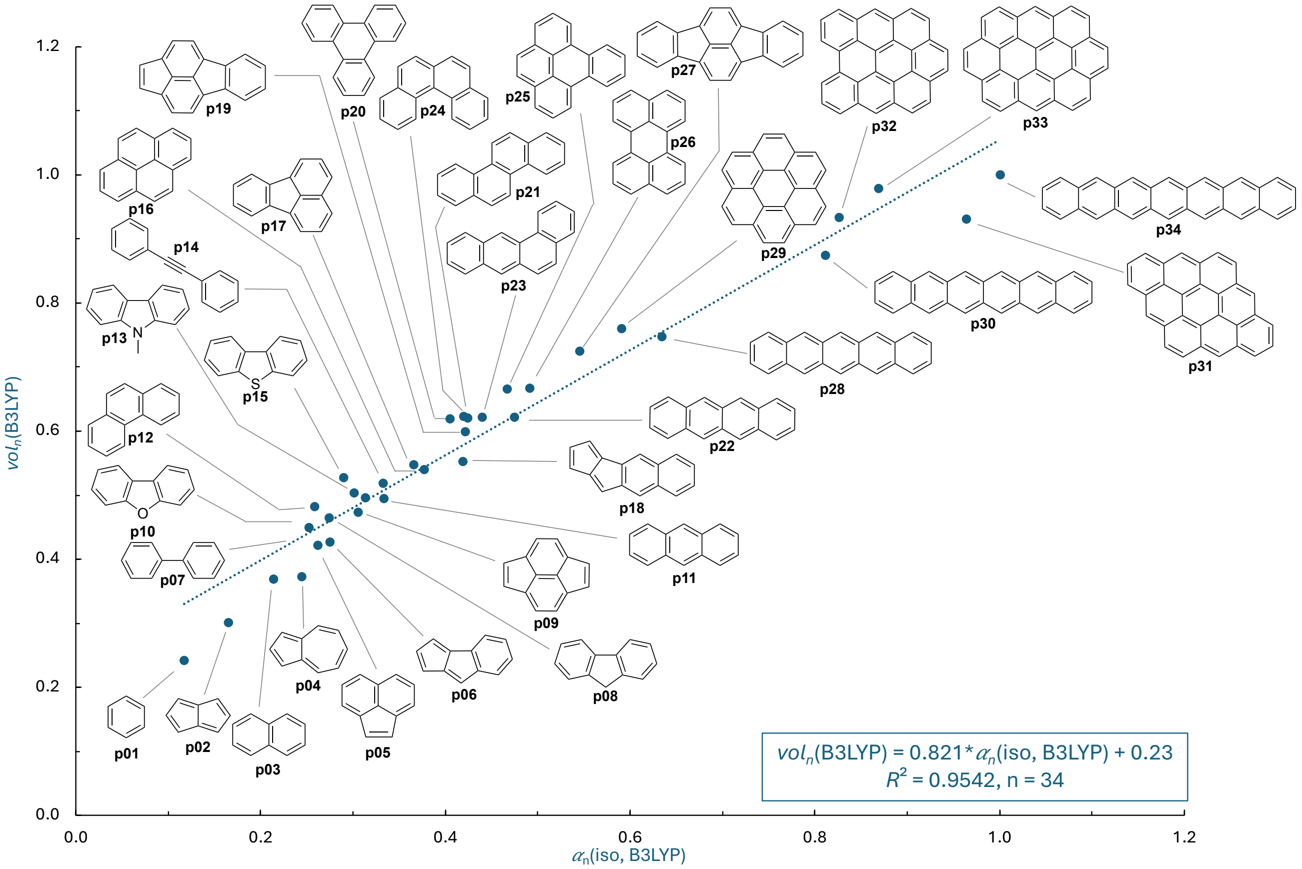

Figure S12. Correlation between normalized molecular volumes (vol_n(B3LYP)) and normalized isotropic polarizabilities (α_n(iso, B3LYP)) for aryl systems **p01 - p34** (data from Table S1) calculated at the SMD(Et_2O)/D3-B3LYP/6-31+G(d) level of theory.

Despite the benefits of using normalized data for correlation analysis, communicating the results to a broad audience may still be easier in conventional (non-normalized) units. As outlined above, several arguments exist for using normalized polarizability α_n data obtained from calculations at a given quantum chemical level. As this is not so for molecular volume data, we will in the following analyses employ non-normalized molecular volumes (in units of 10^{-30} m^{-3}) and normalized isotropic molecular polarizabilities α_n (as dimensionless properties). For the systems **p01** - **p34** shown in Figure S1, this yields the correlation shown in Figure S13.

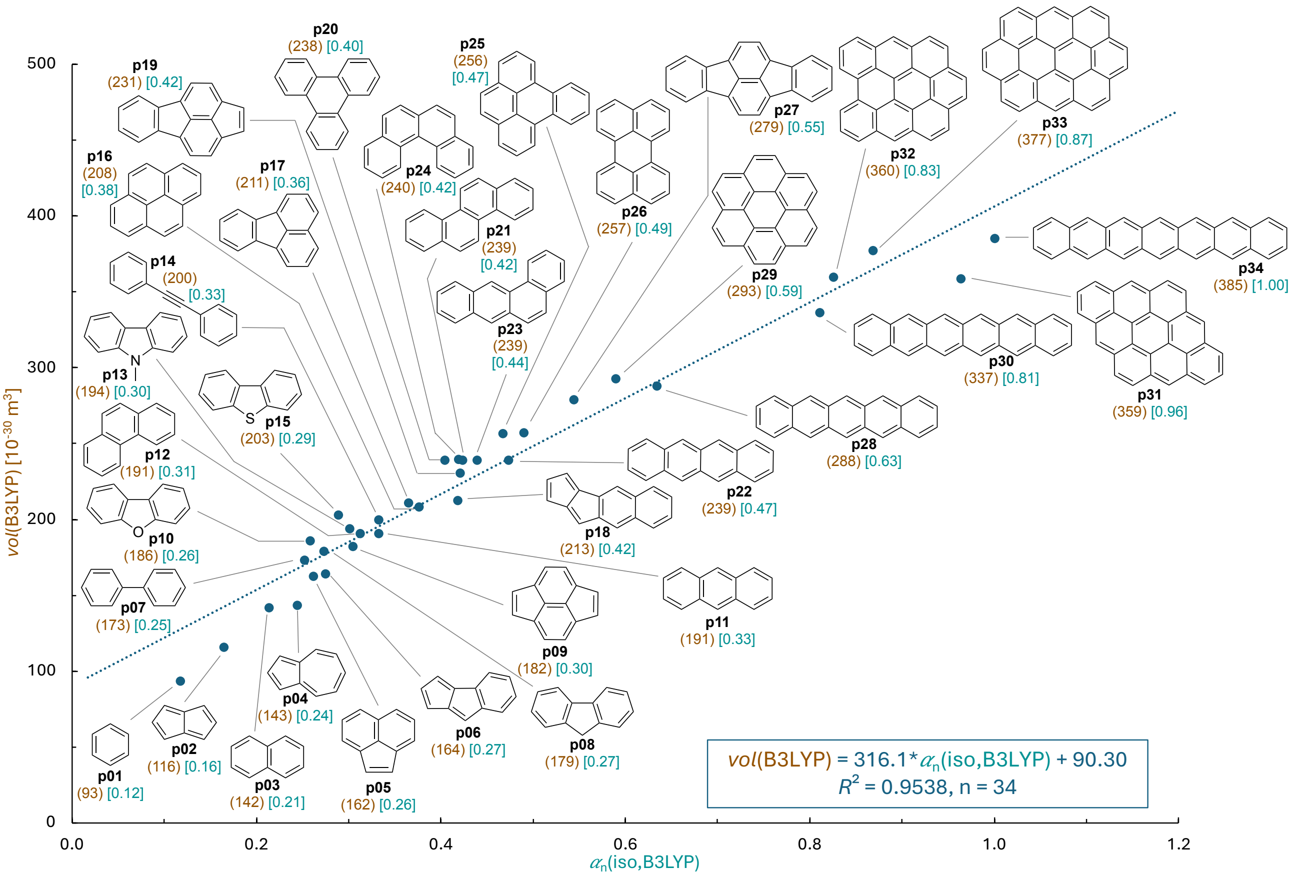

Figure S13. Correlation between molecular volumes (vol(B3LYP) in 10^{-30} m^{-3}) and normalized isotropic polarizabilities (α_n(iso, B3LYP)) for aryl systems **p01 - p34** (data from Table S1) calculated at the SMD(Et_2O)/D3-B3LYP/6-31+G(d) level of theory.

4.2. Effects of methyl substitution

Negligible variations in size and polarizability were observed between the different regioisomers of methyl substitutions. For example, a 1-Me substitution for naphthalene (**03m_1**) has a molecular volume of 159.145 10^{-3} m^3 while a 2-Me substitution (**03m_2**) one of 159.489. In both cases, the normalized isotropic polarizability α_n is 0.24. Therefore, to simplify data treatment for correlation analysis, only one methylated position per system **01m - 29m** was selected, as shown in Figure S14. The linear regression is very good with $R^2 = 0.9463$. However, to remain close to what is synthetically achievable, calculations were only performed up to coronene. The regression slope is steeper at 403.31 the one for aryl systems **p01 - p34**, because it does not include systems up to heptacene.

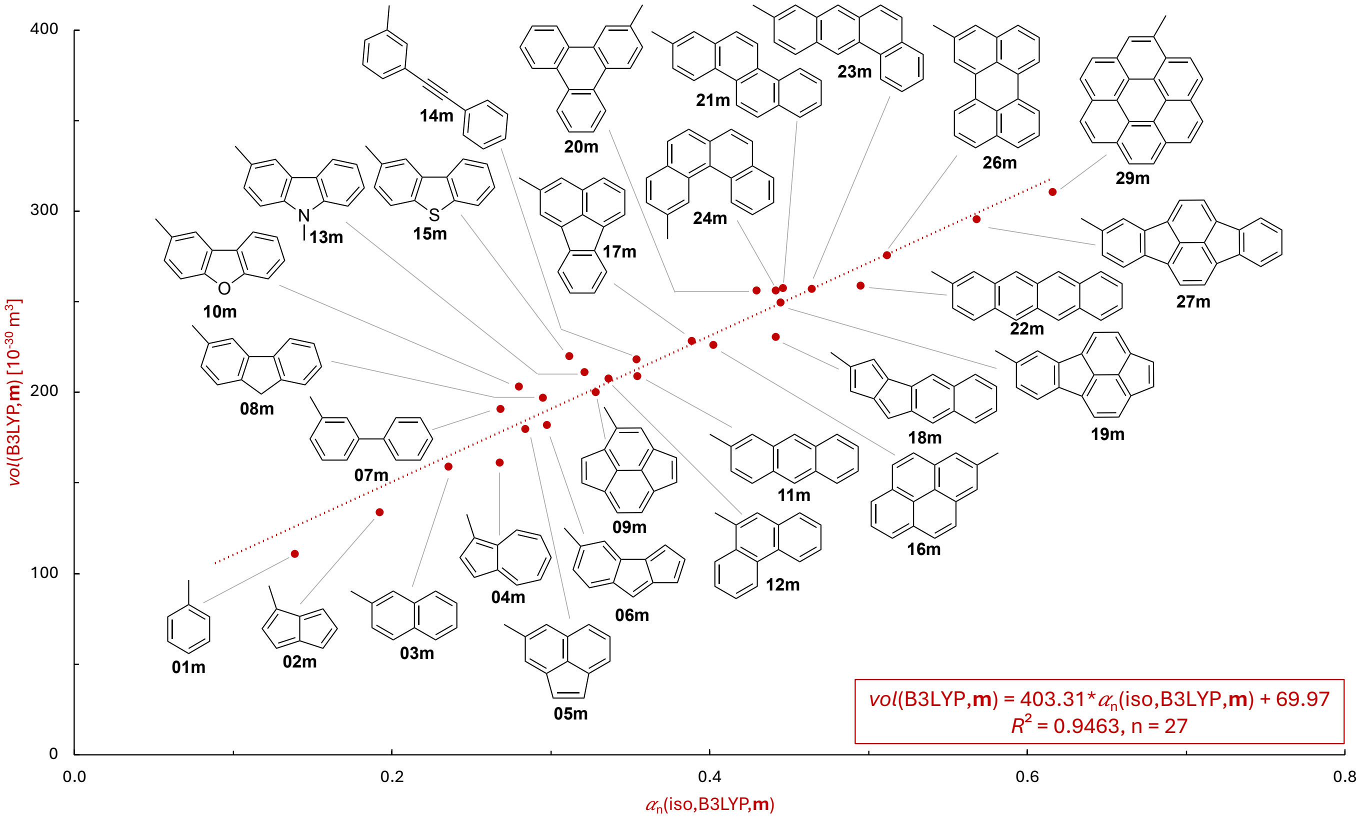

Figure S14. Correlation between molecular volumes (*vol*(B3LYP,**m**) in 10^{-30} m^{-3}) and normalized isotropic polarizabilities (α_n(iso,B3LYP,**m**)) for methylated aryl systems **01m - 29m** (data from Table S3) calculated at the SMD(Et_2O)/D3-B3LYP/6-31+G(d) level of theory.

To be able to compare the influence of methylation on their equivalent aryl systems, the molecular volumes and polarizabilities of systems **01m - 29m** was plotted against that of systems **p01 - p29** (Figure S15). In both cases the slope is of 1.00, with excellent correlation coefficients ($R^2 > 0.999$), indicating that the addition of a methyl group to the π-system consistently increases the molecular volume by 17.72 Å^3 and the isotropic polarizability by 2.34 Å^3. Using a simplified version of the system, that is the equivalent benzenoid system **p01 - p29** instead of the methylated **01m - 29m**, will give the same reliable and consistent results, with a simple shift in values.

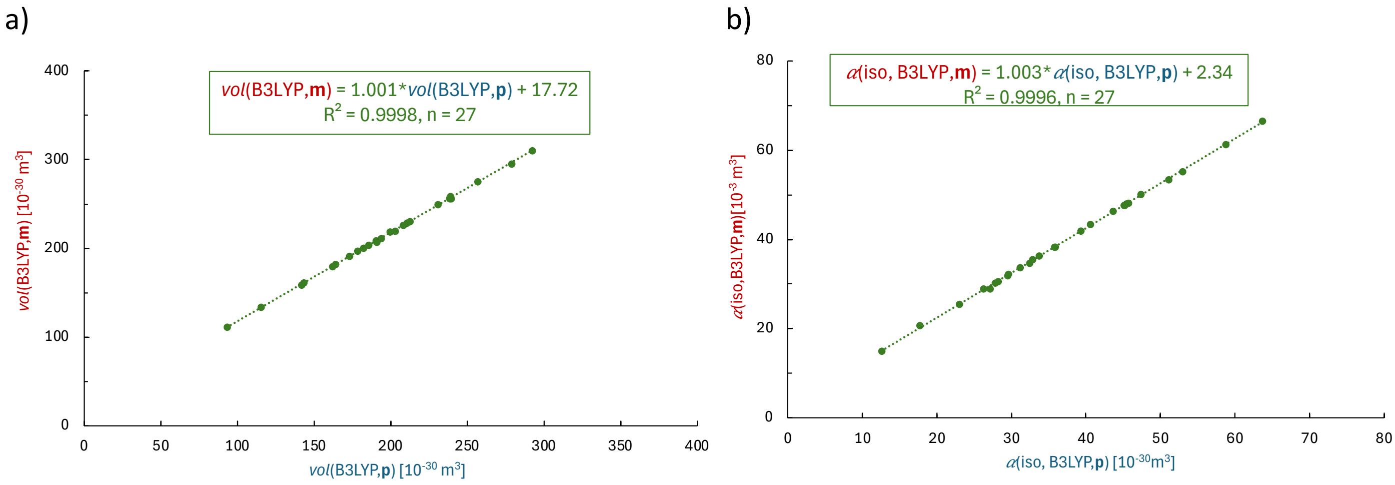

Figure S15. Correlation between a) molecular volumes and b) isotropic polarizabilities of methylated systems (**m**, in red: data in Table S2) and their parent benzenoid system (**p**, in blue data in Table S1) calculated at SMD(Et_2O)/D3-B3LYP/6-31+G(d) level of theory.

4.3. Effects of alcohol substitutions.

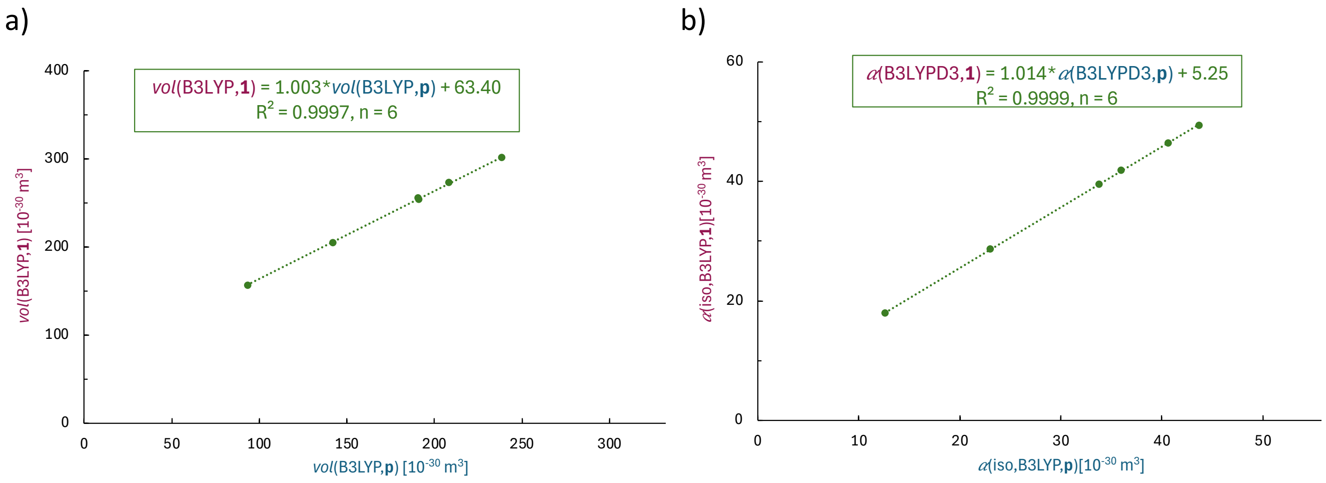

Figure S16. Correlation between a) molecular volumes and b) isotropic polarizabilities for the alcohols **1a**, **1b**, **1c**, **1d**, **1o**, **1p**, and **1r** (data in Table S3) and their parent benzenoid system (**p**, data in Table S1) calculated at SMD(Et_2O)/D3-B3LYP/6-31+G(d) level of theory.

The influence of using the full substrate structure compared to the parent side chain system was evaluated for alcohols **1a**, **1b**, **1c**, **1d**, **1o**, **1p**, and **1r**. Once again a near perfect slope of 1 was obtained with R^2 values > 0.999 (Figure S16). Using the full structure of the alcohol over the parent π-system thus consistently increases the molecular volume by 63.40 Å^3 and the isotropic polarizability by 5.25 Å^3. Using the parent π-system instead of the whole alcohol was estimated to be sufficiently reliable. This theory was tested in Figure S17.

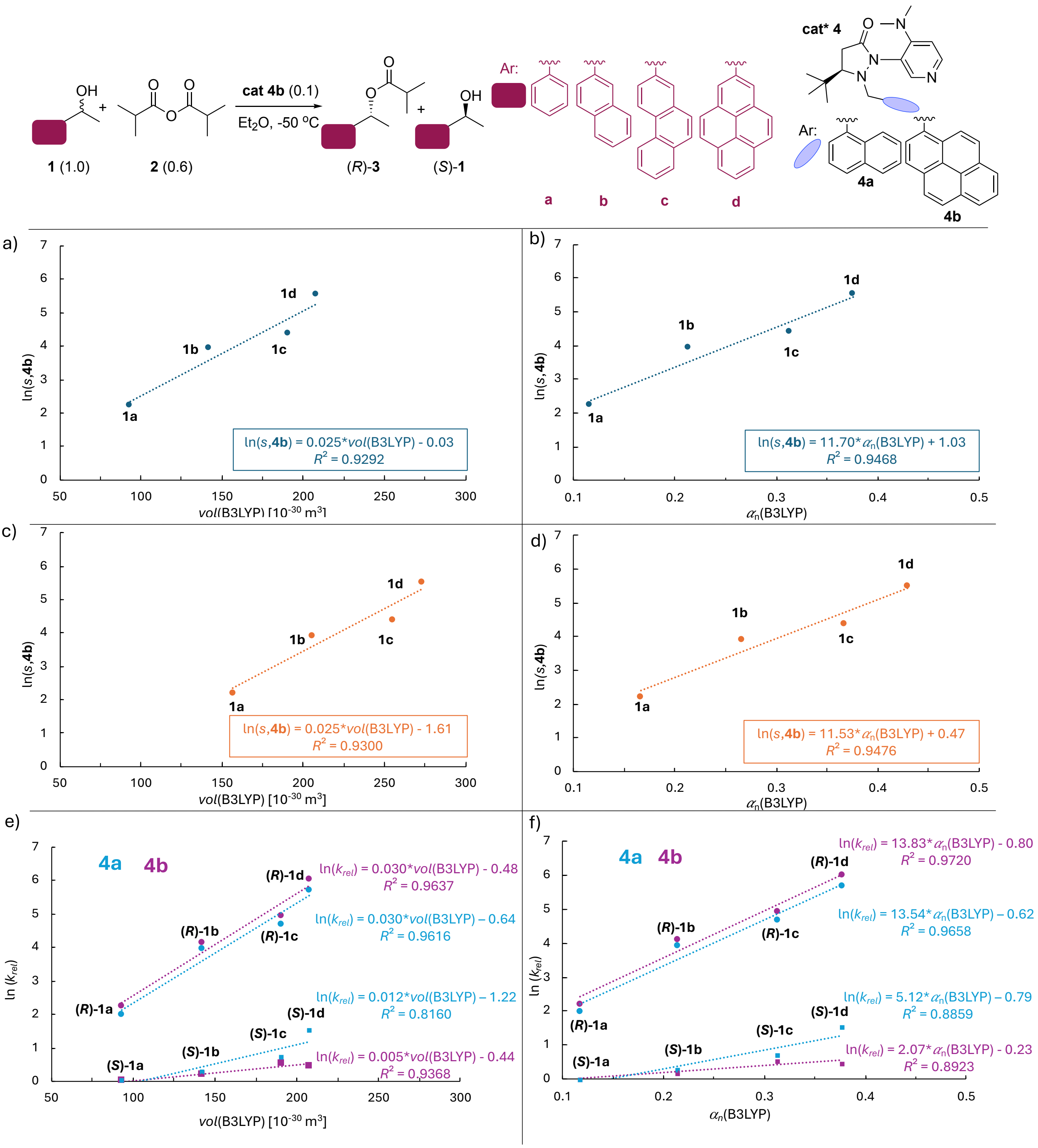

Figure S17. Relationship between reaction selectivity ln(*s*,**4b**) for the kinetic resolution of **1** with **2** with catalyst **4b**, with the a) molecular volume *vol* and b) normalized side chain isotropic polarizability α_n of the parent π-system **p** (in blue: **p01** for **1a**, **p03** for **1b**, **p12** for **1c**, and **p16** for **1d**) or the c) molecular volume *vol* and d) normalized side chain isotropic polarizability α_n of the whole alcohol (in orange: **1a, 1b, 1c** and **1d**). Relationship between reaction rate (k_{eff}) of **(*R*)-1** (round) and **(*S*)-1** (square) and the e) molecular volume *vol* or d) normalized side chain isotropic polarizability α_n of the parent π-system **p** for catalysts **4a** (blue) and **4b** (purple).

Exploring relationships between selectivity data and size was done for the reaction shown in Figure S17. Expressing the reaction selectivity as the natural logarithm ln(*s*,**4b**), we obtain the positive correlation of reaction selectivity with the molecular volume of the aromatic substrate side chain (Figure S17a). The slope of +0.025 of this correlation implies a strong dependence of reaction selectivity on side chain size, and the correlation coefficient is impressively good at R^2 = 0.9292 considering the simplicity of this single descriptor approach. In Figure S17b we see a similarly positive correlation of reaction selectivity with the normalized polarizability α_n, where the slope of +11.70 again signals a strong increase in selectivity with increasing polarizability. The correlation coefficient is in this case even better at R^2 = 0.9468. One of the assumptions made in these analyses is that properties of the aromatic side chain are sufficient to capture the essence of the complete alcohol substrates. How big the error is in making this assumption can be tested by repeating both analyses with molecular volume and polarizability data for the complete alcohol substrates (and not only their side chains). The results of these analyses are shown in Figure S17c and Figure S17d that, on first glance, appear almost identical to Figure S17a and b, with a simple shift for increased values. It is only from the marginally improved correlation coefficients (R^2 = 0.9300 vs. 0.9292 for molecular volumes, and R^2 = 0.9476 vs. 0.9468 for polarizabilities α_n) that we see a small advantage of using whole substrate values for analysis. We can thus conclude that the error made in using side chain molecular volumes and polarizabilities as a surrogate for complete substrate values is minimal and that the most relevant substrate properties responsible for the size-dependent increase in selectivity and reactivity are well represented by the side chain properties alone.

4.4. Heteroaromatic systems

The influence of heteroatoms in aryl systems on the correlation of molecular volumes and normalized isotropic polarizability was explored. In Figure S18, all 29 systems **h01 - h29** containing a single O, S or N heteroatom, or a mixture (N+O or S+O) was plotted, a slope of 495.01 was obtained with a good correlation coefficient of R^2 = 0.9393. For similar systems, those containing one O atom systematically have a lower α_n compared to systems with one N atom. For example, **h15** has an α_n of 0.16 compared to 0.20 for **h20**. Similar observations can be made for **h03**, **h10**, and **h23** (O-containing systems) compared to respectively **h07**, **h11**, and **h26** (N-containing systems). N-containing systems thus need a smaller scaling factor compared to O-containing systems.

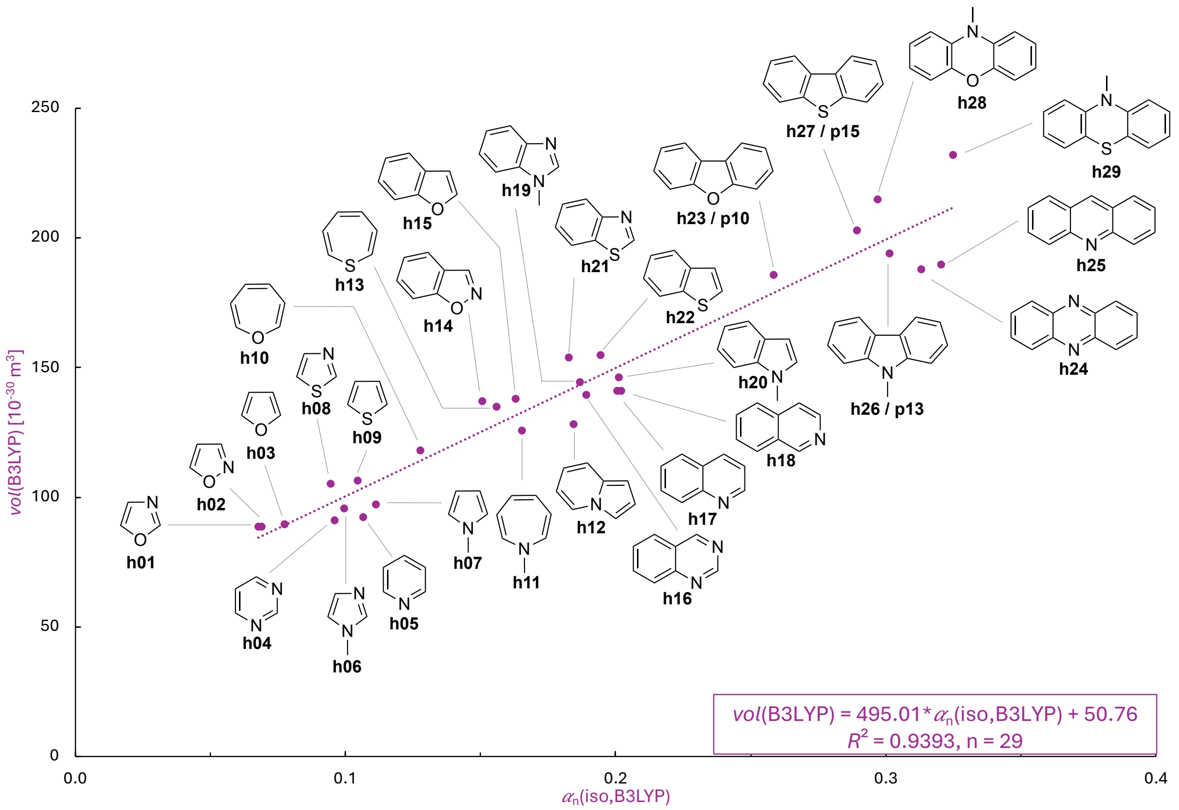

Figure S18. Correlation between molecular volumes (in 10^{-30} m^{-3}) and normalized isotropic polarizabilities for heteroaromatic systems **h01 - h29** (data in Table S4) calculated at the SMD(Et_2O)/D3-B3LYP/6-31+G(d) level of theory.

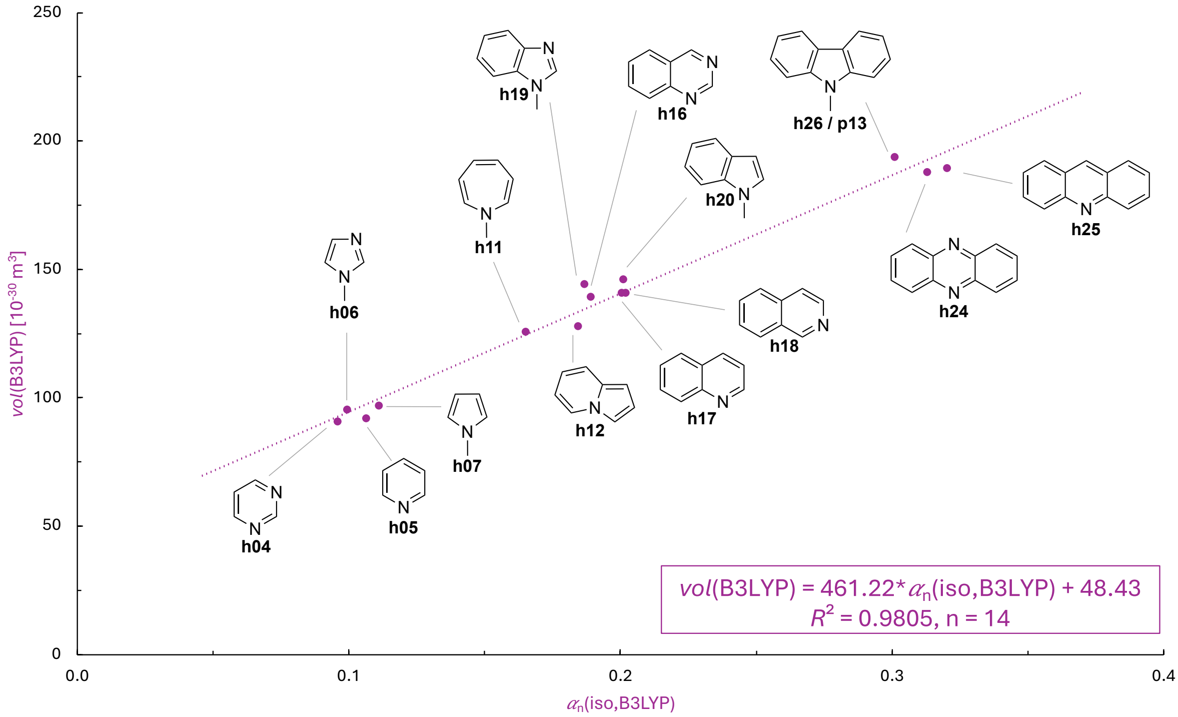

Figure S19. Correlation between molecular volumes (in 10^{-30} m^{-3}) and normalized isotropic polarizabilities for systems **h** with only N heteroatoms (data in Table S4) calculated at the SMD(Et_2O)/D3-B3LYP/6-31+G(d) level of theory.

If only the N-containing systems were plotted (Figure S19), the slope is lower at 461.22, with a superior correlation coefficient with R^2 = 0.9805. Adding an N to the aryl system systemically yields a horizontal shift to lower polarizability. For example, pyrimidine **h04** is shifted to the left compared to pyridine **h05** with little influence on the overall size of the system. For all other systems (only O or S, mix with N+O or N+S, as shown Figure S20) another excellent correlation coefficient is obtained (R^2 = 0.9952), with a steeper slope of 544.06. However, for all three figures, the y intercept remains ~50, indicating that the varying heteroatoms influence isotropic polarizability significantly more than molecular volumes. These results indicate that even though molecular volume remains a strong predictor for polarizability in aryl systems containing heteroatoms, atom specific polarizability effects are observed on top of size-based trends. The steeper slope indicates that O and S atoms contribute more to polarizability compared to N. However, in systems where there are both N and O/S atoms, cooperative electronic effects seem to play a role and increase the role of N in polarizability effects.

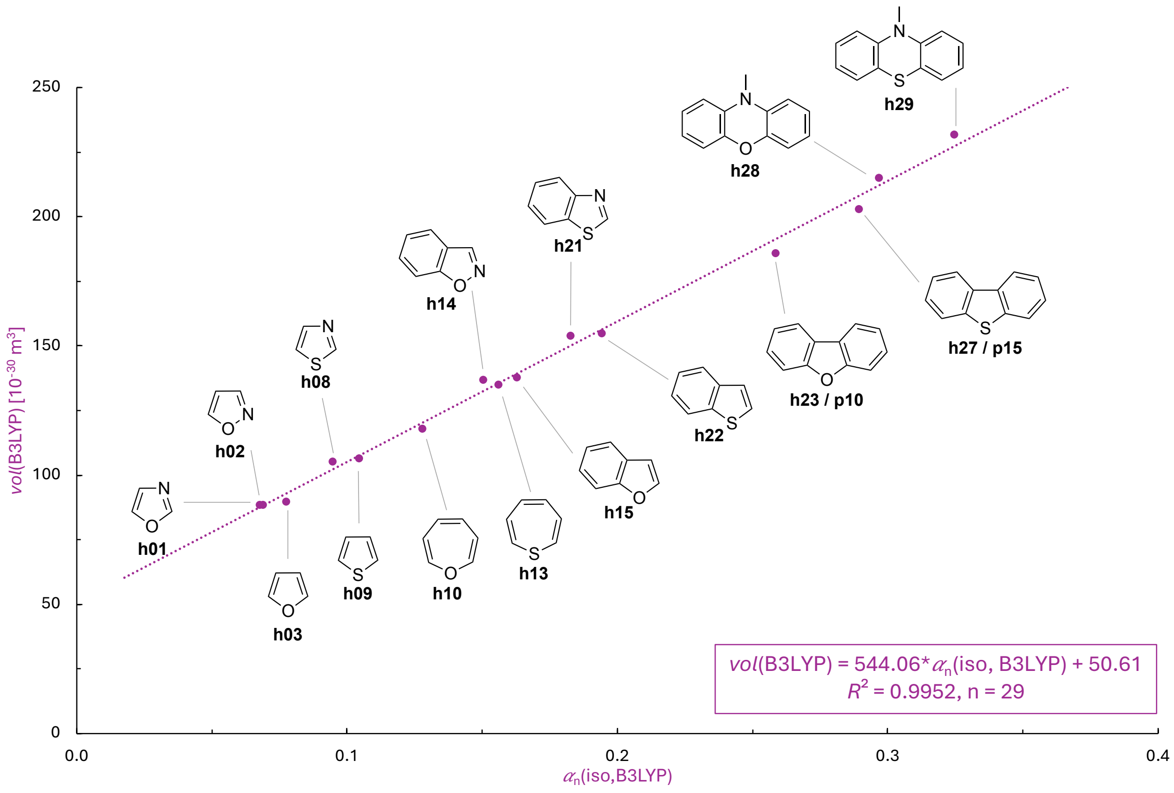

Figure S20. Correlation between molecular volumes (in 10^{-30} m^{-3}) and normalized isotropic polarizabilities for systems **h** with O, S, O+N or S+ N heteroatoms (data in Table S4) calculated at the SMD(Et_2O)/D3-B3LYP/6-31+G(d) level of theory.

5. **Multi-descriptor model**

To build a multi-descriptor model for the kinetic resolution of alcohols with catalyst **24** reported by Carbery and coworker, we initially screened single descriptors that appeared to correlate with the experimentally observed selectivity values in logarithmic form (ln(*s*)).[21] These descriptors were divided into subgroups based on their chemical meaning. In this manner, we analyzed three different subgroups: geometrical, charge-based, and polarizability-based descriptors. The used nomenclature for the tested descriptors is shown in Figure S21.

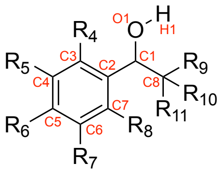

Figure S21. Used nomenclature for Atoms in the studied alcohols of Carbery and coworkers.[21]

The following geometrical descriptors were analyzed regarding their correlation to ln(*s*):

- percent buried volume (%V_{bur}) was calculated with respect to the oxygen atom of the alcohol as the reaction center. The hydrogen atom of the OH group was omitted from the calculation.
- O1 H1 atom distance (D_1)
- C1 O1 atom distance (D_2)
- angle between O1, C1, C2 (ϑ_1)
- angle between O1, C1, C8 (ϑ_2)
- dihedral angle between O1, C1, C2, C3 (ϑ_3)
- dihedral angle between O1, C1, C2, C4 (ϑ_4)

The following charge-based descriptors were analyzed regarding their correlation to ln(*s*):

- charges only based on the aryl group
  - NBO charge of C2 (q_1)
  - average NBO charges of C2, C3, C4, C5, C6, C7 (q_2)
  - sum of NBO charges of the whole aryl moiety (q_3)
- charges only based on the alkyl group
  - NBO charge of C8 (q_4)
  - sum of NBO charge of the whole alkyl moiety (q_6)
- charges only based on both alkyl and aryl groups
  - difference in NBO charges of C2 and C8 (q_7)
  - difference of sum of NBO charges of the alkyl and aryl group (q_8)

The following polarizability-based descriptors were analyzed regarding their correlation to ln(*s*):

- polarizability based on the whole molecule
  - normalized polarizability of the whole molecule (α_1)

- polarizability based on the aryl group
    - local polarizability of C2 (α_2)
    - average local polarizability charges of C2, C3, C4, C5, C6 and C7 (α_3)
    - sum of local polarizability of the whole aryl moiety (α_4)
- charges only based on the alkyl group
    - local polarizability of C8 (α_5)
    - sum of local polarizability of the whole alkyl moiety (α_6)
- Charges only based on both alkyl and aryl groups
    - difference in local polarizability of C2 and C8 (α_7)
    - difference in sum of local polarizability of the alkyl and aryl group (α_8)

The correlations between ln(*s*,**24**) and the computed geometrical descriptors (Table S6), charge-based descriptors (Table S7), and polarizability-based descriptors (Table S8) are shown in the corresponding tables. For further model development, only descriptors with a coefficient of determination (R^2) greater than 0.2 toward ln(*s*,**24**) were used for additional analysis. Surprisingly, no descriptors based only on the alkyl moiety showed sufficient correlation with ln(*s*,**24**), which is likely related to the fact that the 12 alcohols studied (**1a** - **1n**) vary from a methyl group in only 2 cases (**1m** and **1n**), but also highlights that the transition state (TS) of the studied reaction is dominated by the aryl moiety of the alcohol.

A Pearson correlation (R) matrix (Figure S22), R^2 values (Figure S23), and *p*-value matrix (Figure **S24**) of the descriptors selected for further study are shown in the corresponding figures. Polarizability-based descriptors with R^2 values smaller than 0.5 relative to other descriptors were then used to build a dual-descriptor model, which is shown in Figure S25. For all analyzed models, the combination of ϑ_1 with α_1 gave the most satisfactory result with an adjusted R^2 value of 0.86 for the predicted ln(*s*,**24**) values compared to the experimentally observed ones.

We also built dual-descriptor models using the best-performing single descriptor α_3 combined with ϑ_1 or q_1, as shown in Figure S26. However, both models performed worse compared to α_1 combined with ϑ_1. Furthermore, we investigated triple-descriptor models with one charge-based, one geometry-based, and one polarizability-based descriptor using the same criteria as before. The predicted ln(*s*,**24**) values compared to the experimentally observed values showed worse correlation in terms of adjusted R^2 value compared to the ϑ_1 combined with α_1 descriptor model (see Figure S27). However, the model combining ϑ_1, α_1, and q_1 gave a comparable adjusted R^2 of 0.85 compared to the ϑ_1 with α_1 model.

Table S6. Computed geometrical descriptors for the studied alcohols and measured ln(*s*,**24**) values.[21] Correlation between ln(*s*,**24**) and the computed geometrical descriptors is given as Pearson correlation coefficient R and R^2.

Token	ln(*s*,**24**)	$\%V_{bur}$	D_1 [Å]	D_2 [Å]	ϑ_1 [°]	ϑ_2 [°]	ϑ_3 [°]	ϑ_4 [°]
a1	2.83	36.8	0.97235	1.44048	111.915	106.22	137.123	-44.207
a15	2.89	36.8	0.97241	1.44051	111.716	106.167	134.559	-46.799
a16	3.14	37.1	0.97216	1.44100	111.641	105.937	134.378	-47.059
a17	3.26	45.7	0.97150	1.43862	111.114	107.242	143.327	-36.340
a2	3.50	37.4	0.97239	1.43675	112.356	106.051	158.584	-23.105
a3	3.50	36.8	0.97241	1.44255	111.372	106.191	127.933	-52.255
a11	3.14	37.5	0.97235	1.43440	112.028	106.354	155.923	-25.984
a12	3.47	37.4	0.97229	1.43916	112.352	106.040	150.856	-31.326
a13	3.76	39.0	0.97351	1.43860	112.459	111.146	128.042	-53.300
a14	3.30	37.8	0.97229	1.44419	111.021	105.79	131.551	-46.379
a10	3.95	42.3	0.97176	1.44136	113.528	106.975	140.948	-40.939
a4	4.75	41.3	0.97212	1.44295	113.668	107.155	136.611	-45.309
R	-	0.4327	-0.010	0.2874	0.7646	0.3604	-0.083	-0.1167
R^2	-	0.1872	0.000	0.0826	0.5845	0.1299	0.007	0.0136

Table S7. Computed charge-based descriptors for the studied alcohols and measured ln(*s*,**24**) values.[21] Correlation between ln(*s*,**24**) and the computed geometrical descriptors is given as Pearson correlation coefficient R and R^2. Descriptors have been subdivided into ones that only depend on the aryl, alkyl or both.

		Aryl			Alkyl		Both	
Token	ln(*s*,**24**)	q_1	q_2	q_3	q_4	q_6	q_7	q_8
a1	2.83	-0.078	-0.218	-0.040	-0.694	0.044	0.616	0.084
a15	2.89	-0.088	-0.123	-0.044	-0.694	0.045	0.606	0.089
a16	3.14	-0.079	-0.218	-0.039	-0.479	0.035	0.400	0.074
a17	3.26	-0.074	-0.217	-0.036	-0.118	0.010	0.044	0.046
a2	3.50	-0.045	-0.146	-0.040	-0.694	0.046	0.649	0.086
a3	3.50	-0.075	-0.145	-0.043	-0.696	0.043	0.621	0.086
a11	3.14	-0.088	-0.193	-0.066	-0.694	0.051	0.606	0.117
a12	3.47	-0.072	-0.183	-0.044	-0.692	0.043	0.620	0.087
a13	3.76	-0.107	-0.140	-0.035	-0.706	0.023	0.599	0.058
a14	3.30	-0.061	-0.178	-0.2806	-0.691	0.043	0.630	0.324
a10	3.95	-0.082	-0.109	-0.039	-0.692	0.039	0.610	0.078
a4	4.75	-0.011	-0.080	-0.057	-0.696	0.044	0.685	0.101
R	-	0.5958	0.7385	0.0703	-0.1904	-0.0286	0.2697	-0.0719
R^2	-	0.3550	0.5453	0.0049	0.0363	0.0008	0.0727	0.0052

Table S8. Computed polarizability-based descriptors for the studied alcohols and measured ln(*s*,**24**) values.[21] Correlation between ln(*s*,**24**) and the computed geometrical descriptors is given as Pearson correlation coefficient *R* and R^2. Descriptors have been subdivided into ones that only depend on the aryl, alkyl, both or the whole molecule.

		Whole Molecule	*Aryl*			*Alkyl*		*Both*	
Token	ln(*s*,**24**)	α_1 [10^{-3} m^3]	α_2 [a.u.]	α_3 [a.u.]	α_4 [a.u.]	α_5 [a.u.]	α_6 [a.u.]	α_7 [a.u.]	α_8 [a.u.]
a1	2.83	0.17	9.604	9.553	77.377	8.969	20.800	0.635	56.577
a15	2.89	0.16	9.565	9.533	76.723	8.957	20.719	0.608	56.004
a16	3.14	0.19	9.573	9.526	77.151	9.162	37.692	0.411	39.459
a17	3.26	0.23	9.569	9.522	77.113	9.553	72.708	0.016	4.405
a2	3.50	0.26	9.599	9.608	124.134	8.988	20.892	0.611	103.242
a3	3.50	0.26	9.59	9.595	124.027	8.969	20.766	0.621	103.261
a11	3.14	0.20	9.615	9.573	93.889	9.019	21.017	0.596	72.872
a12	3.47	0.19	9.602	9.596	94.271	8.977	21.185	0.625	73.086
a13	3.76	0.19	9.704	9.617	98.052	9.001	20.930	0.703	77.122
a14	3.30	0.29	9.575	9.556	150.534	8.984	20.804	0.591	129.73
a10	3.95	0.23	9.605	9.606	127.542	8.964	20.737	0.641	106.805
a4	4.75	0.38	9.564	9.653	170.862	8.944	20.777	0.62	150.085
R	-	0.7713	0.0840	0.8478	0.7706	- 0.2171	- 0.1788	0.2201	0.6736
R^2	-	0.5950	0.0071	0.7187	0.5938	0.0471	0.0320	0.0485	0.4538

	ln(*s*,**24**)	ϑ_1	q_1	q_2	α_1	α_3	α_4	α_8
ln(*s*,**24**)	1.0000	-	-	-	-	-	-	-
ϑ_1	0.7629	1.0000	-	-	-	-	-	-
q_1	0.5963	0.3531	1.0000	-	-	-	-	-
q_2	0.7389	0.6913	0.3773	1.0000	-	-	-	-
α_1	0.7723	0.3318	0.8566	0.5166	1.0000	-	-	-
α_3	0.8458	0.7808	0.4449	0.7291	0.5722	1.0000	-	-
α_4	0.7715	0.4416	0.7142	0.6585	0.9110	0.7050	1.0000	-
α_8	0.6742	0.5044	0.5845	0.7046	0.7393	0.7630	0.9368	1.0000

Figure S22. Pearson correlation (*R*) matrix of descriptors with $R^2 > 0.2$ towards ln(*s*,**24**).

	ln(s,**24**)	ϑ_1	q_1	q_2	α_1	α_3	α_4	α_8
ln(s,**24**)	1.0000	-	-	-	-	-	-	-
ϑ_1	0.5821	1.0000	-	-	-	-	-	-
q_1	0.3556	0.1247	1.0000	-	-	-	-	-
q_2	0.5459	0.4779	0.1424	1.0000	-	-	-	-
α_1	0.5965	0.1101	0.7337	0.2668	1.0000	-	-	-
α_3	0.7154	0.6097	0.1979	0.5316	0.3274	1.0000	-	-
α_4	0.5953	0.1950	0.5100	0.4337	0.8300	0.4970	1.0000	-
α_8	0.4545	0.2545	0.3416	0.4965	0.5466	0.5822	0.8776	1.0000

Figure S23. Coefficient of determination index (R^2) matrix of descriptors with R^2 > 0,2 towards ln(s,**24**). Polarizability-based descriptors with R^2 < 0.5 towards other descriptors are highlighted in green.

	ln(s,**24**)	ϑ_1	q_1	q_2	α_1	α_3	α_4	α_8
ln(s,**24**)	-	-	-	-	-	-	-	-
ϑ_1	0.0039	-	-	-	-	-	-	-
q_1	0.0407	0.2603	-	-	-	-	-	-
q_2	0.0060	0.0128	0.2266	-	-	-	-	-
α_1	0.0032	0.2921	0.0004	0.0855	-	-	-	-
α_3	0.0005	0.0027	0.1473	0.0071	0.0519	-	-	-
α_4	0.0033	0.1507	0.0091	0.0199	0.0000	0.0105	-	-
α_8	0.0162	0.0944	0.0460	0.0105	0.0060	0.0039	0.0000	-

Figure S24. p-value matrix of descriptors with R^2 > 0.2 towards ln(s,**24**).

a)

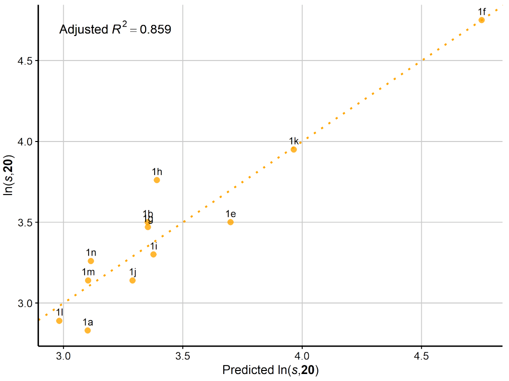

Predicted ln(s,**24**) = -37.13497+0.35207ϑ_1+4.91700α_1

b)

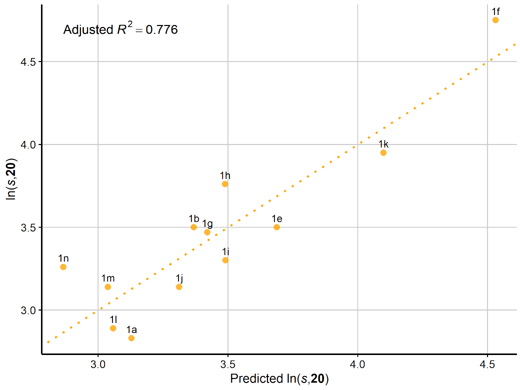

Predicted ln(s,**24**) = -33.859250+ 0.324335ϑ_1+0.008915α_4

c)

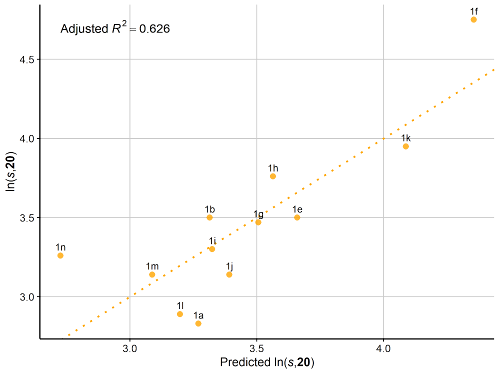

Predicted ln(s,**24**) = -36.263784+0.350705ϑ_1+0.005035α_8

d)

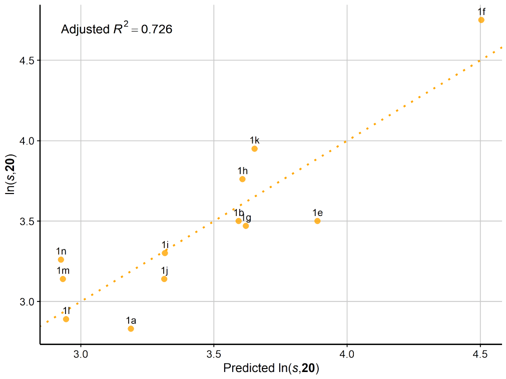

Predicted ln(s,**24**) = -84.381+ 5.882q_1+9.215α_3

e)

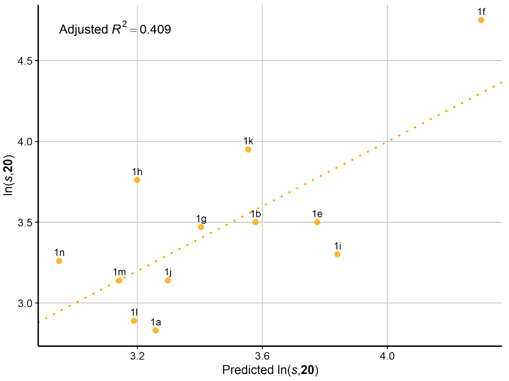

Predicted ln(s,**24**) = 3.409470+6.587382q_1+0.006417α_8

f)

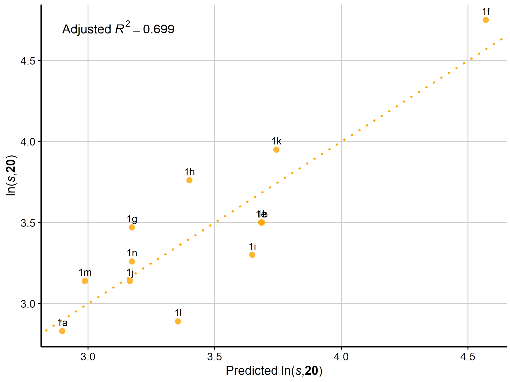

Predicted ln(s,**24**) = 3.2867+5.2816q_2+4.4907α_1

g)

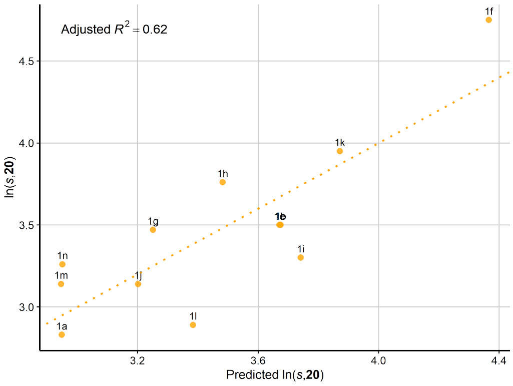

Predicted ln(s,**24**) = 3.317608+4.642386q_2+0.008308α_4

Figure S25. Dual linear regression analysis and plot of predicted ln(s,**24**) values against experimental ln(s,**24**) values for chosen descriptors: a) ϑ_1 and α_1, b) ϑ_1 and α_4, c) ϑ_1 and α_8, d) q_1 and α_3, e) q_1 and α_8, f) q_2 and α_1, and g) q_2 and α_4.

a)

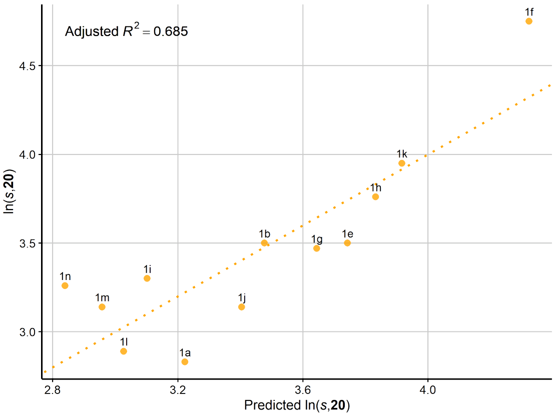

Predicted ln(s,**24**) = -92.8810+0.1624ϑ_1+8.1571α_3

b)

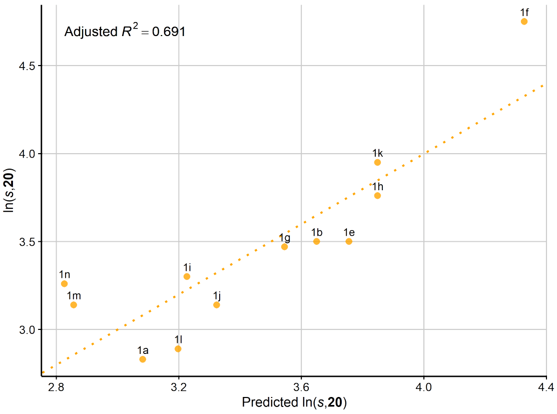

Predicted ln(s,**24**) = -92.8810+0.1624q_2+8.1571α_3

Figure S26. Dual linear regression analysis and plot of predicted ln(s,**24**) values against experimental ln(s,**24**) values for chosen descriptors: a) ϑ_1 and α_3, b) q_2 and α_3.

a)

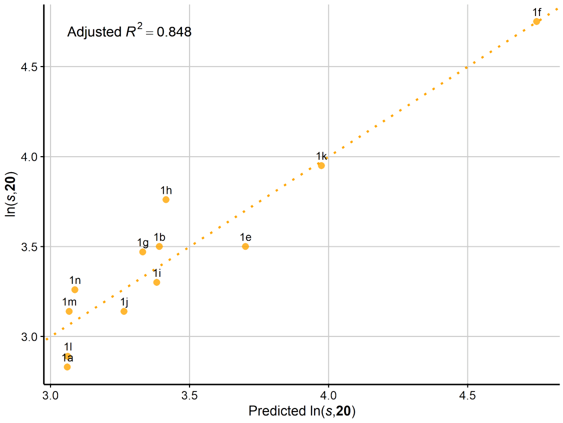

Predicted ln(s,**24**) = -32.7449+0.3152ϑ_1+1.1640q_2+4.6391α_1

b)

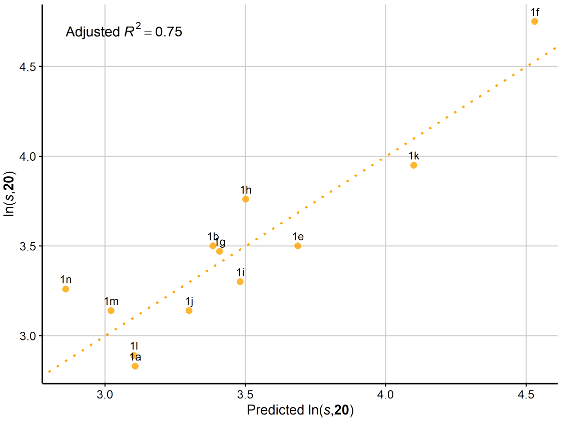

Predicted ln(s,**24**) = -31.765776+0.306984ϑ_1+0.642612q_2+ 0.008506α_4

c)

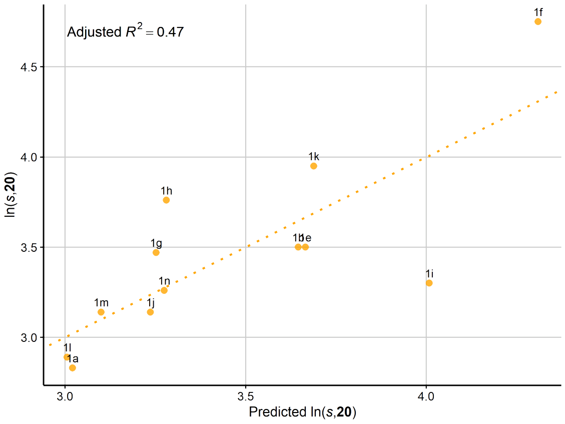

Predicted ln(s,**24**) = +1.930305-0.004911ϑ_1+0.582086q_1+0.018274α_4

Figure S27. Triple linear regression analysis and plot of predicted ln(s,**24**) values against experimental ln(s,**24**) values for chosen descriptors: a) ϑ_1, q_2 and α_1, b) ϑ_1, q_2 and α_1, and c) ϑ_1, q_2 and α_4.

6. Cartesian coordinates

6.1. Parent π-systems

```
12
pi_001.log       Energy: -145752.9017505
C          0.00000        1.39981       -0.00000
C          1.21227        0.69990       -0.00000
C          1.21227       -0.69990       -0.00000
C         -0.00000       -1.39981       -0.00000
C         -1.21227       -0.69990       -0.00000
C         -1.21227        0.69990       -0.00000
H          0.00000        2.48721       -0.00000
H          2.15398        1.24360       -0.00000
H          2.15398       -1.24360       -0.00000
H         -0.00000       -2.48721       -0.00000
H         -2.15398       -1.24360       -0.00000
H         -2.15398        1.24360       -0.00000
18
pi_002.log       Energy: -242174.8526969
C          0.00000        2.43726        0.70971
C          0.00000        1.24618        1.40555
C         -0.00000       -0.00000        0.71782
C          0.00000       -0.00000       -0.71782
C          0.00000        1.24618       -1.40555
C          0.00000        2.43726       -0.70971
H          0.00000       -1.24387        2.49375
H          0.00000        3.38238        1.24719
H          0.00000        1.24387        2.49375
C         -0.00000       -1.24618        1.40555
C         -0.00000       -1.24618       -1.40555
H          0.00000        1.24387       -2.49375
H          0.00000        3.38238       -1.24719
C         -0.00000       -2.43726       -0.70971
C         -0.00000       -2.43726        0.70971
H          0.00000       -1.24387       -2.49375
H         -0.00000       -3.38238       -1.24719
H         -0.00000       -3.38238        1.24719
18
pi_003.log       Energy: -242141.8846119
C          2.01608       -1.49092        0.00000
C          2.29294       -0.11760        0.00000
C          1.39460        0.95630        0.00000
C          0.78411       -2.15798        0.00000
C          0.00000        0.93210        0.00000
C         -0.50579       -1.61359        0.00000
C         -0.89124       -0.27303        0.00000
H          2.89199       -2.13860        0.00000
H          3.34758        0.15060        0.00000
H          1.84454        1.94937        0.00000
H          0.83634       -3.24494        0.00000
H         -1.32395       -2.33431        0.00000
C         -0.84689        2.05854        0.00000
H         -0.51223        3.09023        0.00000
C         -2.18168        1.61340        0.00000
H         -3.05473        2.25901        0.00000
C         -2.21626        0.20676        0.00000
H         -3.10473       -0.41538        0.00000
20
pi_004.log       Energy: -289992.0067112
C          0.00000        2.43285        1.10464
C          0.00000        1.28363        1.88326
C          0.00000       -0.00000        1.25747
C          0.00000        0.00000       -0.14121
C          0.00000        1.16248       -0.95295
C          0.00000        2.39405       -0.32276
H         -0.00000       -1.35956        2.96834
H          0.00000        3.40313        1.59525
H          0.00000        1.35956        2.96834
C         -0.00000       -1.28363        1.88326
C         -0.00000       -1.16248       -0.95295
H          0.00000        3.32617       -0.88346
C         -0.00000       -2.39405       -0.32276
C         -0.00000       -2.43285        1.10464
H         -0.00000       -3.32617       -0.88346
H         -0.00000       -3.40313        1.59525
C          0.00000        0.68390       -2.34646
H          0.00000        1.32266       -3.22336
C         -0.00000       -0.68390       -2.34646
H         -0.00000       -1.32266       -3.22336
24
pi_005.log       Energy: -338598.2064563
C          0.00000        3.56666       -0.29621
C          0.00000        2.84080        0.88137
C          0.00000        1.42403        0.86693
C          0.00000        0.72972       -0.38191
C          0.00000        1.50179       -1.57010
C          0.00000        2.88701       -1.53150
C          0.00000        0.68110        2.09709
C         -0.00000       -0.72972       -0.38191
C         -0.00000       -1.42403        0.86693
C         -0.00000       -0.68110        2.09709
C         -0.00000       -2.84080        0.88137
H         -0.00000       -3.35257        1.84148
C         -0.00000       -3.56666       -0.29621
C         -0.00000       -2.88701       -1.53150
C         -0.00000       -1.50179       -1.57010
H          0.00000        1.23325        3.03442
H          0.00000        4.65353       -0.27125
H          0.00000        3.35257        1.84148
H          0.00000        1.01117       -2.53776
H          0.00000        3.45127       -2.46087
H         -0.00000       -1.23325        3.03442
H         -0.00000       -4.65353       -0.27125
H         -0.00000       -3.45127       -2.46087
H         -0.00000       -1.01117       -2.53776
24
pi_006.log       Energy: -338592.8976434
C          0.00000        3.66503        0.71433
C          0.00000        2.48156        1.41000
C          0.00000        1.22464        0.72347
C          0.00000        1.22464       -0.72347
C          0.00000        2.48156       -1.41000
C          0.00000        3.66503       -0.71433
C          0.00000       -0.00000        1.40687
C          0.00000       -0.00000       -1.40687
C         -0.00000       -1.22464       -0.72347
C         -0.00000       -1.22464        0.72347
C         -0.00000       -2.48156        1.41000
H          0.00000       -2.47956        2.49811
C         -0.00000       -3.66503        0.71433
C         -0.00000       -3.66503       -0.71433
C         -0.00000       -2.48156       -1.41000
H          0.00000       -0.00000        2.49566
H          0.00000        4.61213        1.24824
H          0.00000        2.47956        2.49811
H          0.00000        2.47956       -2.49811
H          0.00000        4.61213       -1.24824
H          0.00000       -0.00000       -2.49566
H         -0.00000       -4.61213        1.24824
H         -0.00000       -4.61213       -1.24824
H          0.00000       -2.47956       -2.49811
26
pi_007.log       Energy: -386439.4902247
C          0.00000        0.00000        3.52803
C          0.00000        1.21306        2.83621
C          0.00000        1.23767        1.43016
C          0.00000        0.00000        0.71400
C         -0.00000       -1.23767        1.43016
C         -0.00000       -1.21306        2.83621
C          0.00000        2.46762        0.68209
C          0.00000        0.00000       -0.71400
C          0.00000        1.23767       -1.43016
C          0.00000        2.46762       -0.68209
C          0.00000        1.21306       -2.83621
H          0.00000        2.15320       -3.38342
C          0.00000       -0.00000       -3.52803
C         -0.00000       -1.21306       -2.83621
C         -0.00000       -1.23767       -1.43016
C         -0.00000       -2.46762       -0.68209
C         -0.00000       -2.46762        0.68209
H          0.00000       -3.40619        1.23216
H          0.00000       -3.40619       -1.23216
H          0.00000        3.40619        1.23216
H          0.00000        0.00000        4.61531
H         -0.00000        2.15320        3.38342
H         -0.00000       -2.15320        3.38342
H          0.00000        3.40619       -1.23216
H          0.00000        0.00000       -4.61531
H         -0.00000       -2.15320       -3.38342
30
pi_008.log       Energy: -435020.0306671
C          2.48826        0.70169       -0.00000
C          1.26116        1.43463       -0.00000
C         -0.00001        0.70981       -0.00000
C          0.00000       -0.70979        0.00000
C          1.25614       -1.39952       -0.00000
C          2.44499       -0.72619       -0.00000
C         -1.25616        1.39953        0.00000
C         -1.26117       -1.43463        0.00000
C         -2.48827       -0.70169        0.00000
```

C	-2.44501	0.72620	0.00000
C	-3.72646	-1.39396	0.00000
H	-4.64713	-0.81415	0.00000
C	-3.76847	-2.77459	0.00000
C	-2.56102	-3.50672	0.00000
C	-1.34138	-2.85267	0.00000
H	-1.27658	2.48346	0.00000
H	1.27654	-2.48346	-0.00000
H	3.38347	-1.27634	-0.00000
H	-3.38350	1.27633	0.00000
H	-4.72220	-3.29625	0.00000
H	-2.58735	-4.59369	0.00000
H	-0.43706	-3.45077	0.00000
C	1.34140	2.85269	-0.00000
H	0.43711	3.45081	-0.00000
C	3.72646	1.39394	-0.00000
H	4.64712	0.81412	-0.00000
C	2.56105	3.50672	-0.00000
H	2.58739	4.59368	-0.00000
C	3.76848	2.77457	-0.00000
H	4.72222	3.29620	-0.00000

30

pi_009.log Energy: -435009.3977570

C	-0.00001	-4.89381	0.71657
C	-0.00001	-3.71373	1.41228
C	-0.00001	-2.45183	0.72677
C	-0.00001	-2.45183	-0.72677
C	-0.00001	-3.71373	-1.41228
C	-0.00001	-4.89381	-0.71657
C	-0.00001	-1.23596	1.40981
C	-0.00001	-1.23596	-1.40981
C	-0.00000	-0.00000	-0.72690
C	-0.00000	-0.00000	0.72690
C	-0.00000	1.23597	1.40981
H	0.00000	1.23585	2.49848
C	-0.00001	2.45182	0.72676
C	-0.00001	2.45182	-0.72676
C	-0.00000	1.23597	-1.40981
H	-0.00001	-1.23582	2.49848
H	-0.00001	-5.84180	1.24882
H	-0.00001	-3.71204	2.50037
H	-0.00001	-3.71204	-2.50037
H	-0.00001	-5.84180	-1.24882
H	-0.00001	-1.23582	-2.49848
H	0.00000	1.23585	-2.49848
C	0.00000	3.71373	-1.41228
H	-0.00002	3.71206	-2.50037
C	0.00000	3.71373	1.41228
H	-0.00002	3.71206	2.50037
C	0.00003	4.89380	-0.71657
H	0.00003	5.84178	-1.24885
C	0.00003	4.89380	0.71657
H	0.00003	5.84178	1.24885

30

pi_010.log Energy: -435017.9095288

C	-4.37144	-1.38069	-0.00023
C	-3.03913	-1.72334	-0.00019
C	-2.02533	-0.71718	-0.00011
C	-2.42301	0.66603	-0.00007
C	-3.81517	0.98503	-0.00011
C	-4.76403	-0.01077	-0.00019
C	-0.65324	-1.04000	-0.00007
C	-1.41957	1.65158	0.00001
C	-0.06281	1.32318	0.00005
C	0.34315	-0.06334	0.00000
C	1.77568	-0.38092	0.00005
C	2.72309	0.68546	0.00013
C	2.26623	2.05442	0.00017
C	0.94216	2.35751	0.00013
H	-0.39146	-2.09355	-0.00011
H	-5.13401	-2.15566	-0.00029
H	-2.73779	-2.76883	-0.00023
H	-4.11116	2.03201	-0.00008
H	-5.82132	0.24259	-0.00022
H	-1.70929	2.70094	0.00004
H	3.01298	2.84546	0.00023
H	0.61204	3.39402	0.00016
C	4.10646	0.39376	0.00017
H	4.81405	1.22037	0.00019
C	2.26925	-1.70557	0.00002
H	1.58049	-2.54397	-0.00003
C	4.56191	-0.91524	0.00017
H	5.62856	-1.12508	0.00019
C	3.63211	-1.97107	0.00010
H	3.97898	-3.00143	0.00009

30

pi_011.log Energy: -435014.0018134

C	2.40899	1.89378	0.35239
C	1.21885	2.54169	0.19517
C	0.00001	1.81577	0.00002
C	0.00001	0.39623	0.00002
C	1.28958	-0.27996	-0.05773
C	2.48488	0.47495	0.19048
C	-1.21883	2.54170	-0.19514
C	-1.28958	-0.27995	0.05775
C	-2.48487	0.47496	-0.19052
C	-2.40896	1.89380	-0.35240
C	-3.74563	-0.17531	-0.19658
H	-4.63202	0.41577	-0.41736
C	-3.85981	-1.51744	0.10910
C	-2.70452	-2.24090	0.47217
C	-1.45908	-1.63718	0.44924
H	-1.17076	3.62751	-0.23883
H	3.32468	2.44909	0.54300
H	1.17080	3.62750	0.23888
H	-3.32464	2.44911	-0.54303
H	-4.83312	-2.00153	0.11116
H	-2.79085	-3.27656	0.79183
H	-0.60646	-2.20793	0.79354
C	3.74563	-0.17534	0.19654
H	4.63204	0.41573	0.41734
C	1.45905	-1.63718	-0.44922
H	0.60641	-2.20790	-0.79351
C	2.70448	-2.24092	-0.47217
H	2.79079	-3.27656	-0.79187
C	3.85979	-1.51747	-0.10911
H	4.83308	-2.00159	-0.11110

30

pi_012.log Energy: -435019.5366703

C	2.43313	-2.88449	0.00000
C	2.43829	-1.49888	0.00000
C	1.23925	-0.74614	0.00000
C	0.00000	-1.44525	0.00000
C	0.02291	-2.86053	0.00000
C	1.21074	-3.57378	0.00000
C	1.25325	0.72243	0.00000
C	-1.26491	-0.69929	0.00000
C	-1.25137	0.72328	0.00000
C	0.02752	1.44501	0.00000
C	-2.48910	1.41054	0.00000
H	-2.50979	2.49422	0.00000
C	-3.69999	0.73693	0.00000
C	-3.71335	-0.66645	0.00000
C	-2.51548	-1.36291	0.00000
H	3.37337	-3.43011	0.00000
H	3.39759	-0.99509	0.00000
H	-0.90608	-3.41816	0.00000
H	1.19071	-4.66071	0.00000
H	-4.63170	1.29698	0.00000
H	-4.65550	-1.20875	0.00000
H	-2.55650	-2.44599	0.00000
C	0.07738	2.85953	0.00000
H	-0.84078	3.43480	0.00000
C	2.46645	1.45223	0.00000
H	3.41606	0.93034	0.00000
C	1.27856	3.55007	0.00000
H	1.27910	4.63719	0.00000
C	2.48764	2.83769	0.00000
H	3.43808	3.36532	0.00000

36

pi_014.log Energy: -578551.8592623

C	1.24929	3.53861	0.00000
C	0.00000	2.85124	0.00000
C	0.00000	1.42855	0.00000
C	1.23720	0.71426	0.00000
C	2.46935	1.42557	0.00000
C	2.43992	2.85116	0.00000
C	-1.23720	0.71426	0.00000
C	1.23720	-0.71426	0.00000
C	-0.00000	-1.42855	0.00000
C	-1.23720	-0.71426	0.00000
C	-0.00000	-2.85124	0.00000
C	1.24929	-3.53861	0.00000
C	2.43992	-2.85116	0.00000
C	2.46935	-1.42557	0.00000
C	3.68941	-0.68741	0.00000
C	3.68941	0.68741	0.00000
H	4.62978	1.23425	0.00000
H	4.62978	-1.23425	0.00000
H	1.24639	4.62650	0.00000
H	3.38333	3.39271	0.00000
H	1.24639	-4.62650	0.00000
H	3.38333	-3.39271	0.00000
C	-1.24929	3.53861	0.00000

```
C        -2.43992     2.85116     0.00000
C        -2.46935     1.42557     0.00000
H        -1.24639     4.62650     0.00000
H        -3.38333     3.39271     0.00000
C        -3.68941     0.68741     0.00000
C        -3.68941    -0.68741     0.00000
C        -2.46935    -1.42557     0.00000
H        -4.62978     1.23425     0.00000
H        -4.62978    -1.23425     0.00000
C        -1.24929    -3.53861     0.00000
H        -1.24639    -4.62650     0.00000
C        -2.43992    -2.85116     0.00000
H        -3.38333    -3.39271     0.00000
32
pi_015.log       Energy: -482854.6785416
C        -3.58024    -1.23342     0.03560
C        -2.87750     0.00000    -0.00000
C        -1.44033    -0.00000    -0.00000
C        -0.73885    -1.25124     0.01588
C        -1.48011    -2.43046     0.05871
C        -2.88878    -2.42491     0.06939
C        -0.73885     1.25124    -0.01588
C         0.73885    -1.25124    -0.01588
C         1.44033    -0.00000     0.00000
C         0.73885     1.25124     0.01588
C         2.87750    -0.00000     0.00000
C         3.58024    -1.23342    -0.03560
C         2.88878    -2.42491    -0.06939
C         1.48011    -2.43046    -0.05871
H        -4.66777    -1.21964     0.03782
H        -0.98005    -3.39213     0.08632
H        -3.42409    -3.37052     0.10253
H         4.66777    -1.21964    -0.03782
H         3.42409    -3.37052    -0.10253
H         0.98005    -3.39213    -0.08632
C         3.58024     1.23342     0.03560
H         4.66777     1.21964     0.03782
C         1.48011     2.43046     0.05871
H         0.98005     3.39213     0.08632
C        -3.58024     1.23342    -0.03560
H        -4.66777     1.21964    -0.03783
C        -2.88878     2.42491    -0.06939
H        -3.42408     3.37052    -0.10253
C         2.88878     2.42491     0.06939
H         3.42408     3.37052     0.10253
C        -1.48011     2.43046    -0.05871
H        -0.98005     3.39213    -0.08632
26
pi_016.log       Energy: -386425.1889676
C        -0.00000     1.41794    -2.50028
C        -0.00000     0.71457    -1.29775
C        -0.00000    -0.71457    -1.29775
C        -0.00000    -1.41794    -2.50028
C         0.00000    -0.70070    -3.70600
C         0.00000     0.70070    -3.70600
H        -0.00000     2.50546    -2.50747
H        -0.00000    -2.50546    -2.50747
H         0.00000    -1.23885    -4.65069
H         0.00000     1.23885    -4.65069
C        -0.00000     1.17273     0.10528
C        -0.00000     0.00000     0.90420
C        -0.00000     2.40064     0.73928
C        -0.00000    -1.17273     0.10528
C        -0.00000     0.00000     2.30888
C        -0.00000     2.43341     2.16477
H        -0.00000     3.33368     0.18090
C        -0.00000    -2.40064     0.73928
C         0.00000    -1.28039     2.93721
C         0.00000     1.28039     2.93721
H        -0.00000     3.40122     2.66027
C        -0.00000    -2.43341     2.16477
H        -0.00000    -3.33368     0.18090
H         0.00000    -1.35059     4.02264
H         0.00000     1.35059     4.02264
H        -0.00000    -3.40122     2.66027
22
pi_017.log       Energy: -290758.1177603
C         2.86323     1.13804     0.40476
C         1.46637     1.13737     0.40532
C         0.74357    -0.00000     0.00000
C         1.46638    -1.13738    -0.40532
C         2.86324    -1.13804    -0.40476
C         3.56871     0.00000     0.00000
H         3.40050     2.02611     0.72937
H         0.93066     2.02056     0.74397
H         0.93067    -2.02056    -0.74398
H         3.40051    -2.02610    -0.72937
H         4.65599     0.00000    -0.00000
C        -0.74357    -0.00000    -0.00000
C        -1.46638    -1.13738     0.40532
C        -1.46637     1.13737    -0.40532
C        -2.86324    -1.13804     0.40476
H        -0.93067    -2.02056     0.74398
C        -2.86323     1.13804    -0.40476
H        -0.93066     2.02056    -0.74397
C        -3.56871     0.00000    -0.00000
H        -3.40051    -2.02610     0.72937
H        -3.40050     2.02611    -0.72937
H        -4.65599     0.00000    -0.00000
23
pi_018.log       Energy: -314678.3783976
H        -0.00003     2.49122    -0.88085
C         3.01802    -1.21473    -0.00015
C         3.46391     0.11518    -0.00014
C         2.54584     1.17539    -0.00009
C         1.18341     0.88872    -0.00003
C         0.73461    -0.45026    -0.00004
C         1.64993    -1.50765    -0.00010
H         3.74272    -2.02550    -0.00019
H         4.53071     0.32576    -0.00018
H         2.89673     2.20534    -0.00008
H         1.30708    -2.53967    -0.00010
C         0.00000     1.83434     0.00003
H         0.00003     2.49119     0.88092
C        -1.18341     0.88872     0.00006
C        -2.54584     1.17539     0.00012
C        -3.46391     0.11518     0.00014
C        -3.01802    -1.21473     0.00011
C        -1.64993    -1.50765     0.00005
C        -0.73461    -0.45026     0.00003
H        -2.89673     2.20534     0.00014
H        -4.53071     0.32576     0.00019
H        -3.74272    -2.02550     0.00013
H        -1.30708    -2.53967     0.00003
21
pi_019.log       Energy: -337208.3312223
C         3.06024    -1.11145    -0.00016
C         3.40395     0.25382    -0.00017
C         2.42281     1.25107    -0.00010
C         1.10173     0.82269    -0.00002
C         0.72609    -0.53516    -0.00002
C         1.72419    -1.51813    -0.00009
H         3.84883    -1.85937    -0.00022
H         4.45200     0.54158    -0.00023
H         2.67508     2.30713    -0.00009
H         1.46412    -2.57335    -0.00009
C        -1.10173     0.82269     0.00006
C        -2.42281     1.25107     0.00009
C        -3.40395     0.25382     0.00011
C        -3.06024    -1.11145     0.00010
C        -1.72419    -1.51813     0.00008
C        -0.72609    -0.53516     0.00005
H        -2.67508     2.30713     0.00011
H        -4.45200     0.54158     0.00013
H        -3.84883    -1.85937     0.00012
H        -1.46412    -2.57335     0.00006
O         0.00000     1.65175     0.00009
21
pi_020.log       Energy: -539880.6475206
C         2.98431    -1.44837    -0.00014
C         3.49778    -0.13901    -0.00014
C         2.64174     0.96260    -0.00009
C         1.26192     0.73381    -0.00004
C         0.72761    -0.57598    -0.00003
C         1.60902    -1.66947    -0.00009
H         3.66666    -2.29438    -0.00018
H         4.57303     0.02028    -0.00017
H         3.04007     1.97361    -0.00010
H         1.21561    -2.68282    -0.00010
C        -1.26192     0.73381     0.00005
C        -2.64174     0.96260     0.00010
C        -3.49778    -0.13901     0.00014
C        -2.98431    -1.44836     0.00012
C        -1.60902    -1.66947     0.00007
C        -0.72761    -0.57598     0.00003
H        -3.04007     1.97361     0.00012
H        -4.57303     0.02028     0.00017
H        -3.66666    -2.29438     0.00015
H        -1.21561    -2.68282     0.00007
S         0.00000     1.97523     0.00001
25
pi_021.log       Energy: -349420.3722171
C         3.04185    -1.43764     0.00999
C         3.42748    -0.08218     0.01264
```

```
C          2.48205        0.94519        0.00117
C          1.12812        0.58546       -0.01738
C          0.72349       -0.77930       -0.01044
C          1.69213       -1.79121       -0.00032
H          3.80432       -2.21233        0.01991
H          4.48459        0.17219        0.02705
H          2.79317        1.98562        0.01103
H          1.39358       -2.83687        0.00277
C         -1.12812        0.58546       -0.01737
C         -2.48205        0.94519        0.00117
C         -3.42748       -0.08218        0.01264
C         -3.04185       -1.43765        0.00999
C         -1.69213       -1.79121       -0.00033
C         -0.72349       -0.77930       -0.01045
H         -2.79317        1.98562        0.01104
H         -4.48458        0.17219        0.02706
H         -3.80431       -2.21233        0.01991
H         -1.39359       -2.83688        0.00275
C         -0.00000        2.84396        0.02396
H         -0.00005        3.19832        1.06332
H          0.88379        3.23603       -0.48596
H         -0.88376        3.23603       -0.48604
N         -0.00000        1.39499       -0.04349
22
pi_022.log       Energy: -337794.3373028
C         -0.00000        2.37518        0.72628
C         -0.00000        1.18208        1.44245
C          0.00000       -0.00000        0.67800
C          0.00000        0.00000       -0.67800
C         -0.00000        1.18208       -1.44245
C         -0.00000        2.37517       -0.72628
H         -0.00000        3.33928        1.22985
C         -0.00000       -1.18208        1.44245
C         -0.00000       -1.18208       -1.44245
H         -0.00000        3.33928       -1.22985
C         -0.00000       -2.37517       -0.72628
C         -0.00000       -2.37518        0.72628
H         -0.00000       -3.33928       -1.22985
H         -0.00000       -3.33928        1.22985
C         -0.00000        0.68495       -2.84989
H         -0.00000        1.30988       -3.73668
C         -0.00000       -0.68495       -2.84989
H         -0.00000       -1.30988       -3.73668
C         -0.00000        0.68495        2.84990
C         -0.00000       -0.68495        2.84990
H         -0.00000        1.30989        3.73668
H         -0.00000       -1.30989        3.73668
28
pi_023.log       Energy: -434230.4971475
C         -0.00000        2.37740        1.78613
C         -0.00000        1.18031        2.49992
C          0.00000        0.00000        1.73281
C          0.00000        0.00000        0.37301
C         -0.00000        1.19153       -0.38071
C         -0.00000        2.38286        0.33974
H         -0.00000        3.33897        2.29476
C         -0.00000       -1.18031        2.49992
C         -0.00000       -1.19153       -0.38071
H         -0.00000        3.34691       -0.16315
C         -0.00000       -2.38286        0.33974
C         -0.00000       -2.37740        1.78613
H         -0.00000       -3.34691       -0.16315
H         -0.00000       -3.33897        2.29476
C         -0.00000        0.72083       -1.79335
C         -0.00000       -0.72083       -1.79335
C         -0.00000        0.68656        3.90145
C         -0.00000       -0.68656        3.90145
H         -0.00000        1.31136        4.78839
H         -0.00000       -1.31136        4.78839
C          0.00000       -1.41688       -2.99507
C          0.00000       -0.69813       -4.20548
H          0.00000       -2.50426       -3.00445
H          0.00000       -1.23859       -5.14873
C          0.00000        1.41688       -2.99507
C          0.00000        0.69813       -4.20548
H          0.00000        2.50426       -3.00445
H          0.00000        1.23859       -5.14873
34
pi_024.log       Energy: -530665.5462245
C         -0.00000        2.38453        0.72115
C         -0.00000        1.18994        1.43769
C          0.00000        0.00000        0.68224
C          0.00000        0.00000       -0.68224
C         -0.00000        1.18994       -1.43769
C         -0.00000        2.38453       -0.72115
H         -0.00000        3.34610        1.22896
C          0.00000       -1.18994        1.43769
C          0.00000       -1.18994       -1.43769
H         -0.00000        3.34610       -1.22896
C          0.00000       -2.38453       -0.72115
C          0.00000       -2.38453        0.72115
H          0.00000       -3.34610       -1.22896
H          0.00000       -3.34610        1.22896
C         -0.00000        0.72086       -2.84710
C          0.00000       -0.72086       -2.84710
C         -0.00000        0.72086        2.84710
C          0.00000       -0.72086        2.84710
C          0.00000       -1.41743       -4.05057
C          0.00000       -0.69907       -5.25892
H          0.00000       -2.50484       -4.05985
H          0.00000       -1.23866       -6.20270
C         -0.00000        1.41743       -4.05057
C         -0.00000        0.69907       -5.25892
H         -0.00000        2.50484       -4.05985
H         -0.00000        1.23866       -6.20270
C         -0.00000        1.41743        4.05057
C         -0.00000        0.69907        5.25892
H         -0.00000        2.50483        4.05986
H         -0.00000        1.23865        6.20270
C          0.00000       -1.41743        4.05057
C          0.00000       -0.69907        5.25892
H          0.00000       -2.50483        4.05986
H          0.00000       -1.23865        6.20270
14
pi_025.log       Energy: -193523.6021356
C         -1.16450        1.28280        0.00000
C          0.03676        2.17519        0.00000
C          1.16450        1.41483        0.00000
C          0.72941        0.00490        0.00000
C         -0.72941       -0.00490       -0.00000
C          1.16450       -1.28280        0.00000
C         -0.03676       -2.17519       -0.00000
C         -1.16450       -1.41483       -0.00000
H         -2.18910        1.64265        0.00000
H         -0.01329        3.25871        0.00000
H          2.18842        1.77089        0.00000
H          2.18910       -1.64265        0.00000
H          0.01329       -3.25871        0.00000
H         -2.18842       -1.77089       -0.00000
20
pi_026.log       Energy: -289961.6276036
C         -0.84262        1.50144        0.00000
C         -1.20302        0.06429       -0.00000
C         -0.00000       -0.71100       -0.00000
C          1.10631        0.25703        0.00000
C          0.51369        1.60125        0.00000
C          2.45962        0.39023        0.00000
C          2.76896        1.84410        0.00000
C          1.61250        2.56910        0.00000
H         -1.56946        2.30849       -0.00000
H          3.20987       -0.39401        0.00000
H          3.77650        2.24726        0.00000
H          1.51977        3.64881        0.00000
C         -0.06331       -2.09460       -0.00000
C         -1.33152       -2.71913       -0.00000
H          0.84237       -2.69620       -0.00000
H         -1.38980       -3.80482       -0.00000
C         -2.44491       -0.55266       -0.00000
C         -2.50248       -1.96388       -0.00000
H         -3.35992        0.03530       -0.00000
H         -3.46865       -2.46187       -0.00000
26
pi_027.log       Energy: -386387.1415738
C          1.80938        1.70483       -0.00000
C          0.49141        1.04855       -0.00001
C          0.69320       -0.39236        0.00000
C          2.14291       -0.59118        0.00000
C          2.77198        0.74047       -0.00000
C          3.13364       -1.52694        0.00000
C          4.42551       -0.80608        0.00000
C          4.21647        0.54689        0.00000
H          1.94977        2.78166       -0.00000
H          3.03423       -2.60737        0.00000
H          5.39230       -1.29963        0.00000
H          4.97239        1.32331        0.00000
C         -0.38014       -1.23978        0.00001
C         -1.71448       -0.70529        0.00000
H         -0.24638       -2.31940        0.00001
C         -0.76805        1.58588       -0.00001
C         -1.91117        0.71718       -0.00000
H         -0.92172        2.66312       -0.00001
C         -2.85028       -1.54962        0.00000
C         -4.13267       -1.02210        0.00000
H         -2.70185       -2.62745        0.00001
```

H -4.99300 -1.68684 0.00000
C -3.23148 1.22740 -0.00000
C -4.32551 0.37596 -0.00000
H -3.37702 2.30570 -0.00000
H -5.33306 0.78409 -0.00000
24
pi_028.log Energy: -338549.0926688
C 0.00000 0.00000 0.60878
C 0.00000 0.00000 -0.60878
C 0.00000 0.00000 2.03971
C 0.00000 1.21560 2.75456
C -0.00000 -1.21560 2.75456
C 0.00000 1.21074 4.14980
H 0.00000 2.15482 2.20838
C -0.00000 -1.21074 4.14980
H -0.00000 -2.15482 2.20838
C 0.00000 0.00000 4.85167
H 0.00000 2.15408 4.69018
H -0.00000 -2.15408 4.69018
H 0.00000 0.00000 5.93882
C 0.00000 0.00000 -2.03971
C 1.21560 -0.00000 -2.75456
C -1.21560 0.00000 -2.75456
C 1.21074 -0.00000 -4.14980
H 2.15482 -0.00000 -2.20838
C -1.21074 0.00000 -4.14980
H -2.15482 0.00000 -2.20838
C 0.00000 0.00000 -4.85167
H 2.15408 -0.00000 -4.69018
H -2.15408 0.00000 -4.69018
H 0.00000 0.00000 -5.93882
44
pi_033.log Energy: -722799.5426583
C -2.81950 2.46298 0.00000
C -3.55666 1.24180 0.00000
C -2.84088 -0.00001 -0.00000
C -1.41936 -0.00000 -0.00000
C -0.70524 1.23483 0.00000
C -1.43694 2.48164 0.00000
C -3.55663 -1.24175 -0.00000
C -0.70527 -1.23488 -0.00000
C -1.43694 -2.48166 -0.00000
C -2.81950 -2.46300 -0.00000
C -0.67927 -3.71534 -0.00000
C 0.67922 -3.71530 -0.00000
C 1.43691 -2.48157 -0.00000
C 0.70517 -1.23483 -0.00000
C 1.41933 -0.00004 -0.00000
C 0.70522 1.23491 0.00000
H -3.36836 3.40275 0.00000
H 1.22960 -4.65356 -0.00000
C -4.96723 1.21389 0.00000
C -5.65646 0.00004 0.00000
C -4.96721 -1.21380 -0.00000
H -5.51510 2.15337 0.00000
H -6.74373 -0.00008 -0.00000
C 2.81949 -2.46298 -0.00000
C 3.55670 -1.24175 -0.00000
H 3.36840 -3.40271 -0.00000
C 2.84086 -0.00002 -0.00000
C 3.55665 1.24179 0.00000
C 2.81954 2.46299 0.00000
C 1.43695 2.48166 0.00000
H 3.36859 3.40273 0.00000
C 0.67930 3.71534 0.00000
C -0.67925 3.71528 0.00000
H 1.22971 4.65366 0.00000
H -1.22952 4.65362 0.00000
H -5.51523 -2.15326 -0.00000
H -3.36865 -3.40268 -0.00000
H -1.22980 -4.65361 -0.00000
C 4.96724 -1.21393 -0.00000
C 5.65651 -0.00006 -0.00000
H 5.51508 -2.15342 -0.00000
H 6.74378 0.00010 0.00000
C 4.96725 1.21375 0.00000
H 5.51531 2.15320 0.00000
36
pi_034.log Energy: -531425.1387987
C 0.00000 0.72858 3.68044
C 0.00000 1.41135 2.46875
C 0.00000 0.72930 1.22702
C 0.00000 -0.72930 1.22702
C 0.00000 -1.41135 2.46875
C 0.00000 -0.72858 3.68044
C -0.00000 1.41194 0.00004
C 0.00000 -1.41194 0.00004
C -0.00000 -0.72930 -1.22706
C -0.00000 0.72930 -1.22706
C -0.00000 1.41135 -2.46872
H -0.00000 2.50000 -2.46884
C -0.00000 0.72849 -3.68047
C 0.00000 -0.72849 -3.68047
C 0.00000 -1.41135 -2.46872
H -0.00000 2.50049 0.00003
H 0.00000 2.50001 2.46882
H 0.00000 -2.50001 2.46882
H 0.00000 -2.50049 0.00003
H 0.00000 -2.50000 -2.46884
C -0.00000 -1.41342 -4.94481
H -0.00000 -2.50150 -4.94338
C -0.00000 1.41342 -4.94481
H -0.00000 2.50150 -4.94338
C -0.00000 -0.71770 -6.12325
H -0.00000 -1.24912 -7.07169
C -0.00000 0.71770 -6.12325
H -0.00000 1.24912 -7.07169
C 0.00000 1.41346 4.94481
C 0.00000 0.71774 6.12324
H 0.00000 2.50155 4.94346
H 0.00000 1.24927 7.07162
C 0.00000 -0.71774 6.12324
C 0.00000 -1.41346 4.94481
H 0.00000 -1.24927 7.07162
H 0.00000 -2.50155 4.94346
42
pi_035.log Energy: -627840.5426695
C -0.00000 0.73075 2.45549
C -0.00000 1.41316 1.23288
C -0.00000 0.73127 -0.00003
C -0.00000 -0.73127 -0.00003
C -0.00000 -1.41316 1.23288
C -0.00000 -0.73075 2.45549
C -0.00000 1.41316 -1.23284
C -0.00000 -1.41316 -1.23284
C 0.00000 -0.73066 -2.45554
C 0.00000 0.73066 -2.45554
C -0.00000 1.41220 -3.70014
H -0.00000 2.50083 -3.70048
C -0.00000 0.72944 -4.90978
C -0.00000 -0.72944 -4.90978
C -0.00000 -1.41220 -3.70014
H -0.00000 2.50168 -1.23304
H -0.00000 2.50169 1.23311
H -0.00000 -2.50169 1.23311
H -0.00000 -2.50168 -1.23304
H -0.00000 -2.50083 -3.70048
C 0.00000 -1.41404 -6.17536
H 0.00000 -2.50211 -6.17405
C 0.00000 1.41404 -6.17536
H 0.00000 2.50211 -6.17405
C 0.00000 -0.71831 -7.35294
H 0.00000 -1.24928 -8.30161
C 0.00000 0.71831 -7.35294
H 0.00000 1.24928 -8.30161
C -0.00000 1.41227 3.70009
C -0.00000 0.72947 4.90972
H -0.00000 2.50091 3.70049
C -0.00000 -0.72947 4.90972
C -0.00000 -1.41227 3.70009
H -0.00000 -2.50091 3.70049
C -0.00000 1.41415 6.17541
C -0.00000 0.71837 7.35300
H -0.00000 2.50221 6.17425
H -0.00000 1.24943 8.30162
C -0.00000 -0.71837 7.35300
C -0.00000 -1.41415 6.17541
H -0.00000 -1.24943 8.30162
H -0.00000 -2.50221 6.17425
32
pi_036.log Energy: -482861.7911293
C -0.00000 3.53287 -0.92153
C -0.00000 2.85077 0.29880
C -0.00000 1.44810 0.35727
C -0.00000 0.71881 -0.86943
C -0.00000 1.42347 -2.11570
C -0.00000 2.83024 -2.11994
C -0.00000 0.71170 1.62859
C -0.00000 -0.71881 -0.86943
C -0.00000 -1.44810 0.35727
C -0.00000 -0.71170 1.62859
C -0.00000 -2.85077 0.29880
H 0.00000 -3.43768 1.20987
C -0.00000 -3.53287 -0.92153

```
C         -0.00000       -2.83024       -2.11994
C         -0.00000       -1.42347       -2.11570
C          0.00000       -0.68054       -3.34629
C          0.00000        0.68054       -3.34629
H         -0.00000        1.23423       -4.28258
H         -0.00000       -1.23423       -4.28258
H         -0.00000        4.62003       -0.92632
H          0.00000        3.43768        1.20987
H         -0.00000        3.35899       -3.07034
H         -0.00000       -4.62003       -0.92632
H         -0.00000       -3.35899       -3.07034
C         -0.00000        1.38761        2.87164
C         -0.00000        0.70187        4.07600
H         -0.00000        2.47147        2.89974
H         -0.00000        1.25254        5.01327
C         -0.00000       -0.70187        4.07600
C         -0.00000       -1.38761        2.87164
H         -0.00000       -1.25254        5.01327
H         -0.00000       -2.47147        2.89974
44
pi_038.log       Energy: -722804.6561263
C         -0.00105        3.54960       -0.29978
C          0.00499        2.86981       -1.54157
C          0.00054        1.43285       -1.56320
C          0.00300        0.72045       -0.32880
C         -0.00561        1.42388        0.89942
C         -0.00499        2.86468        0.90724
C          0.00662        0.73674       -2.81670
C         -0.00300       -0.72045       -0.32880
C         -0.00054       -1.43285       -1.56320
C         -0.00662       -0.73674       -2.81670
C         -0.00499       -2.86981       -1.54157
C          0.00105       -3.54960       -0.29978
C          0.00499       -2.86468        0.90724
C          0.00561       -1.42388        0.89942
C         -0.00000       -0.71258        2.13855
C          0.00000        0.71258        2.13855
H         -0.00003        4.63788       -0.29862
H          0.00003       -4.63788       -0.29862
C          0.01533        3.58152       -2.76847
C          0.02617        2.89761       -3.96837
C          0.02252        1.49081       -3.99230
H          0.01734        4.66890       -2.74817
H          0.03803        3.44411       -4.90808
C         -0.02252       -1.49081       -3.99230
C         -0.01533       -3.58152       -2.76847
H         -0.01734       -4.66890       -2.74817
C         -0.02617       -2.89761       -3.96837
H         -0.03803       -3.44411       -4.90808
C          0.00948       -3.54978        2.17645
C          0.00933       -2.86180        3.35173
H          0.01211       -4.63755        2.17330
H          0.01229       -3.39373        4.30058
C          0.00482       -1.42186        3.37554
C          0.00252       -0.69392        4.58275
H          0.00473       -1.23700        5.52524
C         -0.00252        0.69392        4.58275
C         -0.00482        1.42186        3.37554
H         -0.00473        1.23700        5.52524
C         -0.00933        2.86180        3.35173
C         -0.00948        3.54978        2.17645
H         -0.01229        3.39373        4.30058
H         -0.01211        4.63755        2.17330
H          0.03488        0.99969       -4.95912
H         -0.03488       -0.99969       -4.95912
46
pi_039.log       Energy: -770656.0286361
C          0.00212        3.54367       -0.00000
C          0.00147        2.86031       -1.22515
C          0.00298        1.42717       -1.22879
C         -0.00374        0.71782       -0.00000
C          0.00298        1.42717        1.22879
C          0.00147        2.86031        1.22515
C         -0.00147        0.71378       -2.46609
C          0.00374       -0.71782       -0.00000
C         -0.00298       -1.42717       -1.22879
C          0.00147       -0.71378       -2.46609
C         -0.00147       -2.86031       -1.22515
C         -0.00212       -3.54367       -0.00000
C         -0.00147       -2.86031        1.22515
C         -0.00298       -1.42717        1.22879
C          0.00147       -0.71378        2.46609
C         -0.00147        0.71378        2.46609
H          0.00214        4.63210        0.00000
H         -0.00214       -4.63210        0.00000
C          0.00216        3.54638       -2.48735
C          0.00173        2.85889       -3.66942
C          0.00089        1.42434       -3.69978
H          0.00273        4.63419       -2.48501
H          0.00217        3.39675       -4.61497
C          0.00043        0.69139       -4.91213
C         -0.00043       -0.69139       -4.91213
C         -0.00089       -1.42434       -3.69978
H          0.00091        1.23541       -5.85410
H         -0.00091       -1.23541       -5.85410
C         -0.00216       -3.54638       -2.48735
H         -0.00273       -4.63419       -2.48501
C         -0.00173       -2.85889       -3.66942
H         -0.00217       -3.39675       -4.61497
C         -0.00216       -3.54638        2.48735
C         -0.00173       -2.85889        3.66942
H         -0.00273       -4.63419        2.48501
H         -0.00217       -3.39675        4.61497
C         -0.00089       -1.42434        3.69978
C         -0.00043       -0.69139        4.91213
H         -0.00091       -1.23541        5.85410
C          0.00043        0.69139        4.91213
C          0.00089        1.42434        3.69978
H          0.00091        1.23541        5.85410
C          0.00173        2.85889        3.66942
C          0.00216        3.54638        2.48735
H          0.00217        3.39675        4.61497
H          0.00273        4.63419        2.48501
```

6.2. Methylated π-systems

```
18
01m_12.log       Energy: -195102.5463172
C          1.96520        0.69809       -0.00002
C          0.74772        1.38711       -0.00001
C         -0.47842        0.70721        0.00002
C         -0.47842       -0.70720        0.00001
C          0.74772       -1.38711        0.00002
C          1.96520       -0.69810        0.00001
H          2.90236        1.24991       -0.00004
H          0.74809        2.47563       -0.00001
H          0.74808       -2.47562        0.00003
H          2.90236       -1.24992        0.00001
C         -1.77920       -1.47502       -0.00002
H         -2.38950       -1.23420        0.88096
H         -2.38951       -1.23408       -0.88097
H         -1.60323       -2.55609       -0.00010
C         -1.77920        1.47502        0.00001
H         -2.38949        1.23416       -0.88097
H         -2.38952        1.23411        0.88095
H         -1.60325        2.55609        0.00004
21
01m_135.log      Energy: -219776.9856556
C          1.32812        0.41636       -0.00088
C          0.30259        1.37925       -0.00100
C         -1.02461        0.94181       -0.00045
C         -1.34582       -0.42782        0.00042
C         -0.30348       -1.35856        0.00070
C          1.04323       -0.95172        0.00005
H          2.36618        0.74675       -0.00159
H         -1.82992        1.67545       -0.00083
H         -0.53612       -2.42280        0.00123
C         -2.79247       -0.86677        0.00018
H         -3.32452       -0.48073        0.87977
H         -3.32118       -0.49010       -0.88551
H         -2.88273       -1.95865        0.00567
C          0.64530        2.85169        0.00004
H         -0.25513        3.47559       -0.01893
H          1.22368        3.12362        0.89304
H          1.25739        3.11748       -0.87189
C          2.14711       -1.98453        0.00045
H          2.08432       -2.63211        0.88501
H          2.08066       -2.63690       -0.88028
H          3.13745       -1.51586       -0.00280
18
01m_13.log       Energy: -195102.3282771
C         -1.20987        1.13748       -0.00031
C         -1.23236       -0.26248       -0.00045
C         -0.00634       -0.95074       -0.00034
C          1.22219       -0.27987        0.00005
C          1.21562        1.12626        0.00028
C          0.00954        1.82712        0.00008
H         -2.14604        1.69209       -0.00054
H         -0.01486       -2.04034       -0.00060
H          2.16004        1.66767        0.00050
H          0.01461        2.91504        0.00013
C          2.53050       -1.03712        0.00003
```

```
H          3.13365       -0.78607        0.88250
H          2.36974       -2.12087        0.00039
H          3.13332       -0.78662       -0.88283
C         -2.53392       -1.03117        0.00033
H         -2.60768       -1.68786       -0.87652
H         -2.61607       -1.67117        0.88880
H         -3.39879       -0.35873       -0.00989
18
01m_14.log        Energy: -195102.1789468
C          0.70033        1.20417        0.00413
C         -0.70033        1.20416       -0.00412
C         -1.42508        0.00590       -0.00756
C         -0.69758       -1.19671       -0.00419
C          0.69758       -1.19671        0.00420
C          1.42508        0.00591        0.00756
H          1.23392        2.15295        0.00666
H         -1.23393        2.15295       -0.00665
H         -1.23360       -2.14462       -0.00711
H          1.23361       -2.14461        0.00713
C          2.93628       -0.00713       -0.00261
H          3.33582       -0.66703        0.77799
H          3.32313       -0.37394       -0.96324
H          3.34795        0.99525        0.15977
C         -2.93628       -0.00713        0.00259
H         -3.32315       -0.37422        0.96311
H         -3.34793        0.99530       -0.15951
H         -3.33582       -0.66681       -0.77819
15
01m_1.log        Energy: -170427.6495205
C         -1.90876       -0.00471        0.00596
C         -1.19767       -1.21067        0.00144
C          0.19969       -1.20232       -0.00658
C          0.91657        0.00617       -0.00941
C          0.19159        1.20722       -0.00656
C         -1.20815        1.20488        0.00159
H         -2.99616       -0.00943        0.01033
H         -1.73208       -2.15809        0.00205
H          0.74400       -2.14523       -0.01137
H          0.72759        2.15445       -0.01162
H         -1.74889        2.14866        0.00232
C          2.42765        0.00248        0.00688
H          2.81017       -0.29664        0.99243
H          2.83494       -0.70610       -0.72493
H          2.83492        0.99415       -0.21915
17
02m_1.log        Energy: -218201.9338525
C          1.29806       -0.26531        0.00001
C          1.54667        1.21861        0.00001
C          0.36154        1.88151        0.00000
C         -0.70201        0.85826       -0.00002
C         -0.05522       -0.44412        0.00000
C         -2.05101        0.66986        0.00000
C         -2.30503       -0.79767       -0.00001
C         -1.11406       -1.46362        0.00001
H          2.53958        1.65694        0.00000
H          0.21792        2.95642       -0.00002
H         -2.83116        1.42544        0.00003
H         -3.29364       -1.24434       -0.00001
H         -0.97466       -2.53890        0.00001
C          2.39554       -1.27156       -0.00001
H          3.04063       -1.13698        0.88018
H          2.01389       -2.29726        0.00002
H          3.04058       -1.13703       -0.88024
17
02m_2.log        Energy: -218199.9988643
C          0.76961       -1.15779        0.00003
C          1.68364        0.04187       -0.00000
C          0.92241        1.16921       -0.00002
C         -0.49078        0.74810       -0.00002
C         -0.51185       -0.71704        0.00002
C         -1.77350        1.18989       -0.00003
C         -2.67410       -0.00970        0.00000
C         -1.92492       -1.14312        0.00002
H          1.12840       -2.18358        0.00005
H          1.28920        2.18981       -0.00004
H         -2.12898        2.21591       -0.00005
H         -3.75756        0.04754       -0.00000
H         -2.28778       -2.16452        0.00005
C          3.17360       -0.07886       -0.00000
H          3.52811       -0.63264        0.88153
H          3.52811       -0.63267       -0.88152
H          3.65578        0.90481       -0.00002
21
03m_1.log        Energy: -266849.3996896
C          1.83917       -1.72560        0.00010
C          0.49069       -2.00284        0.00027
C         -0.45885       -0.94267        0.00019
C          0.00126        0.41869       -0.00006
C          1.41252        0.68513       -0.00023
C          2.29269       -0.38232       -0.00015
H         -2.19524       -2.23758        0.00054
H          2.56627       -2.53426        0.00016
H          0.13361       -3.03057        0.00046
C         -1.85790       -1.20306        0.00035
C         -0.97288        1.45824       -0.00014
H          3.36315       -0.18776       -0.00028
C         -2.32382        1.17225        0.00003
C         -2.77311       -0.17151        0.00027
H         -0.64998        2.49489       -0.00032
H         -3.04956        1.98209       -0.00003
H         -3.83916       -0.38548        0.00040
C          1.92918        2.10379       -0.00050
H          1.58655        2.65923       -0.88351
H          1.58666        2.65952        0.88237
H          3.02402        2.11932       -0.00057
21
03m_2.log        Energy: -266850.0217560
C          1.82620        1.21472        0.00000
C          0.53342        1.68858        0.00001
C         -0.57334        0.79294        0.00001
C         -0.30978       -0.61648        0.00001
C          1.04062       -1.06775        0.00003
C          2.10305       -0.18453        0.00002
H         -2.11619        2.31516       -0.00001
H          2.65964        1.91490       -0.00002
H          0.34130        2.75973        0.00001
C         -1.92187        1.24433       -0.00000
C         -1.40997       -1.51872        0.00000
H          1.22758       -2.14052        0.00004
C         -2.70849       -1.05104       -0.00001
C         -2.96803        0.34387       -0.00001
H         -1.21020       -2.58843        0.00001
H         -3.53923       -1.75266       -0.00003
H         -3.99529        0.70011       -0.00002
C          3.53569       -0.65898       -0.00003
H          4.07434       -0.28872       -0.88249
H          4.07436       -0.28878        0.88244
H          3.59871       -1.75255       -0.00008
21
04m_1.log        Energy: -266816.6994853
C          2.69713       -0.69550       -0.00000
C          2.58581        0.70133       -0.00002
C          1.42497        1.48522       -0.00002
C          1.69591       -1.67460        0.00002
C          0.09163        1.07781        0.00000
C          0.30417       -1.50546        0.00002
C         -0.43820       -0.32661        0.00001
H          3.71720       -1.07767       -0.00001
H          3.52570        1.24998       -0.00003
H          1.58225        2.56418       -0.00002
H          2.04412       -2.70560        0.00003
H         -0.27598       -2.42805        0.00002
C         -1.02971        1.92753        0.00004
H         -0.99288        3.01152        0.00007
C         -2.18890        1.13014       -0.00002
H         -3.20773        1.50790       -0.00003
C         -1.85104       -0.23789       -0.00000
C         -2.81756       -1.38823       -0.00002
H         -2.69325       -2.02975       -0.88333
H         -2.69322       -2.02979        0.88326
H         -3.85149       -1.02517        0.00001
21
04m_2.log        Energy: -266817.9116510
C         -2.97627        0.00039        0.00001
C         -2.38190        1.26703        0.00001
C         -1.01944        1.59749        0.00000
C         -2.37944       -1.26897        0.00000
C          0.08685        0.75027       -0.00000
C         -1.02043       -1.59862       -0.00001
C          0.08918       -0.74962       -0.00001
H         -4.06569       -0.00063        0.00001
H         -3.06960        2.11052        0.00001
H         -0.79169        2.66381        0.00000
H         -3.06781       -2.11193        0.00000
H         -0.79175       -2.66477       -0.00000
C          1.43802        1.14904       -0.00002
H          1.78748        2.17655       -0.00003
C          2.25868        0.00423       -0.00001
C          1.43654       -1.14626       -0.00000
H          1.78628       -2.17388       -0.00000
C          3.75867       -0.00024        0.00002
H          4.15313       -0.52263        0.88211
H          4.15317       -0.52303       -0.88183
H          4.16373        1.01756       -0.00021
```

21
04m_4.log   Energy: -266816.2969072

C	-2.41268	-0.65329	-0.00002
C	-1.68255	-1.84455	-0.00002
C	-0.29178	-1.99693	-0.00002
C	-1.97708	0.67905	-0.00001
C	0.69099	-1.01029	-0.00001
C	-0.67878	1.21688	0.00002
C	0.52268	0.47902	0.00001
H	-3.49512	-0.77516	-0.00003
H	-2.26526	-2.76355	-0.00002
H	0.07643	-3.02303	0.00002
H	-2.78009	1.41363	-0.00002
C	2.08165	-1.26535	0.00012
H	2.53075	-2.25260	0.00022
C	2.75714	-0.03862	-0.00007
H	3.83598	0.08467	-0.00010
C	1.82290	1.01921	-0.00006
H	2.07226	2.07298	-0.00011
C	-0.56515	2.72786	0.00005
H	-0.01533	3.07931	0.88214
H	-0.01525	3.07935	-0.88198
H	-1.54850	3.20651	0.00001

21
04m_5.log   Energy: -266815.2131375

C	1.95921	0.98542	-0.00007
C	0.97499	1.97847	0.00002
C	-0.42014	1.84711	0.00004
C	1.86000	-0.41954	-0.00009
C	-1.17749	0.67925	0.00001
C	0.66716	-1.15781	0.00001
C	-0.66143	-0.72640	0.00003
H	2.98100	1.36607	-0.00011
H	1.34754	3.00103	0.00004
H	-0.98562	2.77924	0.00008
H	0.78612	-2.24158	0.00008
C	-2.58578	0.59472	0.00001
H	-3.26236	1.44238	0.00001
C	-2.94793	-0.76335	-0.00005
H	-3.96739	-1.13745	-0.00009
C	-1.79153	-1.56765	0.00001
H	-1.76199	-2.65193	0.00001
C	3.17246	-1.18577	0.00003
H	3.77725	-0.93685	-0.88204
H	3.77584	-0.93888	0.88365
H	3.01254	-2.26874	-0.00132

21
04m_6.log   Energy: -266816.2188469

C	-2.09422	-0.01465	-0.00005
C	-1.46091	-1.26873	-0.00004
C	-0.09630	-1.58700	-0.00002
C	-1.46798	1.24976	-0.00004
C	1.01275	-0.74444	-0.00000
C	-0.11304	1.58249	-0.00001
C	1.00753	0.74745	-0.00000
H	-2.12747	-2.12882	-0.00005
H	0.13022	-2.65378	-0.00001
H	-2.14915	2.09964	-0.00004
H	0.10494	2.65102	-0.00001
C	2.36688	-1.14565	0.00002
H	2.71380	-2.17337	0.00003
C	3.16988	0.00728	0.00001
H	4.25574	0.01278	0.00002
C	2.35397	1.15735	0.00001
H	2.69446	2.18729	0.00002
C	-3.61150	0.00539	0.00006
H	-3.99320	0.53503	0.88275
H	-3.99335	0.53662	-0.88160
H	-4.03836	-1.00193	-0.00081

23
05m_1.log   Energy: -314668.5246654

C	-2.67780	-0.29072	0.00000
C	-2.26459	1.03459	0.00000
C	-0.87117	1.34677	0.00000
C	0.00000	0.25308	0.00000
C	-0.40226	-1.10449	0.00000
C	-1.75663	-1.38199	0.00000
H	-0.87333	3.53364	0.00000
H	-3.74238	-0.51231	0.00000
H	-3.00118	1.83524	0.00000
C	-0.25692	2.63737	0.00000
C	1.41589	0.33924	0.00000
H	-2.13458	-2.40197	0.00000
C	1.98579	1.59892	0.00000
C	1.12626	2.74131	0.00000
H	3.06411	1.74202	0.00000
H	1.58135	3.72888	0.00000
C	0.84081	-1.91160	0.00000
C	1.90512	-1.04949	0.00000
H	2.95034	-1.34218	0.00000
C	0.87566	-3.40744	0.00000
H	0.36464	-3.81789	0.88213
H	0.36464	-3.81789	-0.88213
H	1.90547	-3.78080	0.00000

23
05m_3.log   Energy: -314667.7446711

C	-1.76340	-1.62746	0.00000
C	-0.47837	-2.14210	0.00000
C	0.64423	-1.25717	0.00002
C	0.33728	0.10662	0.00004
C	-0.97464	0.64818	0.00003
C	-2.05593	-0.22149	0.00001
H	2.33857	-2.63478	-0.00001
H	-2.60573	-2.31704	-0.00002
H	-0.32256	-3.21877	-0.00001
C	2.02997	-1.59167	-0.00000
C	1.29790	1.14958	0.00001
C	2.63822	0.80009	-0.00001
C	2.98559	-0.58255	-0.00002
H	3.42584	1.55062	-0.00003
H	4.03903	-0.85211	-0.00004
C	-0.80404	2.11169	0.00001
H	-1.61109	2.83617	-0.00000
C	0.53249	2.40598	-0.00001
H	0.96709	3.40007	-0.00002
C	-3.49147	0.23428	-0.00002
H	-4.02247	-0.14683	0.88228
H	-3.57341	1.32527	0.00016
H	-4.02231	-0.14648	-0.88257

23
05m_4.log   Energy: -314666.6741186

C	2.28178	-0.20166	-0.00017
C	1.43739	-1.30632	-0.00005
C	0.01785	-1.14350	0.00004
C	-0.45095	0.17200	0.00012
C	0.38010	1.32030	0.00001
C	1.74770	1.13103	-0.00012
H	-0.69536	-3.20931	-0.00006
H	1.86141	-2.30862	0.00002
C	-0.98386	-2.16045	-0.00004
C	-1.81499	0.55281	0.00012
H	2.43961	1.97184	-0.00014
C	-2.76789	-0.45112	-0.00000
C	-2.32787	-1.80878	-0.00006
H	-3.83361	-0.23298	-0.00001
H	-3.07888	-2.59527	-0.00009
C	-0.53114	2.47872	-0.00012
H	-0.21784	3.51739	-0.00027
C	-1.82244	2.02705	0.00011
H	-2.71470	2.64430	0.00018
C	3.78374	-0.37058	0.00013
H	4.23579	0.10130	-0.88239
H	4.23550	0.10160	0.88265
H	4.07168	-1.42727	0.00044

23
05m_5.log   Energy: -314667.1768591

C	-2.02112	-1.34188	0.00000
C	-0.71489	-1.82321	0.00000
C	0.36058	-0.86713	0.00000
C	0.00000	0.48519	0.00000
C	-1.33038	0.97415	0.00000
C	-2.35353	0.04543	0.00000
H	2.12790	-2.15687	0.00000
H	-2.83705	-2.06169	0.00000
C	1.76327	-1.13328	0.00000
C	0.91393	1.57071	0.00000
H	-3.40042	0.34174	0.00000
C	2.26741	1.28350	0.00000
C	2.67290	-0.08321	0.00000
H	3.02162	2.06752	0.00000
H	3.73682	-0.30813	0.00000
C	-1.22746	2.44296	0.00000
H	-2.07037	3.12611	0.00000
C	0.09421	2.79485	0.00000
H	0.48710	3.80607	0.00000
C	-0.42835	-3.30350	0.00000
H	0.15482	-3.59776	-0.88258
H	0.15482	-3.59776	0.88258
H	-1.35475	-3.88679	0.00000

23
06m_1.log   Energy: -314637.8457519

C	0.28971	-1.44677	-0.00000
C	-1.06863	-0.85669	0.00000
C	-0.94995	0.57074	0.00000

```
C          0.48949       0.85699       0.00000
C          1.19252      -0.43071       0.00000
C          1.42638       1.84355      -0.00001
C          2.76423       1.19939      -0.00003
C          2.64104      -0.16192      -0.00001
H          0.48756      -2.51475      -0.00001
H          1.26551       2.91721      -0.00002
H          3.70191       1.74675      -0.00005
C         -2.08838       1.36254       0.00001
C         -3.35465       0.73690       0.00001
H         -2.01447       2.44739       0.00001
H         -4.25189       1.35115       0.00001
C         -2.31522      -1.46571       0.00000
C         -3.46897      -0.65284       0.00001
H         -2.40556      -2.54960       0.00000
H         -4.45252      -1.11566       0.00001
C          3.71959      -1.19699       0.00001
H          3.64677      -1.84891       0.88180
H          3.64667      -1.84905      -0.88167
H          4.71302      -0.73543      -0.00008
23
06m_2.log        Energy: -314638.2313207
C          0.19629       1.77297       0.00001
C          1.36234       0.85570      -0.00002
C          0.88670      -0.49359       0.00005
C         -0.58064      -0.40460       0.00015
C         -0.93748       1.02382       0.00018
C         -1.73014      -1.12530      -0.00003
C         -2.87875      -0.16692      -0.00004
C         -2.39702       1.11168       0.00006
H          0.27886       2.85581      -0.00012
H         -1.84822      -2.20487      -0.00020
C          1.78178      -1.55059       0.00008
C          3.16748      -1.27013       0.00000
H          1.43204      -2.58019       0.00015
H          3.87825      -2.09291       0.00002
C          2.72202       1.12590      -0.00010
C          3.63045       0.04378      -0.00009
H          3.08732       2.15030      -0.00018
H          4.69940       0.24158      -0.00015
C         -4.30529      -0.61533      -0.00013
H         -4.52496      -1.23481       0.88130
H         -4.99552       0.23535      -0.00010
H         -4.52491      -1.23474      -0.88161
H         -2.98860       2.02023       0.00014
23
06m_3.log        Energy: -314639.0327300
C          0.14730      -1.96150       0.00001
C          1.21979      -0.94283       0.00001
C          0.61902       0.35853       0.00003
C         -0.83109       0.13397       0.00005
C         -1.04973      -1.31456       0.00003
C         -2.04222       0.76063      -0.00001
C         -3.08293      -0.31285      -0.00003
C         -2.49754      -1.54275      -0.00000
H          0.32607      -3.03263      -0.00000
C          1.42000       1.49171       0.00002
C          2.82333       1.33655       0.00000
H          0.98303       2.48691       0.00002
H          3.45597       2.22097      -0.00000
C          2.60033      -1.08812      -0.00003
C          3.40652       0.06946      -0.00003
H          3.05543      -2.07612      -0.00006
H          4.48910      -0.02891      -0.00004
H         -2.99803      -2.50423      -0.00001
H         -4.14938      -0.10786      -0.00007
C         -2.36261       2.21788      -0.00002
H         -2.96306       2.48629       0.88105
H         -1.45728       2.83243       0.00009
H         -2.96288       2.48635      -0.88119
23
06m_4.log        Energy: -314637.4980502
C          0.71733      -1.89452       0.00000
C         -0.60953      -1.23404       0.00000
C         -0.41709       0.18247       0.00000
C          1.03563       0.39497       0.00000
C          1.67457      -0.92851      -0.00000
C          2.01610       1.33909       0.00001
C          3.32298       0.63022       0.00000
C          3.12401      -0.72011       0.00000
H          0.85881      -2.97130       0.00000
C         -1.49781       1.05690      -0.00001
C         -2.79377       0.48151      -0.00001
H         -3.65814       1.14242      -0.00001
C         -1.88417      -1.77869       0.00000
C         -2.98727      -0.89765       0.00000
H         -2.03557      -2.85551       0.00000
H         -3.99723      -1.29965       0.00000
H          3.88555      -1.49142       0.00000
H          4.28405       1.13451       0.00001
C         -1.29281       2.55028      -0.00000
H         -0.72283       2.87149       0.88247
H         -0.72261       2.87146      -0.88235
H         -2.24705       3.08826      -0.00013
H          1.90591       2.41823       0.00001
23
06m_5.log        Energy: -314636.5724361
C         -1.10228       1.71642       0.00237
C          0.26368       1.14986       0.00067
C          0.16957      -0.27920      -0.00047
C         -1.26766      -0.59035      -0.00021
C         -1.99270       0.68655       0.00192
C         -2.18184      -1.59800      -0.00199
C         -3.53201      -0.97949      -0.00061
C         -3.42342       0.38215       0.00186
H         -1.31788       2.78099       0.00366
C          1.31423      -1.05347      -0.00253
C          2.59049      -0.42856      -0.00386
C          1.50333       1.76719      -0.00134
C          2.66661       0.96537      -0.00413
H          1.58785       2.85160      -0.00147
H          3.64209       1.44627      -0.00566
H         -4.23470       1.10085       0.00338
H         -4.45736      -1.54650      -0.00147
H         -1.99898      -2.66779      -0.00416
H          1.24990      -2.14008      -0.00352
C          3.83415      -1.28423       0.00497
H          3.90427      -1.86933       0.93220
H          3.82890      -2.00285      -0.82510
H          4.74299      -0.67865      -0.07782
23
06m_6.log        Energy: -314636.3052494
C         -0.75063      -1.64553      -0.00036
C          0.44198      -0.76594      -0.00005
C          0.00725       0.59755       0.00016
C         -1.46200       0.55602       0.00027
C         -1.86040      -0.85961      -0.00021
C         -2.59158       1.31306       0.00073
C         -3.75453       0.38698       0.00042
C         -3.32362      -0.90813      -0.00017
H         -0.70383      -2.73067      -0.00071
C          0.94464       1.61420      -0.00045
C          2.31953       1.28032      -0.00114
C          1.78907      -1.08297      -0.00044
C          2.75675      -0.04495      -0.00086
H          2.11430      -2.12199      -0.00069
H         -3.93851      -1.80052      -0.00058
H         -4.78876       0.71604       0.00063
H         -2.67221       2.39532       0.00127
H          0.64057       2.65831      -0.00070
C          4.22593      -0.39431       0.00133
H          4.85347       0.50315      -0.02189
H          4.49609      -0.96978       0.89703
H          4.48924      -1.01170      -0.86787
H          3.05535       2.08173      -0.00190
23
06m_7.log        Energy: -314636.6188485
C         -0.35765      -1.50129       0.00002
C          0.70893      -0.47209      -0.00004
C          0.09377       0.81961      -0.00004
C         -1.35709       0.58145       0.00002
C         -1.56388      -0.87114       0.00005
C         -2.57669       1.18407       0.00005
C         -3.60754       0.11338       0.00011
C         -3.00795      -1.11316       0.00011
H         -0.17302      -2.57104       0.00004
C          0.86947       1.96653      -0.00007
C          2.27421       1.82509      -0.00010
C          2.09450      -0.62072      -0.00007
C          2.86861       0.56548      -0.00010
H         -3.49955      -2.07899       0.00015
H         -4.67603       0.30335       0.00015
H         -2.79892       2.24642       0.00004
H          0.41463       2.95382      -0.00006
H          2.90088       2.71375      -0.00013
H          3.95344       0.48486      -0.00013
C          2.74820      -1.98135       0.00000
H          2.45905      -2.56515       0.88357
H          2.45801      -2.56578      -0.88280
H          3.84009      -1.89641      -0.00064
23
06m_8.log        Energy: -314640.2892079
C         -0.44560       1.35729      -0.00001
C          0.84176       0.60408       0.00000
```

```
C          0.56449       -0.79795        0.00000
C         -0.89960       -0.92053       -0.00001
C         -1.45287        0.43517       -0.00001
C         -1.93576       -1.80497       -0.00001
C         -3.19221       -1.02016       -0.00002
C         -2.90873        0.31822       -0.00002
C          1.60443       -1.71328        0.00001
C          2.93403       -1.23444        0.00002
C          2.14715        1.06995        0.00001
C          3.20392        0.13261        0.00002
H         -3.62346        1.13318       -0.00003
H         -4.18330       -1.46278       -0.00002
H         -1.89021       -2.88945       -0.00001
H          1.40787       -2.78271        0.00002
H          3.75566       -1.94685        0.00003
H          4.23318        0.48259        0.00002
C         -0.53335        2.84380        0.00000
H         -0.02645        3.26202        0.88104
H         -0.02640        3.26204       -0.88099
H         -1.57255        3.18649       -0.00002
H          2.35968        2.13673        0.00001
25
07m_1.log         Energy: -315431.1424332
C         -2.92565        0.77386       -0.83639
C         -1.52878        0.75228       -0.84077
C         -0.81923       -0.16420       -0.04564
C         -1.55054       -1.06307        0.75059
C         -2.94816       -1.04063        0.75960
C         -3.64156       -0.12092       -0.03357
H         -3.45491        1.48735       -1.46373
H         -0.98232        1.44353       -1.47659
H         -1.01710       -1.77507        1.37591
H         -3.49412       -1.73938        1.38922
H         -4.72871       -0.10208       -0.02755
C          0.67192       -0.21590       -0.06071
C          1.29668       -1.44051       -0.35898
C          1.47182        0.91540        0.22994
C          2.68657       -1.56107       -0.39411
H          0.67526       -2.30498       -0.58089
C          2.86820        0.77418        0.19125
C          3.47843       -0.44278       -0.12065
H          3.14453       -2.51729       -0.63531
H          3.48824        1.63819        0.42302
H          4.56316       -0.51696       -0.14215
C          0.87839        2.25717        0.60108
H          0.61891        2.84649       -0.28805
H         -0.03647        2.15023        1.19315
H          1.59507        2.84717        1.18312
25
07m_2.log         Energy: -315432.8940349
C          3.04132       -1.31392       -0.51307
C          1.66182       -1.09850       -0.55688
C          1.09617        0.07624       -0.02771
C          1.95850        1.03011        0.54404
C          3.33824        0.81604        0.58731
C          3.88652       -0.35770        0.05974
H          3.45688       -2.22615       -0.93489
H          1.01865       -1.83896       -1.02553
H          1.54322        1.93629        0.97757
H          3.98433        1.56374        1.04143
H          4.96021       -0.52536        0.09390
C         -0.37323        0.30292       -0.07026
C         -0.90026        1.57618       -0.35687
C         -1.26936       -0.75130        0.17403
C         -2.28059        1.77370       -0.39862
H         -0.22936        2.40362       -0.57238
C         -2.65908       -0.56697        0.13876
H         -0.87687       -1.73581        0.42119
C         -3.15648        0.71074       -0.15374
H         -2.67740        2.75861       -0.63412
H         -4.23131        0.87543       -0.19000
C         -3.58723       -1.72003        0.44479
H         -3.29557       -2.62594       -0.10085
H         -3.56716       -1.96716        1.51501
H         -4.62361       -1.48340        0.17951
25
07m_3.log         Energy: -315432.9344936
C          3.33732       -1.14799       -0.38261
C          1.94059       -1.14428       -0.38478
C          1.21806        0.00116       -0.00061
C          1.94447        1.14390        0.38419
C          3.34122        1.14253        0.38270
C          4.04543       -0.00402        0.00029
H          3.87277       -2.04348       -0.68959
H          1.40470       -2.03264       -0.70909
H          1.41147        2.03399        0.70851
H          3.87977        2.03597        0.69021
H          5.13272       -0.00605        0.00078
C         -0.26848        0.00357       -0.00109
C         -0.99591        1.14270       -0.38560
C         -0.99879       -1.13654        0.38535
C         -2.39320        1.14196       -0.38291
H         -0.46675        2.03445       -0.71213
C         -2.39294       -1.13307        0.38443
H         -0.46986       -2.02814        0.71266
C         -3.11937        0.00599        0.00029
H         -2.92584        2.03902       -0.69267
H         -2.92852       -2.02796        0.69687
C         -4.62935       -0.00792       -0.00049
H         -5.02482       -0.28648        0.98480
H         -5.04056        0.97258       -0.26417
H         -5.01930       -0.73927       -0.72109
26
08m_1.log         Energy: -339353.8843215
H          0.14363       -2.26410       -0.87959
C         -3.47586        0.85967       -0.00007
C         -3.68343       -0.52754       -0.00003
C         -2.59437       -1.41151        0.00001
C         -1.30293       -0.89170        0.00000
C         -1.09403        0.50480       -0.00004
C         -2.17963        1.38643       -0.00008
H         -4.33048        1.53207       -0.00011
H         -4.69718       -0.92069       -0.00002
H         -2.76042       -2.48687        0.00005
H         -2.02117        2.46231       -0.00012
C          0.02795       -1.61479        0.00004
H          0.14361       -2.26406        0.87969
C          1.02722       -0.47752        0.00002
C          2.42380       -0.54576        0.00006
C          3.12847        0.67086        0.00002
C          2.46249        1.90500       -0.00003
C          1.06588        1.96388       -0.00005
C          0.35240        0.76125       -0.00003
H          4.21658        0.65205        0.00003
H          3.04196        2.82538       -0.00005
H          0.55003        2.92099       -0.00009
C          3.13514       -1.87646        0.00015
H          2.86591       -2.47331        0.88243
H          2.86547       -2.47369       -0.88173
H          4.22333       -1.74972       -0.00014
26
08m_2.log         Energy: -339352.9116661
H          0.34660       -2.46584        0.87130
C          3.49213        1.13889        0.00742
C          3.89265       -0.20509        0.00703
C          2.93853       -1.23348        0.00263
C          1.58690       -0.90077       -0.00139
C          1.18350        0.45289       -0.00072
C          2.13449        1.47823        0.00367
H          4.24412        1.92455        0.01079
H          4.95169       -0.45187        0.01008
H          3.25438       -2.27477        0.00241
H          1.82715        2.52145        0.00426
C          0.37096       -1.80535       -0.00651
H          0.34947       -2.45862       -0.88983
C         -0.77926       -0.81898       -0.00527
C         -2.14903       -1.06222       -0.01035
C         -3.05411        0.01443       -0.01056
C         -2.54635        1.32700       -0.01229
C         -1.17167        1.58204       -0.00791
C         -0.28431        0.50206       -0.00324
H         -2.52585       -2.08386       -0.01512
H         -3.24376        2.16248       -0.01818
H         -0.80439        2.60582       -0.01180
C         -4.54485       -0.23347        0.02364
H         -4.80505       -1.18849       -0.44749
H         -4.91463       -0.26903        1.05816
H         -5.09725        0.56108       -0.49150
26
08m_3.log         Energy: -339352.9871875
H         -0.81917        2.60281        0.88087
C         -3.13722       -1.57597        0.00000
C         -3.81008       -0.34530       -0.00003
C         -3.09247        0.85992       -0.00003
C         -1.70099        0.81749       -0.00001
C         -1.02351       -0.42198        0.00002
C         -1.73887       -1.62370        0.00003
H         -3.70796       -2.50165        0.00001
H         -4.89736       -0.32559       -0.00006
H         -3.61935        1.81207       -0.00006
H         -1.21981       -2.57931        0.00006
C         -0.70186        1.95652       -0.00003
H         -0.81913        2.60272       -0.88101
C          0.62809        1.23106       -0.00001
```

```
C          1.92437        1.74054        0.00002
C          3.00610        0.85168        0.00006
C          2.81479       -0.54396        0.00008
C          1.50684       -1.04719        0.00011
C          0.42243       -0.16329        0.00005
H          2.10040        2.81445        0.00001
H          4.02134        1.24450        0.00008
H          1.33924       -2.12241        0.00015
C          4.00902       -1.47125       -0.00013
H          4.64135       -1.30480        0.88170
H          3.70181       -2.52285        0.00174
H          4.63893       -1.30735       -0.88419
26
08m_4.log        Energy: -339352.8545226
H         -0.36842       -2.71403       -0.88054
C         -3.03258        1.23768       -0.00015
C         -3.59402       -0.04547       -0.00015
C         -2.76617       -1.17686       -0.00009
C         -1.38577       -1.00572       -0.00004
C         -0.81009        0.28721       -0.00004
C         -1.64381        1.41504       -0.00009
H         -3.68209        2.10991       -0.00019
H         -4.67477       -0.16446       -0.00019
H         -3.19838       -2.17544       -0.00009
H         -1.23610        2.41891       -0.00010
C         -0.30400       -2.05979        0.00003
H         -0.36849       -2.71398        0.88062
C          0.96481       -1.23759        0.00006
C          2.28172       -1.68609        0.00012
C          3.31087       -0.73762        0.00014
C          3.01191        0.63042        0.00011
C          1.69065        1.10494        0.00005
C          0.65951        0.14343        0.00003
H          2.50694       -2.75053        0.00014
H          4.34934       -1.06057        0.00018
H          3.82657        1.35203        0.00013
C          1.41799        2.58920        0.00002
H          0.84264        2.89385       -0.88369
H          0.84259        2.89388        0.88369
H          2.35395        3.15780        0.00004
26
08m_5.log        Energy: -339351.8490936
C         -3.01912       -1.46075        0.10507
C         -3.46300       -0.14349       -0.08076
C         -2.54258        0.90334       -0.23736
C         -1.18087        0.61657       -0.20534
C         -0.73463       -0.70877       -0.01351
C         -1.65107       -1.75365        0.14044
H         -3.74510       -2.26164        0.22373
H         -4.52944        0.06785       -0.10370
H         -2.89242        1.92345       -0.38084
H         -1.31018       -2.77616        0.28591
C          0.00000        1.56270       -0.34582
H          0.00000        2.00542       -1.35372
C          1.18087        0.61657       -0.20534
C          2.54258        0.90334       -0.23736
C          3.46300       -0.14349       -0.08076
C          3.01912       -1.46075        0.10507
C          1.65107       -1.75365        0.14044
C          0.73463       -0.70877       -0.01351
H          2.89242        1.92345       -0.38084
H          4.52944        0.06785       -0.10370
H          3.74510       -2.26164        0.22373
H          1.31018       -2.77616        0.28591
C          0.00000        2.71126        0.67861
H          0.88699        3.34259        0.55087
H         -0.88699        3.34259        0.55087
H          0.00000        2.31961        1.70255
25
09m_1.log        Energy: -362471.0748702
C          1.67096       -1.82937        0.00000
C          0.29531       -2.04294        0.00000
C         -0.51162       -0.88923        0.00000
C          0.00000        0.36595        0.00000
C          1.38068        0.62716        0.00000
C          2.21754       -0.48396        0.00000
H          2.37412       -2.65936        0.00000
C         -1.89534       -1.15163        0.00000
C         -0.80366        1.52332        0.00000
H          3.30032       -0.37952        0.00000
C         -2.17818        1.30950        0.00000
C         -2.72731       -0.03679        0.00000
H         -2.88188        2.13901        0.00000
H         -3.81046       -0.13752        0.00000
C          1.46438        2.12697        0.00000
C          0.18913        2.63461        0.00000
H         -0.05070        3.69308        0.00000
C         -0.69522       -3.15864        0.00000
C         -1.96463       -2.64154        0.00000
H         -0.45116       -4.21571        0.00000
H         -2.87759       -3.22775        0.00000
C          2.74539        2.89748        0.00000
H          3.35433        2.65236        0.88173
H          3.35433        2.65236       -0.88173
H          2.56406        3.97770        0.00000
25
09m_2.log        Energy: -362469.7607023
C          0.88382       -2.03919        0.00003
C         -0.46758       -1.72423        0.00009
C         -0.78582       -0.35154        0.00022
C          0.16297        0.61582        0.00008
C          1.54391        0.33735        0.00003
C          1.91898       -1.00524        0.00003
H          1.22163       -3.07446       -0.00007
C         -2.16376       -0.07668        0.00008
C         -0.15068        1.98786        0.00002
C         -1.50726        2.30766       -0.00002
C         -2.52003        1.27029       -0.00002
H         -1.84467        3.34184       -0.00007
H         -3.56220        1.58196       -0.00013
C          2.16728        1.69252       -0.00005
C          1.18504        2.64911       -0.00005
H          1.35688        3.72036       -0.00015
C         -1.80438       -2.38878       -0.00009
C         -2.78693       -1.43411       -0.00011
H         -1.97409       -3.46030       -0.00020
H         -3.85297       -1.63596       -0.00023
C          3.35972       -1.44893       -0.00005
H          4.04455       -0.59496        0.00004
H          3.58330       -2.06318        0.88246
H          3.58323       -2.06296       -0.88274
H          3.23267        1.89622       -0.00014
24
10m_1.log        Energy: -361884.0545235
C         -3.06275        1.18274       -0.00014
C         -3.55085       -0.13675       -0.00015
C         -2.67979       -1.23157       -0.00010
C         -1.32137       -0.94570       -0.00005
C         -0.79794        0.36386       -0.00004
C         -1.69063        1.44628       -0.00009
H         -3.76669        2.01091       -0.00018
H         -4.62343       -0.31209       -0.00019
H         -3.04114       -2.25548       -0.00010
H         -1.32621        2.46833       -0.00009
C          0.86832       -1.18791        0.00005
C          2.12928       -1.76943        0.00010
C          3.21251       -0.88673        0.00014
C          3.02177        0.50704        0.00012
C          1.74633        1.08517        0.00007
C          0.64740        0.20316        0.00003
H          2.25860       -2.84714        0.00012
H          4.22378       -1.28508        0.00018
H          3.89283        1.15833        0.00015
O         -0.31834       -1.88941        0.00001
C          1.55136        2.57949        0.00005
H          0.98738        2.90779       -0.88296
H          0.98731        2.90781        0.88301
H          2.51266        3.10392        0.00008
24
10m_2.log        Energy: -361882.7156172
C         -2.82498       -0.49228        0.00000
C         -2.92787        0.91806        0.00001
C         -1.80583        1.75012        0.00000
C         -0.56571        1.12436       -0.00000
C         -0.41585       -0.27328       -0.00001
C         -1.55828       -1.08584       -0.00001
H         -3.91698        1.37114        0.00001
H         -1.89590        2.83222       -0.00000
H         -1.46048       -2.16890       -0.00002
C          1.60796        0.77098        0.00000
C          2.98126        0.97855        0.00000
C          3.78777       -0.16463        0.00000
C          3.22697       -1.45612        0.00000
C          1.84265       -1.64039        0.00000
C          1.01690       -0.50852        0.00001
H          3.40171        1.97960       -0.00001
H          4.86858       -0.05064       -0.00000
H          3.88386       -2.32199        0.00001
H          1.41430       -2.63922        0.00000
O          0.65589        1.76729       -0.00001
C         -4.08113       -1.33457        0.00000
H         -3.84601       -2.40446       -0.00007
H         -4.69969       -1.12734        0.88299
H         -4.69973       -1.12726       -0.88293
```

```
24
10m_3.log        Energy: -361883.0093645
C        -3.54194       0.99459      -0.00003
C        -3.81596      -0.38616      -0.00002
C        -2.78421      -1.33167      -0.00000
C        -1.48688      -0.83686       0.00000
C        -1.18052       0.53870       0.00000
C        -2.22806       1.46883      -0.00002
H        -4.36707       1.70210      -0.00004
H        -4.84795      -0.72718      -0.00002
H        -2.98225      -2.39928       0.00002
H        -2.02186       2.53592      -0.00002
C         0.71394      -0.72266       0.00002
C         2.05498      -1.08609      -0.00000
C         3.00615      -0.05685      -0.00000
C         2.57551       1.29068       0.00002
C         1.22512       1.63640       0.00003
C         0.26938       0.61120       0.00003
H         2.35086      -2.13145      -0.00000
H         3.32584       2.07856       0.00002
H         0.92110       2.67996       0.00004
O        -0.34359      -1.60818       0.00004
C         4.48359      -0.37361      -0.00005
H         4.97962       0.05089       0.88256
H         4.66434      -1.45381       0.00002
H         4.97953       0.05078      -0.88276
24
10m_4.log        Energy: -361883.9737938
C        -3.47714       0.76884      -0.00014
C        -3.58025      -0.63529      -0.00015
C        -2.44170      -1.44836      -0.00010
C        -1.21403      -0.79860      -0.00005
C        -1.07822       0.60368      -0.00005
C        -2.23112       1.39981      -0.00009
H        -4.38310       1.36931      -0.00018
H        -4.56300      -1.09944      -0.00019
H        -2.50875      -2.53209      -0.00011
H        -2.15648       2.48412      -0.00009
C         0.95756      -0.41430       0.00004
C         2.33546      -0.63138       0.00009
C         3.11525       0.53378       0.00013
C         2.54528       1.82147       0.00011
C         1.16163       1.99944       0.00006
C         0.35192       0.85562       0.00002
H         4.19834       0.43333       0.00017
H         3.19964       2.68944       0.00014
H         0.72388       2.99395       0.00004
O         0.01408      -1.42366       0.00000
C         2.91795      -2.01966       0.00012
H         2.59448      -2.58750       0.88197
H         2.59452      -2.58751      -0.88174
H         4.01229      -1.98467       0.00014
27
11m_1.log        Energy: -363267.6982415
C         3.95541      -0.46281       0.00000
C         2.86973      -1.30342       0.00000
C         1.53693      -0.78060       0.00000
C         1.35207       0.65184       0.00000
C         2.51049       1.49319       0.00000
C         3.77338       0.95387       0.00000
C         0.40534      -1.60817       0.00000
C         0.04996       1.17651       0.00000
C        -1.08543       0.35204       0.00000
C        -0.89526      -1.08442       0.00000
C        -2.04295      -1.94001       0.00000
H        -1.89201      -3.01729       0.00000
C        -3.30487      -1.40565       0.00000
C        -3.49172       0.00885       0.00000
C        -2.42901       0.88445       0.00000
H         0.53932      -2.68858       0.00000
H         4.96276      -0.87166       0.00000
H         3.00566      -2.38289       0.00000
H         2.36984       2.57210       0.00000
H         4.64475       1.60410       0.00000
H        -0.06536       2.25653       0.00000
H        -4.17643      -2.05582       0.00000
H        -4.50596       0.40236       0.00000
C        -2.65848       2.37562       0.00000
H        -2.21363       2.85386       0.88287
H        -2.21363       2.85386      -0.88287
H        -3.72895       2.60576       0.00000
27
11m_2.log        Energy: -363268.2218417
C        -4.13201       0.47092       0.00001
C        -3.01496       1.26960       0.00001
C        -1.70237       0.69817       0.00000
C        -1.57198      -0.74232      -0.00001
C        -2.76219      -1.53843      -0.00001
C        -4.00365      -0.95146       0.00000
C        -0.54261       1.48815      -0.00000
C        -0.29030      -1.31389      -0.00001
C         0.86811      -0.52415      -0.00001
C         0.73589       0.91506      -0.00001
C         1.93090       1.70474      -0.00001
H         1.83921       2.78900      -0.00001
C         3.16663       1.11250      -0.00001
C         3.31341      -0.31593      -0.00000
C         2.18147      -1.09637      -0.00001
H        -0.63889       2.57258       0.00001
H        -5.12314       0.91772       0.00002
H        -3.11015       2.35347       0.00002
H        -2.66301      -2.62197      -0.00001
H        -4.89917      -1.56813       0.00000
H        -0.19375      -2.39829      -0.00001
H         4.06324       1.72945      -0.00001
H         2.26883      -2.18158       0.00000
C         4.69904      -0.91152       0.00002
H         5.26791      -0.58802       0.88234
H         4.66858      -2.00650       0.00007
H         5.26794      -0.58807      -0.88230
27
11m_9.log        Energy: -363264.5297649
C         3.65200      -1.00585      -0.00001
C         2.45221      -1.67102       0.00001
C         1.21077      -0.95734       0.00000
C         1.22199       0.48827      -0.00003
C         2.50132       1.13838      -0.00005
C         3.67264       0.41937      -0.00005
C        -0.01350      -1.63500       0.00003
C         0.00241       1.21361      -0.00001
C        -1.22892       0.51053      -0.00001
C        -1.22710      -0.94071       0.00002
C        -2.47281      -1.64912       0.00003
H        -2.44470      -2.73693       0.00006
C        -3.66994      -0.98201       0.00000
C        -3.68378       0.44374      -0.00006
C        -2.51157       1.15900      -0.00007
H        -0.02160      -2.72351       0.00009
H         4.58814      -1.55868       0.00002
H         2.42166      -2.75877       0.00005
H         2.55159       2.22178      -0.00007
H         4.62689       0.94076      -0.00009
H        -4.60801      -1.53150       0.00003
H        -4.63553       0.96964      -0.00011
H        -2.56839       2.24143      -0.00014
C         0.06319       2.72547       0.00012
H         0.59820       3.09849      -0.88283
H        -0.91996       3.19509       0.00021
H         0.59826       3.09829       0.88314
27
12m_1.log        Energy: -363272.2932756
C        -3.14122       1.01387       0.00000
C        -2.65295      -0.28502       0.00000
C        -1.23825      -0.49480       0.00000
C        -0.34592       0.62422       0.00000
C        -0.89898       1.92837       0.00000
C        -2.26850       2.11871       0.00000
C        -0.68864      -1.82425       0.00000
C         1.09610       0.39201       0.00000
C         1.58700      -0.94863       0.00000
C         0.65729      -2.04047       0.00000
C         2.98375      -1.18890       0.00000
H         3.33580      -2.21838       0.00000
C         3.88660      -0.14170       0.00000
C         3.40963       1.18535       0.00000
C         2.04817       1.44295       0.00000
H        -1.36068      -2.67598       0.00000
H        -4.21606       1.18106       0.00000
H        -0.25117       2.79807       0.00000
H        -2.67511       3.12710       0.00000
H         1.04941      -3.05530       0.00000
H         4.95587      -0.33799       0.00000
H         4.11374       2.01387       0.00000
H         1.71987       2.47674       0.00000
C        -3.61235      -1.45282       0.00000
H        -3.47629      -2.09018      -0.88330
H        -3.47629      -2.09018       0.88330
H        -4.64949      -1.10212       0.00000
27
12m_2.log        Energy: -363273.3930782
C         3.17551      -0.20617      -0.00000
C         2.40616       0.94666      -0.00002
C         0.98948       0.90483      -0.00003
C         0.31723      -0.35349      -0.00003
```

```
C          1.11869       -1.52244       -0.00004
C          2.50009       -1.45134       -0.00002
C          0.22312        2.12042       -0.00002
C         -1.14010       -0.38378       -0.00002
C         -1.85955        0.85122       -0.00001
C         -1.13941        2.09454       -0.00000
C         -3.27629        0.83735        0.00003
H         -3.80704        1.78725        0.00005
C         -3.97852       -0.35453        0.00003
C         -3.27406       -1.57616        0.00002
C         -1.88844       -1.58752       -0.00001
H          0.75630        3.06868       -0.00002
H          2.89314        1.92034       -0.00002
H          0.65249       -2.50227       -0.00005
H          3.08210       -2.37116       -0.00001
H         -1.70861        3.02172       -0.00001
H         -5.06571       -0.35139        0.00006
H         -3.81967       -2.51666        0.00003
H         -1.37828       -2.54529       -0.00002
C          4.68413       -0.16035        0.00007
H          5.05631        0.86982        0.00017
H          5.09531       -0.66832        0.88252
H          5.09540       -0.66814       -0.88245
27
12m_3.log        Energy: -363273.2426259
C         -3.19732        0.63382        0.00229
C         -2.24225        1.63097        0.00167
C         -0.85936        1.32127        0.00144
C         -0.45345       -0.04629        0.00318
C         -1.46250       -1.04200        0.00492
C         -2.81541       -0.72997        0.00319
C          0.12828        2.36369       -0.00076
C          0.97178       -0.36011        0.00225
C          1.91881        0.71067       -0.00111
C          1.45946        2.07177       -0.00222
C          3.30564        0.41882       -0.00336
H          4.01208        1.24649       -0.00605
C          3.76105       -0.88719       -0.00233
C          2.83149       -1.94751        0.00125
C          1.47066       -1.68649        0.00344
H         -0.21050        3.39763       -0.00169
H         -4.25449        0.89224        0.00215
H         -2.54389        2.67651        0.00146
H         -1.18254       -2.09081        0.00642
H          2.20051        2.86822       -0.00440
H          4.82784       -1.09673       -0.00422
H          3.18217       -2.97667        0.00214
H          0.78277       -2.52537        0.00597
C         -3.87346       -1.80696       -0.00777
H         -3.43367       -2.80744        0.06704
H         -4.57555       -1.68284        0.82696
H         -4.46525       -1.76826       -0.93235
27
12m_4.log        Energy: -363266.4614957
C          3.49617       -0.32030       -0.12635
C          2.63424       -1.39678       -0.12308
C          1.23611       -1.19755       -0.04887
C          0.67393        0.12540       -0.01410
C          1.59537        1.22708        0.06233
C          2.96581        0.97366       -0.00937
C          0.39164       -2.35772        0.02491
C         -0.79305        0.23385       -0.03167
C         -1.58602       -0.95682        0.08944
C         -0.95739       -2.24496        0.13128
C         -2.99958       -0.87891        0.11986
H         -3.56442       -1.80222        0.23143
C         -3.65688        0.33012       -0.01093
C         -2.89681        1.49747       -0.20243
C         -1.51038        1.44654       -0.21457
H          0.86545       -3.33680        0.01653
H          4.57178       -0.46663       -0.18521
H          3.01515       -2.41470       -0.16378
H          3.65192        1.81580        0.04682
H         -1.58571       -3.12869        0.21695
H         -4.74289        0.37487        0.01011
H         -3.39446        2.45277       -0.35126
H         -0.98665        2.36903       -0.40147
C          1.20806        2.67629        0.27984
H          0.80747        3.14814       -0.62607
H          0.46388        2.79082        1.07527
H          2.09108        3.25340        0.57286
27
12m_9.log        Energy: -363272.7788902
C         -2.10983       -2.78653        0.00000
C         -2.39414       -1.43139        0.00000
C         -1.36271       -0.45773        0.00000
C          0.00000       -0.89566        0.00000
```

```
C          0.25668       -2.28871        0.00000
C         -0.77039       -3.21797        0.00000
C         -1.67369        0.96013        0.00000
C          1.07546        0.09041        0.00000
C          0.73104        1.47368        0.00000
C         -0.64930        1.86412        0.00000
C          1.75558        2.45241        0.00000
H          1.47330        3.50330        0.00000
C          3.09004        2.08760        0.00000
C          3.43677        0.72107        0.00000
C          2.45020       -0.25266        0.00000
H         -2.91832       -3.51353        0.00000
H         -3.43089       -1.11082        0.00000
H          1.27951       -2.64935        0.00000
H         -0.53842       -4.28021        0.00000
H         -0.87252        2.92963        0.00000
H          3.86697        2.84818        0.00000
H          4.48347        0.42686        0.00000
H          2.75409       -1.29432        0.00000
C         -3.10816        1.42843        0.00000
H         -3.65201        1.06756       -0.88296
H         -3.65201        1.06756        0.88296
H         -3.15850        2.52203        0.00000
28
13m_1.log        Energy: -374092.3950486
C          3.51312       -1.00014        0.01453
C          3.64614        0.40294        0.02231
C          2.53084        1.24211        0.01016
C          1.26192        0.64470       -0.01492
C          1.11492       -0.76740       -0.01174
C          2.24945       -1.58994       -0.00091
H          4.40314       -1.62413        0.02519
H          4.63924        0.84527        0.04202
H          2.65517        2.32066        0.02629
H          2.14282       -2.67216       -0.00117
C         -0.96790        0.23117       -0.02016
C         -2.37971        0.31324        0.00585
C         -3.06712       -0.90724        0.01562
C         -2.42207       -2.15816        0.00389
C         -1.03361       -2.22759       -0.00961
C         -0.30446       -1.03165       -0.01626
H         -4.15474       -0.88079        0.03782
H         -3.01701       -3.06798        0.01272
H         -0.51859       -3.18507       -0.00933
C         -0.19478        2.67728       -0.00432
H         -0.36188        3.03421        1.01961
H          0.69322        3.16982       -0.40596
H         -1.03874        2.96320       -0.63046
N          0.00524        1.23689       -0.04397
C         -3.16706        1.60575        0.02348
H         -2.83671        2.28374        0.81827
H         -3.09217        2.14873       -0.92731
H         -4.22856        1.39579        0.19257
28
13m_2.log        Energy: -374094.9547871
C          2.60842       -1.52121        0.00412
C          3.04889       -0.17584        0.00514
C          2.11020        0.86065       -0.00829
C          0.74702        0.53496       -0.02595
C          0.30440       -0.81481       -0.01671
C          1.25387       -1.84548       -0.00524
H          3.35024       -2.31724        0.01486
H          2.44012        1.89601       -0.00013
H          0.93697       -2.88588       -0.00038
C         -1.50866        0.59991       -0.02090
C         -2.85180        0.99687        0.00178
C         -3.82633       -0.00342        0.01927
C         -3.47856       -1.36873        0.01792
C         -2.13895       -1.75976        0.00303
C         -1.14179       -0.77599       -0.01314
H         -3.13479        2.04537        0.01048
H         -4.87580        0.28075        0.03709
H         -4.26192       -2.12233        0.03220
H         -1.87059       -2.81366        0.00696
C         -0.31401        2.82444        0.02750
H         -0.27799        3.16856        1.06990
H          0.56761        3.19699       -0.50072
H         -1.19840        3.24697       -0.45600
N         -0.35738        1.37689       -0.05218
C          4.53009        0.12622        0.02293
H          4.72290        1.20478        0.02753
H          5.01355       -0.30478        0.90964
H          5.03301       -0.30062       -0.85499
28
13m_3.log        Energy: -374094.5390006
C          3.00494       -1.96185        0.00997
C          3.68841       -0.72918        0.01241
```

```
C          3.00065        0.48563        0.00111
C          1.60030        0.44358       -0.01723
C          0.89523       -0.79466       -0.01075
C          1.61034       -1.99973       -0.00075
H          3.57145       -2.88955        0.01981
H          4.77576       -0.72113        0.02607
H          3.54014        1.42818        0.00887
H          1.08310       -2.95086        0.00201
C         -0.59645        0.95127       -0.02093
C         -1.83615        1.60285       -0.00658
C         -2.98555        0.81330        0.00391
C         -2.93535       -0.60079        0.00359
C         -1.68881       -1.23222       -0.00374
C         -0.51404       -0.46609       -0.01238
H         -1.90839        2.68670        0.00037
H         -3.95779        1.30264        0.01554
H         -1.62926       -2.31874        0.00051
C          1.00197        2.89963        0.03476
H          0.98715        3.25629        1.07339
H          1.99354        3.07821       -0.38803
H          0.27693        3.47379       -0.54931
N          0.68565        1.48693       -0.04513
C         -4.21982       -1.39972        0.01522
H         -4.02001       -2.47715        0.01637
H         -4.82647       -1.17239        0.90216
H         -4.83967       -1.17661       -0.86355
28
13m_4.log        Energy: -374095.7363075
C          2.91650       -1.66424        0.00813
C          3.51341       -0.38909        0.01268
C          2.73981        0.77267        0.00297
C          1.34671        0.63120       -0.01573
C          0.72223       -0.65129       -0.01044
C          1.52760       -1.80160       -0.00226
H          3.54481       -2.55130        0.01635
H          4.59738       -0.30433        0.02679
H          3.20906        1.75207        0.01321
H          1.08108       -2.79033       -0.00110
C         -0.87738        1.00112       -0.01738
C         -2.14437        1.59786        0.00101
C         -3.24940        0.74796        0.01173
C         -3.09853       -0.65148        0.00847
C         -1.83754       -1.25814       -0.00120
C         -0.70854       -0.41271       -0.01084
H         -2.26642        2.67684        0.01063
H         -4.24943        1.17477        0.02578
H         -3.98606       -1.28034        0.01789
C          0.59393        3.04411        0.02291
H          0.53886        3.41216        1.05597
H          1.57892        3.27863       -0.38671
H         -0.15489        3.56435       -0.58130
N          0.36752        1.61296       -0.04163
C         -1.69504       -2.75919        0.00326
H         -1.14665       -3.11216       -0.88010
H         -1.14072       -3.10563        0.88561
H         -2.67490       -3.24869        0.00860
27
14m_2.log        Energy: -363225.4606551
C          0.00000       -0.51188        0.00000
C          0.51775        0.59104        0.00000
C         -0.61827       -1.79718        0.00000
C         -2.03367       -1.91113        0.00000
C          0.18228       -2.95869        0.00000
C         -2.59412       -3.19347        0.00000
C         -0.40235       -4.22379        0.00000
H          1.26379       -2.85347        0.00000
C         -1.79578       -4.34203        0.00000
H         -3.67730       -3.29355        0.00000
H          0.22557       -5.11109        0.00000
H         -2.26163       -5.32439        0.00000
C          1.12460        1.88189        0.00000
C          0.32729        3.04672        0.00000
C          2.52965        2.01410        0.00000
C          0.92398        4.30725        0.00000
H         -0.75497        2.95085        0.00000
C          3.11672        3.27931        0.00000
H          3.14863        1.12125        0.00000
C          2.31851        4.42919        0.00000
H          0.29924        5.19705        0.00000
H          4.20023        3.36814        0.00000
H          2.77982        5.41347        0.00000
C         -2.90357       -0.67986        0.00000
H         -2.70799       -0.05522        0.88111
H         -2.70799       -0.05522       -0.88111
H         -3.96546       -0.94673        0.00000
27
14m_3.log        Energy: -363224.8283421
C          0.19812       -0.23628       -0.00155
C         -1.01501       -0.12428       -0.00109
C          1.61927       -0.36447       -0.00033
C          2.43659        0.78561       -0.01331
C          2.22314       -1.63856        0.01058
C          3.83160        0.68508       -0.01348
C          3.61357       -1.74278        0.00824
H          1.60049       -2.52866        0.01742
C          4.41125       -0.59445       -0.00427
H          4.07934       -2.72525        0.01280
H          5.49503       -0.69044       -0.00891
C         -2.43616        0.00093       -0.00056
C         -3.04419        1.27464        0.00552
C         -3.25523       -1.14863       -0.00584
C         -4.43408        1.39010        0.00644
H         -2.41900        2.16328        0.00977
C         -4.64410       -1.02243       -0.00497
H         -2.79302       -2.13182       -0.01067
C         -5.23887        0.24477        0.00119
H         -4.89047        2.37681        0.01133
H         -5.26401       -1.91556       -0.00918
H         -6.32180        0.33905        0.00192
C          4.69611        1.92413        0.00500
H          4.17043        2.78822       -0.41639
H          4.98493        2.18371        1.03284
H          5.62026        1.77483       -0.56520
H          1.96573        1.76557       -0.02511
27
14m_4.log        Energy: -363225.0679948
C         -0.09009        0.00081       -0.00170
C          1.12835       -0.00041       -0.00049
C         -1.51607        0.00223       -0.00246
C         -2.23670        1.21381       -0.00433
C         -2.23916       -1.21045       -0.00366
C         -3.63115        1.20637       -0.00692
C         -3.63128       -1.20140       -0.00611
H         -1.69941       -2.15361       -0.00464
C         -4.35463        0.00389       -0.00664
H         -4.16948       -2.14732       -0.00829
C          2.55490       -0.00068        0.00048
C          3.27185        1.21538        0.00138
C          3.27173       -1.21679        0.00053
C          4.66648        1.20988        0.00233
H          2.72681        2.15533        0.00136
C          4.66637       -1.21140        0.00147
H          2.72663       -2.15670       -0.00018
C          5.36908       -0.00079        0.00237
H          5.20661        2.15342        0.00304
H          5.20643       -2.15498        0.00148
H          6.45620       -0.00084        0.00309
H         -1.69680        2.15686       -0.00590
H         -4.16717        2.15309       -0.00998
C         -5.86370       -0.00506        0.01438
H         -6.26993       -0.71421       -0.71742
H         -6.24052       -0.30911        1.00072
H         -6.27522        0.98562       -0.20710
24
15m_1.log        Energy: -564554.2780841
C         -2.95638        1.50947       -0.00014
C         -3.59490        0.25933       -0.00015
C         -2.84022       -0.91247       -0.00011
C         -1.44590       -0.81169       -0.00005
C         -0.77235        0.43789       -0.00004
C         -1.56576        1.60330       -0.00009
H         -3.55113        2.41934       -0.00018
H         -4.68030        0.20106       -0.00019
H         -3.32508       -1.88522       -0.00011
H         -1.10971        2.58417       -0.00008
C          1.05288       -1.09278        0.00005
C          2.38384       -1.51864        0.00011
C          3.37963       -0.54683        0.00015
C          3.04122        0.81443        0.00012
C          1.71357        1.25492        0.00006
C          0.68398        0.27759        0.00003
H          2.63299       -2.57629        0.00013
H          4.42555       -0.84315        0.00019
H          3.83642        1.55636        0.00015
C          1.43256        2.73765        0.00004
H          0.85985        3.04208       -0.88485
H          0.85981        3.04211        0.88488
H          2.36868        3.30525        0.00005
S         -0.33688       -2.18242        0.00001
24
15m_2.log        Energy: -564555.3900208
C         -2.88059       -0.66334        0.00001
C         -3.09086        0.73368       -0.00000
C         -2.02918        1.63518       -0.00001
```

```
C         -0.72418         1.13094         0.00000
C         -0.48056        -0.25948         0.00001
C         -1.57138        -1.14533         0.00002
H         -4.10992         1.11522        -0.00001
H         -2.21600         2.70587        -0.00002
H         -1.38849        -2.21763         0.00003
C          1.74129         0.59921        -0.00000
C          3.13817         0.52987        -0.00002
C          3.74135        -0.72820        -0.00002
C          2.96188        -1.89911         0.00000
C          1.57100        -1.82357         0.00001
C          0.94139        -0.56805         0.00001
H          3.74174         1.43346        -0.00002
H          4.82594        -0.80066        -0.00002
H          3.44945        -2.87053        -0.00000
H          0.97033        -2.72975         0.00002
C         -4.06384        -1.60293        -0.00002
H         -3.74535        -2.65099         0.00011
H         -4.69643        -1.44337         0.88288
H         -4.69622        -1.44354        -0.88311
S          0.77112         2.07929         0.00001
24
15m_3.log         Energy: -564555.4949580
C         -3.40090         1.42931         0.00021
C         -3.90337         0.11580         0.00024
C         -3.03749        -0.97845         0.00012
C         -1.65995        -0.73769        -0.00003
C         -1.13637         0.57675        -0.00008
C         -2.02735         1.66249         0.00006
H         -4.09079         2.26924         0.00033
H         -4.97717        -0.05264         0.00038
H         -3.42713        -1.99290         0.00017
H         -1.64232         2.67903         0.00008
C          0.86504        -0.71370        -0.00017
C          2.24686        -0.93326        -0.00015
C          3.11537         0.16201        -0.00011
C          2.57173         1.46616        -0.00026
C          1.19845         1.68309        -0.00031
C          0.31814         0.58786        -0.00020
H          2.64500        -1.94502        -0.00010
H          3.24840         2.31813        -0.00030
H          0.80469         2.69654        -0.00044
C          4.61321        -0.03186         0.00057
H          5.07179         0.42912         0.88519
H          4.88336        -1.09334        -0.00241
H          5.07355         0.43479        -0.88011
S         -0.38560        -1.96713        -0.00014
24
15m_4.log         Energy: -564556.7181478
C         -3.47149         1.05703        -0.00015
C         -3.74879        -0.32196        -0.00015
C         -2.71345        -1.25720        -0.00011
C         -1.39447        -0.79145        -0.00005
C         -1.09735         0.59108        -0.00004
C         -2.15587         1.51423        -0.00010
H         -4.29064         1.77148        -0.00019
H         -4.77989        -0.66608        -0.00019
H         -2.92993        -2.32218        -0.00011
H         -1.94307         2.58032        -0.00009
C          1.09193        -0.34666         0.00005
C          2.49696        -0.35052         0.00010
C          3.12808         0.89817         0.00013
C          2.39611         2.09832         0.00010
C          1.00458         2.08101         0.00005
C          0.33492         0.84698         0.00002
H          4.21525         0.93520         0.00017
H          2.92556         3.04771         0.00012
H          0.43765         3.00831         0.00003
C          3.27587        -1.64079         0.00012
H          3.03970        -2.24969         0.88360
H          3.03973        -2.24970        -0.88335
H          4.35426        -1.45230         0.00015
S          0.06558        -1.79203         0.00001
29
16m_1.log         Energy: -411113.7738937
C         -1.04743        -3.08961         0.00000
C         -1.94049        -2.01034         0.00000
C         -1.41776        -0.69264         0.00000
C          0.00000        -0.49106         0.00000
C          0.88545        -1.61401         0.00000
C          0.33486        -2.90470         0.00000
C         -2.26576         0.47182         0.00000
C          0.54068         0.83268         0.00000
C         -0.33137         1.96537         0.00000
C         -1.74956         1.73535         0.00000
C          0.22030         3.25955         0.00000
H         -0.44572         4.11971         0.00000
C          1.60362         3.44308         0.00000
C          2.46469         2.34341         0.00000
C          1.95788         1.03193         0.00000
C          2.81931        -0.12097         0.00000
C          2.30592        -1.38401         0.00000
H          2.96837        -2.24708         0.00000
H          3.89549         0.03745         0.00000
H         -3.34306         0.33982         0.00000
H         -1.44589        -4.10180         0.00000
H          0.99809        -3.76716         0.00000
H         -2.41406         2.59685         0.00000
H          2.01464         4.44955         0.00000
H          3.54194         2.49464         0.00000
C         -3.42951        -2.26355         0.00000
H         -3.91637        -1.82908         0.88304
H         -3.91637        -1.82908        -0.88304
H         -3.64207        -3.33763         0.00000
29
16m_2.log         Energy: -411114.4215287
C         -3.89942        -0.00313         0.00440
C         -3.20608        -1.21594         0.00316
C         -1.80046        -1.23894         0.00092
C         -1.08615        -0.00018         0.00013
C         -1.80307         1.23698         0.00104
C         -3.20931         1.21076         0.00331
C         -1.04794        -2.46688        -0.00088
C          0.34064         0.00152        -0.00163
C          1.06253        -1.23200        -0.00332
C          0.31623        -2.46405        -0.00342
C          2.46611        -1.20280        -0.00654
H          3.01182        -2.14516        -0.00912
C          3.17817         0.00519        -0.00713
C          2.46466         1.20953        -0.00683
C          1.05875         1.23544        -0.00343
C          0.31101         2.46557        -0.00341
C         -1.05369         2.46602        -0.00069
H         -1.60235         3.40546        -0.00013
H          0.86034         3.40463        -0.00505
H         -1.59492        -3.40731        -0.00047
H         -4.98673        -0.00454         0.00609
H         -3.75272        -2.15649         0.00382
H         -3.75787         2.15018         0.00414
H          0.86732        -3.40209        -0.00506
H          3.00701         2.15340        -0.00990
C          4.68909        -0.00235         0.01439
H          5.06631        -0.29558         1.00365
H          5.09604        -0.71750        -0.71099
H          5.09938         0.98655        -0.21740
29
16m_4.log         Energy: -411114.3968136
C          1.14060        -3.23573         0.00000
C          1.96691        -2.10880         0.00000
C          1.42539        -0.80991         0.00000
C          0.00000        -0.66575         0.00000
C         -0.83812        -1.82560         0.00000
C         -0.24666        -3.10102         0.00000
C          2.25849         0.38113         0.00000
C         -0.59529         0.63203         0.00000
C          0.23852         1.79041         0.00000
C          1.66459         1.61344         0.00000
C         -0.35942         3.06327         0.00000
H          0.27420         3.94769         0.00000
C         -1.74958         3.19764         0.00000
C         -2.57248         2.06978         0.00000
C         -2.01726         0.77741         0.00000
C         -2.83115        -0.40878         0.00000
C         -2.26697        -1.65039         0.00000
H         -2.89441        -2.53914         0.00000
H         -3.91300        -0.29486         0.00000
H          1.58688        -4.22715         0.00000
H          3.04324        -2.24638         0.00000
H         -0.88360        -3.98299         0.00000
H          2.28555         2.50752         0.00000
H         -2.19474         4.18956         0.00000
H         -3.65431         2.18290         0.00000
C          3.76152         0.25074         0.00000
H          4.11848        -0.29626         0.88272
H          4.11848        -0.29626        -0.88272
H          4.23869         1.23611         0.00000
29
17m_1.log         Energy: -411100.7627070
C         -2.51633         1.10492         0.00000
C         -1.29365         0.43106         0.00000
C         -1.27143        -1.00023         0.00000
C         -2.45845        -1.72878         0.00000
C         -3.67979        -1.03914         0.00000
C         -3.70639         0.36102         0.00000
```

H -2.55837 2.18944 0.00000
H -2.44107 -2.81627 0.00000
H -4.61315 -1.59677 0.00000
H -4.66138 0.88094 0.00000
C 0.10391 0.91061 0.00000
C 0.91742 -0.25342 0.00000
C 0.71494 2.15910 0.00000
C 0.13527 -1.43741 0.00000
C 2.32172 -0.24492 0.00000
C 2.14615 2.18223 0.00000
C 0.78231 -2.65919 0.00000
C 2.96518 -1.51658 0.00000
C 2.93322 1.04173 0.00000
H 2.63473 3.15435 0.00000
C 2.20702 -2.67927 0.00000
H 0.23227 -3.59727 0.00000
H 4.05152 -1.57263 0.00000
H 4.01748 1.12911 0.00000
H 2.71292 -3.64173 0.00000
C -0.06170 3.45144 0.00000
H -0.70975 3.52701 -0.88290
H -0.70975 3.52701 0.88290
H 0.60809 4.31779 0.00000
29
17m_2.log Energy: -411100.0015393
C -0.02682 2.93315 0.00000
C 0.16965 1.55426 0.00000
C 1.49298 1.01329 0.00000
C 2.59879 1.86068 0.00000
C 2.39036 3.24820 0.00000
C 1.09312 3.77823 0.00000
H -1.03102 3.35041 0.00000
H 3.60836 1.45655 0.00000
H 3.24537 3.91979 0.00000
H 0.95230 4.85625 0.00000
C -0.78500 0.42793 0.00000
C 0.00000 -0.75353 0.00000
C -2.15864 0.30552 0.00000
C 1.38647 -0.45953 0.00000
C -0.53878 -2.04845 0.00000
C -2.75476 -0.99820 0.00000
H -2.80705 1.17990 0.00000
C 2.28090 -1.51391 0.00000
C 0.40454 -3.11746 0.00000
C -1.96245 -2.14076 0.00000
C 1.76601 -2.84295 0.00000
H 3.35637 -1.35375 0.00000
H 0.05592 -4.14788 0.00000
H -2.43268 -3.12214 0.00000
H 2.47134 -3.67059 0.00000
C -4.26255 -1.09885 0.00000
H -4.69199 -0.60642 0.88256
H -4.69199 -0.60642 -0.88256
H -4.59776 -2.14147 0.00000
29
17m_3.log Energy: -411100.3996708
C -1.95055 -2.54682 0.00000
C -1.06140 -1.47439 0.00000
C 0.34875 -1.70563 0.00000
C 0.84759 -3.00659 0.00000
C -0.05587 -4.08006 0.00000
C -1.43881 -3.85307 0.00000
H -3.02475 -2.37722 0.00000
H 1.91939 -3.19091 0.00000
H 0.32178 -5.09961 0.00000
H -2.12331 -4.69779 0.00000
C -1.28607 -0.01588 0.00000
C 0.00000 0.58530 0.00000
C -2.39927 0.80334 0.00000
C 1.02561 -0.39516 0.00000
C 0.22586 1.97237 0.00000
C -2.20395 2.21357 0.00000
H -3.40873 0.39921 0.00000
C 2.33573 0.03861 0.00000
C 1.59475 2.40817 0.00000
C -0.94119 2.79204 0.00000
H -3.07921 2.85879 0.00000
C 2.59406 1.43971 0.00000
H 3.17103 -0.65786 0.00000
H -0.84837 3.87462 0.00000
H 3.63171 1.76692 0.00000
C 1.93071 3.87783 0.00000
H 1.51549 4.38296 -0.88225
H 1.51549 4.38296 0.88225
H 3.01381 4.03794 0.00000
29
17m_7.log Energy: -411100.5763449
C 1.91987 -2.08996 0.00000
C 0.90300 -1.13927 0.00000
C 1.20504 0.25809 0.00000
C 2.53423 0.70354 0.00000
C 3.54360 -0.27735 0.00000
C 3.24848 -1.64556 0.00000
H 1.69036 -3.15279 0.00000
H 4.58381 0.04190 0.00000
H 4.06018 -2.36914 0.00000
C -0.56284 -1.29655 0.00000
C -1.10231 0.01420 0.00000
C -1.43265 -2.37083 0.00000
C -0.07573 0.99810 0.00000
C -2.47815 0.29800 0.00000
C -2.83428 -2.11380 0.00000
H -1.07434 -3.39751 0.00000
C -0.45658 2.32944 0.00000
C -2.83712 1.67814 0.00000
C -3.35413 -0.82708 0.00000
H -3.51722 -2.95988 0.00000
C -1.84696 2.64841 0.00000
H 0.26747 3.13745 0.00000
H -3.88604 1.96597 0.00000
H -4.43084 -0.67291 0.00000
H -2.13401 3.69729 0.00000
C 2.88172 2.17100 0.00000
H 2.47147 2.67858 -0.88282
H 2.47147 2.67858 0.88282
H 3.96651 2.32139 0.00000
29
17m_8.log Energy: -411099.8376112
C 1.92652 -1.78203 -0.00359
C 0.81734 -0.94127 0.00109
C 1.00130 0.47503 -0.00067
C 2.28088 1.02134 -0.00719
C 3.40605 0.17478 -0.01010
C 3.21013 -1.21680 -0.01003
H 1.80646 -2.86296 -0.00645
H 2.41852 2.10106 -0.01260
H 4.07822 -1.87275 -0.01698
C -0.63173 -1.21725 0.00193
C -1.27468 0.04852 0.00121
C -1.41883 -2.35307 0.00270
C -0.33208 1.10942 -0.00078
C -2.66774 0.22865 0.00146
C -2.83697 -2.20253 0.00286
H -0.98575 -3.35045 0.00294
C -0.80366 2.40822 -0.00202
C -3.12676 1.57893 0.00013
C -3.45576 -0.96051 0.00231
H -3.45201 -3.09928 0.00328
C -2.21315 2.62361 -0.00152
H -0.13021 3.26199 -0.00339
H -4.19441 1.78741 0.00023
H -4.54125 -0.89094 0.00220
H -2.58049 3.64700 -0.00254
C 4.79935 0.75982 0.01420
H 4.85888 1.67934 -0.58025
H 5.10100 1.01697 1.03936
H 5.53973 0.05341 -0.37779
29
18m_10.log Energy: -411065.7089274
C -1.77717 1.35129 0.00001
C -0.41020 0.77772 0.00000
C -0.50499 -0.67175 -0.00000
C -1.93622 -0.97391 -0.00000
C -2.65767 0.30434 0.00000
C -2.85580 -1.98246 -0.00001
C -4.19316 -1.35913 -0.00000
C -4.08156 0.00822 0.00000
C 0.62862 -1.43794 -0.00000
C 1.91933 -0.80569 -0.00000
C 0.80445 1.40763 0.00000
C 2.00959 0.62732 0.00000
C 3.11630 -1.56208 -0.00001
C 4.35505 -0.93941 -0.00001
C 3.28770 1.23579 0.00000
C 4.44266 0.46947 0.00000
H -4.89369 0.72576 0.00001
H -5.12292 -1.91955 -0.00001
H -2.67534 -3.05238 -0.00001
H 0.57559 -2.52459 -0.00001
H 3.05022 -2.64817 -0.00001
H 5.26312 -1.53737 -0.00001
H 5.41679 0.95199 0.00000
H 3.35057 2.32212 0.00001
C -2.06009 2.81397 0.00001

```
H         -1.61398        3.29552        0.88110
H         -1.61399        3.29553       -0.88108
H         -3.13498        3.01770        0.00001
H          0.87753        2.49327        0.00001
29
18m_1.log         Energy: -411063.0731109
C          1.54869       -1.46044        0.00001
C          0.18416       -0.90639        0.00003
C          0.27476        0.54637        0.00003
C          1.70385        0.85467       -0.00001
C          2.43144       -0.42442        0.00001
C          2.62603        1.85782       -0.00014
C          3.96704        1.23287       -0.00029
C          3.86842       -0.13383       -0.00008
H          1.77076       -2.52359       -0.00002
H          2.44883        2.92857       -0.00022
H          4.89760        1.79289       -0.00044
C         -0.86338        1.30743        0.00006
C         -2.15094        0.67064        0.00006
H         -0.81502        2.39436        0.00008
C         -1.03022       -1.53974        0.00001
C         -2.23652       -0.76318        0.00001
H         -1.09862       -2.62575       -0.00003
C         -3.35072        1.42320        0.00010
C         -4.58725        0.79677        0.00007
H         -3.28805        2.50953        0.00015
H         -5.49726        1.39177        0.00010
C         -3.51335       -1.37540       -0.00003
C         -4.67036       -0.61275       -0.00000
H         -3.57287       -2.46192       -0.00008
H         -5.64304       -1.09823       -0.00004
C          4.96866       -1.14736        0.00014
H          4.91268       -1.79985        0.88277
H          4.91148       -1.80161       -0.88109
H          5.95164       -0.66377       -0.00100
29
18m_2.log         Energy: -411063.7739587
C         -1.26065        1.91500       -0.00003
C          0.00822        1.16583       -0.00003
C         -0.29515       -0.25683       -0.00001
C         -1.75550       -0.35147       -0.00000
C         -2.29043        1.02391        0.00004
C         -2.80883       -1.21148       -0.00005
C         -4.05926       -0.40448        0.00001
C         -3.74220        0.92907        0.00008
H         -1.32204        2.99922       -0.00003
H         -2.79104       -2.29717       -0.00019
C          0.71391       -1.17994        0.00001
C          2.08345       -0.74362        0.00001
H          0.50301       -2.24731        0.00001
C          1.30310        1.60983       -0.00003
C          2.38132        0.66110       -0.00001
H          1.53402        2.67330       -0.00004
C          3.15566       -1.66739        0.00003
C          4.47276       -1.23404        0.00002
H          2.93011       -2.73186        0.00004
H          5.28271       -1.95938        0.00003
C          3.73497        1.07457       -0.00001
C          4.76519        0.14666        0.00001
H          3.95732        2.13982       -0.00002
H          5.79952        0.48141        0.00001
C         -5.42360       -1.01743       -0.00001
H         -5.56934       -1.65795        0.88148
H         -6.20759       -0.25250        0.00006
H         -5.56938       -1.65785       -0.88156
H         -4.44494        1.75462        0.00017
29
18m_3.log         Energy: -411064.0916730
C         -1.39825       -2.08003       -0.00005
C         -0.15401       -1.29476       -0.00004
C         -0.49856        0.12007       -0.00009
C         -1.95996        0.17160       -0.00004
C         -2.44983       -1.21419       -0.00003
C         -3.03460        1.01447        0.00004
C         -4.24995        0.15575        0.00009
C         -3.90835       -1.16664        0.00003
H         -1.43082       -3.16550       -0.00006
C          0.49015        1.06812       -0.00006
C          1.86885        0.66681       -0.00002
H          0.25561        2.13000       -0.00004
C          1.15351       -1.70483        0.00001
C          2.20484       -0.72986        0.00001
H          1.41104       -2.76224        0.00004
C          2.91834        1.61803       -0.00000
C          4.24543        1.21898        0.00002
H          2.66584        2.67647       -0.00003
H          5.03637        1.96511        0.00002
C          3.56984       -1.10804        0.00004
C          4.57492       -0.15442        0.00005
H          3.81934       -2.16725        0.00006
H          5.61780       -0.46144        0.00007
H         -4.58258       -2.01528        0.00006
H         -5.26023        0.55500        0.00014
C         -3.07728        2.50755        0.00000
H         -3.61502        2.88567        0.88129
H         -2.07295        2.94218        0.00005
H         -3.61486        2.88562       -0.88140
29
18m_4.log         Energy: -411061.9068469
C         -1.81897       -1.89598        0.00005
C         -0.50499       -1.23830        0.00004
C         -0.70024        0.20400        0.00008
C         -2.15598        0.40135        0.00004
C         -2.78229       -0.93403        0.00002
C         -3.15956        1.32736       -0.00004
C         -4.44538        0.59799       -0.00007
C         -4.22701       -0.75293       -0.00006
H         -1.95701       -2.97312        0.00005
C          0.36934        1.06671        0.00007
C          1.70731        0.50069        0.00003
C          0.74628       -1.78749        0.00000
C          1.89133       -0.92400       -0.00000
H          0.89160       -2.86572       -0.00003
C          2.86394        1.31838       -0.00002
C          4.13890        0.76919       -0.00004
H          2.75863        2.39859       -0.00004
H          5.00710        1.42372       -0.00006
C          3.20200       -1.45715       -0.00003
C          4.31289       -0.62863       -0.00004
H          3.32525       -2.53820       -0.00004
H          5.31307       -1.05427       -0.00006
H         -4.97589       -1.53621       -0.00010
H         -5.41511        1.08592       -0.00014
C          0.18271        2.56159        0.00003
H         -0.87489        2.83107        0.00015
H          0.64366        3.02359        0.88315
H          0.64346        3.02346       -0.88327
H         -3.08151        2.40872       -0.00002
29
18m_5.log         Energy: -411061.5504291
C          2.16706       -1.74570        0.00004
C          0.79512       -1.21069        0.00001
C          0.86321        0.23950        0.00001
C          2.28873        0.57121        0.00005
C          3.03735       -0.69722        0.00006
C          3.18912        1.59422        0.00008
C          4.54199        0.99506        0.00011
C          4.45793       -0.37133        0.00010
H          2.40445       -2.80547        0.00004
C         -0.28232        0.98868       -0.00001
C         -1.56969        0.34547       -0.00004
C         -0.41309       -1.85367       -0.00002
C         -1.62920       -1.09063       -0.00004
H         -0.47285       -2.94022       -0.00001
C         -2.78538        1.09401       -0.00006
C         -3.99776        0.41151       -0.00008
H         -4.92488        0.98084       -0.00009
C         -2.88770       -1.73775       -0.00005
C         -4.05615       -0.99699       -0.00007
H         -2.92129       -2.82517       -0.00005
H         -5.02191       -1.49632       -0.00008
H          5.28159       -1.07553        0.00011
H          5.45975        1.57475        0.00014
H          2.99057        2.66091        0.00008
H         -0.22260        2.07255       -0.00001
C         -2.76681        2.60450       -0.00007
H         -2.25083        3.00368        0.88299
H         -2.25082        3.00367       -0.88313
H         -3.78555        3.00522       -0.00008
29
18m_6.log         Energy: -411062.2724980
C         -2.35633        1.64580        0.00002
C         -0.98472        1.11242        0.00001
C         -1.05382       -0.34029        0.00003
C         -2.47981       -0.67061        0.00006
C         -3.22760        0.59758        0.00007
C         -3.38063       -1.69354       -0.00005
C         -4.73297       -1.09408       -0.00006
C         -4.64803        0.27253        0.00000
H         -2.59386        2.70560       -0.00002
C          0.09266       -1.08701        0.00003
C          1.37286       -0.43312        0.00001
C          0.22182        1.76150       -0.00001
C          1.43671        0.99925       -0.00002
```

H 0.27693 2.84833 -0.00004
C 2.58045 -1.17194 0.00002
C 3.82325 -0.55060 -0.00000
C 2.70958 1.61931 -0.00003
C 3.87046 0.86643 -0.00003
H 2.76400 2.70619 -0.00005
H 4.83774 1.36507 -0.00004
H -5.47141 0.97712 0.00002
H -5.65107 -1.67326 -0.00013
H -3.18215 -2.76026 -0.00012
H 0.05770 -2.17449 0.00006
C 5.10553 -1.34719 0.00001
H 5.71622 -1.11436 0.88252
H 4.91031 -2.42500 0.00006
H 5.71618 -1.11444 -0.88255
H 2.52307 -2.25923 0.00004
29
18m_7.log Energy: -411062.1146153
C 2.14921 -1.72982 -0.00002
C 0.86348 -1.01235 0.00003
C 1.13202 0.41664 -0.00001
C 2.58991 0.54784 -0.00006
C 3.15641 -0.81225 -0.00009
C 3.62379 1.43567 0.00006
C 4.88066 0.65497 0.00005
C 4.60862 -0.68653 -0.00005
H 2.23864 -2.81213 0.00002
C 0.09721 1.31164 -0.00003
C -1.25850 0.83676 -0.00001
C -0.41959 -1.49208 0.00006
C -1.52184 -0.57271 0.00004
H -0.62089 -2.56158 0.00009
C -2.36146 1.72417 -0.00001
C -3.66147 1.24908 0.00001
C -2.86392 -1.02436 0.00006
C -3.93616 -0.14185 0.00004
H -3.04927 -2.09737 0.00010
H 5.32751 -1.49740 -0.00010
H 5.86956 1.10267 0.00011
H 3.57555 2.51966 0.00016
H 0.27858 2.38443 -0.00006
H -2.17307 2.79588 -0.00003
H -4.49227 1.95228 -0.00000
C -5.36519 -0.62789 -0.00006
H -5.41896 -1.72198 0.00023
H -5.90737 -0.26282 0.88227
H -5.90704 -0.26332 -0.88281
29
18m_8.log Energy: -411061.4899598
C 1.89742 1.69388 0.00008
C 0.65269 0.90792 0.00003
C 0.99722 -0.50228 0.00001
C 2.46007 -0.55677 0.00004
C 2.95270 0.83139 0.00009
C 3.53958 -1.38858 0.00005
C 4.75296 -0.54209 0.00010
C 4.40944 0.78307 0.00012
H 1.92809 2.77956 0.00011
C 0.00797 -1.44568 -0.00004
C -1.37210 -1.04354 -0.00006
C -0.65398 1.32081 0.00001
C -1.71562 0.35254 -0.00005
H -0.88695 2.38125 0.00003
C -2.40430 -2.01094 -0.00009
C -3.73177 -1.62029 -0.00010
C -3.09042 0.73849 -0.00007
C -4.06754 -0.25122 -0.00008
H 5.08423 1.63101 0.00016
H 5.76447 -0.93598 0.00012
H 3.54865 -2.47361 0.00003
H 0.24100 -2.50841 -0.00006
H -2.13866 -3.06588 -0.00010
H -4.52130 -2.36797 -0.00011
C -3.48796 2.19602 -0.00003
H -3.10181 2.72178 0.88305
H -3.10202 2.72178 -0.88320
H -4.57757 2.30200 0.00010
H -5.11619 0.03803 -0.00008
29
18m_9.log Energy: -411061.5216998
C -1.83145 1.54252 0.00000
C -0.52833 0.85169 0.00000
C -0.78403 -0.58279 -0.00000
C -2.23794 -0.73822 -0.00000
C -2.82460 0.60937 0.00000
C -3.25738 -1.64418 -0.00000
C -4.52519 -0.88416 -0.00000
C -4.27378 0.46244 0.00000
H -1.95720 2.61993 0.00001
C 0.25044 -1.47242 -0.00000
C 1.60143 -0.98585 -0.00000
C 0.74926 1.36553 0.00000
C 1.85733 0.42765 0.00000
C 2.69352 -1.88531 -0.00000
C 4.00153 -1.42699 -0.00000
C 3.20550 0.86313 0.00000
C 4.25787 -0.04169 0.00000
H -5.00529 1.26201 0.00000
H -5.50714 -1.34695 -0.00001
H -3.19135 -2.72723 -0.00001
H 0.07522 -2.54600 -0.00000
H 2.48811 -2.95381 -0.00001
H 4.82708 -2.13446 -0.00000
H 5.28277 0.32096 0.00000
H 3.42676 1.92583 0.00000
C 1.02189 2.84846 0.00001
H 1.60254 3.14539 0.88306
H 1.60254 3.14540 -0.88305
H 0.09960 3.43396 0.00001
31
19m_1.log Energy: -458907.1030352
C -1.65538 -2.12255 0.00000
C -2.25184 -0.86391 0.00000
C -1.38037 0.23868 0.00000
C -0.02696 0.11311 0.00000
C 0.61250 -1.14353 0.00000
C -0.21581 -2.26314 0.00000
H -2.25260 -3.03197 0.00000
C -2.03862 1.48421 0.00000
C 0.83450 1.23028 0.00000
H 0.19563 -3.26967 0.00000
C 0.22573 2.48130 0.00000
C -1.21669 2.60911 0.00000
H 0.81405 3.39606 0.00000
H -1.63327 3.61399 0.00000
C 2.06224 -0.80583 0.00000
C 2.19618 0.62998 0.00000
C -3.61535 -0.25024 0.00000
C -3.47682 1.11825 0.00000
H -4.30478 1.82007 0.00000
C 3.45797 1.21074 0.00000
C 4.59588 0.38260 0.00000
H 3.56875 2.29240 0.00000
H 5.58548 0.83260 0.00000
C 3.19439 -1.61061 0.00000
C 4.46625 -1.00787 0.00000
H 3.10251 -2.69415 0.00000
H 5.35552 -1.63325 0.00000
C -4.89243 -1.02944 0.00000
H -4.95881 -1.68226 -0.88163
H -4.95881 -1.68226 0.88163
H -5.76595 -0.36839 0.00000
31
19m_3.log Energy: -458905.6653476
C -2.61805 0.64027 0.00000
C -2.39614 -0.74198 0.00000
C -1.05968 -1.17748 0.00000
C 0.00000 -0.32607 0.00000
C -0.16905 1.07243 0.00000
C -1.47859 1.54167 0.00000
C -0.91309 -2.57835 0.00000
C 1.33438 -0.77571 0.00000
H -1.68925 2.60903 0.00000
C 1.52452 -2.15576 0.00000
C 0.39626 -3.05944 0.00000
H 2.52172 -2.58916 0.00000
H 0.60507 -4.12716 0.00000
C 1.22366 1.59991 0.00000
C 2.13397 0.48124 0.00000
C -3.17439 -2.00603 0.00000
C -2.31125 -3.07608 0.00000
H -2.60870 -4.11948 0.00000
C 3.50511 0.70081 0.00000
C 3.98960 2.02289 0.00000
H 4.19996 -0.13566 0.00000
H 5.06219 2.20000 0.00000
C 1.71575 2.89824 0.00000
C 3.10825 3.10559 0.00000
H 1.03603 3.74705 0.00000
H 3.49863 4.12015 0.00000
C -4.01367 1.21591 0.00000
H -4.57686 0.88581 -0.88251
H -4.57686 0.88581 0.88251
H -4.00073 2.31084 0.00000

```
H         -4.25672       -2.07969        0.00000
31
19m_4.log        Energy: -458905.7689507
C         -2.75584       -0.44686        0.00000
C         -2.10577       -1.67826        0.00000
C         -0.70011       -1.63878        0.00000
C          0.00000       -0.47373        0.00000
C         -0.62842        0.78763        0.00000
C         -2.02664        0.80994        0.00000
C         -0.09004       -2.90626        0.00000
C          1.40970       -0.44809        0.00000
C          2.05595       -1.68211        0.00000
C          1.30405       -2.91694        0.00000
H          3.14129       -1.74980        0.00000
H          1.86340       -3.84997        0.00000
C          0.50628        1.75062        0.00000
C          1.74052        1.00196        0.00000
C         -2.41456       -3.13220        0.00000
C         -1.24185       -3.84660        0.00000
H         -1.17028       -4.92919        0.00000
C          2.96021        1.66539        0.00000
C          2.97806        3.07303        0.00000
H          3.89295        1.10633        0.00000
H          3.93060        3.59702        0.00000
C          0.54096        3.14079        0.00000
C          1.78583        3.79889        0.00000
H         -0.37872        3.71859        0.00000
H          1.81524        4.88577        0.00000
H         -3.40970       -3.56434        0.00000
H         -3.84279       -0.38922        0.00000
C         -2.79548        2.10933        0.00000
H         -2.55093        2.71472        0.88278
H         -2.55093        2.71472       -0.88278
H         -3.87717        1.93884        0.00000
31
19m_5.log        Energy: -458906.1363148
C         -2.70662       -1.15504        0.00000
C         -1.81093       -2.22169        0.00000
C         -0.44454       -1.88162        0.00000
C          0.00000       -0.59652        0.00000
C         -0.87651        0.51003        0.00000
C         -2.23936        0.21431        0.00000
C          0.41943       -2.99303        0.00000
C          1.37213       -0.27648        0.00000
C          2.26240       -1.34720        0.00000
C          1.78455       -2.71139        0.00000
H          3.33774       -1.18597        0.00000
H          2.52659       -3.50701        0.00000
C          0.03389        1.68954        0.00000
C          1.39360        1.21157        0.00000
C         -1.80278       -3.70743        0.00000
C         -0.50498       -4.15610        0.00000
H         -0.20409       -5.19846        0.00000
C          2.45401        2.10697        0.00000
C          2.17347        3.48516        0.00000
H          3.48187        1.75263        0.00000
H          2.99170        4.20121        0.00000
C         -0.24275        3.05801        0.00000
C          0.85637        3.94476        0.00000
H          0.66443        5.01562        0.00000
H         -2.68334       -4.34120        0.00000
H         -3.78187       -1.32028        0.00000
C         -1.66052        3.57216        0.00000
H         -2.21081        3.22070       -0.88248
H         -2.21081        3.22070        0.88248
H         -1.68673        4.66694        0.00000
H         -2.98980        0.99896        0.00000
31
19m_6.log        Energy: -458905.3764485
C         -1.93349       -2.45770        0.00000
C         -2.75322       -1.33007        0.00000
C         -2.09612       -0.08482        0.00000
C         -0.74180        0.03809        0.00000
C          0.11640       -1.08063        0.00000
C         -0.49303       -2.33242        0.00000
C         -2.96741        1.02112        0.00000
C         -0.09789        1.29355        0.00000
C         -0.92438        2.41419        0.00000
C         -2.36419        2.27761        0.00000
H         -0.51138        3.42003        0.00000
H         -2.95725        3.18970        0.00000
C          1.48104       -0.48395        0.00000
C          1.34990        0.95178        0.00000
C         -4.19317       -0.96571        0.00000
C         -4.31785        0.40203        0.00000
H         -5.25801        0.94348        0.00000
C          2.48969        1.74171        0.00000
C          3.75682        1.12705        0.00000
H          2.41291        2.82654        0.00000
H          4.64721        1.75183        0.00000
C          2.73657       -1.07042        0.00000
C          3.89895       -0.26468        0.00000
H         -5.01932       -1.66897        0.00000
H         -2.35299       -3.46135        0.00000
H          0.09508       -3.24718        0.00000
H          2.83608       -2.15427        0.00000
C          5.26178       -0.91628        0.00000
H          5.39571       -1.55554        0.88269
H          5.39571       -1.55554       -0.88269
H          6.06485       -0.17137        0.00000
33
20m_1.log        Energy: -459687.0589819
C         -2.79169       -2.21740        0.06444
C         -2.49899       -0.86303        0.23169
C         -1.16216       -0.40669       -0.00560
C         -0.13377       -1.38079       -0.19832
C         -0.49040       -2.73350       -0.38661
C         -1.80888       -3.14444       -0.29464
C         -0.78378        1.01573       -0.06721
C          1.28151       -0.98610       -0.10954
C          1.62742        0.37178        0.12232
C          0.58417        1.39888        0.04667
C          2.98268        0.70066        0.35938
H          3.26077        1.72382        0.58608
C          3.98249       -0.25828        0.31877
C          3.65007       -1.59187        0.03529
C          2.32390       -1.94253       -0.16551
H         -3.80996       -2.55896        0.23716
H          0.26909       -3.47815       -0.59169
H         -2.06913       -4.18896       -0.44718
H          5.01510        0.02480        0.50695
H          4.42352       -2.35460       -0.00940
H          2.09818       -2.98630       -0.34889
C          0.91624        2.77240       -0.00145
H          1.95243        3.08107        0.07758
C         -1.73087        2.03374       -0.33469
H         -2.75605        1.77169       -0.54813
C         -0.04506        3.75272       -0.19459
H          0.24561        4.79960       -0.23225
C         -1.38019        3.37496       -0.38989
H         -2.14012        4.12260       -0.60321
C         -3.63321        0.00286        0.75065
H         -3.28009        0.79788        1.41449
H         -4.21721        0.47137       -0.05175
H         -4.32889       -0.62046        1.32328
33
20m_2.log        Energy: -459694.6463740
C         -3.43077       -0.38425        0.00921
C         -2.45576        0.60413        0.01428
C         -1.06922        0.31605       -0.00050
C         -0.65693       -1.04305       -0.01338
C         -1.65991       -2.04276       -0.01981
C         -3.00675       -1.72839       -0.01042
C         -0.06331        1.38629       -0.00271
C          0.77059       -1.38104       -0.01158
C          1.74615       -0.34552        0.01103
C          1.32222        1.05997        0.00681
C          3.11468       -0.70760        0.03143
H          3.88034        0.05919        0.05574
C          3.52171       -2.03210        0.02244
C          2.55939       -3.05332       -0.00907
C          1.21321       -2.72554       -0.02416
H         -2.79153        1.63483        0.03348
H         -1.38529       -3.09153       -0.02987
H         -3.74549       -2.52751       -0.01534
H          4.58149       -2.27382        0.03853
H          2.86330       -4.09709       -0.01947
H          0.49196       -3.53392       -0.04562
C          2.26474        2.11624        0.00454
H          3.32600        1.89702        0.00784
C         -0.43356        2.75273       -0.01776
H         -1.48023        3.03310       -0.03324
C          1.87552        3.44583       -0.00620
H          2.62752        4.23097       -0.00818
C          0.50958        3.76789       -0.01838
H          0.18841        4.80641       -0.03063
C         -4.90174       -0.04632        0.02650
H         -5.39482       -0.47071        0.91108
H         -5.06818        1.03636        0.03567
H         -5.41252       -0.45877       -0.85364
33
21m_1.log        Energy: -459694.3423299
C          2.20225       -0.56714       -0.00002
C          1.53300        0.69873       -0.00004
```

C 0.07828 0.74243 -0.00003
C -0.66933 -0.46383 0.00002
C 0.04062 -1.70523 0.00003
C 1.40706 -1.75524 0.00001
C -0.63170 1.98843 -0.00006
C -2.12347 -0.42066 0.00004
C -2.78404 0.84645 0.00001
C -1.99688 2.03875 -0.00005
C -4.20111 0.90661 0.00004
H -4.68159 1.88281 0.00001
C -4.96078 -0.24719 0.00009
C -4.31651 -1.50358 0.00012
C -2.93492 -1.58623 0.00010
H -0.08411 2.92363 -0.00012
H -0.50661 -2.64115 0.00006
H 1.89531 -2.72406 0.00002
H -2.50635 2.99981 -0.00008
H -6.04645 -0.19113 0.00011
H -4.90885 -2.41535 0.00017
H -2.47944 -2.57013 0.00013
C 2.32826 1.87535 -0.00006
H 1.86482 2.85492 -0.00006
C 3.63235 -0.63246 -0.00004
C 3.70713 1.79981 -0.00008
H 4.29792 2.71272 -0.00010
C 4.35707 0.54846 -0.00007
H 5.44432 0.51108 -0.00008
C 4.34812 -1.96310 -0.00002
H 4.09266 -2.56338 0.88320
H 4.09263 -2.56342 -0.88320
H 5.43340 -1.81849 -0.00004
33
21m_2.log Energy: -459695.1372395
C 2.01762 -0.89695 0.00009
C 1.46386 0.41850 0.00006
C 0.02036 0.58582 0.00009
C -0.82359 -0.55631 0.00009
C -0.22156 -1.85620 0.00016
C 1.13542 -2.02021 0.00017
C -0.58015 1.88719 0.00012
C -2.26885 -0.38951 0.00001
C -2.81956 0.92948 0.00004
C -1.93621 2.05242 0.00012
C -4.22643 1.10986 -0.00002
H -4.62118 2.12372 0.00001
C -5.08191 0.02541 -0.00013
C -4.54687 -1.28127 -0.00019
C -3.17734 -1.48157 -0.00013
H 0.04857 2.77038 0.00017
H -0.84851 -2.74063 0.00023
H 1.56313 -3.02047 0.00024
H -2.36379 3.05270 0.00016
H -6.15872 0.17419 -0.00018
H -5.21512 -2.13891 -0.00030
H -2.80720 -2.50072 -0.00020
C 2.37777 1.50662 -0.00004
H 2.01379 2.52819 -0.00009
C 3.42518 -1.07701 0.00003
H 3.81420 -2.09390 0.00006
C 3.74381 1.30088 -0.00011
H 4.41282 2.15943 -0.00019
C 4.29914 -0.00441 -0.00007
C 5.79648 -0.18947 -0.00018
H 6.25346 0.27807 0.88211
H 6.07231 -1.24960 -0.00007
H 6.25329 0.27785 -0.88267
33
21m_3.log Energy: -459695.0557342
C -2.63775 -1.06291 0.00467
C -2.24583 0.31158 0.00005
C -0.82976 0.64721 0.00457
C 0.14291 -0.38808 0.00878
C -0.30148 -1.75034 0.01418
C -1.62868 -2.07404 0.01328
C -0.38765 2.00944 0.00501
C 1.55866 -0.05371 0.00659
C 1.94705 1.31964 0.00636
C 0.94067 2.33152 0.00717
C 3.32539 1.65525 0.00380
H 3.60649 2.70647 0.00521
C 4.29528 0.67569 -0.00139
C 3.93420 -0.69616 -0.00364
C 2.58906 -1.03280 0.00196
H -1.11430 2.81354 0.00436
H 0.42543 -2.55418 0.01992
H -1.93615 -3.11761 0.01801
H 1.24945 3.37479 0.00814
H 5.34824 0.95050 -0.00429
H 2.33338 -2.08728 0.00044
C -3.27681 1.28899 -0.01051
H -3.03080 2.34515 -0.01524
C -4.01365 -1.40748 0.00001
H -4.28640 -2.46079 0.00394
C -4.61326 0.92917 -0.01565
H -5.37741 1.70267 -0.02402
C -4.99079 -0.43132 -0.01027
H -6.04279 -0.70538 -0.01426
C 5.01217 -1.75311 -0.02137
H 4.59108 -2.76265 0.03694
H 5.60961 -1.68996 -0.94091
H 5.70583 -1.62655 0.82011
33
21m_4.log Energy: -459687.7397487
C -2.15738 -1.26684 0.10619
C -1.74247 0.10637 0.02715
C -0.30549 0.37949 0.09588
C 0.63211 -0.68889 -0.00097
C 0.15191 -2.03580 -0.00568
C -1.17970 -2.30851 0.09434
C 0.21979 1.69355 0.33459
C 2.06047 -0.41537 -0.03475
C 2.52222 0.92470 0.13973
C 1.56179 1.95435 0.36034
C 3.91231 1.20719 0.11472
H 4.23735 2.23626 0.25357
C 4.83666 0.20092 -0.08389
C 4.39009 -1.12706 -0.26443
C 3.03940 -1.42616 -0.23698
H -0.45228 2.50732 0.55141
H 0.85219 -2.86199 -0.04053
H -1.52391 -3.33926 0.14269
H 1.91310 2.96398 0.56248
H 5.90000 0.42622 -0.10452
H 5.11263 -1.92254 -0.42959
H 2.73931 -2.45657 -0.39227
C -2.77524 1.09624 -0.14706
C -3.52825 -1.61810 0.15845
H -3.79640 -2.66913 0.24083
C -4.10862 0.69482 -0.09850
H -4.88121 1.44861 -0.23330
C -4.49918 -0.64379 0.08347
H -5.55441 -0.90242 0.12258
C -2.54051 2.55971 -0.47264
H -2.31230 3.16336 0.41504
H -1.72690 2.69977 -1.19158
H -3.44662 2.98540 -0.91654
33
21m_5.log Energy: -459687.4624981
C -2.44874 0.77599 -0.00972
C -1.91812 -0.54529 -0.00856
C -0.47904 -0.71719 0.08480
C 0.39751 0.40847 0.05483
C -0.18548 1.73654 0.17009
C -1.55261 1.87178 0.13071
C 0.06898 -2.03194 0.24371
C 1.83554 0.15581 -0.04576
C 2.33350 -1.17426 0.15088
C 1.41212 -2.24828 0.32871
C 3.72695 -1.43442 0.10980
H 4.06821 -2.45228 0.28693
C 4.63250 -0.43324 -0.18024
C 4.15118 0.85987 -0.46761
C 2.79649 1.14144 -0.40377
H -0.59816 -2.88075 0.33761
H -1.98558 2.86509 0.23361
H 1.79930 -3.25176 0.49060
H 5.69817 -0.64479 -0.21685
H 4.84491 1.64596 -0.75580
H 2.47944 2.13450 -0.68008
C -2.83486 -1.62560 -0.12011
H -2.47736 -2.64878 -0.14735
C -3.84992 0.98052 -0.10773
H -4.23050 1.99976 -0.09928
C -4.19698 -1.40492 -0.22361
H -4.87258 -2.25154 -0.31766
C -4.71486 -0.09037 -0.21440
H -5.78661 0.07370 -0.29345
C 0.60381 3.00357 0.44184
H 1.42837 2.83525 1.14200
H 1.02296 3.45006 -0.46844
H -0.05831 3.75485 0.88488
33
21m_6.log Energy: -459694.6458036
C 2.32325 0.55257 0.00004

C 1.68684 -0.73027 0.00005
C 0.23517 -0.81730 0.00002
C -0.53212 0.37330 -0.00001
C 0.14995 1.63109 -0.00003
C 1.51547 1.74758 0.00000
C -0.44544 -2.07825 0.00002
C -1.98519 0.30055 -0.00004
C -2.61950 -0.98066 -0.00003
C -1.80958 -2.15729 -0.00000
C -4.03528 -1.07008 -0.00006
H -4.49496 -2.05625 -0.00005
C -4.81919 0.06719 -0.00008
C -4.20136 1.33697 -0.00008
C -2.82190 1.44854 -0.00006
H 0.12305 -3.00118 0.00004
H -0.43129 2.54649 -0.00006
H -2.29979 -3.12828 -0.00000
H -5.90344 -0.01183 -0.00010
H -4.81270 2.23611 -0.00010
H -2.38779 2.44195 -0.00006
C 2.51520 -1.88368 0.00009
H 2.07129 -2.87252 0.00010
C 3.74156 0.62057 0.00005
H 4.22936 1.59004 0.00002
C 3.89522 -1.78809 0.00010
H 4.49846 -2.69265 0.00013
C 4.51761 -0.52361 0.00008
H 5.60218 -0.44809 0.00009
C 2.15905 3.11305 -0.00003
H 2.79436 3.26154 0.88305
H 2.79455 3.26140 -0.88301
H 1.39809 3.90023 -0.00018
38
23m_10.log Energy: -485121.2717384
C -3.44813 1.74686 -0.01285
C -2.08820 1.96847 0.00262
C -1.15058 0.89013 -0.00718
C -1.64518 -0.45936 -0.03132
C -3.05825 -0.66785 -0.04537
C -3.92495 0.39665 -0.03674
C 0.24125 1.11064 0.00597
C -0.71978 -1.51680 -0.04028
C 0.65839 -1.29003 -0.02725
C 1.16358 0.06245 -0.00432
C 2.61541 0.27406 0.00817
C 3.48258 -0.85887 -0.00103
C 2.92732 -2.19053 -0.02345
C 1.58432 -2.39527 -0.03646
H 0.58148 2.14140 0.02410
H -1.70441 2.98721 0.02141
H -3.43785 -1.68669 -0.06249
H -4.99854 0.21996 -0.04762
H -1.08702 -2.54149 -0.05774
H 3.61446 -3.03397 -0.03019
H 1.17956 -3.40488 -0.05375
C 4.88368 -0.66825 0.01171
H 5.52984 -1.54363 0.00485
C 3.20444 1.55915 0.02825
H 2.57780 2.44490 0.03484
C 5.43318 0.60390 0.03189
H 6.51218 0.73553 0.04123
C 4.58274 1.72480 0.03998
H 5.00371 2.72700 0.05536
C -4.44395 2.87986 -0.01247
H -3.94882 3.85427 0.05958
H -5.04696 2.87439 -0.93076
H -5.14442 2.79203 0.82868
C -6.14122 -2.75782 0.10125
H -5.93869 -2.09674 0.94987
H -5.40784 -3.57099 0.08797
H -7.14734 -3.17988 0.20147
H -6.07702 -2.19146 -0.83351
33
23m_11.log Energy: -459692.6328931
C -4.29209 0.79158 0.00022
C -3.00443 1.28724 0.00019
C -1.90221 0.35828 0.00011
C -2.17603 -1.05679 0.00007
C -3.52937 -1.51154 0.00011
C -4.56141 -0.60603 0.00018
C -0.55792 0.78516 0.00007
C -1.09396 -1.95461 -0.00002
C 0.23171 -1.51950 -0.00006
C 0.51789 -0.10592 -0.00001
C 1.91851 0.33100 -0.00006
C 2.95206 -0.65286 -0.00018
C 2.61205 -2.05521 -0.00023
C 1.31791 -2.46761 -0.00017
H -0.36425 1.85048 0.00011
H -5.12866 1.48718 0.00027
H -3.72389 -2.58191 0.00007
H -5.59339 -0.94863 0.00021
H -1.29790 -3.02404 -0.00006
H 3.42282 -2.78061 -0.00031
H 1.07454 -3.52791 -0.00021
C 4.30657 -0.24773 -0.00024
H 5.07991 -1.01317 -0.00033
C 2.30173 1.69212 -0.00000
H 1.54715 2.47194 0.00009
C 4.65231 1.09427 -0.00018
H 5.69794 1.39160 -0.00023
C 3.63787 2.06924 -0.00006
H 3.89802 3.12490 -0.00001
C -2.75519 2.77564 0.00022
H -2.18390 3.09082 0.88353
H -2.18396 3.09086 -0.88312
H -3.70043 3.32805 0.00026
33
23m_12.log Energy: -459683.7352801
C 4.40307 -1.12020 -0.08807
C 3.08972 -1.50269 0.07751
C 2.02596 -0.54555 0.07378
C 2.38320 0.83705 -0.10351
C 3.75448 1.20244 -0.25649
C 4.74517 0.24922 -0.25148
C 0.65426 -0.92355 0.22670
C 1.36593 1.80470 -0.07293
C 0.02648 1.44308 0.01609
C -0.35850 0.04681 0.06238
C -1.79931 -0.24893 -0.04073
C -2.74738 0.80696 0.13583
C -2.29295 2.16962 0.26451
C -0.97722 2.47321 0.14129
H 5.18639 -1.87440 -0.09833
H 2.86452 -2.55836 0.18257
H 3.99838 2.25561 -0.38042
H 5.78661 0.53621 -0.37408
H 1.63194 2.85956 -0.10775
H -3.03560 2.95358 0.39544
H -0.64195 3.50785 0.16382
C -4.13193 0.53105 0.09209
H -4.83005 1.34960 0.25455
C -2.31418 -1.51548 -0.40772
H -1.63976 -2.31230 -0.68945
C -4.60250 -0.74247 -0.19019
H -5.67108 -0.93901 -0.22706
C -3.68072 -1.76274 -0.47478
H -4.03117 -2.75042 -0.76434
C 0.36874 -2.34602 0.66892
H -0.59220 -2.42256 1.18109
H 0.36897 -3.06966 -0.15771
H 1.13098 -2.67473 1.38236
33
23m_1.log Energy: -459686.4533964
C -4.43202 1.35796 0.20960
C -3.08692 1.64457 0.26200
C -2.11344 0.61078 0.11054
C -2.56417 -0.73831 -0.08425
C -3.96611 -1.00203 -0.13537
C -4.87668 0.01970 0.00670
C -0.72904 0.88325 0.14712
C -1.59169 -1.74833 -0.18346
C -0.22527 -1.47182 -0.12466
C 0.24800 -0.10465 -0.00832
C 1.70249 0.15255 -0.00136
C 2.57091 -0.98437 0.10848
C 2.04111 -2.32592 0.05117
C 0.71630 -2.56047 -0.10766
H -0.45140 1.90831 0.33423
H -5.16436 2.15367 0.32203
H -2.74646 2.66687 0.41414
H -4.30103 -2.02639 -0.28467
H -5.94283 -0.19000 -0.03246
H -1.91387 -2.78393 -0.27806
H 2.74528 -3.15310 0.10854
H 0.33489 -3.57615 -0.18501
C 3.96759 -0.82490 0.22718
H 4.58955 -1.71149 0.32753
C 2.31759 1.44194 -0.12096
C 4.53565 0.43525 0.19725
H 5.61105 0.56406 0.29072
C 3.70742 1.54627 -0.00386
H 4.16134 2.53064 -0.09293
C 1.58731 2.73130 -0.44006

```
H          0.83327        2.59713       -1.22307
H          1.08770        3.16450        0.43583
H          2.30269        3.47934       -0.79726
33
23m_2.log         Energy: -459692.5757647
C          4.44342       -1.74155        0.00028
C          3.07786       -1.90922        0.00022
C          2.20250       -0.78065        0.00013
C          2.77568        0.53936        0.00010
C          4.19723        0.67610        0.00017
C          5.00978       -0.43386        0.00025
C          0.79992       -0.92309        0.00006
C          1.90805        1.64647        0.00002
C          0.52029        1.49668       -0.00004
C         -0.06180        0.17399       -0.00002
C         -1.52440        0.04425       -0.00008
C         -2.32302        1.22620       -0.00013
C         -1.69341        2.52424       -0.00015
C         -0.34132        2.65367       -0.00010
H          0.40353       -1.93372        0.00009
H          5.09960       -2.60841        0.00034
H          2.64459       -2.90727        0.00023
H          4.62553        1.67626        0.00015
H          6.09087       -0.31892        0.00030
H          2.33071        2.64967        0.00001
H         -2.33212        3.40508       -0.00019
H          0.12034        3.63858       -0.00012
C         -3.72924        1.10750       -0.00018
H         -4.33153        2.01378       -0.00022
C         -2.18401       -1.20312       -0.00009
H         -1.60329       -2.12109       -0.00006
C         -4.34479       -0.13463       -0.00018
H         -5.43055       -0.20197       -0.00021
C         -3.57347       -1.31514       -0.00013
C         -4.22950       -2.67658       -0.00012
H         -3.93713       -3.26005       -0.88308
H         -3.93733       -3.25994        0.88297
H         -5.32196       -2.59570       -0.00025
33
23m_3.log         Energy: -459692.9048771
C          4.68931       -1.55291       -0.00000
C          3.34077       -1.82504       -0.00000
C          2.38090       -0.76681       -0.00000
C          2.85135        0.59347        0.00000
C          4.25853        0.83858        0.00000
C          5.15370       -0.20563        0.00000
C          0.99355       -1.01613       -0.00000
C          1.90206        1.63124        0.00000
C          0.52955        1.37572        0.00000
C          0.05061        0.01269        0.00000
C         -1.39550       -0.22671        0.00000
C         -2.28611        0.88442       -0.00000
C         -1.75851        2.22758       -0.00000
C         -0.41995        2.46119       -0.00000
H          0.67394       -2.05373       -0.00001
H          5.41032       -2.36676       -0.00000
H          2.98541       -2.85353       -0.00001
H          4.60942        1.86842        0.00001
H          6.22282       -0.00794        0.00000
H          2.24785        2.66350        0.00000
H         -2.46287        3.05685       -0.00001
H         -0.03688        3.47938       -0.00000
C         -3.68344        0.66613        0.00000
H         -4.34178        1.53338       -0.00000
C         -1.96576       -1.52064        0.00000
H         -1.32678       -2.39785        0.00001
C         -4.22999       -0.61025        0.00000
C         -3.33900       -1.70731        0.00001
H         -3.74060       -2.71879        0.00001
C         -5.72170       -0.83987       -0.00000
H         -6.03400       -1.41346        0.88267
H         -6.27496        0.10558        0.00009
H         -6.03402       -1.41330       -0.88277
33
23m_4.log         Energy: -459691.8569438
C         -4.78436        1.18357        0.00007
C         -3.47587        1.60757        0.00004
C         -2.40128        0.66630        0.00008
C         -2.71331       -0.73896        0.00014
C         -4.08330       -1.14336        0.00016
C         -5.09188       -0.20825        0.00013
C         -1.05237        1.07444        0.00004
C         -1.65033       -1.65888        0.00015
C         -0.31701       -1.24464        0.00011
C          0.00497        0.16260        0.00006
C          1.41591        0.56968       -0.00000
C          2.43658       -0.43014       -0.00008
C          2.05478       -1.82348        0.00000
C          0.75131       -2.20887        0.00010
H         -0.86039        2.14263       -0.00001
H         -5.59291        1.91049        0.00004
H         -3.23968        2.66973        0.00000
H         -4.31372       -2.20678        0.00020
H         -6.13159       -0.52622        0.00014
H         -1.87182       -2.72474        0.00019
H          2.82985       -2.58256       -0.00000
H          0.49071       -3.26516        0.00016
C          3.81256       -0.05270       -0.00021
C          1.80446        1.92844       -0.00001
H          1.05728        2.71452        0.00008
C          4.14162        1.29834       -0.00023
H          5.18831        1.59455       -0.00033
C          3.14277        2.28629       -0.00011
H          3.42147        3.33731       -0.00010
C          4.90815       -1.09430       -0.00032
H          4.85433       -1.74364        0.88302
H          4.85393       -1.74392       -0.88343
H          5.89389       -0.61805       -0.00062
33
23m_5.log         Energy: -459692.5016389
C         -4.72317       -1.11540       -0.00023
C         -3.43248       -1.59163       -0.00018
C         -2.32128       -0.69446       -0.00013
C         -2.57529        0.72227       -0.00015
C         -3.92780        1.18121       -0.00020
C         -4.97367        0.28746       -0.00025
C         -0.98918       -1.15572       -0.00007
C         -1.47564        1.59947       -0.00010
C         -0.15993        1.13250       -0.00003
C          0.10093       -0.28515       -0.00002
C          1.49632       -0.73640        0.00007
C          2.55180        0.22751        0.00014
C          2.24647        1.65351        0.00011
C          0.94479        2.05546        0.00002
H         -0.83778       -2.23047       -0.00006
H         -5.56032       -1.80911       -0.00027
H         -3.23884       -2.66239       -0.00017
H         -4.11557        2.25300       -0.00021
H         -5.99955        0.64761       -0.00029
H         -1.65547        2.67317       -0.00011
H          0.70950        3.11840       -0.00001
C          3.89222       -0.22690        0.00025
H          4.70185        0.49525        0.00031
C          1.84126       -2.10727        0.00009
H          1.06289       -2.86309        0.00002
C          4.20022       -1.57933        0.00027
H          5.23866       -1.90128        0.00035
C          3.16387       -2.52699        0.00018
H          3.39335       -3.58972        0.00019
C          3.36210        2.66918        0.00018
H          4.00589        2.56269        0.88323
H          4.00607        2.56263       -0.88273
H          2.96011        3.68745        0.00012
33
23m_6.log         Energy: -459692.5999664
C          4.42974       -1.54545        0.00033
C          3.10405       -1.91346        0.00029
C          2.07288       -0.92554        0.00019
C          2.44197        0.46267        0.00012
C          3.82721        0.80911        0.00016
C          4.79549       -0.16826        0.00026
C          0.70630       -1.26654        0.00014
C          1.42095        1.43166        0.00001
C          0.06617        1.09094       -0.00004
C         -0.30740       -0.30796        0.00002
C         -1.72793       -0.67173       -0.00006
C         -2.69832        0.36946       -0.00019
C         -2.27564        1.74656       -0.00023
C         -0.96527        2.11986       -0.00016
H          0.46092       -2.32390        0.00018
H          5.20711       -2.30563        0.00040
H          2.82205       -2.96438        0.00034
H          4.10311        1.86163        0.00011
H          5.84768        0.10561        0.00029
H          1.71576        2.47648       -0.00004
H         -3.05241        2.50929       -0.00032
C         -4.07473        0.04501       -0.00027
H         -4.80236        0.85411       -0.00036
C         -2.18724       -2.00846       -0.00002
H         -1.47882       -2.83005        0.00008
C         -4.49810       -1.27475       -0.00022
H         -5.55943       -1.51044       -0.00028
C         -3.54320       -2.30798       -0.00010
H         -3.86437       -3.34670       -0.00006
```

C	-0.58069	3.57838	-0.00019
H	0.01745	3.83914	0.88314
H	0.01767	3.83905	-0.88341
H	-1.47166	4.21454	-0.00033

33

23m_7.log Energy: -459688.8662236

C	4.12354	-1.88482	-0.00004
C	2.76760	-2.10215	-0.00005
C	1.84405	-1.01119	-0.00002
C	2.33910	0.34412	0.00002
C	3.76174	0.52063	0.00004
C	4.62207	-0.55290	0.00001
C	0.46001	-1.25190	-0.00002
C	1.40666	1.41898	0.00003
C	0.02076	1.14407	0.00001
C	-0.46842	-0.21516	0.00000
C	-1.91498	-0.46719	0.00001
C	-2.81437	0.63716	-0.00003
C	-2.28724	1.97640	-0.00005
C	-0.94888	2.21636	-0.00002
H	0.13440	-2.28686	-0.00006
H	4.81672	-2.72232	-0.00007
H	2.37023	-3.11509	-0.00009
H	4.18400	1.51852	0.00008
H	5.69580	-0.38170	0.00003
H	-2.99078	2.80646	-0.00009
H	-0.60450	3.24399	-0.00004
C	-4.21024	0.41037	-0.00003
H	-4.87923	1.26847	-0.00007
C	-2.46832	-1.76878	0.00006
H	-1.81935	-2.63800	0.00011
C	-4.72396	-0.87628	0.00000
H	-5.79876	-1.03898	-0.00001
C	-3.84144	-1.97250	0.00005
H	-4.23387	-2.98633	0.00009
C	1.86445	2.86301	0.00003
H	1.49178	3.39713	-0.88321
H	1.49174	3.39714	0.88325
H	2.94894	2.96825	0.00006

33

23m_8.log Energy: -459692.6656410

C	-3.92631	-1.92675	-0.00022
C	-2.56971	-2.14010	-0.00018
C	-1.66790	-1.03288	-0.00010
C	-2.19782	0.30724	-0.00005
C	-3.62525	0.50497	-0.00010
C	-4.44791	-0.60245	-0.00017
C	-0.27220	-1.22891	-0.00006
C	-1.28088	1.37488	0.00003
C	0.10165	1.17133	0.00007
C	0.63416	-0.16869	0.00002
C	2.08913	-0.35442	0.00006
C	2.93417	0.79493	0.00013
C	2.35247	2.11548	0.00018
C	1.00570	2.29463	0.00015
H	0.08264	-2.25492	-0.00010
H	-4.61453	-2.76857	-0.00028
H	-2.16409	-3.14952	-0.00022
H	-5.52687	-0.46323	-0.00021
H	-1.64443	2.39835	0.00007
H	3.02304	2.97216	0.00024
H	0.58184	3.29650	0.00019
C	4.33861	0.63155	0.00016
H	4.96778	1.51923	0.00022
C	2.70205	-1.62827	0.00001
H	2.09236	-2.52585	-0.00005
C	4.91220	-0.63018	0.00011
H	5.99356	-0.74127	0.00014
C	4.08341	-1.76724	0.00004
H	4.52367	-2.76127	0.00000
C	-4.20548	1.89784	-0.00006
H	-3.88785	2.46813	0.88305
H	-3.88779	2.46820	-0.88310
H	-5.29993	1.86434	-0.00010

33

23m_9.log Energy: -459693.0941722

C	3.94338	-1.45740	-0.00011
C	2.60956	-1.78388	-0.00007
C	1.60639	-0.76669	0.00004
C	2.02901	0.60728	0.00010
C	3.42663	0.90364	0.00008
C	4.37798	-0.09273	-0.00003
C	0.23096	-1.06852	0.00004
C	1.04237	1.60920	0.00014
C	-0.32069	1.30333	0.00010
C	-0.74996	-0.07521	0.00007
C	-2.18690	-0.36953	0.00004
C	-3.11727	0.71183	-0.00008
C	-2.63806	2.07275	-0.00010
C	-1.30875	2.35390	0.00001
H	-0.04823	-2.11774	-0.00002
H	4.69342	-2.24611	-0.00022
H	2.29834	-2.82668	-0.00012
H	3.73309	1.94834	0.00013
H	1.34965	2.65366	0.00016
H	-3.37189	2.87594	-0.00019
H	-0.96219	3.38515	0.00003
C	-4.50519	0.44131	-0.00016
H	-5.20030	1.27846	-0.00026
C	-2.70106	-1.68631	0.00014
H	-2.02424	-2.53436	0.00028
C	-4.98089	-0.86039	-0.00009
H	-6.05065	-1.05381	-0.00015
C	-4.06767	-1.93081	0.00007
H	-4.43053	-2.95567	0.00016
C	5.85551	0.21175	-0.00010
H	6.04595	1.29040	0.00002
H	6.34782	-0.21946	0.88200
H	6.34767	-0.21923	-0.88239

33

24m_1.log Energy: -459682.4164491

C	-2.24561	2.13645	-0.41965
C	-1.06965	2.73836	-0.07881
C	0.10672	1.95593	0.15545
C	0.04803	0.54191	0.05925
C	-1.26417	-0.09550	0.05063
C	-2.38717	0.71238	-0.34248
C	1.35312	2.60663	0.42629
C	1.30479	-0.16257	-0.12082
C	2.53831	0.50991	0.16627
C	2.51988	1.89954	0.50174
C	3.76670	-0.18529	0.02270
H	4.68924	0.33291	0.27640
C	3.80213	-1.47403	-0.47473
C	2.59973	-2.09626	-0.87752
C	1.38696	-1.45463	-0.70447
H	1.34998	3.68455	0.57329
H	-3.11735	2.73040	-0.68536
H	-0.98925	3.82249	-0.04294
H	3.45829	2.39730	0.73579
H	4.75104	-1.99075	-0.59529
H	2.62695	-3.07965	-1.34060
H	0.48122	-1.93139	-1.06088
C	-3.65466	0.12018	-0.55507
H	-4.47658	0.74696	-0.89399
C	-1.53847	-1.42974	0.50343
C	-2.80811	-1.96363	0.29194
H	-3.00899	-2.97653	0.63631
C	-3.85181	-1.22089	-0.28861
H	-4.82320	-1.68169	-0.44986
C	-0.58750	-2.24700	1.35998
H	-0.10653	-3.06470	0.81120
H	0.20397	-1.63790	1.80184
H	-1.15416	-2.70241	2.18100

33

24m_2.log Energy: -459689.1398221

C	2.96836	1.52615	0.46137
C	1.94606	2.41791	0.32009
C	0.60755	1.97764	0.06362
C	0.30868	0.59135	-0.01779
C	1.42980	-0.33776	-0.09188
C	2.74899	0.13338	0.22124
C	-0.42331	2.95581	-0.10840
C	-1.09509	0.20201	-0.02345
C	-2.09332	1.20643	-0.24536
C	-1.71884	2.58300	-0.32098
C	-3.45958	0.83040	-0.31558
H	-4.19981	1.60239	-0.51566
C	-3.85523	-0.47249	-0.10048
C	-2.89941	-1.46028	0.24438
C	-1.55807	-1.10969	0.27829
H	-0.14621	4.00747	-0.08866
H	3.97419	1.86500	0.69919
H	2.12570	3.48575	0.42413
H	-2.49239	3.32817	-0.49263
H	-4.90912	-0.73917	-0.14964
H	-0.84973	-1.86092	0.60412
C	3.84449	-0.76787	0.21504
H	4.82777	-0.38968	0.48704
C	1.32322	-1.67800	-0.55669
H	0.38058	-2.03791	-0.94771
C	2.41429	-2.52950	-0.58959
H	2.29107	-3.54272	-0.96436
C	3.68378	-2.08668	-0.16304

```
H          4.53277       -2.76558       -0.17174
C         -3.35408       -2.86007        0.58155
H         -3.85707       -3.32760       -0.27581
H         -2.51302       -3.50086        0.86756
H         -4.07451       -2.85485        1.41006
33
24m_3.log        Energy: -459689.2457759
C          3.01964        1.60916        0.35691
C          1.93840        2.42539        0.19844
C          0.62625        1.88771       -0.00057
C          0.41562        0.48342       -0.00500
C          1.59222       -0.37538       -0.05898
C          2.88496        0.19464        0.19445
C         -0.47130        2.78609       -0.19268
C         -0.95916        0.00678        0.04535
C         -2.02977        0.92938       -0.19499
C         -1.74487        2.32198       -0.34991
C         -3.37364        0.47372       -0.20771
H         -4.15766        1.19740       -0.42487
C         -3.70974       -0.83696        0.08073
C         -2.65861       -1.71659        0.44093
C         -1.33763       -1.31145        0.42584
H         -0.26290        3.85299       -0.23262
H          4.00673        2.02327        0.54961
H          2.05065        3.50637        0.24374
H         -2.56809        3.00809       -0.53602
H         -2.89921       -2.73109        0.75341
H         -0.58606       -2.01046        0.76948
C          4.03584       -0.63462        0.20542
H          4.99885       -0.18017        0.42921
C          1.56123       -1.74301       -0.44945
H          0.63511       -2.18155       -0.79754
C          2.70383       -2.52458       -0.46618
H          2.63748       -3.56219       -0.78426
C          3.95187       -1.97942       -0.09872
H          4.84311       -2.60192       -0.09596
C         -5.13833       -1.32158        0.06571
H         -5.43276       -1.72000        1.04578
H         -5.83392       -0.51643       -0.19476
H         -5.27216       -2.13246       -0.66267
33
24m_4.log        Energy: -459688.0458516
C         -2.66522        1.95791       -0.34766
C         -1.44839        2.56123       -0.22201
C         -0.25471        1.78967       -0.04539
C         -0.30665        0.37239       -0.03626
C         -1.61994       -0.25323        0.06029
C         -2.79127        0.54433       -0.16784
C          0.99549        2.46080        0.12426
C          0.95293       -0.35510       -0.12227
C          2.18896        0.34226        0.10781
C          2.16172        1.76475        0.26645
C          3.43287       -0.36701        0.10017
C          3.43789       -1.71577       -0.21783
C          2.24593       -2.37858       -0.57092
C          1.03481       -1.71625       -0.52537
H          0.99683        3.54800        0.16376
H         -3.56308        2.54604       -0.52436
H         -1.35924        3.64401       -0.27604
H          3.08640        2.30369        0.44319
H          4.37981       -2.25963       -0.23584
H          2.28124       -3.41525       -0.89738
H          0.14578       -2.23584       -0.85825
C         -4.07574       -0.05657       -0.13322
H         -4.94465        0.56540       -0.33801
C         -1.83126       -1.59777        0.47463
H         -0.99173       -2.19632        0.80400
C         -3.09799       -2.15254        0.53729
H         -3.21575       -3.18010        0.87277
C         -4.23345       -1.38940        0.19389
H         -5.22431       -1.83523        0.22763
C          4.73475        0.34338        0.39286
H          4.96353        1.10286       -0.36670
H          4.71440        0.85398        1.36397
H          5.56625       -0.36861        0.40810
33
24m_5.log        Energy: -459688.6204576
C          2.33840       -2.11502       -0.40315
C          1.05376       -2.56176       -0.29598
C         -0.03248       -1.64935       -0.10089
C          0.20144       -0.25370       -0.05092
C          1.58125        0.20036        0.05884
C          2.64270       -0.73409       -0.18930
C         -1.35655       -2.16712        0.05128
C         -0.96475        0.61653       -0.11063
C         -2.27597        0.06448        0.09767
C         -2.45122       -1.36367        0.22021
C         -3.39849        0.93390        0.10391
H         -4.38527        0.52550        0.29640
C         -3.27088        2.28419       -0.16507
C         -2.00524        2.80673       -0.49362
C         -0.88803        1.99056       -0.46847
H         -1.47689       -3.24884        0.06044
H          3.15507       -2.80790       -0.59308
H          0.82811       -3.62259       -0.37984
H         -4.14726        2.92756       -0.16321
H         -1.90353        3.84898       -0.78600
H          0.05934        2.40769       -0.78444
C          3.99288       -0.30091       -0.14666
H          4.77570       -1.02288       -0.37018
C          1.96148        1.49729        0.50397
H          1.20567        2.19025        0.85074
C          3.28837        1.88547        0.57489
H          3.53578        2.88180        0.93321
C          4.31828        0.99348        0.20908
H          5.35756        1.30989        0.24795
C         -3.81487       -1.96385        0.45845
H         -4.25907       -1.59830        1.39344
H         -4.51548       -1.71745       -0.35020
H         -3.75028       -3.05484        0.52295
33
24m_6.log        Energy: -459688.1899184
C         -2.25868        1.91203       -0.42938
C         -0.99806        2.41552       -0.28178
C          0.13029        1.56146       -0.05160
C         -0.05502        0.15193       -0.02226
C         -1.41729       -0.35852        0.05740
C         -2.51350        0.52341       -0.21853
C          1.44131        2.13943        0.14997
C          1.12528       -0.69871       -0.07349
C          2.40574       -0.10752        0.16998
C          2.51466        1.30803        0.32156
C          3.56996       -0.91803        0.18186
H          4.52698       -0.44775        0.39850
C          3.50466       -2.26563       -0.11575
C          2.26372       -2.83301       -0.47427
C          1.10931       -2.06868       -0.45562
H         -3.09502        2.57194       -0.64902
H         -0.84248        3.48550       -0.36687
H          3.49951        1.72849        0.51618
H          4.40566       -2.87403       -0.11460
H          2.21227       -3.87309       -0.78695
H          0.18670       -2.52227       -0.79500
C         -3.84427        0.03143       -0.20278
H         -4.65353        0.71718       -0.44532
C         -1.74673       -1.67095        0.49850
H         -0.96726       -2.32732        0.86359
C         -3.05571       -2.11825        0.54330
H         -3.26548       -3.12336        0.90102
C         -4.11751       -1.27466        0.15262
H         -5.14188       -1.63790        0.17265
C          1.62570        3.63759        0.19723
H          1.36927        4.10713       -0.76175
H          0.99492        4.10483        0.96440
H          2.66723        3.88946        0.42135
35
26m_1.log        Energy: -507523.7877193
C          3.51453        1.31408       -0.30273
C          2.92690        0.05472       -0.00754
C          1.49703       -0.07207        0.00217
C          0.67601        1.09421       -0.16925
C          1.30757        2.28199       -0.53753
C          2.71313        2.39201       -0.60593
C          0.91437       -1.37118        0.14140
C         -0.78812        0.98072        0.01752
C         -1.36283       -0.33925       -0.06209
C         -0.53929       -1.51392       -0.05273
C         -2.78583       -0.50305       -0.11566
C         -3.61108        0.64286        0.01782
C         -3.04555        1.87181        0.25215
C         -1.63865        2.07262        0.27446
H          4.59853        1.40162       -0.31368
H          0.72317        3.15253       -0.79837
H          3.15556        3.34478       -0.88617
H         -4.69175        0.52784       -0.02378
H         -3.68892        2.73206        0.42390
C         -3.34885       -1.79593       -0.27090
H         -4.43049       -1.89446       -0.32931
C         -1.13834       -2.76233       -0.21617
H         -0.52668       -3.65761       -0.25442
C          3.73118       -1.08567        0.25441
H          4.81302       -0.97462        0.26466
C          3.14490       -2.31274        0.48613
H          3.75930       -3.18285        0.70375
```

```
C         -2.53325       -2.90599       -0.33769
H         -2.96012       -3.89712       -0.46860
C          1.74539       -2.45627        0.41446
H          1.32262       -3.44269        0.57260
C         -1.18495        3.47193        0.63468
H         -0.24434        3.47376        1.19410
H         -1.04870        4.11357       -0.24604
H         -1.94670        3.95181        1.25947
35
26m_2.log         Energy: -507529.8688565
C         -3.38519       -1.98176       -0.05421
C         -3.03690       -0.60665        0.01193
C         -1.65247       -0.22240       -0.00587
C         -0.64251       -1.24011       -0.05739
C         -1.04222       -2.57250       -0.13695
C         -2.40144       -2.94328       -0.13779
C         -1.31100        1.17075        0.02790
C          0.78151       -0.84560       -0.01070
C          1.11978        0.54814       -0.02587
C          0.11142        1.56602       -0.03595
C          2.50398        0.92441       -0.02594
C          3.50756       -0.07949        0.02488
C          3.17795       -1.41907        0.07222
C          1.80929       -1.78065        0.05059
H         -4.43677       -2.25921       -0.04315
H         -0.30308       -3.36347       -0.20031
H         -2.66527       -3.99619       -0.19931
H          4.55119        0.22919        0.03038
H          1.57865       -2.84087        0.08673
C          2.85460        2.29936       -0.07191
H          3.90683        2.57473       -0.07599
C          0.51159        2.90005       -0.09360
H         -0.22616        3.69425       -0.12349
C         -4.04238        0.39266        0.09629
H         -5.08634        0.08829        0.11373
C         -3.69372        1.72456        0.15574
H         -4.46119        2.49130        0.22706
C          1.87166        3.26554       -0.11465
H          2.13829        4.31866       -0.15880
C         -2.33830        2.10740        0.12026
H         -2.11158        3.16679        0.16803
C          4.23092       -2.49789        0.15240
H          4.13963       -3.06735        1.08704
H          4.12963       -3.21578       -0.67194
H          5.24140       -2.07723        0.11167
35
26m_3.log         Energy: -507529.2921075
C         -3.77124       -1.49430       -0.02107
C         -3.17786       -0.20450        0.00060
C         -1.74618       -0.08044        0.00026
C         -0.93811       -1.26466       -0.01067
C         -1.57436       -2.50461       -0.03781
C         -2.97813       -2.62125       -0.04314
C         -1.15325        1.22501        0.01147
C          0.53296       -1.13421        0.00894
C          1.13232        0.16943       -0.00160
C          0.31993        1.35280       -0.01065
C          2.56722        0.29383       -0.00332
C          3.39304       -0.87766        0.01685
C          2.77430       -2.11344        0.03897
C          1.37370       -2.24156        0.03527
H         -4.85576       -1.57519       -0.02134
H         -0.99267       -3.41960       -0.05738
H         -3.42888       -3.61035       -0.06369
H          3.38129       -3.01600        0.05899
H          0.96258       -3.24474        0.05478
C          3.14516        1.59145       -0.02583
H          4.22427        1.70049       -0.02887
C          0.94730        2.59669       -0.03920
H          0.36152        3.50884       -0.05835
C         -3.98380        0.96431        0.02259
H         -5.06607        0.85750        0.02280
C         -3.39646        2.21070        0.04594
H         -4.01032        3.10772        0.06761
C          2.34755        2.71631       -0.04686
H          2.79789        3.70560       -0.06859
C         -1.99342        2.33674        0.04077
H         -1.57880        3.33811        0.06163
C          4.89820       -0.77306        0.01600
H          5.26871       -0.25143       -0.87652
H          5.26778       -0.21859        0.88889
H          5.35529       -1.76779        0.03497
37
27m_1.log         Energy: -555341.2132055
C         -1.79407        1.60855        0.00000
C         -0.43234        1.89744        0.00000
C          0.44504        0.79387        0.00000
C          0.00000       -0.49597        0.00000
C         -1.37193       -0.82694        0.00000
C         -2.26534        0.24488        0.00000
H         -2.53921        2.40081        0.00000
C          1.81610        1.12039        0.00000
C          0.87954       -1.59628        0.00000
H         -3.33926        0.08528        0.00000
C          2.24284       -1.30855        0.00000
C          2.71260        0.05416        0.00000
H          2.98667       -2.10198        0.00000
H          3.78697        0.22222        0.00000
C         -1.38461       -2.31347        0.00000
C         -0.02009       -2.77749        0.00000
C          0.47081        3.07666        0.00000
C          1.83322        2.60560        0.00000
C          0.25981       -4.13876        0.00000
C         -0.81105       -5.04721        0.00000
H          1.28657       -4.49602        0.00000
H         -0.60993       -6.11575        0.00000
C         -2.44856       -3.22038        0.00000
C         -2.13255       -4.59457        0.00000
H         -2.94412       -5.31923        0.00000
C          0.20585        4.44176        0.00000
C          1.27990        5.34858        0.00000
H         -0.81845        4.80656        0.00000
H          1.07857        6.41695        0.00000
C          2.88535        3.51480        0.00000
C          2.60127        4.89163        0.00000
H          3.91598        3.16794        0.00000
H          3.41912        5.60797        0.00000
C         -3.88056       -2.74835        0.00000
H         -4.09590       -2.13225        0.88292
H         -4.09590       -2.13225       -0.88292
H         -4.57845       -3.59237        0.00000
37
27m_2.log         Energy: -555340.3869939
C         -0.95395       -2.35163        0.00000
C         -1.73281       -1.19651        0.00000
C         -1.04128        0.03184        0.00000
C          0.32127        0.10368        0.00000
C          1.13853       -1.04494        0.00000
C          0.48609       -2.27574        0.00000
H         -1.41044       -3.33860        0.00000
C         -1.85910        1.18012        0.00000
C          1.01344        1.33249        0.00000
H          1.04390       -3.20938        0.00000
C          0.23373        2.48702        0.00000
C         -1.20658        2.41067        0.00000
H          0.68946        3.47432        0.00000
H         -1.76434        3.34437        0.00000
C          2.52178       -0.50143        0.00000
C          2.44434        0.93817        0.00000
C         -3.16465       -0.80318        0.00000
C         -3.24112        0.63658        0.00000
C          3.61416        1.68621        0.00000
C          4.85534        1.02500        0.00000
H          3.57761        2.77319        0.00000
H          5.76921        1.61508        0.00000
C          3.75659       -1.13474        0.00000
C          4.94572       -0.37297        0.00000
C         -4.32957       -1.56286        0.00000
C         -5.57429       -0.90966        0.00000
H         -4.28114       -2.64926        0.00000
H         -6.48850       -1.49806        0.00000
C         -4.48007        1.26815        0.00000
C         -5.64842        0.48664        0.00000
H         -4.54702        2.35345        0.00000
H         -6.61972        0.97504        0.00000
C          6.28526       -1.07181        0.00000
H          6.39786       -1.71514        0.88269
H          6.39786       -1.71514       -0.88269
H          7.11330       -0.35482        0.00000
H          3.81559       -2.22163        0.00000
37
27m_5.log         Energy: -555340.9287611
C         -1.80161        1.45455        0.00000
C         -0.44195        1.75140        0.00000
C          0.44370        0.65672        0.00000
C          0.00000       -0.63288        0.00000
C         -1.36901       -0.96449        0.00000
C         -2.28898        0.09044        0.00000
H         -2.54585        2.24844        0.00000
C          1.81196        0.98997        0.00000
C          0.88448       -1.73061        0.00000
C          2.24700       -1.43697        0.00000
C          2.71307       -0.07289        0.00000
H          2.99350       -2.22798        0.00000
```

```
H          3.78692        0.09840        0.00000
C         -1.37815       -2.44935        0.00000
C         -0.01127       -2.91359        0.00000
C          0.45307        2.93762        0.00000
C          1.81911        2.47585        0.00000
C          0.26460       -4.27621        0.00000
C         -0.80112       -5.19285        0.00000
H          1.29220       -4.63182        0.00000
H         -0.59086       -6.25952        0.00000
C         -2.42177       -3.37120        0.00000
C         -2.12527       -4.74576        0.00000
C          0.17883        4.30074        0.00000
C          1.24668        5.21515        0.00000
H         -0.84794        4.65861        0.00000
H          1.03793        6.28212        0.00000
C          2.86462        3.39236        0.00000
C          2.57102        4.76741        0.00000
H          3.89768        3.05276        0.00000
H          3.38398        5.48933        0.00000
H         -3.45562       -3.03849        0.00000
H         -2.93790       -5.46813        0.00000
C         -3.77705       -0.16192        0.00000
H         -4.08065       -0.73950       -0.88289
H         -4.08065       -0.73950        0.88289
H         -4.34449        0.77437        0.00000
39
29m_1.log         Energy: -603226.4047389
C          2.86788       -2.06142        0.00000
C          2.51267       -0.67944        0.00000
C          1.12835       -0.34066        0.00000
C          0.13474       -1.36955        0.00000
C          0.52405       -2.73744        0.00000
C          1.91208       -3.05054        0.00000
C          0.72207        1.02884        0.00000
C         -1.25375       -1.02800        0.00000
C         -1.65365        0.34280        0.00000
C         -0.66402        1.37181        0.00000
C         -3.03502        0.68340        0.00000
C         -4.00083       -0.36490        0.00000
C         -3.61703       -1.68503        0.00000
C         -2.23999       -2.05375        0.00000
C         -1.81842       -3.41586        0.00000
C         -0.48473       -3.74571        0.00000
H         -0.17860       -4.78962        0.00000
H         -2.57616       -4.19636        0.00000
H          3.91599       -2.34318        0.00000
H          2.21220       -4.09620        0.00000
H         -5.05656       -0.10254        0.00000
H         -4.36739       -2.47273        0.00000
C          3.49786        0.36944        0.00000
C          3.08146        1.68344        0.00000
C          1.70627        2.05210        0.00000
H          3.82415        2.47934        0.00000
C          1.28445        3.41390        0.00000
C         -0.05002        3.74533        0.00000
C         -1.05922        2.73862        0.00000
H          2.04205        4.19460        0.00000
H         -0.35418        4.78987        0.00000
C         -3.40334        2.06099        0.00000
H         -4.46023        2.31857        0.00000
C         -2.45045        3.05178        0.00000
H         -2.74975        4.09764        0.00000
C          4.97016        0.03606        0.00000
H          5.25162       -0.55235        0.88334
H          5.25162       -0.55235       -0.88334
H          5.57573        0.94798        0.00000
```

6.3. Secondary aryl alcohols

```
25
1npEtOH_001_opt_fr.log    Energy: -338722.8769703
C          2.09444       -0.78361       -0.23299
C          2.44333       -1.40797        1.11748
O          3.30693       -0.18556       -0.71926
H          1.80047       -1.58009       -0.92970
H          1.56868       -1.89736        1.55822
H          3.23932       -2.15172        0.99658
H          2.78891       -0.63097        1.80924
H          3.13842        0.17106       -1.60806
C          0.95500        0.22624       -0.11606
C          1.24158        1.57323        0.00969
C         -0.41550       -0.20483       -0.09841
C          0.21715        2.54226        0.14546
H          2.27892        1.89461        0.00613
C         -1.45247        0.78261        0.03922
C         -0.80802       -1.57188       -0.20601
C         -1.10394        2.15665        0.15803
H          0.48428        3.59213        0.23915
C         -2.81379        0.36848        0.05802
C         -2.13810       -1.94045       -0.18234
H         -0.05476       -2.34682       -0.30296
H         -1.89802        2.89328        0.25974
C         -3.15450       -0.96277       -0.04875
H         -3.58589        1.12818        0.16150
H         -2.40822       -2.99046       -0.26519
H         -4.19862       -1.26520       -0.03068
33
1pyrEtOH_001_opt_fr.log   Energy: -482987.3538708
C          3.44594       -0.74069       -0.17903
C          3.88087       -1.20098        1.21176
O          4.56921       -0.04002       -0.73953
H          3.24890       -1.62416       -0.79984
H          4.77012       -1.83742        1.13768
H          4.11905       -0.33340        1.83792
H          3.08020       -1.76855        1.69786
H          4.34466        0.22247       -1.64851
C          2.19340        0.13011       -0.12320
C          2.33439        1.52081       -0.04021
C          0.89372       -0.43685       -0.11151
C          1.23017        2.36642        0.03396
H          3.33438        1.94421       -0.03659
C         -0.24771        0.42856       -0.03543
C          0.65466       -1.85855       -0.16574
C         -0.07316        1.84603        0.03415
H          1.37418        3.44308        0.09306
C         -1.57029       -0.11641       -0.02425
C         -0.60745       -2.37644       -0.15079
H          1.49661       -2.54081       -0.21361
C         -1.23357        2.69352        0.10356
C         -2.70718        0.75016        0.04738
C         -1.76598       -1.53067       -0.08197
H         -0.75487       -3.45351       -0.19076
C         -2.49280        2.17151        0.10913
H         -1.08258        3.76972        0.15218
C         -3.99664        0.18920        0.05771
C         -3.07367       -2.04808       -0.06771
H         -3.36075        2.82506        0.16252
C         -4.17553       -1.19441        0.00071
H         -4.86000        0.84878        0.11220
H         -3.21832       -3.12533       -0.11012
H         -5.18027       -1.60939        0.01105
31
2AnEtOH_003_opt_fr.log    Energy: -435143.0713884
C          3.97449       -0.71236       -0.06100
C          4.73450       -0.44560        1.23517
O          4.76575       -0.13004       -1.11792
H          3.90891       -1.79975       -0.21501
H          4.82357        0.63094        1.42019
H          4.21275       -0.90240        2.08340
H          5.74252       -0.87141        1.17195
H          4.29577       -0.26223       -1.95889
C          2.56723       -0.14345       -0.04886
C          2.37638        1.27778       -0.01472
C          1.46937       -0.96924       -0.06620
C          1.11860        1.82029        0.00956
H          3.24994        1.92380       -0.01772
C          0.13612       -0.44753       -0.03626
H          1.60091       -2.04977       -0.10046
C         -0.04654        0.98596        0.00326
H          0.98470        2.89976        0.03400
C         -0.99141       -1.28015       -0.04586
C         -1.34537        1.51051        0.03046
C         -2.29287       -0.75650       -0.01780
H         -0.85413       -2.35972       -0.07468
C         -2.47511        0.67797        0.02158
H         -1.48232        2.59009        0.05930
C         -3.45277       -1.59589       -0.02559
C         -3.80765        1.20038        0.04949
C         -4.71459       -1.05514        0.00257
H         -3.31359       -2.67454       -0.05423
C         -4.89443        0.36113        0.04062
H         -3.94284        2.27955        0.07825
H         -5.58687       -1.70408       -0.00362
H         -5.90103        0.77117        0.06237
25
2npEtOH_003_opt_fr.log    Energy: -338724.8517652
C          2.79630       -0.57311       -0.08129
C          3.53746       -0.27983        1.21992
O          3.53944        0.08893       -1.12551
H          2.81153       -1.65858       -0.25960
H          3.55146        0.79674        1.42509
H          4.57240       -0.63405        1.14991
```

```
H          3.04857      -0.78732       2.05891
H          3.07542      -0.05481      -1.96789
C          1.34967      -0.11140      -0.05348
C          0.31357      -1.02357      -0.07537
C          1.04833       1.28032      -0.00005
C         -1.04673      -0.60654      -0.03214
H          0.52915      -2.09011      -0.12517
C         -0.25581       1.71902       0.03733
H          1.86495       1.99732       0.00124
C         -1.34040       0.79595       0.02540
C         -2.12500      -1.53434      -0.04895
H         -0.47525       2.78411       0.07617
C         -2.69875       1.21498       0.06520
C         -3.43338      -1.09724      -0.00941
H         -1.90062      -2.59822      -0.09218
C         -3.72341       0.29044       0.04798
H         -2.91767       2.28002       0.10912
H         -4.24835      -1.81693      -0.02147
H         -4.75830       0.62237       0.07904
31
2PhAnEtOH_003_opt_fr.log  Energy: -435148.1774882
C         -3.96170       0.21864      -0.07780
C         -4.57272      -0.10516       1.28303
O         -4.59440      -0.66588      -1.02336
H         -4.20502       1.26015      -0.33673
H         -5.66147       0.01580       1.24512
H         -4.17089       0.56618       2.05002
H         -4.34458      -1.13787       1.57075
H         -4.20424      -0.50638      -1.89961
C         -2.45103       0.05916      -0.08557
C         -1.61515       1.16279      -0.10176
C         -1.86685      -1.22874      -0.05193
C         -0.20516       1.02958      -0.06909
H         -2.04011       2.16497      -0.13722
C         -0.49292      -1.38668      -0.02818
H         -2.51153      -2.10347      -0.05224
C          0.38188      -0.27087      -0.03107
C          0.63719       2.19334      -0.07666
H         -0.09040      -2.39387      -0.00485
C          1.83443      -0.39427       0.00291
C          1.99444       2.07944      -0.04391
H          0.16632       3.17346      -0.10716
C          2.63133       0.79228      -0.00327
C          2.50405      -1.64283       0.04280
H          2.62286       2.96749      -0.04855
C          4.04387       0.68873       0.03187
C          3.88722      -1.71923       0.07591
H          1.93431      -2.56625       0.04947
C          4.66812      -0.54505       0.07050
H          4.63358       1.60313       0.02770
H          4.37146      -2.69220       0.10659
H          5.75292      -0.61094       0.09679
33
2pyrEtOH_001_opt_fr.log   Energy: -482989.2965535
C         -4.02916       0.32895      -0.10233
C         -4.61763       0.08269       1.28473
O         -4.63108      -0.64561      -0.97391
H         -4.31527       1.33823      -0.43480
H         -4.34779      -0.91834       1.64059
H         -4.23371       0.81995       1.99849
H         -5.71040       0.16124       1.25082
H         -4.24829      -0.53956      -1.86143
C         -2.51075       0.23347      -0.10593
C         -1.73418       1.39618      -0.11720
C         -1.88094      -1.01668      -0.06558
C         -0.33016       1.33882      -0.07808
H         -2.22142       2.36921      -0.15738
C         -0.48180      -1.12638      -0.03265
H         -2.48654      -1.91965      -0.06476
C          0.31108       0.06327      -0.03672
C          0.48932       2.52211      -0.08556
C          0.18942      -2.40012       0.00795
C          1.73508      -0.02268      -0.00056
C          1.85091       2.44105      -0.05017
H         -0.00279       3.49161      -0.11924
C          1.55049      -2.48278       0.04418
H         -0.41664      -3.30359       0.01192
C          2.52423       1.16958      -0.00650
C          2.37378      -1.30127       0.04065
H          2.45514       3.34580      -0.05572
H          2.04089      -3.45320       0.07670
C          3.92585       1.06032       0.03116
C          3.77790      -1.36148       0.07772
C          4.54201      -0.19206       0.07267
H          4.52889       1.96562       0.02749
H          4.26714      -2.33253       0.11060
H          5.62686      -0.25783       0.10137
37
2tPhnEtOH_003_opt_fr.log  Energy: -531569.5474976
C         -0.10074      -0.97371      -0.01631
C         -0.41566       0.41183      -0.02258
C         -1.77838       0.79405      -0.05277
C         -2.81330      -0.12932      -0.06467
C         -2.49135      -1.50026      -0.06568
C         -1.16898      -1.90340      -0.04551
C         -4.26228       0.32618      -0.06590
C         -4.96866       0.03558       1.25544
O         -5.02715      -0.34504      -1.08697
H         -2.04520       1.84526      -0.07056
H         -3.28514      -2.24188      -0.09266
H         -0.96310      -2.96737      -0.05406
H         -4.28763       1.41087      -0.24915
H         -6.00774       0.38167       1.20976
H         -4.96769      -1.04028       1.46563
H         -4.46248       0.55079       2.07931
H         -4.58479      -0.20487      -1.94153
C          1.29935      -1.41017       0.01166
C          2.34465      -0.44494       0.00033
C          1.64680      -2.78223       0.04782
C          3.68445      -0.90088       0.01307
C          2.96712      -3.20200       0.06199
H          0.87142      -3.53933       0.06768
C          3.99837      -2.25018       0.04122
H          4.50058      -0.18829       0.00366
H          3.19819      -4.26390       0.08980
H          5.03899      -2.56451       0.05064
C          0.66187       1.40922      -0.01016
C          0.38614       2.79781       0.00139
C          2.02088       0.98728      -0.01384
C          1.39746       3.74479      -0.00391
H         -0.63869       3.14972       0.01680
C          3.03481       1.97548      -0.02272
C          2.73761       3.32882      -0.01991
H          1.14919       4.80315       0.00457
H          4.07900       1.68476      -0.03082
H          3.54132       4.06082      -0.02708
31
9AnEtOH_001_opt_fr.log    Energy: -435136.2490245
C         -0.14187       2.29193      -0.14223
C         -0.48176       2.89973       1.21849
O         -1.14039       2.75357      -1.07587
H          0.80955       2.71921      -0.46655
H          0.30668       2.65916       1.94038
H         -0.55305       3.99015       1.13159
H         -1.43003       2.51588       1.60728
H         -0.96567       2.34065      -1.93840
C         -0.00428       0.76709      -0.09299
C          1.29529       0.18993      -0.06019
C         -1.15044      -0.07255      -0.03881
C          1.44008      -1.25204      -0.02089
C          2.51277       0.95690      -0.04212
C         -0.98390      -1.51133       0.00281
C         -2.50056       0.42374      -0.00630
C          2.74638      -1.83942       0.00130
C          0.30113      -2.06074       0.00004
C          3.74799       0.35679      -0.01175
H          2.47609       2.03972      -0.03837
C         -2.13555      -2.36193       0.05542
C         -3.57846      -0.42512       0.05737
H         -2.67121       1.49010      -0.04872
C          3.87465      -1.06214       0.00449
H          2.81998      -2.92473       0.02160
H          0.41791      -3.14255       0.02695
H          4.64314       0.97389       0.00333
H         -1.97771      -3.43815       0.07803
C         -3.40166      -1.83898       0.08411
H         -4.58392      -0.01190       0.08426
H          4.86124      -1.51795       0.02449
H         -4.26882      -2.49319       0.12852
19
PhEtOH_001_opt_fr.log     Energy: -242302.5862490
C          2.63124      -0.19062       0.10610
C          1.76512      -1.28163       0.24768
C          0.38391      -1.10088       0.14002
C         -0.15146       0.17109      -0.11506
C          0.72280       1.25636      -0.26199
C          2.10628       1.08004      -0.14818
C         -1.65637       0.37408      -0.20078
C         -2.32343       0.35041       1.17215
O         -2.29483      -0.65844      -0.97619
H          3.70617      -0.33175       0.18918
H          2.16672      -2.27341       0.44248
H         -0.28735      -1.94904       0.24683
H          0.31963       2.24650      -0.46770
```

```
H          2.77164        1.93219       -0.26557
H         -1.85001        1.35011       -0.67086
H         -1.90618        1.13552        1.81250
H         -3.40233        0.51598        1.07144
H         -2.16131       -0.61792        1.66018
H         -1.86688       -0.69375       -1.84859
28
PhEtOH_2-4-6Me_002_opt_fr.log      Energy: -
316321.9745121
C          1.73196        1.10580       -0.05349
C          0.34452        1.27718       -0.11517
C         -0.50546        0.14211       -0.09642
C          0.06813       -1.14593        0.00639
C          1.46673       -1.26876        0.06238
C         -2.01783        0.36146       -0.14598
C         -2.66838        0.32581        1.23673
O         -2.71247       -0.60174       -0.96283
H          2.37328        1.98604       -0.06802
H          1.89783       -2.26564        0.14145
H         -2.20956        1.35159       -0.57413
H         -2.23375        1.10266        1.87570
H         -3.74586        0.50967        1.14997
H         -2.51823       -0.64122        1.72730
H         -2.31364       -0.58827       -1.84887
C          2.31794       -0.16262        0.02931
C         -0.74376       -2.42668        0.05180
H         -1.06260       -2.73641       -0.95079
H         -0.14008       -3.23897        0.47295
H         -1.65312       -2.33143        0.64751
C          3.81940       -0.31449        0.10241
H          4.21970        0.13946        1.01894
H          4.11882       -1.36843        0.09430
H          4.31309        0.18189       -0.74312
C         -0.20077        2.69048       -0.18362
H         -0.74568        2.87984       -1.11794
H         -0.89061        2.90810        0.64108
H          0.61525        3.41891       -0.12966
19
PhEtOH_2Br_001_opt_fr.log Energy: -1855713.2811625
C          2.19408        1.95276        0.09566
C          2.11863        0.56462       -0.02597
C          0.88616       -0.10404       -0.09185
C         -0.27254        0.68169       -0.02509
C         -0.21786        2.07156        0.09456
C          0.85161       -1.62659       -0.18883
C          0.73583       -2.27231        1.19053
O          2.04531       -2.15502       -0.78325
H          3.16478        2.43933        0.14404
H          3.02715       -0.02877       -0.07161
H         -1.13437        2.65158        0.13923
H         -0.01172       -1.92535       -0.79708
H         -0.16248       -1.92190        1.70867
H          0.67956       -3.36183        1.08932
H          1.61145       -2.01809        1.79902
H          2.09386       -1.84977       -1.70517
C          1.02193        2.71062        0.15451
H          1.06380        3.79275        0.24598
Br        -2.01719       -0.12593       -0.10022
22
PhEtOH_2Me_001_opt_fr.log Energy: -266976.3462162
C          1.72092       -1.65583        0.16786
C          0.34801       -1.42890        0.03995
C         -0.16354       -0.13225       -0.11000
C          0.72245        0.96857       -0.12639
C          2.09836        0.72386        0.00278
C         -1.67209        0.07156       -0.20339
C         -2.30754        0.22655        1.17721
O         -2.33754       -1.04312       -0.82455
H          2.09643       -2.66991        0.28290
H         -0.34405       -2.26650        0.05420
H          2.78675        1.56669       -0.01274
H         -1.88072        0.97533       -0.79154
H         -2.14453       -0.68213        1.76818
H         -1.86396        1.07190        1.71418
H         -3.38607        0.39784        1.08279
H         -1.95964       -1.16972       -1.71141
C          2.60177       -0.57208        0.14813
H          3.67328       -0.73086        0.24461
C          0.22514        2.38999       -0.27639
H         -0.28978        2.54145       -1.23453
H         -0.48545        2.66139        0.51416
H          1.05720        3.10074       -0.23298
23
PhEtOH_2OMe_004_opt_fr.log         Energy: -
314173.1644574
C         -2.31513       -1.45142       -0.02925
C         -0.95191       -1.76159       -0.11303
C          0.03047       -0.76866       -0.10082
C         -0.38756        0.57785       -0.01141
C         -1.74575        0.90412        0.06617
C          1.50611       -1.14201       -0.15263
C          2.19927       -0.96350        1.20431
O          2.21350       -0.44369       -1.19259
H         -3.05794       -2.24469       -0.03968
H         -0.64148       -2.80202       -0.18768
H         -2.06537        1.93836        0.13028
H          1.57475       -2.19591       -0.44136
H          3.24882       -1.27068        1.12577
H          2.16356        0.08254        1.52825
H          1.71158       -1.57874        1.97052
H          2.08853        0.50700       -1.02436
C         -2.70546       -0.11605        0.05953
H         -3.75884        0.14654        0.12024
O          0.61760        1.51596       -0.01622
C          0.27727        2.89979        0.09632
H         -0.33856        3.22746       -0.75040
H         -0.24433        3.10173        1.04002
H          1.22674        3.43899        0.08381
29
PhEtOH_2Ph_001b_opt_fr.log         Energy: -
387305.4744070
C          3.00285       -1.59042       -0.21372
C          2.67893       -0.25030       -0.00653
C          1.34451        0.18458        0.04914
C          0.30846       -0.76356       -0.11046
C          0.64935       -2.11621       -0.30711
C          1.08433        1.66183        0.32685
C          1.43642        2.55650       -0.85883
O          1.89229        2.13216        1.42761
H          4.04484       -1.89875       -0.25386
H          3.47016        0.48060        0.13740
H         -0.14899       -2.84426       -0.42984
H          0.02814        1.80566        0.58108
H          0.85590        2.27521       -1.74331
H          1.22161        3.60359       -0.61662
H          2.50111        2.46542       -1.10342
H          1.69860        1.57943        2.20370
C          1.97896       -2.53200       -0.36207
H          2.21394       -3.58144       -0.52260
C         -1.14067       -0.40671       -0.06188
C         -1.98784       -1.03952        0.86481
C         -1.70131        0.52684       -0.95048
C         -3.35227       -0.74001        0.91000
H         -1.56858       -1.76222        1.56073
C         -3.06581        0.82532       -0.90904
H         -1.06577        1.01249       -1.68548
C         -3.89642        0.19463        0.02312
H         -3.98853       -1.23498        1.63993
H         -3.47995        1.54789       -1.60826
H         -4.95772        0.42830        0.05678
19
PhEtOH_4F_001_opt_fr.log  Energy: -304578.3013426
C          2.16918       -0.07171        0.03627
C          1.37966       -1.20979        0.15269
C         -0.00662       -1.06056        0.07957
C         -0.58852        0.20270       -0.11142
C          0.24668        1.32090       -0.23191
C          1.63827        1.19580       -0.15514
C         -2.10106        0.35456       -0.15547
C         -2.74163        0.18441        1.21943
O         -2.71139       -0.63377       -1.00739
H          1.84441       -2.18044        0.29743
H         -0.64460       -1.93577        0.16488
H         -0.18905        2.30563       -0.38723
H          2.29622        2.05456       -0.24806
H         -2.34022        1.35863       -0.53619
H         -3.82821        0.30927        1.14826
H         -2.52858       -0.81410        1.61857
H         -2.34725        0.92921        1.91955
H         -2.30978       -0.56675       -1.89045
F          3.52915       -0.20977        0.10758
22
PhPrOH_003_opt_fr.log     Energy: -266975.3758519
C          2.83953        0.18500        0.26407
C          2.09708       -0.76349        0.97871
C          0.73940       -0.94424        0.70447
C          0.10440       -0.18380       -0.28998
C          0.85628        0.75830       -1.00405
C          2.21542        0.94621       -0.72839
C         -1.38034       -0.35928       -0.56217
O         -1.74098       -1.75063       -0.66491
H          3.89615        0.32613        0.47867
H          2.57710       -1.36067        1.75065
H          0.16434       -1.68193        1.25850
```

```
H          0.37582         1.35101        -1.78014
H          2.78455         1.68267        -1.29095
H         -1.62564         0.14938        -1.50765
H         -1.15855        -2.16892        -1.32134
C         -2.26621         0.22469         0.54510
H         -3.30599        -0.02829         0.30049
H         -2.02368        -0.28361         1.48766
C         -2.12098         1.74019         0.70741
H         -2.34635         2.26549        -0.23022
H         -2.81262         2.11354         1.47204
H         -1.10478         2.01882         1.01050
28
PhtBuOH_001_opt_fr.log    Energy: -316320.7210437
C          3.37814         0.12714         0.34136
C          2.64501        -1.02069         0.66143
C          1.30279        -1.12597         0.28513
C          0.67071        -0.08649        -0.41418
C          1.41917         1.05344        -0.74264
C          2.76049         1.16492        -0.36450
C         -0.79306        -0.18518        -0.81981
C         -1.81638         0.27113         0.26645
O         -1.13806        -1.53133        -1.19196
H          4.42263         0.20876         0.63266
H          3.11812        -1.83547         1.20480
H          0.73749        -2.01962         0.53252
H          0.94703         1.86145        -1.29745
H          3.32404         2.05713        -0.62761
H         -0.94538         0.47540        -1.68815
H         -0.49887        -1.83243        -1.85874
C         -1.73598        -0.60991         1.52531
H         -0.76536        -0.51244         2.02467
H         -2.50955        -0.30754         2.24270
H         -1.89356        -1.66617         1.28197
C         -3.23223         0.17648        -0.33668
H         -3.31684         0.78724        -1.24579
H         -3.48782        -0.85516        -0.59752
H         -3.97587         0.54281         0.38245
C         -1.53674         1.73630         0.64807
H         -2.30385         2.09325         1.34642
H         -0.56210         1.85318         1.13470
H         -1.55724         2.39320        -0.23176
```

6.4. Heteroaromatic systems

```
23
acridine_001_opt_fr.log   Energy: -348658.6375164
C          3.64490         0.66139         0.00006
C          2.48605         1.39653         0.00001
C          1.21258         0.74559        -0.00008
C          1.15850        -0.70012        -0.00017
C          2.39062        -1.42844         0.00006
C          3.59346        -0.76623         0.00012
C          0.00000         1.44331        -0.00008
C         -1.15850        -0.70012        -0.00006
C         -1.21258         0.74559        -0.00006
C         -2.48605         1.39653         0.00004
H         -2.51504         2.48411         0.00006
C         -3.64490         0.66139         0.00008
C         -3.59346        -0.76623         0.00007
C         -2.39062        -1.42844         0.00002
H         -0.00000         2.53214        -0.00002
H          4.61020         1.16098         0.00012
H          2.51504         2.48411         0.00002
H          2.33979        -2.51393         0.00010
H          4.52304        -1.33011         0.00021
H         -4.61020         1.16098         0.00015
H         -4.52304        -1.33011         0.00011
H         -2.33979        -2.51393         0.00001
N          0.00000        -1.38552        -0.00012
17
azepine_001_opt_fr.log    Energy: -205122.4083542
C         -1.85573         0.67848         0.30456
C         -1.85566        -0.67857         0.30471
C         -0.80261        -1.51866        -0.26108
C         -0.80271         1.51862        -0.26113
C          0.50448        -1.19187        -0.32382
C          0.50445         1.19194        -0.32370
H         -2.73983         1.19400         0.67895
H         -2.73969        -1.19412         0.67922
H         -1.09747        -2.48073        -0.67651
H         -1.09751         2.48078        -0.67639
H          1.22062        -1.86307        -0.80220
H          1.22059         1.86339        -0.80175
N          1.05329         0.00006         0.22334
C          2.50725        -0.00003         0.36095
H          3.02614        -0.00015        -0.61432
H          2.81875         0.88526         0.92327
H          2.81859        -0.88527         0.92343
15
Bzfuran_001_opt_fr.log    Energy: -240779.4716997
C          2.11861        -0.71576        -0.00011
C          2.14459         0.69486        -0.00006
C          0.96688         1.44201         0.00001
C         -0.25935         0.75572         0.00003
C         -0.24942        -0.65340        -0.00001
C          0.91148        -1.41932        -0.00008
H          3.05525        -1.26714        -0.00016
H          3.10358         1.20687        -0.00008
H          0.99531         2.52868         0.00004
H          0.87608        -2.50475        -0.00012
C         -1.65478         1.13112         0.00010
H         -2.07020         2.13035         0.00014
C         -2.35669        -0.03066         0.00010
H         -3.41615        -0.24635         0.00014
O         -1.53398        -1.13438         0.00002
18
Bzimidazole_001_opt_fr.log        Energy: -
263067.8916826
C          2.57712         0.02110         0.00001
C          2.03072        -1.28242        -0.00002
C          0.65173        -1.49436        -0.00002
C         -0.15791        -0.35367        -0.00001
C          0.37296         0.96085         0.00002
C          1.76216         1.15236         0.00003
H          3.65764         0.14044         0.00001
H          2.70052        -2.13865        -0.00004
H          0.23046        -2.49589        -0.00001
H          2.18128         2.15508         0.00005
N         -1.53257        -0.19052        -0.00004
C         -2.52442        -1.25324         0.00005
H         -2.41124        -1.87731        -0.89266
H         -2.41189        -1.87659         0.89334
H         -3.52191        -0.80826        -0.00051
C         -1.76005         1.16100        -0.00003
H         -2.77068         1.55039        -0.00004
N         -0.66287         1.88781        -0.00000
14
Bzoxazole_001_opt_fr.log  Energy: -250850.8336585
C          2.09632        -0.71396        -0.00011
C          2.11167         0.69657        -0.00007
C          0.93103         1.44258         0.00000
C         -0.27320         0.73108         0.00004
C         -0.25993        -0.67023        -0.00000
C          0.89795        -1.43566        -0.00008
H          3.03858        -1.25524        -0.00017
H          3.06791         1.21292        -0.00010
H          0.94016         2.52867         0.00004
H          0.87427        -2.52089        -0.00012
C         -2.29351         0.06655         0.00011
H         -3.37012        -0.04362         0.00006
O         -1.56995        -1.09461        -0.00000
N         -1.60759         1.16192         0.00015
14
Bzthiazole_001_opt_fr.log Energy: -453521.3565217
C         -2.29290        -0.88836         0.00011
C         -2.47808         0.50852         0.00005
C         -1.38888         1.37434        -0.00002
C         -0.09360         0.83318        -0.00003
C          0.08002        -0.57362         0.00003
C         -1.01491        -1.44523         0.00010
H         -3.15903        -1.54491         0.00017
H         -3.48638         0.91368         0.00006
H         -1.51917         2.45278        -0.00007
H         -0.87556        -2.52243         0.00015
C          2.11685         0.79295        -0.00011
H          3.14308         1.14780        -0.00016
N          1.08469         1.57390        -0.00009
S          1.79583        -0.94218        -0.00002
15
Bzthiophene_001_opt_fr.log        Energy: -
443452.5362141
C          2.31377        -0.88711        -0.00012
C          2.50588         0.50961        -0.00006
C          1.41879         1.37688         0.00002
C          0.11093         0.85292         0.00004
C         -0.06200        -0.55606        -0.00002
C          1.03137        -1.43169        -0.00011
H          3.17560        -1.54954        -0.00018
H          3.51608         0.91087        -0.00007
H          1.56850         2.45394         0.00006
H          0.88466        -2.50851        -0.00015
C         -1.15066         1.55265         0.00010
H         -1.23601         2.63467         0.00012
```

```
C         -2.22110        0.71371        0.00012
H         -3.27159        0.98091        0.00017
S         -1.76994       -0.98174        0.00002
9
furan_001_opt_fr.log      Energy: -144352.4451167
C         -1.09880       -0.34833       -0.00006
C         -0.71923        0.96077       -0.00005
C          0.71923        0.96078        0.00001
C          1.09880       -0.34832        0.00008
H         -2.05625       -0.84947       -0.00011
H         -1.37700        1.82025       -0.00009
H          1.37699        1.82025        0.00002
H          2.05625       -0.84946        0.00015
O          0.00000       -1.16137        0.00002
12
imidazole_001_opt_fr.log  Energy: -166641.7273572
C         -0.22409       -1.11240       -0.00197
C         -1.50574       -0.61055        0.00244
C         -0.19444        1.08914       -0.00156
N          0.60791       -0.01232       -0.00639
H          0.16673       -2.12033       -0.00343
H         -2.43888       -1.15898        0.00356
H          0.21509        2.09070       -0.00373
C          2.06459       -0.03384        0.00400
H          2.43519        0.99019       -0.08065
H          2.43228       -0.47149        0.93763
H          2.43860       -0.61739       -0.84255
N         -1.47805        0.76849        0.00235
19
indole_001_opt_fr.log     Energy: -252993.0910993
C          2.59722        0.01115       -0.00000
C          2.03525       -1.28644        0.00000
C          0.65453       -1.47511        0.00000
C         -0.15459       -0.33005       -0.00001
C          0.39030        0.98919        0.00001
C          1.78953        1.14554        0.00000
H          3.67905        0.12106       -0.00000
H          2.69253       -2.15259        0.00000
H          0.22267       -2.47229        0.00001
H          2.23067        2.13984        0.00001
N         -1.53066       -0.22048       -0.00004
C         -2.45800       -1.33771        0.00002
H         -2.31357       -1.95838       -0.89194
H         -2.31394       -1.95800        0.89232
H         -3.48031       -0.95292       -0.00029
C         -0.72547        1.89386        0.00002
H         -0.68975        2.97584        0.00003
C         -1.86338        1.12054       -0.00001
H         -2.90517        1.41499       -0.00002
16
indolizine_001_opt_fr.log Energy: -228309.8807190
C         -2.14243        0.69557       -0.00010
C         -2.09548       -0.73442       -0.00007
C         -0.89513       -1.38601       -0.00001
C          0.27927        0.75328       -0.00000
C         -0.97402        1.41906       -0.00007
H         -3.10350        1.20156       -0.00015
H         -3.01063       -1.31785       -0.00009
H         -0.78768       -2.46502        0.00002
H         -0.97971        2.50551       -0.00010
C          2.41412       -0.00493        0.00011
H          3.49678       -0.04335        0.00015
C          1.61515        1.16217        0.00001
H          1.95443        2.19001       -0.00004
C          1.57904       -1.11402        0.00012
H          1.79556       -2.17323        0.00019
N          0.27880       -0.66312        0.00002
17
isoquinoline_001_opt_fr.log        Energy: -
252238.5476414
C         -2.41751       -0.71347        0.00000
C         -1.23211       -1.41947       -0.00000
C          0.01059       -0.72875        0.00000
C          0.00777        0.70088       -0.00000
C         -1.22751        1.40316        0.00000
C         -2.41803        0.70708        0.00000
H          1.32955       -2.46326       -0.00001
H         -3.36530       -1.24608        0.00000
H         -1.23335       -2.50708       -0.00000
C          1.27417       -1.37743        0.00000
H         -1.21826        2.49101        0.00000
H         -3.36422        1.24192        0.00000
C          2.42443       -0.61984        0.00001
H          3.40247       -1.09507       -0.00000
N          2.43290        0.74597       -0.00000
C          1.26667        1.36483       -0.00000
H          1.28803        2.45496       -0.00000
8
isoxazole_001_opt_fr.log  Energy: -154402.1635228
C          1.10507        0.23866        0.00002
C          0.12079        1.17938        0.00003
H          2.18599        0.28912        0.00003
H          0.22167        2.25506        0.00007
O          0.57282       -0.99861       -0.00002
C         -1.07322        0.40226       -0.00001
H         -2.10262        0.74087       -0.00001
N         -0.82907       -0.88828       -0.00003
8
oxazole_001_opt_fr.log    Energy: -154423.8827418
C          0.93455        0.65050       -0.00007
C          0.92160       -0.70624       -0.00005
C         -1.10643       -0.05807        0.00008
H          1.70027        1.41174       -0.00013
H          1.75402       -1.39601       -0.00011
H         -2.18242        0.05109        0.00012
O         -0.36959        1.08080        0.00000
N         -0.40193       -1.14720        0.00005
13
oxepine_001_opt_fr.log    Energy: -192913.4968275
C         -1.43592        0.68022        0.15071
C         -1.43592       -0.68024        0.15070
C         -0.30466       -1.50575       -0.24376
C         -0.30467        1.50574       -0.24376
C          0.98785       -1.15041       -0.13158
C          0.98784        1.15042       -0.13158
H         -2.36989        1.19806        0.36262
H         -2.36988       -1.19809        0.36261
H         -0.51162       -2.47519       -0.69422
H         -0.51165        2.47518       -0.69422
H          1.81386       -1.75750       -0.49689
H          1.81384        1.75752       -0.49689
O          1.39603        0.00001        0.54407
22
phenazine_001_opt_fr.log  Energy: -358720.7175760
C          3.57559        0.71503        0.00010
C          2.39744        1.41835        0.00006
C          1.14759        0.72444       -0.00011
C          1.14759       -0.72444       -0.00014
C          2.39744       -1.41835        0.00006
C          3.57559       -0.71503        0.00012
C         -1.14759       -0.72443       -0.00009
C         -1.14759        0.72443       -0.00012
C         -2.39744        1.41835        0.00007
H         -2.37896        2.50480        0.00008
C         -3.57559        0.71503        0.00012
C         -3.57559       -0.71503        0.00010
C         -2.39744       -1.41835        0.00003
H          4.52474        1.24454        0.00015
H          2.37896        2.50480        0.00005
H          2.37896       -2.50480        0.00009
H          4.52474       -1.24454        0.00021
H         -4.52474        1.24454        0.00019
H         -4.52474       -1.24454        0.00015
H         -2.37896       -2.50480        0.00002
N          0.00000       -1.42162       -0.00015
N          0.00000        1.42162       -0.00017
26
phenothiazine_001_opt_fr.log       Energy: -
599276.3615879
C         -3.51477        0.71873       -0.66788
C         -2.31532        1.33778       -0.30194
C         -1.21594        0.57408        0.12657
C         -1.34830       -0.83097        0.14990
C         -2.53527       -1.44547       -0.25444
C         -3.63166       -0.67206       -0.64881
C          1.34831       -0.83099        0.14995
C          1.21593        0.57406        0.12658
C          2.31531        1.33776       -0.30196
H          2.24262        2.41843       -0.35397
C          3.51476        0.71872       -0.66791
C          3.63166       -0.67206       -0.64881
C          2.53527       -1.44549       -0.25442
H         -4.35363        1.33448       -0.98246
H         -2.24270        2.41845       -0.35391
H         -2.60578       -2.53028       -0.24296
H         -4.55962       -1.15525       -0.94216
H          4.35361        1.33448       -0.98248
H          4.55962       -1.15527       -0.94214
H          2.60581       -2.53029       -0.24294
S         -0.00002       -1.81165        0.78852
N          0.00002        1.16961        0.53654
C          0.00004        2.59672        0.83724
H          0.00011        3.22928       -0.06343
H         -0.88309        2.83515        1.43491
```

```
H          0.88314        2.83510        1.43499
26
phenoxazine_001_opt_fr.log         Energy: -
396607.1560190
C         -3.57514        0.36017       -0.40729
C         -2.41543        1.12877       -0.23391
C         -1.19721        0.51621        0.09275
C         -1.17379       -0.88891        0.20256
C         -2.31327       -1.65561        0.01109
C         -3.53194       -1.02781       -0.28413
C          1.17379       -0.88892        0.20261
C          1.19720        0.51620        0.09275
C          2.41542        1.12876       -0.23391
H          2.46892        2.20514       -0.35317
C          3.57515        0.36014       -0.40728
C          3.53192       -1.02783       -0.28418
C          2.31326       -1.65562        0.01112
H         -4.50973        0.86106       -0.64615
H         -2.46890        2.20515       -0.35319
H         -2.23943       -2.73490        0.11032
H         -4.42918       -1.62507       -0.42039
H          4.50973        0.86105       -0.64612
H          4.42915       -1.62510       -0.42048
H          2.23938       -2.73491        0.11033
N          0.00000        1.21587        0.32845
C          0.00005        2.66905        0.35838
H          0.00014        3.11307       -0.64881
H         -0.88059        3.02029        0.90133
H          0.88061        3.02025        0.90149
O         -0.00001       -1.53560        0.55529
11
pyridine_001_opt_fr.log   Energy: -155816.5946381
C         -1.14734       -0.72283       -0.00022
C         -1.20064        0.67400       -0.00012
C          0.00042        1.38673        0.00009
C          1.20104        0.67331        0.00022
C          1.14690       -0.72349        0.00013
N         -0.00042       -1.42102       -0.00010
H          0.00071        2.47397        0.00016
H         -2.06442       -1.30901       -0.00037
H         -2.16008        1.18369       -0.00022
H          2.16081        1.18239        0.00036
H          2.06363       -1.31022        0.00019
10
pyrimidine_001_opt_fr.log Energy: -165882.7476615
C          0.61918        1.18953       -0.00023
C          1.35837        0.00701       -0.00012
C          0.63185       -1.18310        0.00007
C         -1.31758       -0.00675        0.00013
N         -0.72282        1.19241       -0.00013
H          1.13049       -2.15016        0.00030
H          1.10817        2.16149       -0.00043
H          2.44387        0.01262       -0.00013
H         -2.40453       -0.01329        0.00045
N         -0.70989       -1.19967        0.00024
13
pyrrole_001_opt_fr.log    Energy: -156567.1912215
C         -0.17246       -1.12012       -0.01460
C         -1.49550       -0.71312        0.01576
C         -1.49558        0.71301        0.01595
C         -0.17258        1.12015       -0.01473
N          0.62460        0.00006       -0.04109
H          0.26761       -2.10853       -0.02341
H         -2.35985       -1.36576        0.02413
H         -2.36000        1.36557        0.02448
H          0.26738        2.10861       -0.02358
C          2.07844        0.00001        0.02677
H          2.46740        0.88982       -0.47564
H          2.42355       -0.00298        1.06766
H          2.46773       -0.88671       -0.48089
16
quinazoline_001_opt_fr.log         Energy: -
262306.0723183
C          2.39938        0.69700       -0.00012
C          1.21824        1.40867       -0.00026
C         -0.01890        0.71334       -0.00012
C         -0.03890       -0.71405        0.00015
C          1.19010       -1.42231        0.00029
C          2.38080       -0.72356        0.00016
H         -1.34354        2.44062       -0.00047
H          3.35241        1.21906       -0.00022
H          1.21704        2.49637       -0.00046
C         -1.28476        1.35212       -0.00023
H          1.16704       -2.50839        0.00050
H          3.32324       -1.26528        0.00026
N         -2.42396        0.68430       -0.00017
N         -1.22592       -1.39542        0.00026
C         -2.32825       -0.67641        0.00007
H         -3.27329       -1.21340        0.00008
17
quinoline_001_opt_fr.log  Energy: -252239.4745796
C         -2.41598        0.70360        0.00002
C         -1.23158        1.41015        0.00020
C          0.01465        0.72593        0.00013
C          0.02721       -0.70757       -0.00013
C         -1.20959       -1.40799       -0.00030
C         -2.40303       -0.71593       -0.00023
H          1.29290        2.48244        0.00049
H         -3.36607        1.23175        0.00007
H         -1.23366        2.49821        0.00038
C          1.26698        1.39474        0.00030
H         -1.18476       -2.49443       -0.00049
H         -3.34488       -1.25890       -0.00037
C          2.33229       -0.76216       -0.00002
C          2.42732        0.65403        0.00023
H          3.24214       -1.36104       -0.00012
H          3.40430        1.12884        0.00035
N          1.19149       -1.42796       -0.00021
8
thiazole_001_opt_fr.log   Energy: -357094.2315163
C         -0.08889       -1.22411       -0.00008
C         -1.29896       -0.58761       -0.00005
C         -0.01618        1.21130        0.00008
H          0.11401       -2.28784       -0.00015
H         -2.26248       -1.08478       -0.00009
H          0.27216        2.25721        0.00015
N         -1.25116        0.79213        0.00005
S          1.19116       -0.05169        0.00000
13
thiepine_001_opt_fr.log   Energy: -395585.4528708
C          1.66437        0.67991       -0.27926
C          1.66437       -0.67991       -0.27925
C          0.66125       -1.55998        0.30457
C          0.66125        1.55998        0.30457
C         -0.67134       -1.36756        0.36401
C         -0.67134        1.36756        0.36401
H          2.56079        1.17606       -0.65031
H          2.56079       -1.17606       -0.65031
H          1.04350       -2.47717        0.75654
H          1.04350        2.47717        0.75654
H         -1.31126       -2.07353        0.89046
H         -1.31126        2.07353        0.89047
S         -1.52734        0.00000       -0.41658
9
thiophene_001_opt_fr.log  Energy: -347026.1060367
C         -0.01117       -1.24543       -0.00007
C         -1.27546       -0.71624       -0.00008
C         -1.27546        0.71623        0.00003
C         -0.01118        1.24543        0.00006
H          0.27951       -2.28930       -0.00012
H         -2.17561       -1.32282       -0.00014
H         -2.17562        1.32281        0.00006
H          0.27950        2.28931        0.00013
S          1.20199        0.00000        0.00002
```

7. References

[1] J. R. C. G. Gaussion 09 R. D. 01, M. J. Frisch, G. W. Trucks, H. B. Schlegel, G. E. Scuseria, M. A. Robb, H. P. H. A. F. Scalmani V. Barone, B. Mennucci, G. A. Petersson, H. Nakatsuji, M. Caricato, X. Li, J. H. M. Ishida Izmaylov J. Bloino, G. Zheng, J. L. Sonnenberg, M. Hada, M. Ehara, K. Toyota, R. Fukuda, F. O. M. Bearpark T. Nakajima Y. Honda, O. Kitao, H. Nakai, T. Vreven, J. A. Montgomery Jr. ,. J. E. Peralta, A. R. J. J. J. Heyd E. Brothers, K. N. Kudin, V. N. Staroverov, R. Kobayashi, J. Normand, K. Raghavachari, J. B. C. V. Bakken C. Burant S. S. Iyengar, J. Tomasi, M. Cossi, N. Rega, J. M. Millam, M. Klene, J. E. Knox, C. P. J. W. C. Adamo J. Jaramillo, R. Gomperts, R. E. Stratmann, O. Yazyev, A. J. Austin, R. Cammi, J. J. D. S. Dapprich Ochterski R. L. Martin, K. Morokuma, V. G. Zakrzewski, G. A. Voth, P. Salvador, WaÖ. F. 2010. A. D. Daniels J. B. Foresman, J. V. Ortiz, J. Cioslowski, D. J. Fox, Gaussian, Inc., Wallingford CT, **2009**.

[2] C. Lee, W. Yang, R. G. Parr, *Physical Review B* **1988**, *37*, 785–789.

[3] S. Grimme, J. Antony, S. Ehrlich, H. Krieg, *The Journal of Chemical Physics* **2010**, *132*, 154104.

[4] R. Ditchfield, W. J. Hehre, J. A. Pople, *The Journal of Chemical Physics* **1971**, *54*, 724–728.

[5] A. V. Marenich, C. J. Cramer, D. G. Truhlar, *The Journal of Physical Chemistry B* **2009**, *113*, 6378–6396.

[6] Y. Zhao, D. G. Truhlar, *Theor Chem Account* **2008**, *120*, 215–241.

[7] F. Weigend, R. Ahlrichs, *Phys. Chem. Chem. Phys.* **2005**, *7*, 3297.

[8] E. D. Glendenning, A. E. Reed, J. E. Carpenter, F. Weinhold, **2001**.

[9] A. Tkatchenko, M. Scheffler, *Phys. Rev. Lett.* **2009**, *102*, 073005.

[10] T. Lu, F. Chen, *J Comput Chem* **2012**, *33*, 580–592.

[11] T. Lu, *The Journal of Chemical Physics* **2024**, *161*, 082503.

[12] R Core Team (2025). R: A Language and Environment for Statistical Computing. R Foundation for Statistical Computing, Vienna, Austria. https://www.R-project.org/., **2025**.

[13] T. D. Goddard, C. C. Huang, E. C. Meng, E. F. Pettersen, G. S. Couch, J. H. Morris, T. E. Ferrin, *Protein Science* **2018**, *27*, 14–25.

[14] E. F. Pettersen, T. D. Goddard, C. C. Huang, E. C. Meng, G. S. Couch, T. I. Croll, J. H. Morris, T. E. Ferrin, *Protein Science* **2021**, *30*, 70–82.

[15] E. C. Meng, T. D. Goddard, E. F. Pettersen, G. S. Couch, Z. J. Pearson, J. H. Morris, T. E. Ferrin, *Protein Science* **2023**, *32*, e4792.

[16] A. J. Schaefer, V. M. Ingman, S. E. Wheeler, *J Comput Chem* **2021**, *42*, 1750–1754.

[17] V. M. Ingman, A. J. Schaefer, L. R. Andreola, S. E. Wheeler, *WIREs Comput Mol Sci* **2021**, *11*, e1510.

[18] 2025. Schrödinger Release 2025-2: Meastro LLC New York, NY, **n.d.**

[19] M. Gussoni, M. Rui, G. Zerbi, *Journal of Molecular Structure* **1998**, *447*, 163–215.

[20] D. Umadevi, G. N. Sastry, *J. Phys. Chem. C* **2011**, *115*, 9656–9667.

[21] M. R. Crittall, H. S. Rzepa, D. R. Carbery, *Organic Letters* **2011**, *13*, 1250–1253.
